# An Exploration of Machine Learning Methods in Human Biomonitoring

**DOI:** 10.3390/ijerph23050680

**Published:** 2026-05-20

**Authors:** Kavita Singh, Jiazhou Bi, Malo Musende, Sean P. Collins, Michael M. Borghese, Janice M.Y. Hu, Tyler Pollock, Annie St-Amand, Deirdre Hennessy, David L. Buckeridge

**Affiliations:** 1 Environmental Health Science and Research Bureau, Healthy Environments and Consumer Safety Branch, Health Canada, Ottawa, ON K1A 0K9, Canada; jiazhou.bi@hc-sc.gc.ca (J.B.); malo.musende@mail.mcgill.ca (M.M.); michael.borghese@hc-sc.gc.ca (M.M.B.); janice.hu@hc-sc.gc.ca (J.M.Y.H.); tyler.pollock@hc-sc.gc.ca (T.P.); annie.st-amand@hc-sc.gc.ca (A.S.-A.); 2 Department of Epidemiology and Biostatistics, McGill University, Montreal, QC H3A 1G1, Canada; david.buckeridge@mcgill.ca; 3 Existing Substances Risk Assessment Bureau, Healthy Environments and Consumer Safety Branch, Health Canada, Ottawa, ON K1A 0K9, Canada; sean.p.collins@hc-sc.gc.ca; 4 Health Analysis and Modelling Division, Analytical Studies and Modelling Branch, Statistics Canada, Ottawa, ON K1A 0T6, Canada; deirdre.hennessy@statcan.gc.ca

**Keywords:** biomonitoring, artificial intelligence, machine learning

## Abstract

**Highlights:**

**Public health relevance—How does this work relate to a public health issue?**

- Chemical exposures are ubiquitous and can have adverse health impacts on individuals and populations. These exposures occur alongside many other determinants of health and disease risk factors. Advanced analytical methods are required to analyze multiple variables simultaneously and to fully understand their combined impacts.
- In this work, we explored how machine learning can be used to analyze data on environmental chemicals measured in people. We provide a detailed categorization of methods and how they can address issues of public health concern, such as chemical mixtures, exposure factors, and health outcomes. We also surveyed human biomonitoring programs about machine learning and artificial intelligence uses, implementation, and barriers.

**Public health significance—Why is this work of significance to public health?**

- This work provides researchers, regulators, and policymakers with evidence about machine learning methods to analyze chemical exposure data. These methods will continue to advance and be incorporated into work processes. Familiarity with these methods will help to navigate research outputs and support responsible adoption.
- The survey of programs demonstrated that a few key barriers, such as a lack of technical expertise, may be limiting wider adoption of machine learning and artificial intelligence in the field. Resources devoted to education, upskilling, and development of collaborations between content experts and analysts should encourage greater awareness and informed implementation.

**Public health implications—What are the key implications or messages for practitioners, policymakers, and/or researchers in public health?**

- Machine learning can accommodate many variables and model non-linear relationships, thereby providing insights into complex exposure–response relationships and chemical mixtures. They can also help identify factors that place populations at higher risk of chemical exposures.
- Machine learning methods also have limitations and may not be the best approach in all circumstances. These include difficulty with incorporating complex survey weights to represent a population, limited ability to quantify the magnitude of effects, and challenges with the interpretability of outputs.

**Abstract:**

Artificial intelligence (AI) is being broadly integrated into processes to manage and analyze large amounts of data accurately and efficiently. In this work, we explored how AI methods, in particular machine learning (ML), are being implemented in human biomonitoring using a mixed methodology approach that consisted of: (1) a scoping literature review and (2) an international scan of biomonitoring programs to contextualize current practices and perceptions from researchers in the field. We synthesized findings from the review according to the year of publication, biomonitoring study and location, study outcomes, and the most frequent ML methods. We additionally categorized all published ML methods from the review according to three dimensions (paradigm, type of task, and model structure), mapped studies to biomonitoring themes and other applications, and provided details for the more commonly applied ML methods. The international scan was administered through a 30-question online survey and gathered information on current uses, perspectives, and barriers related to AI. Scoping review: We found 286 studies that applied a ML method to human biomonitoring data. Eighty-two ML methods were identified, with the most common being supervised approaches. ML was predominantly applied to predict health-related outcomes based on chemical exposure. International scan: The survey yielded 30 responses from programs across 15 countries. Approximately 27% of respondents reported implementing AI-related methods in the collection and analysis of biomonitoring data, and the primary barrier to adopting these methods was a lack of technical expertise (80%). This exploratory work provides an integrated understanding of ML applications in the human biomonitoring field. ML clearly holds promise for furthering our understanding of chemical exposure in people and will likely undergo continued growth in applications.

## 1. Introduction

Artificial intelligence (AI) has developed rapidly over the past two decades due to the emergence of sophisticated and efficient algorithms and advances in computing capabilities. It has demonstrated results in processing large quantities of data to detect patterns and produce meaningful insights. The benefits of AI have been observed in various fields, including healthcare (e.g., diagnostics [1] and drug discovery [2]), public health surveillance [3], and environmental monitoring (e.g., air pollution [4], marine pollution [5], extreme heat [6], and climate change [7]). In the era of “big data”, workflows that incorporate AI systems will be required to analyze data efficiently and optimally.

AI is an emerging tool for use in human biomonitoring, but the breadth of its use to date is not well documented or understood. We were interested in exploring the capabilities of AI, in particular machine learning (ML), for leveraging insights from human biomonitoring data. Human biomonitoring is the measurement of chemical concentrations and their metabolites in matrices, such as blood and urine. These data provide important information on exposure to legacy and emerging chemicals of concern and allow for the tracking of chemical exposure over time [8]. Human biomonitoring efforts range in scale, from large national-level programs that monitor chemicals, in general, populations, to more targeted efforts that focus on smaller and specific subpopulations. In addition to chemical concentrations, biomonitoring studies routinely collect other data about people and their health that can assist with identifying the sources of chemical exposures and potential health effects.

The Government of Canada conducts ongoing monitoring of chemical exposures in people living in Canada through the Canadian Health Measures Survey (CHMS). The CHMS is a national-level, direct health measures survey conducted in two-year cycles [9]. From 2007 to 2024, the program has collected data from more than 35,000 participants over seven cycles, making it one of the largest human biomonitoring datasets in Canada. The CHMS contains a rich source of information on over 300 environmental chemical biomarkers, as well as sociodemographic characteristics, lifestyle and behavioral factors, and various health indicators and physical measures. It has resulted in a multidimensional dataset with great potential to improve our understanding of chemical exposures in people living across Canada. Like many similar national-level biomonitoring programs, analyses of CHMS data are generally conducted on a chemical-by-chemical and factor-by-factor basis, with few exceptions. This approach is time-consuming, does not make full use of the data, and does not always address real-world exposure (RWE) scenarios. We anticipate that the application of AI tools, such as ML, will help to further our understanding of chemical exposures.

ML is a subfield of AI that uses algorithms to make predictions or decisions from data through experience and prioritizes predictive performance over understanding the underlying data-generating process that statistical modeling seeks to characterize. While ML itself is not new (e.g., linear and logistic regression are ML methods), advances in algorithms and computing abilities have paved the way for a subsequent generation of methods that have led to significant progress in data analysis capabilities. Despite resources and efforts spent on developing new algorithms, optimizing the architectures, and making ML more interpretable, little attention has been paid to systematically categorizing the available methods. While newer methods are being invented and developed frequently, researchers can find it challenging to determine what specific ML method(s) are best suited for their research and surveillance needs. This concern is prominent because many ML methods are designed to perform specific tasks and have different strengths, weaknesses, and underlying assumptions.

While surveys about utilization of and perspectives on AI have been conducted in healthcare settings [10,11,12], to our knowledge, this is the first attempt to administer a survey on the applications of ML in the field of human biomonitoring. Reviews of AI applications have been conducted in other environmental fields [4,5,6,7]. A review about the applications of AI and ML on the impacts of air pollution on human health identified the use of regressions, random forests, neural networks, and deep learning to predict hospitalizations and emergency department visits [4]. Another review examined ML applications to model human health effects of extreme heat events and found that random forest was the most frequently applied ML method to predict all-cause mortality or heat-related illness [6]. Other reviews have examined AI applications more broadly, providing information about areas in which it has been applied rather than focusing on specific methods, such as in the fields of marine pollution [5] and climate change [7]. AI is also increasingly being adopted in environmental exposure assessments [13,14,15]. Iyer et al. conducted a narrative review of geospatial AI and how it has been used to derive information from spatial data to inform exposure modeling [13]. Whereas biomonitoring is a direct method used to assess chemical exposures, exposure modeling is an indirect method that estimates exposures based on environmental data, including data from geographic information systems. A review of the use of ML to elucidate early life environmental exposures (e.g., chemicals, air pollution, and built environment) and child health found that most methods focused on mixture analyses [14].

In this work, we sought to explore how AI methods (with a focus on ML) have been used in human biomonitoring research and surveillance to characterize and understand their advantages, limitations, and challenges. To this end, we conducted: (1) a scoping literature review of human biomonitoring studies to identify ML methods that have been applied for quantitative data analysis and (2) an international scan of biomonitoring groups to gauge the degree of implementation of AI-related methods to date (including unpublished work) and perspectives on their applications. This was a mixed methodology approach that allowed for a detailed collation of information from the literature, along with contextualization of current practices and perceptions from researchers in the field.

## 2. Materials and Methods

### 2.1. Literature Review

We identified studies that applied ML methods to human biomonitoring data by conducting literature searches in multiple databases and gray literature sources. Searches were run in Medline, Embase, and Global Health through the Ovid platform, as well as the Scopus database (see Supplementary Materials for the search strategies). A modified version of the University of Alberta’s ML search filter was applied to the search strategy [16]. Duplicates were removed from the results using the Systematic Review Accelerator’s de-duplication tool [17]. The searches were performed to an end date of 4 August 2025.

The records were imported, screened, and extracted in Covidence, an online review platform. Titles and abstracts were initially screened, followed by full texts by a single reviewer (KS). An English language study was deemed eligible if it met the following inclusion criteria:

- A human population exposed to at least one chemical, and
- The chemical was measured in a biological matrix, such as blood or urine (i.e., individual-level human biomonitoring data available), and
- At least one ML method was applied to the human biomonitoring data (e.g., unsupervised methods such as k-means, hierarchical clustering, and principal component analysis, and supervised methods such as regularized regressions, k-nearest neighbors, tree-based methods, naïve Bayes, support vector machines, boosting algorithms, and deep learning).

Studies that applied traditional statistical techniques only, such as analysis of variance (ANOVA), t-tests, and simple linear or logistic regression, were excluded. However, if such traditional statistical methods were part of a broader ML testing framework (i.e., as described by the study’s objectives and/or handling of data, such as partitioning into training and test sets), the study was included. In the review, all included studies that used simple linear or logistic regression applied these methods alongside other ML approaches.

The following data were extracted from the included studies:

- Author, country, study design, and name of biomonitoring survey;
- Sample size and population description;
- Chemical class, number of chemicals analyzed, and specific chemicals;
- Biological matrix of chemical measurement;
- Outcome (categorized into one of the following: birth outcome, disease, health-based lab/measurement, questionnaires for behavioral and cognitive assessments, mortality, chemical exposures, and other. The other category of outcome did not clearly fall under a disease or health-based lab/measurement and included the following: genetic and immunological markers, oxidative/DNA damage, gut microbiome, metabolome, proteins, neurophysiological dynamics, sex steroids, semen quality, fatty acids and blood cell fractions, dietary contaminants, occupation type, sleep quality, precocious puberty, timing of menarche, age at menopause, reproductive lifespan, biologic aging (leukocyte telomere length), accelerated aging, difference between phenotypical, and chronological ages, accelerated bone maturation, and frailty.
- ML analyses performed and their intended purpose.

### 2.2. Categorization of ML Algorithms

We categorized the ML methods identified in the literature review based on three distinct dimensions, which have been used in prior studies [18,19]:

(1) Learning paradigm (how the method learns);
(2) Type of task (the intended function of the method);
(3) Model structure (the method’s architecture).

Dimension 1—Learning paradigm: The first dimension of categorization defines how the ML method learns a model from the data and has two primary classes of methods: supervised and unsupervised. Although additional classes exist, such as semi-supervised learning, self-supervised learning, and reinforcement learning, these classes of methods were beyond the scope of this work, as we did not identify their applications for human biomonitoring. A supervised ML method learns about the data from paired labels of predictor–response variables (known as the feature–target in ML [20]). These labels are often considered to be the “ground truth” of the dataset. These labels are generally provided by a human, and thus the method is “supervised”. In an unsupervised learning environment, the machine is not provided with any labeled data. Instead, the ML model will examine the dataset based on certain decisions and criteria and find underlying patterns within the dataset [21]. Some methods can be used in both supervised and unsupervised learning tasks.

Dimension 2—Type of task: ML methods can perform different tasks to address various types of research questions. We included four core tasks [22]: classification, regression, clustering, and dimensionality reduction. We also included tasks for quantifying variable (feature) importance, because knowing which variables contribute to predictions about human biomonitoring outputs is important for the interpretability and transparency of ML models. Furthermore, we included variable (feature) selection as a sub-task under dimensionality reduction. These six tasks are not mutually exclusive, and many methods can be used for more than one task.

Dimension 3—Model structure: Model structure refers to the mathematical form or structure that the ML method uses to learn and represent the relationship between variables in the data. We identified 19 structural types, which are not mutually exclusive: linear models, neural networks, graph models, kernel methods, Bayesian models, tree-based, clustering, ensemble, dimensionality reduction, instance-based, mathematical and statistical, model interpretation, rule-based, spline-based, online learning, audio compression, evolutionary, meta-learning, and additive models [23,24,25,26,27,28,29,30,31,32].

### 2.3. Mapping of Studies to Biomonitoring Themes

To gain a deeper understanding of the types of biomonitoring questions addressed by the ML methods, we mapped the studies according to the framework provided in Table 1. Leist et al. mapped ML approaches, in the social and health sciences, to overarching research purposes (description, prediction, and causal inference) [33]. A similar approach was used here, where we classified questions that are often asked in the human biomonitoring field into themes, which we classified into one of two broad categories: description or association/prediction. Human biomonitoring is primarily concerned with characterizing the internal levels of chemicals in people. As such, questions that arise from human biomonitoring surveys are often descriptive in nature, relating to chemicals, exposure factors, and mixtures. The collection of other variables in biomonitoring surveys, such as sociodemographic, lifestyle, and health-related factors, allows for additional questions of an analytic or etiological nature, such as relationships (i.e., associations or predictions) between chemical exposure and health outcomes or, alternatively, relationships between other factors and chemical exposures. These analytic questions are provided under the association/prediction category in Table 1.

### 2.4. International Scan

A total of 161 individuals from approximately 109 unique biomonitoring programs and research groups were invited to participate in a 30-question online survey. The contacts were identified through professional networks, colleague referrals, and public program listings, and represented various program types (i.e., national, state/provincial, and academic). Primary outreach was conducted via direct emails, with additional recruitment occurring through email forwarding by the initial contacts. If no response was received after one reminder email, follow-up efforts included reaching out to colleagues or collaborators within the same organization or research group.

The survey was hosted on the Government of Canada’s GC Forms platform and was designed to be brief, user-friendly, and accessible to respondents across a range of scientific and policy backgrounds. The survey included a mix of closed- and open-ended questions, allowing participants to share both structured responses and detailed perspectives (see Supplementary Materials for a copy of the survey).

The first seven questions (Q1 to Q7) asked about the respondent’s biomonitoring program and datasets, role in the program, and familiarity with AI. The next eight questions (Q8 to Q15) asked if AI methods have been implemented in the respondent’s biomonitoring program, plans for implementation, collaborations, and perspectives on AI. An additional 15 questions (Q16 to Q30) asked respondents for more details about any AI methods that have been implemented. Skip logic was applied based on the response to Question 8, which asked whether or not respondents had implemented AI. Respondents who selected “No” to Question 8 were presented with Questions 9–15 but were not shown Questions 16–30. Respondents who selected “Yes” to Question 8 were eligible to answer all 30 questions.

A link to the survey was distributed via email with an introductory message outlining the study’s purpose, confidentiality protections, and participants’ rights to withdraw at any time. Participation in the survey was fully voluntary, responses were kept confidential, and all data were handled in accordance with established privacy and security protocols. Ethics approval was obtained from the Health Canada Research Ethics Board and McGill University. All survey data were stored securely on internal Health Canada platforms and anonymized for analysis and reporting.

Raw responses were imported into Microsoft Excel for cleaning and analysis and tabulated as frequencies for graphical presentation. A thematic qualitative analysis was conducted for free-text responses. The themes were initially identified by one researcher (MM). ChatGPT (OpenAI GPT-4o model) was used as a secondary analytical aid to validate and refine emerging themes, ensure completeness of thematic coverage and interpretations, and support clarity in theme labeling and summary language. All themes were subsequently reviewed and finalized by a researcher.

## 3. Results

### 3.1. Literature Review

#### 3.1.1. Study Selection

**Figure 1:**
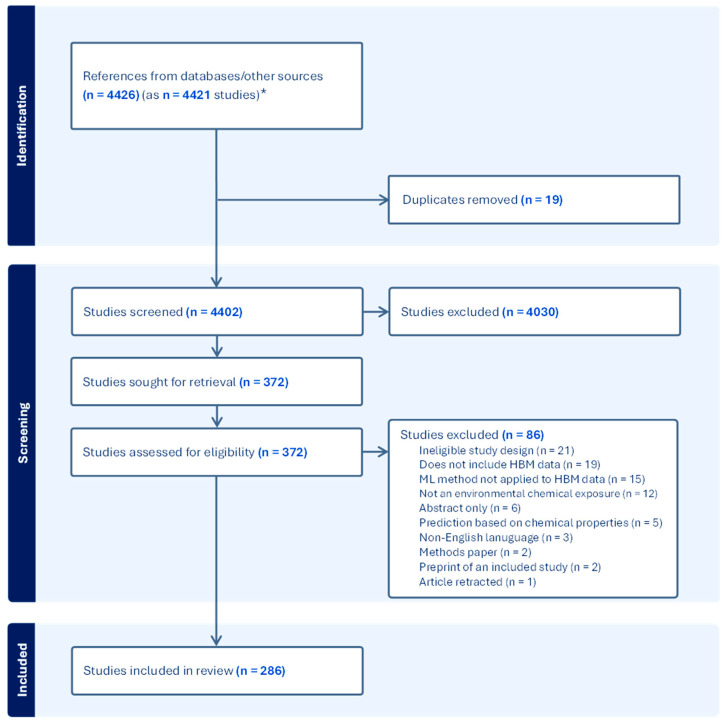
PRISMA diagram of study selection. Abbreviations: HBM = human biomonitoring; * 5 references were merged because they referred to the same study (e.g., an article preprint and article corrections).

The literature searches identified 4413 records, and an additional 13 records were identified from other sources (i.e., from reference lists and expert nomination), for a total of 4426 records. The 4426 records corresponded to 4402 unique studies after removing duplicates and the linking of companion articles (Figure 1). Upon title and abstract screening, 4030 records were excluded, leaving 372 studies for full-text review. Of these, 86 studies were excluded after review, primarily due to ineligible study design (e.g., review article, prediction at an aggregate geographic level rather than at the individual level, and viewpoints or descriptions of talks) or not including human biomonitoring data. A total of 286 studies were deemed relevant.

#### 3.1.2. Overview of Studies

**Figure 2:**
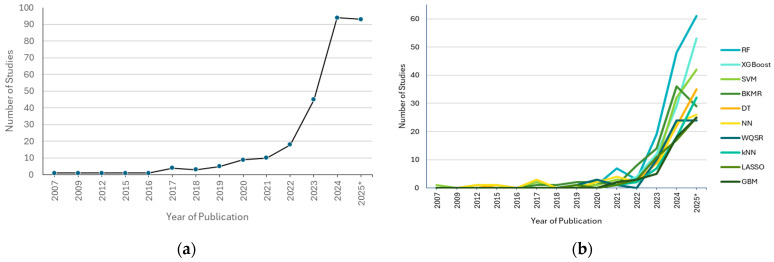
Number of studies using at least one ML method to analyze human biomonitoring data by year of publication. (**a**) Total number of studies published by year. (**b**) The top 10 ML methods and their publication in studies by year. * Search included articles published up to 4 August 2025. Abbreviations: BKMR = Bayesian kernel machine regression; DT = decision tree; GBM = gradient boosting machine; kNN = k-nearest neighbors; LASSO = least absolute shrinkage and selection operator; NN = neural network; RF = random forest; SVM = support vector machine; WQSR = weighted quantile sum regression; XGBoost = extreme gradient boosting.

Figure 2 shows the number of studies that have used at least one ML method to analyze human biomonitoring data, published by year. Beginning in approximately 2020, the use of ML methods has grown dramatically. The searches covered only part of 2025, which is why there is a slight dip for that year. Individual ML methods, as shown in panel b, also demonstrated corresponding increases by year, with the random forest and extreme gradient boosting (XGBoost) methods seeing the largest growth in use. Random forest and XGBoost are well-established tree-based methods that offer several advantages, such as good interpretability, reliability, and ability to handle tabular data efficiently, which have likely contributed to the increasing uptake of these methods in human biomonitoring.

The included studies were based on biomonitoring data from large-scale, national-level biomonitoring programs, as well as several smaller study cohorts. Table 2 provides the national-level biomonitoring programs represented in this review.

**Figure 3:**
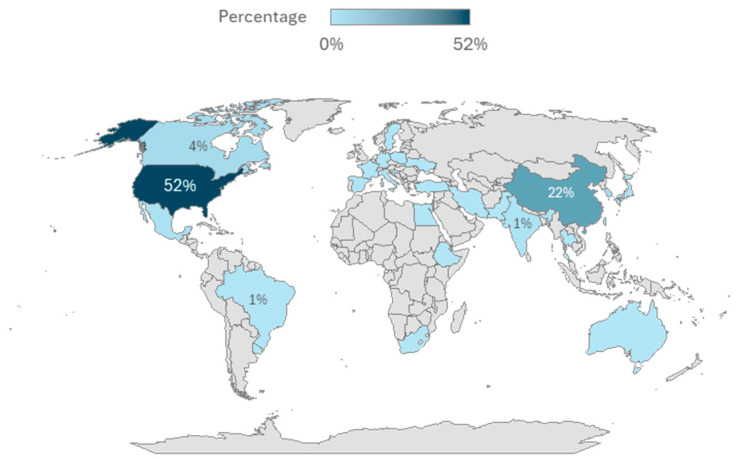
Country of human biomonitoring study.

More than half of all studies (n = 149, 52%) were based on biomonitoring surveys conducted in the United States (Figure 3). Most of these studies (n = 136, 49%) examined data from the National Health and Nutrition Examination Survey (NHANES), the national human biomonitoring survey of adults and children in the United States (Table 2). The NHANES data are publicly available, which makes its biomonitoring data readily accessible for analysis and research purposes. The second largest number of studies (n = 64, 23%) recruited participants from China, which included a study from the National Human Biological Monitoring Project of China and several additional smaller cohorts. The third largest number of studies came from South Korea (n = 12, 4%), of which eight studies used data from national-level biomonitoring programs (Table 2). There were 11 studies from Canada, which used data from the CHMS (1 study), the Maternal-Infant Research on Environmental Chemicals (MIREC) pan-Canadian birth cohort (6 studies), the Alberta Pregnancy Outcomes and Nutrition (APrON) cohort (3 studies), and the Qanuilirpitaa? study of Nunavik Inuit (1 study). Other countries were represented by much smaller numbers of studies.

The sample sizes of the studies ranged from 11 to 807,694, with most studies including an adult population exclusively (n = 199, 70%). A smaller number of studies focused exclusively on children/adolescents (n = 28, 10%) or newborns (n = 2, 1%). In addition, 25 studies (8%) examined mother–child pairs to examine prenatal exposures and a health effect in the child, predict levels of a chemical in the child based on mothers’ exposures, or identify biomarkers of prenatal exposures. The number of chemicals examined per study ranged from 1 to 192. The majority of studies exclusively examined metals/metalloids (n = 140, 49%).

The 20 most frequently applied ML methods are listed in Table 3. Random forest was the most common (n = 142, 50%), followed by XGBoost (n = 99, 35%) and support vector machine (SVM) (n = 95, 33%). Three methods used for mixture analysis—Bayesian kernel machine regression (BKMR), weighted quantile sum regression (WQSR), and quantile-based G-computation (QGC)—were also a part of the top 20 ML methods.

Techniques to explain the outputs of models (i.e., explainable AI) were also applied frequently. The Shapley Additive Explanations (SHAP) approach was the most popular and used in 83 studies to show the contribution of features to model outputs. Less common interpretability methods were partial dependence plots, permutation feature importance, local interpretable model-agnostic explanations, and accumulated local effects.

**Figure 4:**
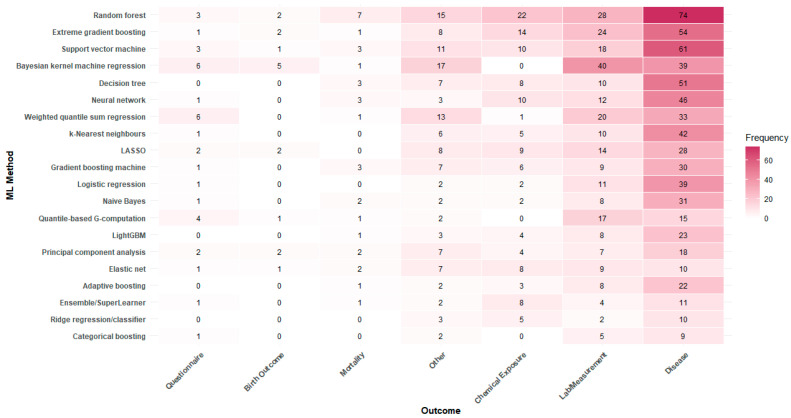
Heat map of top 20 ML methods and outcome categories (based on number of studies).

The majority of studies used human biomonitoring data to predict a disease or health-based lab/measurement outcome using supervised methods such as random forest, XGBoost, SVM, BKMR, decision tree, and neural network (Figure 4). There were also studies using these and other ML methods for predicting chemical exposures. ML methods were less frequently applied to human biomonitoring data for assessing mortality, birth outcomes, and cognitive or learning-based questionnaire outcomes.

### 3.2. Categorization of ML Methods

**Figure 5:**
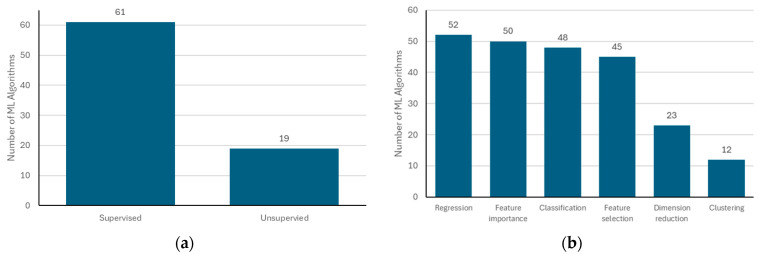
ML methods categorized by learning paradigm and type of task: (**a**) number of methods by learning paradigm and (**b**) number of methods by type of task.

We identified a total of 82 ML methods that were applied to human biomonitoring data. Certain methods were classified under broader classes of methods rather than counting them individually (e.g., a multi-layer perceptron was placed under a neural network and a gradient boosting decision tree under a gradient boosting machine). The classification of methods by learning paradigm and type of task is shown in Figure 5. None of these categories were mutually exclusive, and a method could fall under multiple categories. Supervised methods were used most frequently (i.e., methods that used labeled data). Regression (i.e., prediction of a continuous outcome) was the most common type of task, and this was closely followed by feature importance, classification, and feature selection. Fewer of the ML methods were used to perform dimensionality reduction and clustering. The Supplementary Materials, Table S1, indicates each method’s classification by learning paradigm, type of task, and specification by structure.

### 3.3. Mapping of Studies by Biomonitoring Themes

**Figure 6:**
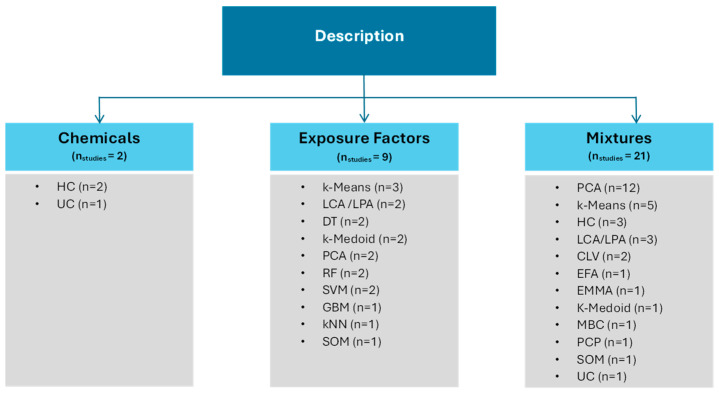
ML methods that addressed description-related biomonitoring questions. Abbreviations: CLV = clustering of variables around latent variables; DT = decision tree; EFA = exploratory factor analysis; EMMA = end-member modeling analysis; GBM = gradient boosting machine; HC = hierarchical clustering; kNN = k-nearest neighbors; LCA = latent class analysis; LPA = latent profile analysis; MBC = model-based clustering; PCA = principal component analysis; PCP = principal component pursuit; RF = random forest; SOM = self-organizing maps; SVM = support vector machine; UC = unsupervised clustering.

**Figure 7:**
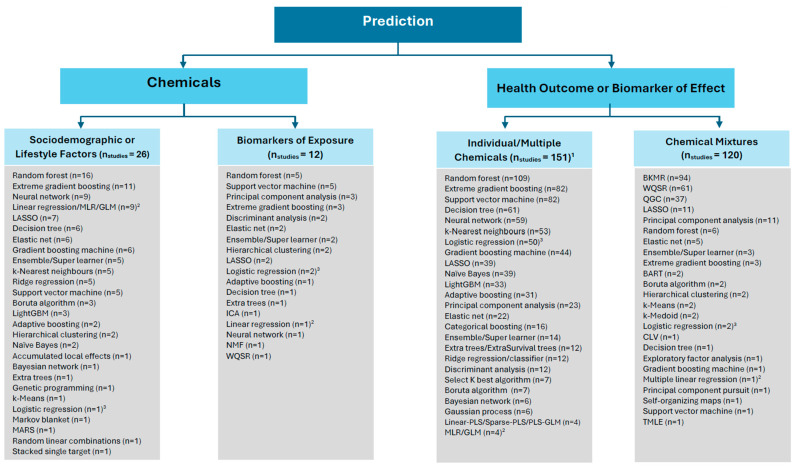
ML methods that addressed association/prediction-related biomonitoring questions. Abbreviations: BART = Bayesian additive regression tree; BKMR = Bayesian kernel machine regression; CLV = clustering of variables around latent variables; GLM = generalized linear model; ICA = independent component analysis; LASSO = least absolute shrinkage and selection operator; LightGBM = light gradient boosting machine; MARS = multivariate adaptive regression splines; MLR = multiple linear regression; NMF = non-negative matrix factorization; PLS = partial least squares; QGC = quantile based G-computation; TMLE = targeted maximum likelihood/minimum loss estimation; WQSR = weighted quantile sum regression. ^1^ The top 20 ML methods are provided. ^2^ Linear regression/MLR/GLM was not counted if it was part of a regularization method, such as LASSO and ENET (in these cases, the study was counted under the respective regularization method) or if applied to components of a clustering method (in these cases, the study was counted under the respective clustering method). Studies that only applied linear regression/MLR/GLM without another ML method were excluded. ^3^ Logistic regression was not counted if it was part of a regularization method, such as LASSO and ENET (in these cases, the study was counted under the respective regularization method) or if applied to components of a clustering method (in these cases, the study was counted under the respective clustering method). Studies that only applied logistic regression without another ML method were excluded.

The mapping of studies according to Themes 1–3 (description of chemicals, exposure factors, and mixtures) and Themes 4–5 (association/prediction of chemicals and health-related outcomes) is shown in Figure 6 and Figure 7, respectively. The Supplementary Materials, Tables S2–S8, provide details of the studies classified under each theme.

#### 3.3.1. Description of Chemicals, Exposure Factors, and Mixtures

Unsupervised ML methods were primarily applied to describe chemicals, exposure factors, and mixtures. Under Theme 1 (chemicals), two studies applied an ML technique to examine chemical concentrations and trends (Table S2). Hierarchical clustering, combined with linear mixed-effects modeling, was used to identify chemical clusters and explore temporal patterns of 192 endocrine-disrupting chemicals in serum and urine in a cohort of 50 pregnant women [34]. Hierarchical clustering was also used to explore trends of 151 metabolites and 179 parent compounds in the urine of NHANES participants from 1999 to 2016 [35].

Under Theme 2 (exposure factors), nine studies examined the variation in chemical concentrations by potential exposure factors, such as sociodemographic or lifestyle-related variables (Table S3). Most of these studies used an unsupervised method (e.g., k-means and k-medoid clustering) to form groups of participants with similar chemical exposure profiles, and then applied descriptive statistics to show the differences in characteristics between the participant groups [36,37,38]. In addition, a few supervised ML methods (e.g., decision tree and random forest) were applied to separate occupation types based on chemical exposures [39,40].

The studies listed under Theme 3 (mixtures) provided some information about the composition of chemical mixtures (Table S4). Principal component analysis (PCA) was commonly applied in these studies to reduce complex mixtures to a smaller number of components or for the identification of chemical clusters. Less commonly used approaches were k-means, hierarchical clustering, latent class/profile analysis, clustering of variables around latent variables, and exploratory factor analysis, among others.

#### 3.3.2. Association/Prediction of Chemicals or Health-Related Outcomes

Under Theme 4 (chemicals), 38 studies applied ML methods by using chemical exposure as the outcome. Of these, 26 studies predicted chemical exposures from sociodemographic or lifestyle-related factors (Table S5) and 12 studies from biomarkers of exposure, such as a gene expression marker (Table S6). One of the more commonly predicted exposures was blood lead levels in children, using supervised ML methods (e.g., [41,42]). Regression methods were used to predict blood lead level, while classification methods were used to predict group placement of children based on a blood lead threshold (e.g., ≥5 and <5 µg/dL or ≥10 and <10 µg/dL).

By far, the majority of studies focused on predicting a health-related outcome from chemical exposure (Theme 5). The purpose/objective was used to determine if the study was conducted in the context of chemical mixtures or not. Individual or multiple chemicals (not as chemical mixtures) predicted a health-related outcome by using ML in 151 studies (Table S7). Figure 7 lists the 20 most frequently applied methods by these studies (out of a total of 50 methods). Random forest was the most popular method, followed by XGBoost and SVM (with an equal number of studies each). While supervised ML was more common, unsupervised methods, such as PCA, were also applied in combination with supervised methods in some cases (e.g., [43,44]).

In the context of chemical mixtures, 120 studies were identified (Table S8). The most commonly applied methods to analyze mixtures and health-related outcomes were BKMR, followed by WQSR and QGC. Some studies also examined mixtures with clustering techniques, such as PCA, in addition to the application of BKMR (e.g., [45]).

### 3.4. Other ML Applications

In addition to the prediction of chemical exposures and health outcomes, ML methods were applied to address other specific areas relevant to biomonitoring and data analysis. These are described below, with examples from the literature.

#### 3.4.1. Environmental Risk Scores/Exposure Indices

Chemical exposure from the environment rarely occurs in isolation, and a major challenge encountered in biomonitoring is examining co-occurring, correlated exposures. The mixture methods mentioned in Section 3.3.2, and described in more detail below in Section 3.5.2, aim to address this problem by modeling the chemical mixture as a whole. Another approach is the construction of an environmental risk score (ERS) or an exposure index by using ML (e.g., [46,47]). This approach involves the use of supervised ML to first estimate a quantitative score for multiple chemicals from their relative effects on an outcome, followed by modeling the score or index on outcomes of interest. An illustrative example of the ERS is provided by Park et al. [46]. In this study, an ERS was constructed for 20 metal biomarkers based on their relative effects on an oxidative stress marker, gamma-glutamyl transferase, using four ML methods (adaptive elastic net, Bayesian additive regression tree, BKMR, and super learner). The adaptive elastic net model performed optimally, and its ERS was associated with cardiovascular endpoints, such as blood pressure and hypertension, using traditional statistical methods. The ERS provides a single quantitative representation of a complex mixture, which is intuitively appealing and easy to incorporate into subsequent modeling, although it will be heavily dependent on the methodological choices that go into its construction.

#### 3.4.2. Multi-Media Biomarkers

Another commonly encountered situation in biomonitoring is the presence of multiple biomarkers for a single chemical. For example, metals can be measured in blood, urine, and hair, or a chemical may be measured in its parent form and metabolites. Traditional statistical methods generally allow for biomarkers of a chemical to be evaluated one at a time, although this may not accurately represent the total body burden. An interesting application of ML was to develop a biomarker for lead that incorporated measurements in blood, urine, hair, and nails to capture total exposures in adolescents [48]. Two unsupervised methods (independent component analysis and non-negative matrix factorization) and one supervised method (WQSR) were used to create a multi-media biomarker from these individual biomarkers. The WQSR approach was based on the relative effects of each biomarker on an outcome (similar to the construction of an ERS described in Section 3.4.1), whereas the unsupervised methods were based on factor loadings and were independent of an outcome. Each of the three ML methods produced a set of weights that represented the relative contribution of the individual biomarkers, and these weights were used to construct three separate multi-media biomarkers (one from each ML method). The multi-media biomarkers were then associated with measures of intelligence quotient using linear regressions and were found to produce a more complete assessment compared with a single blood lead biomarker [48].

#### 3.4.3. Imputation of Missing Data

Missing data can result in lower sample sizes and loss of valuable information. It can have significant impacts on analytical outputs depending on the amount of missing data, how it is handled (e.g., removal, imputation, and imputation method), and whether or not the data are missing at random. There are statistical methods for imputing missing data, such as replacements (e.g., with the mean or median), last-observation-carried-forward imputation, regression imputation, and multiple imputation. More advanced ML methods have also been used to address missing data that can account for the underlying patterns in the dataset, which may impute missing data with values that are closer to the true values [49]. In the biomonitoring literature, we found examples of missing data being imputed with k-nearest neighbors [50] and gradient boosting machine [51] for continuous variables, decision tree [50], and super learner (random forest and neural network as the base learners) [51] for categorical variables, and light gradient boosting machine [52] and random forest [53] for both continuous and categorical variables. Random forest was more commonly used to impute missing data, using the missForest package in R. This is a promising ML method for imputing missing data, as it can handle multiple types of variables and is robust to outliers. The choice of whether to use the more advanced ML methods for data imputation will depend on multiple factors, including the complexity of the dataset, the mechanism of missing data, and available computing resources. Datasets with small amounts of missing at random data may be easily handled with simpler methods, like single-value replacements. Alternatively, datasets with variables that are not missing at random and/or with complex relationships between variables may be better suited to ML methods for data imputations.

#### 3.4.4. Causal Pathways

The traditional statistical inference and ML methods in the biomonitoring studies were generally applied to elucidate associations or predictive performance of variables rather than causality. A few studies attempted to apply causal ML methods to address underlying mechanisms and causal relationships between variables (e.g., [54,55]). These ML methods involve the construction of graphical models, directed acyclic graphs (DAGs), and networks to depict and quantify connections between variables. Bowles et al. (2024) explored modifiable and non-modifiable risk factors, including heavy metals, on cardiovascular disease risk scores using a mixed graphical model and a Bayesian network [54]. The mixed graphical model contains nodes to represent the variables and edges between nodes to represent the conditional independent relationships between variables, with the thickness of the edge being proportional to the strength of the relationship. A Bayesian network was used to output DAGs to understand the directional relationship between variables [54]. These methods allowed for the proximal causal pathways (i.e., direct and immediate cause–effect relationships) and distal causal pathways (i.e., indirect cause–effect relationships and mediators) to be identified. The study found that race/ethnicity was the most influential on cardiovascular scores and that it acted via blood mercury and cadmium [54]. Causality is a complicated phenomenon that cannot be based on a single study or analytical approach. While causal ML methods may be used in support of causality, conclusions will require that many other factors be taken into consideration, such as study design, plausibility, and multiple lines of evidence.

### 3.5. ML Recommendations

Table 4 and Table 5 provide details of the ML methods that were most frequently identified in the literature to address the biomonitoring themes in Table 1. It is difficult to recommend any particular method, as the choice of method will be influenced by many factors, such as the specific research context (e.g., exploratory vs. hypothesis testing), the type of data available (e.g., dataset size, variable types, missingness), the degree of interpretability required, and resource availability (e.g., computing power). In many cases, the performance of multiple methods may need to be compared to determine which method is optimal. Furthermore, it is important that all ML outputs are interpreted in the context of theory, domain knowledge, and findings from other studies to ensure alignment with the conceptual understanding of the topic area. For more information about additional ML methods, implementation, and interpretation of outputs, readers are encouraged to consult textbooks on ML (e.g., [56]).

#### 3.5.1. Unsupervised ML Methods

Unsupervised methods, such as clustering techniques and PCA, can be used for data exploration and to address description-related questions (Table 4). They can also be combined with supervised ML or other traditional statistical inference methods to address analytical questions, such as comparing the chemical exposure levels of clusters or using principal components as variables in a regression model. The choice of unsupervised method will depend on the analysis goals (e.g., identification of similar observations, which would require a clustering method, or dimensionality reduction, which would be better suited to PCA), and if the clusters are known or unknown a priori. Unsupervised ML methods can be especially useful for identifying patterns in datasets containing a large number of correlated chemical biomarkers. For example, the study by Buck Louis et al. [34] illustrated the use of hierarchical clustering, with cross-correlation as the distance metric, to identify patterns in 192 chemicals across pregnancy trimesters. Four clusters of chemicals were identified based on time course during pregnancy, with the first chemical cluster increasing across trimesters, the second cluster having a mid-pregnancy peak, the third cluster decreasing across trimesters, and the fourth cluster having a mid-pregnancy trough.

#### 3.5.2. Supervised ML Methods

There are many types of supervised ML methods available to address analytical questions in biomonitoring, and each method will have its advantages, limitations, and context-specific considerations for implementation. As described in Section 3.3.2, supervised methods can be used for the prediction of chemicals or health outcomes in classification and regression-based tasks and are able to incorporate many more variables simultaneously than traditional statistical methods.

Table 5 provides the main highlights for the most frequently applied supervised ML methods, random forest, XGBoost, SVM, and neural networks, as well as the mixture methods, BKMR, WQSR, and QGC. The tree-based ensemble methods (i.e., random forest and XGBoost) are well-suited for prediction and providing interpretable outputs through variable importance scores; these methods are among the strongest candidates for biomonitoring purposes. Support vector machine may be an option if datasets contain a smaller number of samples than variables, although it is less interpretable than the tree-based methods. Neural networks offer the greatest predictive flexibility for high-dimensional, multi-modal data, but they require substantially larger samples, computing resources, and have low interpretability. Of the chemical mixture methods, BKMR is a strong candidate as it can accommodate multicollinearity, non-linearity, and chemical interactions, and examine the overall mixture as well as individual chemicals. The main limitations of BKMR are its computing requirements and the absence of quantitative effect estimates, which can make interpretation more difficult. When deciding on what ML methods to apply, the advantages and limitations of each will need to be carefully assessed and balanced for the specific research questions, objectives, and datasets at hand.

### 3.6. International Scan

The survey questions and results are shown in the Supplementary Materials. Here, we provide a summary of the key results.

#### 3.6.1. Respondent Profile

**Figure 8:**
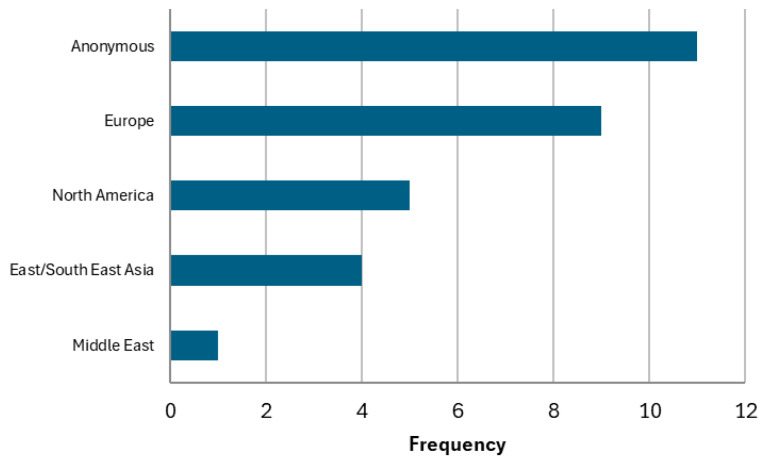
Region of origin of biomonitoring programs of survey respondents.

The survey received 30 responses (18.6% response rate), and the respondents were from 15 countries in Europe, North America, East/Southeast Asia, and the Middle East (Figure 8, countries aggregated into regions to maintain confidentiality). Eleven respondents submitted responses anonymously. Respondents were part of national-level biomonitoring programs (n = 17); other (n = 9), which included a local- and regional-level program, a workplace biomonitoring program, an occupational program, and a targeted biomonitoring study; and provincial/state-level programs (n = 3). Most (n = 23) had over five years of experience in human biomonitoring. However, the majority (n = 18) had beginner-level experience with AI/ML. Of the 30 respondents, eight indicated that they have implemented AI/ML in biomonitoring data collection and analysis.

#### 3.6.2. Future Plans and Perspectives on Potential and Barriers of AI/ML

Many of the respondents (n = 13) planned to implement or expand the use of AI/ML methodologies in their programs over the next one to two years. While most (n = 22) thought that AI/ML has potential to improve the future of biomonitoring, when asked about specific methodologies that hold the most potential, the majority of respondents (n = 21) were unsure. Five respondents provided a response, indicating the following methodologies: hierarchical clustering, tree-based methods, random forests, k-nearest neighbors, and mixture analyses.

Seven respondents felt that certain external factors could increase the urgency to adopt AI/ML, such as funding priorities, the need to deliver results more quickly and efficiently, and developments in international harmonization.

**Figure 9:**
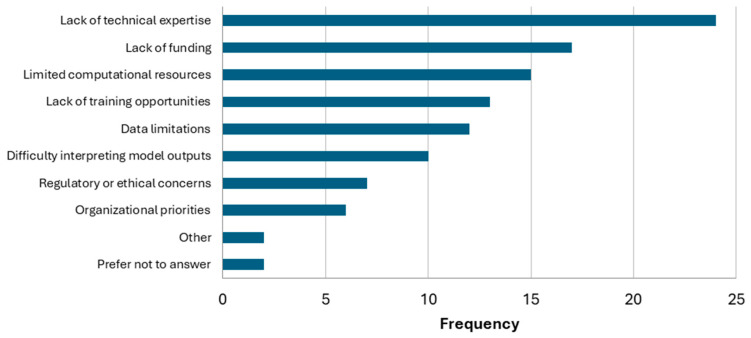
Primary barriers to the implementation of AI/ML methods.

The primary barriers to the implementation of AI/ML are shown in Figure 9. Lack of technical expertise was the most commonly selected (n = 24), followed by lack of funding (n = 17) and limited computational resources (n = 15). Lack of training opportunities (n = 13), data limitations (n = 12), difficulty with interpreting model outputs (n = 10), regulatory or ethical concerns (n = 7), and organizational priorities (n = 6) were selected as well. In the other category, two respondents mentioned issues related to sustainability and that AI/ML may be excessive for their particular datasets.

#### 3.6.3. Details of AI/ML Methods Implemented in Biomonitoring Programs

**Figure 10:**
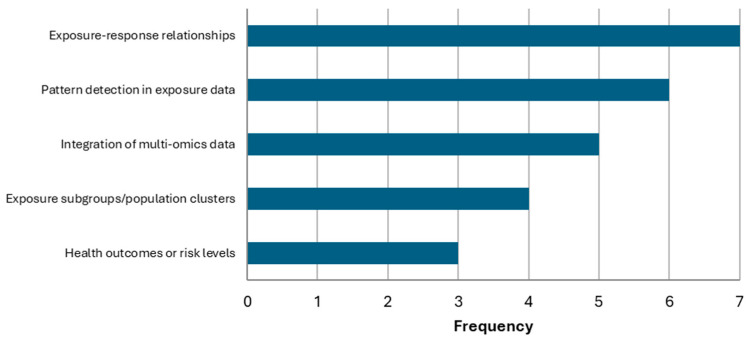
Objectives for AI/ML applications in biomonitoring programs.

Of the eight respondents who indicated that AI/ML has been implemented in their programs, Figure 10 presents the main objectives for applying these methods. The most common objective was to interpret exposure–response relationships (n = 7), followed by pattern detection in exposure data (n = 6), integration of multi-omics data (n = 5), identification of exposure subgroups/population clusters (n = 4), and prediction of health outcomes or risk levels (n = 3).

Supervised learning methods were primarily adopted, such as random forest, regression trees, least absolute shrinkage and selection operator (LASSO), SVM, BKMR, and QGC. Unsupervised methods, such as k-means and PCA, were mentioned less commonly for the purposes of exploratory data analysis, identification of latent groups, and visualization of complex datasets. All eight respondents utilized quantitative data in AI/ML applications, and two respondents indicated the use of images and text each. Six respondents indicated that they integrate multiple data types in analyses (e.g., epigenetics + metabolomics + microbiome and chemical exposures + -omics + health/clinical outcomes).

To address ethical concerns when implementing AI/ML, such as data privacy and the protection of sensitive data, respondents mentioned the use of de-identified or aggregated data and organizational-supported secure platforms, and following institutional policies for AI use.

All eight respondents were unsure if there were any measurable improvements due to AI/ML implementation. However, respondents indicated that AI/ML did have benefits in the following specific program areas: data analysis (n = 7), data collection (n = 2), data management (n = 2), and knowledge dissemination (n = 2).

## 4. Discussion

In this work, we identified several applications of ML methods to human biomonitoring datasets. While some of these methods, such as clustering techniques and random forest, have been around for decades, other newer methods, such as neural networks, have also been applied. The identified ML approaches were primarily supervised methods for predictive purposes. They can be applied to predict levels of chemicals from sociodemographic/lifestyle factors or biomarkers of exposure, or to predict a health-based outcome or biomarker of effect from chemical levels. It is important to note that the identified ML methods were generally used for prediction, not to infer causal relationships, and, therefore, while a chemical may be predictive of an outcome, it may not be causally related. Causation requires consideration of other factors, such as study design, temporality, and plausibility, or the use of other methods, such as graphical models [54,55], which are better suited for inferring causality.

ML holds significant promise for the analysis of human biomonitoring data of chemical exposures. Compared with traditional statistical techniques, ML provides flexibility by accommodating a larger number of variables and improving performance (i.e., learning) as additional data become available. Furthermore, the same dataset can be input into different algorithms to assess performance across methods, allowing for the selection of outputs with optimal performance metrics. In real-world scenarios, relationships between variables may not follow simple linear patterns, as often modeled by linear regressions. ML algorithms can model complex non-linear relationships without needing to specify a functional form in advance. ML models can also serve as a stepping stone for more sophisticated and tailored statistical analyses. For example, decision trees can provide information on feature importance, and analysts can use this information to select variables for further analysis. Lastly, when analytical solutions are not available, such as may be encountered in high-dimensional scenarios, ML models can use gradient-based optimization to find a well-fitted approximation to the data.

We did not identify foundational models (i.e., general-purpose AI models that can be used for multiple tasks) in human biomonitoring. However, based on applications in other fields, foundational models are likely to be useful for prediction-related tasks. For example, tabular foundational models could be applied to make predictions on smaller datasets and be extended to generate synthetic data [57].

One potential limitation of ML is the incorporation of survey weights to account for complex sampling designs. Biomonitoring surveys, in particular those from national-level programs, typically have survey weights that are designed to make the survey representative of the underlying population. Most studies, however, did not incorporate weights to account for complex survey designs in ML analyses. Although a few studies indicated that sampling weights were taken into consideration, it was unclear how the weights were incorporated or if the results did in fact represent weighted analyses (e.g., [58,59]). A couple of studies more clearly explained how they incorporated weights. One study applied NHANES weights to k-medoid cluster analysis and another to targeted maximum likelihood/minimum loss estimation [36,60]. These examples illustrate that while it is possible to adapt certain ML methods to accommodate complex designs, it is not yet standard practice, and guidance remains limited. The procedures to incorporate complex survey design weights into ML methods to represent an underlying population appear to be an area of ongoing investigation. There are approaches, such as the synthetic minority over-sampling technique (SMOTE), that could be used to simulate weights. Unlike traditional statistical methods, software for ML has limited options for inputting parameters to define complex survey designs, and it is unclear to what extent ignoring the survey design affects the generalizability of the results [61].

In our experience, ML methods have not been widely incorporated into chemical risk assessments for regulatory and policy-related decisions. This could be due to limited expertise in implementing and interpreting the outputs of these methods and unclear criteria for how to validate their accuracy against established statistical methods. Also, transparency is paramount for regulatory and policy contexts, and this can become an issue when method interpretability is difficult or unclear. This can be addressed by using models that provide explicit variable importance scores (e.g., tree-based methods, such as random forest) or post hoc explainability methods, such as SHAP. As ML expands in use and familiarity, there may be a greater uptake for decision-making, particularly for specific scenarios, such as the assessment of chemical mixtures.

While we attempted to identify all studies through the published peer-reviewed literature and gray sources, we may have missed some relevant evidence, as we only included English language studies and studies were screened by a single reviewer. We also did not evaluate the quality of the studies. The readiness of data availability appears to be related to the uptake of ML, with open-source data sources, such as the NHANES, being used much more frequently than sources with restricted data, such as the CHMS. While open data increases access and transparency in research, there is also concern that it is prone to excessive data mining and the generation of publications through “paper mills” [62]. Future efforts should be devoted to focusing on the validity of the ML methods using established criteria [63,64]. Additionally, as most studies came from the NHANES, the results of this review are heavily influenced by the use of a single dataset from one country. While specific findings of ML that use NHANES data may not be generalizable to other settings, the ML applications and recommendations presented in this review should be relevant to any setting that makes use of tabular data. The NHANES is the largest and most comprehensive human biomonitoring dataset in the world and arguably an ideal dataset on which to experiment with different ML methods. Also, the review focused on studies that applied ML to analyze human biomonitoring data and did not evaluate applications related to other areas of human biomonitoring, such as environmental sensors for chemical exposures. This was beyond the scope of the current work.

The international scan survey provided an early, cross-program overview of AI/ML awareness, implementation, and readiness within human biomonitoring. In concordance with the results of the literature review, respondents primarily mentioned the use of supervised learning methods to analyze chemical exposure and health relationships. Few respondents, however, had actually implemented AI/ML into their programs. Respondents were also unsure if these approaches had led to observed and measurable improvements in outcomes, such as accuracy and efficiency. This suggests that evaluation frameworks to assess the impacts of AI tools are not readily available. With the implementation of new methods, evaluation will be critical to ensure that the desired outcomes have been achieved and, furthermore, to ensure that there are no unintended consequences or biases. Overall, the findings of the survey suggest that while there is broad recognition of AI/ML’s potential, its use is currently in the early stages, and uncertainties about its implementation remain.

Several types of barriers may be preventing more wide-scale adoption. A recurring theme relating to the need for more technical expertise was apparent in the survey responses. AI is a highly specialized field requiring advanced knowledge and skills in computing and mathematics. This expertise can be difficult to acquire and to recruit on project teams. Ideally, teams should consist of specialists in AI, who are able to implement methods, as well as experts in chemical biomonitoring with the content knowledge to guide decisions and interpret outputs. Efforts aimed at providing educational resources and establishing collaborations with technical experts should encourage experimentation and wider adoption of AI methodologies. Other common barriers, such as lack of funding, limited computational resources, and lack of training opportunities, reflect structural limitations that will require additional institutional support and investments to address. Concerns about data limitations may also point to structural and resource issues with respect to having high-quality data available that is ML-ready.

While we received responses from several countries, the response rate of about 19% was low and will not necessarily be representative of all human biomonitoring initiatives. Also, we recruited respondents from professional networks, colleague referrals, and public program listings. This approach can introduce selection bias, as it will have likely resulted in a selective sample that is not representative of all researchers in the field. Most respondents were from national-level programs and/or had more than five years of experience. It is possible that our outreach did not identify smaller and less established programs, or these programs may have been less likely to respond to the survey. Future surveying efforts could target these programs, as well as specific roles, such as data analysts and researchers who work more closely with datasets. We were also limited in our ability to discern geographic and institutional diversity, as respondents could submit responses anonymously, and some submissions may have come from the same biomonitoring program.

## 5. Conclusions

Through a comprehensive literature review and survey of biomonitoring programs, this work provides insights into the extent of adoption of ML methodologies in human biomonitoring. ML has been applied to address complex questions in human biomonitoring related to exposure–response relationships, factor–exposure relationships, and chemical mixtures. It has also demonstrated uses in combining multiple chemical exposures into quantitative risk scores, integrating multiple biomarkers for a chemical to reflect total body burden, and imputing missing data. Several of these methods are well established and can be used alongside conventional statistical analyses. Also, the methods are constantly evolving and, in time, foundational models (i.e., deep learning neural networks) that can adapt and perform multiple tasks could be integrated into sophisticated processes. For the application of more advanced technologies and development of automated workflows, ongoing investment in developing an AI infrastructure, including fostering partnerships with AI experts, internal capacity-building, and procurement of equipment and secure platforms, will be required.

## Figures and Tables

**Table 1:** Themes and questions relevant to human biomonitoring.

Category	Theme	Examples of Specific Questions of Interest
Description	Chemicals	What chemicals are people exposed to and at what levels? Are there trends in chemical concentrations over time?
	2. Exposure Factors	How do chemical concentrations vary by demographic and lifestyle factors? What are the characteristics of subgroups that are highly exposed to chemicals? What are the characteristics of subgroups that are close to the limits of detection (i.e., less exposed to chemicals)?
	3. Mixtures	Can chemical clusters be identified (i.e., chemicals that co-exist) in the population? What are the most prevalent chemicals or chemical combinations in mixtures?
Association or Prediction	4. Chemicals	Can sociodemographic and lifestyle factors predict what chemicals are likely to be present in an individual? Can biomarkers of exposure predict chemical exposures?
	5. Health-related outcome (including biomarkers of effects)	Is there an association between a chemical and a health-related outcome? OR Can exposure to a chemical predict a health-related outcome? Is there an association between chemical mixtures and a health-related outcome? OR Can chemical mixtures predict a health-related outcome?

**Table 2:** National-level human biomonitoring surveys represented in review.

Survey	Country	n_studies_
National Health and Nutrition Examination Survey (NHANES)	United States	136
Japan Environment and Children’s Study (JECS)	Japan	4
Korean National Environmental Health Study (KoNEHS)	South Korea	4
Korea National Health and Nutrition Examination Survey (KNHANES)	South Korea	3
Korean Children’s Environmental Health Study (Ko-CHENS)	South Korea	1
National Human Biological Monitoring Project of China (NHBMP)	China	1
German Environmental Survey (GerES)	Germany	1
Canadian Health Measures Survey (CHMS)	Canada	1

**Table 3:** The top 20 most frequently applied machine learning methods.

Method	n_studies_ (%)
1. Random Forest	142 (50)
2. Extreme Gradient Boosting (XGBoost)	99 (35)
3. Support Vector Machine (SVM)	95 (33)
4. Bayesian Kernel Machine Regression (BKMR)	94 (33)
5. Decision Tree	72 (25)
6. Neural Network	69 (24)
7. Weighted Quantile Sum Regression (WQSR)	63 (22)
8. k-Nearest Neighbors (kNN)	61 (21)
9. Least Absolute Shrinkage and Selection Operator (LASSO)	58 (20)
10. Gradient Boosting Machine (GBM)	53 (18)
11. Logistic Regression	51 (18)
12. Naïve Bayes	41 (14)
13. Quantile-Based G-Computation (QGC)	38 (13)
14. Light Gradient Boosting Machine (LightGBM)	36 (13)
15. Principal Component Analysis (PCA)	36 (13)
16. Elastic Net (ENET)	35 (12)
17. Adaptive Boosting (AdaBoost)	34 (12)
18. Ensemble/Super Learner	25 (9)
19. Ridge Regression/Classifier	19 (7)
20. Categorical Boosting	16 (6)

**Table 4:** Unsupervised ML method details.

Applications	ML Method	Theory	Pros	Cons
To address description-related questions (Themes 1–3 in Table 1); Exploratory data analysis; Can be combined with traditional statistical inference methods or supervised ML methods (e.g., as a pre-processing step) to address analytical questions (Themes 4–5 in Table 1).	Hierarchical clustering	An iterative clustering method based on a measure of dissimilarity between observations and groups of observations. The number of clusters is based on a selected height in the dendrogram.	No need to pre-specify the number of clusters; Provides a tree-based visualization of observation clustering (dendrogram); Can be the optimal clustering method if the number of clusters is unknown.	Careful consideration required when choosing the dissimilarity measure (e.g., Euclidean distance, correlation-based distance) and the method to estimate dissimilarity between groups of observations, as these will have a large influence on the dendrogram Careful consideration required when choosing cut-off height of dendrogram, as that will determine the number of clusters Variable scaling may be required Sensitive to outliers
	K-means	The dataset is split into K clusters that are distinct and non-overlapping. Each observation belongs to only one cluster. The algorithm minimizes the within-cluster variation (usually determined by the squared Euclidean distance).	Computationally efficient; Can be the optimal clustering method if the dataset is known to contain a specific number of clusters.	Need to pre-specify number of clusters (K) Results depend on the initial cluster assignment, which is performed randomly (should be run multiple times using different initial assignments) Difficulty with non-spherical clusters Variable scaling may be required Sensitive to outliers
	Principal component analysis	Transforms the dataset into orthogonal principal components that reduce the number of dimensions while explaining the maximum variance in the data.	Reduces the number of dimensions in the dataset; Reduces noise and multicollinearity; The principal components score vectors can be input as variables in regression modeling.	Principal components are linear combinations of original variables and non-linear relationships not captured Optimal number of principal components based on visual inspection of scree plot and is subjective Loss of information Can be difficult to interpret meaning of principal components Variable scaling may be required

**Table 5:** Supervised ML method details.

Application	ML Method	Theory	Pros	Cons
To address analytical questions related to the prediction of chemicals and health outcomes (Themes 4a–b and 5a Table 1).	Random forest	Tree-based ensemble that aggregates simple decision trees.	Classification and regression tasks; Handles missing values; Robust to noise and outliers; Interpretable: provides explicit variable information; Variable scaling not required.	Long running times; Memory intensive; Sensitive to imbalanced outcome groups; Hyperparameter tuning can be time-consuming.
	Extreme gradient boosting	A gradient boosting method that builds decision trees sequentially, with each tree correcting the error or residual made by the previous trees.	Classification and regression tasks; Handles missing values; Prevents overfitting by regularization; Faster training due to efficient computation; Interpretable: provides variable importance information; Variable scaling not required.	Computationally efficient, but intensive; Sensitive to noisy data; Hyperparameter tuning is time-consuming.
	Support vector machine	Finds the optimal hyperplane that maximally separates or fits the data.	Classification and regression tasks; Performs well in high-dimensional spaces (flexible kernel functions); Effective when number of variables > number of samples; Robust to overfitting in low-noise scenarios.	Computationally expensive, especially with large datasets; Interpretability: no built-in variable importance; Sensitive to the choice of kernel and hyperparameters; No handling of missing values; Variable scaling may be required.
	Neural networks	One or more layers of interconnected nodes called “neurons”. Each neuron receives information from all neurons in previous layer, and then weight and bias terms are added. Gradient descent and back-propagation adjust these terms to minimize the model’s loss function.	Classification and regression tasks; Can handle extremely complex relationships; Automatic variable extraction and engineering; Can utilize GPU’s computational power for faster training.	Training can take a very long time, depending on the data size and model’s architecture; Due to long training, hyperparameter tuning can be extremely time-consuming; May not perform well on small datasets; Low interpretability; Variable scaling may be required.
To address analytical questions related to chemical mixtures; (Theme 5b in Table 1).	Bayesian kernel machine regression	Models the combined effect of multiple chemicals in a mixture, while allowing for non-linear effects and potential interactions. Implements the MCMC algorithm and uses a kernel function to estimate the exposure–response surface in a flexible, non-parametric approach.	Handles multicollinearity, non-linearity, and interactions among individual chemicals; Examines overall mixture effects, individual chemical contributions to the mixture, and pairwise interactive effects; Identifies important mixture components through variable selection; Provides comprehensive visualizations.	Time-consuming and memory intensive; Not easily interpretable quantitatively; Complete case analysis only; Among highly correlated exposures, BKMR may indicate that exposure A is more important, but the potential adverse effects of other exposures cannot be disregarded.
	Weighted quantile sum regression	Transforms chemical exposures into quantiles and constructs a weighted exposure index. The index represents the mixture, and the weights represent the relative contributions of individual chemicals to the index. The index is modeled as a predictor in a GLM.	Handles multicollinearity; Examines overall mixture effects (joint effects of components acting in the same direction); Provides exposure index weights for the relative contribution of individual chemicals to an outcome; Faster computing time compared to Bayesian kernel machine regression.	Assumes additivity (less flexible); Constrains all exposures to act in the same direction on the outcome (i.e., assumes directional homogeneity), though extensions can model positive and negative direction effects; Cannot directly account for interactions.
	Quantile-based G-computation	Transforms all chemical exposures into quantiles and models a GLM combined with G-computation to estimate the joint effect of simultaneous increases in exposures. Also constructs weights that represent the relative contribution of individual chemicals.	Examines overall mixture effects; Relaxes the assumption of directional homogeneity (i.e., components can act in different directions); More flexible than weighted quantile sum regression in allowing for non-linearity and non-additivity (interactions) of the effects of individual chemicals and the mixture as a whole; Faster computing time compared to Bayesian kernel machine regression.	More difficult to interpret the individual contribution of chemicals to the overall mixture effect because, unlike weighted quantile sum regression, it does not rely on constrained weights to estimate the mixture effect; Unlike Bayesian kernel machine regression, which can flexibly capture non-linearity and interactions through its kernel structure, quantile-based G-computation requires nonlinearity or interaction effects to be pre-specified in the model.

Abbreviations: GLM = generalized linear model; GPU = graphics processing unit; MCMC = Markov Chain Monte Carlo.

## Data Availability

The data extracted for the literature review are available upon request. Individual responses to the survey cannot be released to comply with privacy obligations.

## Supplementary Materials

The following supporting information can be downloaded at https://www.mdpi.com/article/10.3390/ijerph23050680/s1, The Literature Search Strategies; International Scan Survey; International Scan Results (question by question); Table S1: Categorization of ML Methods; Table S2: Description—Chemicals; Table S3: Description—Exposure Factors; Table S4: Description—Mixtures; Table S5: Association/Prediction—Chemicals (from exposure factors); Table S6: Association/Prediction—Chemicals (from biomarkers of exposure); Table S7: Association/Prediction—Health-Based Outcomes (from individual or multiple chemicals); Table S8: Association/Prediction—Health-Based Outcomes (from chemical mixtures). References [34,35,36,37,38,39,40,41,42,43,44,45,46,47,48,50,51,52,53,54,55,58,59,60,65,66,67,68,69,70,71,72,73,74,75,76,77,78,79,80,81,82,83,84,85,86,87,88,89,90,91,92,93,94,95,96,97,98,99,100,101,102,103,104,105,106,107,108,109,110,111,112,113,114,115,116,117,118,119,120,121,122,123,124,125,126,127,128,129,130,131,132,133,134,135,136,137,138,139,140,141,142,143,144,145,146,147,148,149,150,151,152,153,154,155,156,157,158,159,160,161,162,163,164,165,166,167,168,169,170,171,172,173,174,175,176,177,178,179,180,181,182,183,184,185,186,187,188,189,190,191,192,193,194,195,196,197,198,199,200,201,202,203,204,205,206,207,208,209,210,211,212,213,214,215,216,217,218,219,220,221,222,223,224,225,226,227,228,229,230,231,232,233,234,235,236,237,238,239,240,241,242,243,244,245,246,247,248,249,250,251,252,253,254,255,256,257,258,259,260,261,262,263,264,265,266,267,268,269,270,271,272,273,274,275,276,277,278,279,280,281,282,283,284,285,286,287,288,289,290,291,292,293,294,295,296,297,298,299,300,301,302,303,304,305,306,307,308,309,310,311,312,313,314,315,316,317,318,319,320,321,322,323,324,325,326,327] are cited in the Supplementary Materials.

## Abbreviations

The following abbreviations are used in this manuscript:

AI	Artificial intelligence
ANOVA	Analysis of variance
BKMR	Bayesian kernel machine regression
CHMS	Canadian Health Measures Survey
ERS	Environmental risk score
LASSO	Least absolute shrinkage and selection operator
ML	Machine learning
NHANES	National Health and Nutrition Examination Survey
PCA	Principal component analysis
QGC	Quantile-based G-computation
RWE	Real-world exposure
SHAP	Shapley Additive Explanations
SMOTE	Synthetic minority over-sampling technique
SVM	Support vector machine
WQSR	Weighted quantile sum regression
XGBoost	Extreme gradient boosting

## References

[1] Lawrence R., Dodsworth E., Massou E., Sherlaw-Johnson C., Ramsay A.I.G., Walton H., O’Regan T., Gleeson F., Crellin N., Herbert K. (2025). Artificial intelligence for diagnostics in radiology practice: A rapid systematic scoping review. eClinicalMedicine.

[2] Bassey G.E., Daniel E.A., Okesina K.B., Odetayo A.F. (2025). Transformative role of artificial intelligence in drug discovery and translational medicine: Innovations, challenges, and future prospects. Drug Des. Dev. Ther..

[3] Mendes V.I.S., Mendes B.M.F., Moura R.P., Lourenço I.M., Oliveira M.F.A., Ng K.L., Pinto C.S. (2025). Harnessing artificial intelligence for enhanced public health surveillance: A narrative review. Front. Public Health.

[4] Neo E.X., Hasikin K., Mokhtar M.I., Lai K.W., Azizan M.M., Razak S.A., Hizaddin H.R. (2022). Towards integrated air pollution monitoring and health impact assessment using federated learning: A systematic review. Front. Public Health.

[5] Ning J., Pang S., Arifin Z., Zhang Y., Epa U.P.K., Qu M., Zhao J., Zhen F., Chowdhury A., Guo R. (2024). The diversity of artificial intelligence applications in marine pollution: A systematic literature review. J. Mar. Sci. Eng..

[6] Boudreault J., Lamothe F., Campagna C., Chebana F. (2025). Machine learning for modelling the health impacts of extreme heat: A comprehensive literature review. Environ. Int..

[7] Tan X., Peng Z., Cheng Y., Wang Y., Chao Q., Huang X., Yan H., Chen D. (2025). Leveraging artificial intelligence for research and action on climate change: Opportunities, challenges, and future directions. Sci. Bull..

[8] Pollock T., Karthikeyan S., Walker M., Werry K., St-Amand S. (2021). Trends in environmental chemical concentrations in the Canadian population: Biomonitoring data from the Canadian Health Measures Survey 2007–2017. Environ. Int..

[9] Canadian Health Measures Survey (CHMS). https://www.statcan.gc.ca/en/survey/household/5071.

[10] Labrague L.J., Aguilar-Rosales R., Yboa B.C., Sabio J.B., de Los Santos J.A. (2023). Student nurses’ attitudes, perceived utilization, and intention to adopt artificial intelligence (AI) technology in nursing practice: A cross-sectional study. Nurse Educ. Pract..

[11] Pecqueux M., Riediger C., Distler M., Oehme F., Bork U., Kolbinger F.R., Schöffski O., van Wijngaarden P., Weitz J., Schweipert J. (2022). The use and future perspective of artificial intelligence-A survey among German surgeons. Front. Public Health.

[12] Ardon O., Schmidt R.L. (2020). Clinical laboratory employees’ attitudes toward artificial intelligence. Lab. Med..

[13] Iyer H.S., Karasaki S., Yi L., Hswen Y., James P., VoPham T. (2025). Harnessing geospatial artificial intelligence (GeoAI) for environmental epidemiology: A narrative review. Curr. Environ. Health Rep..

[14] Oskar S., Stingone J.A. (2020). Machine learning within studies of early-life environmental exposures and child health: Review of the current literature and discussion of next steps. Curr. Environ. Health Rep..

[15] Huang L., Duan Q., Liu Y., Wu Y., Li Z., Guo Z., Liu M., Lu X., Wang P., Liu F. (2025). Artificial intelligence: A key fulcrum for addressing complex environmental health issues. Environ. Int..

[16] Campbell S.M., Kung J. Filter to Retrieve Studies Related to Artificial Intelligence from the OVID MEDLINE Database. Geoffrey & Robyn Sperber Health Sciences Library, University of Alberta 2024. https://docs.google.com/document/d/1eWyO0jv9_6FYsxyC5LUYwFe9eH_3h83-tPNZ6wmos18/edit?tab=t.0#heading=h.qi55eeyvgzy9.

[17] Forbes C., Greenwood H., Carter M., Clark J. (2024). Automation of duplicate record detection for systematic reviews: Deduplicator. Syst. Rev..

[18] Barbierato E., Gatti A. (2024). The challenges of machine learning: A critical review. Electronics.

[19] Woodman R.J., Mangoni A.A. (2023). A comprehensive review of machine learning algorithms and their application in geriatric medicine: Present and future. Aging Clin. Exp. Res..

[20] Mooney S.J., Pejaver V. (2018). Big data in public health: Terminology, machine learning, and privacy. Annu. Rev. Public Health.

[21] Wang Y., Zhao Y., Therneau T.M., Atkinson E.J., Tafti A.P., Zhang N., Amin S., Limper A.H., Khosla S., Liu H. (2020). Unsupervised machine learning for the discovery of latent disease clusters and patient subgroups using electronic health records. J. Biomed. Inform..

[22] Faouzi J., Colliot O., Colliot O. (2023). Classic Machine Learning Methods. Machine Learning for Brain Disorders.

[23] Bishop C.M. (2006). Pattern Recognition and Machine Learning.

[24] Hofmann T., Schölkopf B., Smola A.J. (2008). Kernel methods in machine learning. Ann. Stat..

[25] Linero A.R. (2017). A review of tree-based Bayesian methods. Commun. Stat. Appl. Methods.

[26] Dietterich T.G., Arbib M.A. (2002). Ensemble Learning. The Handbook of Brain Theory and Neural Networks.

[27] Ayesha S., Hanif M.K., Talib R. (2020). Overview and comparative study of dimensionality reduction techniques for high dimensional data. Inf. Fusion.

[28] Aha D.W., Kibler D., Albert M.K. (1991). Instance-based learning algorithms. Mach. Learn..

[29] Burkart N., Huber M.F. (2021). A survey on the explainability of supervised machine learning. J. Artif. Intell. Res..

[30] Swartout W.R. (1985). Rule-based expert systems: The MYCIN experiments of the Stanford Heuristic Programming Project [Book Review]. Artif. Intell..

[31] Wahba G. (1990). Spline Models for Observational Data.

[32] Gunawardena C.N., Lowe C., Carabajal K. Evaluating online learning: Models and methods. Proceedings of the Society for Information Technology & Teacher Education International Conference: Proceedings of SITE 2000.

[33] Leist A.K., Klee M., Kim J.H., Rehkopf D.H., Bordas S.P.A., Muniz-Terrera G., Wade S. (2022). Mapping of machine learning approaches for description, prediction, and causal inference in the social and health sciences. Sci. Adv..

[34] Buck Louis G.M., Yeung E., Kannan K., Maisog J., Zhang C., Grantz K.L., Sundaram R. (2019). Patterns and variability of endocrine-disrupting chemicals during pregnancy: Implications for understanding the exposome of normal pregnancy. Epidemiology.

[35] Stanfield Z., Setzer R.W., Hull V., Sayre R.R., Isaacs K.K., Wambaugh J.F. (2024). Characterizing chemical exposure trends from NHANES urinary biomonitoring data. Environ. Health Perspect..

[36] Fu Y.-P., Chen W.-Y., Guo L.-Q., Zhu Y.-Q., Yuan J.-S., Liu Y.-H. (2022). The association between hearing threshold and urinary personal care and consumer product metabolites in middle-aged and elderly people from the USA. Environ. Sci. Pollut. Res..

[37] Liu J., Wang K. (2024). Disentangling the relationship between urinary metal exposure and osteoporosis risk across a broad population: A comprehensive supervised and unsupervised analysis. Toxics.

[38] Yonkman A.M., Alampi J.D., Kaida A., Allen R.W., Chen A., Lanphear B.P., Braun J.M., Muckle G., Arbuckle T.E., McCandless L.C. (2023). Using latent profiles analysis to identify associations between gestational chemical mixtures and child neurodevelopment. Epidemiology.

[39] Wang J.-R., Kuang H.-X., Liu Y., Li X.-Y., Chen T.-H., Zhu X.-H., Fan R.-F., Xiang M.-D., Yu Y.-J. (2024). Associations between volatile organic compounds exposure and multiple oxidative damage biomarkers: Method development, human exposure, and application for e-waste pollution prediction. Sci. Total Environ..

[40] Moro A.M., Brucker N., Goethel G., Flesch I., Nascimento S., Charão M., Gauer B., Sauer E., Cestonaro L.V., Viçozzi G.P. (2025). The influence of blood titanium levels on DNA damage in Brazilian workers occupationally exposed to different chemical agents. Biol. Trace Elem. Res..

[41] Abbasi A., DiTraglia F.J., Gazze L., Pals B. (2023). Hidden hazards and screening policy: Predicting undetected lead exposure in Illinois. J. Health Econ..

[42] Liu X., Taylor M.P., Aelion C.M., Dong C. (2021). Novel application of machine learning algorithms and model-agnostic methods to identify factors influencing childhood blood lead levels. Environ. Sci. Technol..

[43] Zhang J., Gu W., Zhai S., Liu Y., Yang C., Xiao L., Chen D. (2024). Phthalate metabolites and sex steroid hormones in relation to obesity in US adults: NHANES 2013–2016. Front. Endocrinol..

[44] Wei H., Sun J., Shan W., Xiao W., Wang B., Ma X., Hu W., Wang X., Xia Y. (2022). Environmental chemical exposure dynamics and machine learning-based prediction of diabetes mellitus. Sci. Total Environ..

[45] Chiu Y.H., Bellavia A., James-Todd T., Correia K.F., Valeri L., Messerlian C., Ford J.B., Mínguez-Alarcón L., Calafat A.M., Hauser R. (2018). Evaluating effects of prenatal exposure to phthalate mixtures on birth weight: A comparison of three statistical approaches. Environ. Int..

[46] Park S.K., Zhao Z., Mukherjee B. (2017). Construction of environmental risk score beyond standard linear models using machine learning methods: Application to metal mixtures, oxidative stress and cardiovascular diseases in NHANES. Environ. Health.

[47] Yan C., Zhu Z., Guo X., Zong W., Liu G., Jin Y., Cui S., Liu F., Gao S. (2025). The impact of multipollutant exposure on hepatic steatosis: A machine learning-based investigation into multipollutant synergistic effects. Front. Public Health.

[48] Levin-Schwartz Y., Gennings C., Henn B.C., Coull B.A., Placidi D., Lucchini R., Smith D.R., Wright R.O. (2020). Multi-media biomarkers: Integrating information to improve lead exposure assessment. Environ. Res..

[49] Alabadla M., Sidi F., Ishak I., Ibrahim H., Affendey L.S., Ani Z.C., Jabar M.A., Bukar U.A., Devaraj N.K., Muda A.S. (2022). Systematic review of using machine learning in imputing missing values. IEEE Access.

[50] You Z.-M., Li Y.-S., Meng F.-S., Zhang R.-X., Xie C.-X., Liang Z., Zhou J.-Y. (2025). Interpretable machine learning approaches for predicting prostate cancer by using multiple heavy metals exposures based on the data from NHANES 2003–2018. Ecotoxicol. Environ. Saf..

[51] Puvvula J., Hwang W.-T., McCandless L., Xie C., Braun J.M., Vuong A.M., Oulhote Y., Schisterman E.F., Shinohara R.T., Booij L. (2025). Gestational exposure to environmental chemical mixtures and cognitive abilities in children: A pooled analysis of two North American birth cohorts. Environ. Int..

[52] Pala D., Xu J., Xie Y., Zhang Y., Shen L. (2024). Identifying biological markers and sociodemographic factors that influence the gap between phenotypic and chronological ages. Inform. Health Soc. Care.

[53] Zhu J., Hu S., Wang S., Zhang Y., Zhu Q., Zhang M., Shi Z. (2023). Association between metal mixture exposure and abnormal glucose metabolism in multiple mixture exposure models: Evidence from NHANES 2015–2016. Curr. Res. Toxicol..

[54] Bowles N.P., He Y., Huang Y.-H., Stecker E.C., Seixas A., Thosar S.S. (2024). Cardiovascular disease risk: It is complicated, but race and ethnicity are key, a Bayesian network analysis. Front. Public Health.

[55] Yang L., Gao C., Qin R., Wu Q., Sun H., Zhang T. (2025). Impact of e-waste pollutant exposure on renal injury and oxidative stress biomarkers: Evidence from causal machine learning. J. Hazard. Mater..

[56] James G., Witten D., Hastie T., Tibshirani R. (2021). An Introduction to Statistical Learning with Applications in R.

[57] Hollmann N., Müller S., Purucker L., Krishnakumar A., Körfer M., Hoo S.B., Schirrmeister R.T., Hutter F. (2025). Accurate predictions on small data with a tabular foundation model. Nature.

[58] Duan S., Wu Y., Zhu J., Wang X., Zhang Y., Gu C., Fang Y. (2024). Development of interpretable machine learning models associated with environmental chemicals to predict all-cause and specific-cause mortality: A longitudinal study based on NHANES. Ecotoxicol. Environ. Saf..

[59] Jeong S., Choi Y.-J. (2024). Investigating the influence of heavy metals and environmental factors on metabolic syndrome risk based on nutrient intake: Machine learning analysis of data from the eighth Korea National Health and Nutrition Examination Survey (KNHANES). Nutrients.

[60] Keil A.P., O’Brien K.M. (2024). Considerations and targeted approaches to identifying bad actors in exposure mixtures. Stat. Biosci..

[61] MacNell N., Feinstein L., Wilkerson J., Salo P.M., Molsberry S.A., Fessler M.B., Thorne P.S., Motsinger-Reif A.A., Zeldin D.C. (2023). Implementing machine learning methods with complex survey data: Lessons learned on the impacts of accounting sampling weights in gradient boosting. PLoS ONE.

[62] Suchak T., Aliu A.E., Harrison C., Zwiggelaar R., Geifman N., Spick M. (2025). Explosion of formulaic research articles, including inappropriate study designs and false discoveries, based on the NHANES US national health database. PLoS Biol..

[63] Ethical Considerations in the Use of Machine Learning for Research and Statistics. UK Statistics Authority. https://uksa.statisticsauthority.gov.uk/publication/ethical-considerations-in-the-use-of-machine-learning-for-research-and-statistics.

[64] Collins G.S., Moons K.G., Dhiman P., Riley R.D., Beam A.L., Van Calster B., Ghassemi M., Liu X., Reitsma J.B., Van Smeden M. (2024). TRIPOD+AI statement: Updated guidance for reporting clinical prediction models that use regression or machine learning methods. BMJ.

[65] Kalloo G., Wellenius G.A., McCandless L., Calafat A.M., Sjodin A., Karagas M., Chen A., Yolton K., Lanphear B.P., Braun J.M. (2018). Profiles and predictors of environmental chemical mixture exposure among pregnant women: The Health Outcomes and Measures of the Environment Study. Environ. Sci. Technol..

[66] Carroll R., White A.J., Keil A.P., Meeker J.D., McElrath T.F., Zhao S., Ferguson K.K. (2020). Latent classes for chemical mixtures analyses in epidemiology: An example using phthalate and phenol exposure biomarkers in pregnant women. J. Expo. Sci. Environ. Epidemiol..

[67] Sen P., Qadri S., Luukkonen P.K., Ragnarsdottir O., McGlinchey A., Jäntti S., Juuti A., Arola J., Schlezinger J.J., Webster T.F. (2022). Exposure to environmental contaminants is associated with altered hepatic lipid metabolism in non-alcoholic fatty liver disease. J. Hepatol..

[68] Zhou T., Abrishamcar S., Christensen G., Eick S.M., Barr D.B., Vanker A., Hoffman N., Donald K.A., Wedderburn C.J., Andra S.S. (2025). Associations between prenatal exposure to environmental phenols and child neurodevelopment at two years of age in a South African birth cohort. Environ. Res..

[69] Hendryx M., Luo J. (2018). Latent class analysis of the association between polycyclic aromatic hydrocarbon exposures and body mass index. Environ. Int..

[70] Gibson E.A., Nunez Y., Abuawad A., Zota A.R., Renzetti S., Devick K.L., Gennings C., Goldsmith J., Coull B.A., Kioumourtzoglou M.-A. (2019). An overview of methods to address distinct research questions on environmental mixtures: An application to persistent organic pollutants and leukocyte telomere length. Environ. Health.

[71] Matta K., Vigneau E., Cariou V., Mouret D., Ploteau S., Le Bizec B., Antignac J.-P., Cano-Sancho G. (2020). Associations between persistent organic pollutants and endometriosis: A multipollutant assessment using machine learning algorithms. Environ. Pollut..

[72] Tao C., Li Z., Fan Y., Li X., Qian H., Yu H., Xu Q., Lu C. (2021). Independent and combined associations of urinary heavy metals exposure and serum sex hormones among adults in NHANES 2013–2016. Environ. Pollut..

[73] Grau-Perez M., Caballero-Mateos M.J., Domingo-Relloso A., Navas-Acien A., Gomez-Ariza J.L., Garcia-Barrera T., Leon-Latre M., Soriano-Gil Z., Jarauta E., Cenarro A. (2022). Toxic metals and subclinical atherosclerosis in carotid, femoral, and coronary vascular territories: The Aragon Workers Health Study. Arterioscler. Thromb. Vasc. Biol..

[74] Babić Leko M., Mihelčić M., Jurasović J., Nikolac Perković M., Španić E., Sekovanić A., Orct T., Zubčić K., Langer Horvat L., Pleić N. (2023). Heavy metals and essential metals are associated with cerebrospinal fluid biomarkers of Alzheimer’s disease. Int. J. Mol. Sci..

[75] Yu Y., Wang Y., Dong Y., Shu S., Zhang D., Xu J., Zhang Y., Shi W., Wang S.-L. (2023). Butyl benzyl phthalate as a key component of phthalate ester in relation to cognitive impairment in NHANES elderly individuals and experimental mice. Environ. Sci. Pollut. Res. Int..

[76] Wang F., Lin Y., Xu J., Wei F., Huang S., Wen S., Zhou H., Jiang Y., Wang H., Ling W. (2024). Risk of papillary thyroid carcinoma and nodular goiter associated with exposure to semi-volatile organic compounds: A multi-pollutant assessment based on machine learning algorithms. Sci. Total Environ..

[77] Wu H., Kalia V., Manz K.E., Chillrud L., Dishon N.H., Jackson G.L., Dye C.K., Orvieto R., Aizer A., Levine H. (2024). Exposome profiling of environmental pollutants in seminal plasma and novel associations with semen parameters. Environ. Sci. Technol..

[78] Zang X., Feng L., Qin W., Wang W., Zang X. (2024). Using machine learning methods to analyze the association between urinary polycyclic aromatic hydrocarbons and chronic bowel disorders in American adults. Chemosphere.

[79] Chi H.-B., Tang J.-J., Fan X.-Y., Zhang H.-W., Tang F., Lin X.-S., Yang B.-R., Li N., Guo J., Wu L.-A.-S. (2025). Single- and combined-heavy metals/metalloids exposures are associated with infertility in US women aged 20–44: NHANES 2013–2020 analysis. Reprod. Toxicol..

[80] Lai Y., Nakayama S.F., Nishihama Y., Isobe T. (2025). The Japan Environment and Children’s Study Group. Determinants of plasma poly- and perfluoroalkyl substances during pregnancy: The Japan Environment and Children’s Study. Ecotoxicol. Environ. Saf..

[81] Jovanović G., Romanić S.H., Stojić A., Klinčić D., Sarić M.M., Letinić J.G., Popović A. (2019). Introducing of modeling techniques in the research of POPs in breast milk—A pilot study. Ecotoxicol. Environ. Saf..

[82] Potash E., Ghani R., Walsh J., Jorgensen E., Lohff C., Prachand N., Mansour R. (2020). Validation of a machine learning model to predict childhood lead poisoning. JAMA Netw. Open.

[83] Nishihama Y., Nakayama S.F., Isobe T., Jung C.-R., Iwai-Shimada M., Kobayashi Y., Michikawa T., Sekiyama M., Taniguchi Y., Yamazaki S. (2021). Urinary metabolites of organophosphate pesticides among pregnant women participating in the Japan Environment and Children’s Study (JECS). Int. J. Environ. Res. Public Health.

[84] Mulhern R., Roostaei J., Schwetschenau S., Pruthi T., Campbell C., MacDonald Gibson J. (2022). A new approach to a legacy concern: Evaluating machine-learned Bayesian networks to predict childhood lead exposure risk from community water systems. Environ. Res..

[85] Chaurasia P., McClean S.I., Mahdi A.A., Yogarajah P., Ansari J.A., Kunwar S., Ahmad M.K. (2023). Automated lead toxicity prediction using computational modelling framework. Health Inf. Sci. Syst..

[86] Sambanis A., Osiecki K., Cailas M., Quinsey L., Jacobs D.E. (2023). Using artificial intelligence to identify sources and pathways of lead exposure in children. J. Public Health Manag. Pract..

[87] Shin S., Ryoo J.-H. (2023). Environment-wide association study to identify exposure pathways of bisphenol A in Korean children and adolescents: Korean National Environmental Health Survey (KoNEHS) 2018–2020. Environ. Res..

[88] Wang X., Bakulski K.M., Mukherjee B., Hu H., Park S.K. (2023). Predicting cumulative lead (Pb) exposure using the Super Learner algorithm. Chemosphere.

[89] Aker A., Nguyen V., Ayotte P., Ricard S., Lemire M. (2024). Characterizing important dietary exposure sources of perfluoroalkyl acids in Inuit youth and adults in Nunavik using a feature selection tool. Environ. Health Perspect..

[90] Frndak S., Queirolo E.I., Mañay N., Yu G., Ahmed Z., Barg G., Colder C., Kordas K. (2024). Predicting blood lead in Uruguayan children: Individual- vs. neighborhood-level ensemble learners. PLoS Glob. Public Health.

[91] Mahfouz M., Mahfouz Y., Harmouche-Karaki M., Matta J., Younes H., Helou K., Finan R., Abi-Tayeh G., Meslimani M., Moussa G. (2024). Utilizing machine learning to classify persistent organic pollutants in the serum of pregnant women: A predictive modeling approach. Environ. Sci. Pollut. Res. Int..

[92] Suwannarin N., Nishihama Y., Isobe T., Nakayama S.F. (2024). Urinary concentrations of environmental phenol among pregnant women in the Japan Environment and Children’s Study. Environ. Int..

[93] Sy M., Conrad A., Jung C., Lindtner O., Greiner M. (2024). Analysis of human co-exposure to lead and cadmium using human biomonitoring (HBM) data in a Bayesian copula-based regression framework. Expo. Health.

[94] Tao L., Tang W., Xia Z., Wu B., Liu H., Fu J., Lu Q., Guo L., Gao C., Zhou Q. (2024). Machine learning predicts the serum PFOA and PFOS levels in pregnant women: Enhancement of fatty acid status on model performance. Environ. Int..

[95] Yang Y., Li C., Yang L., Zhu H., Xie Z., Falandysz J., Weber R., Qin L., Liu G. (2024). Linking industrial emissions and dietary exposure to human burdens of polychlorinated naphthalenes. Sci. Total Environ..

[96] Chaurasia P., Yogarajah P., Mahdi A.A., McClean S., Ahmad M.K., Jafar T., Singh S.K. (2025). Machine learning and explainable artificial intelligence to predict and interpret lead toxicity in pregnant women and unborn baby. Front. Digit. Health.

[97] Chou W.-C., Gaynor J.W., Graham E.M., Klepczynski B., Walker T., Coker E.S., Ittenbach R.F., Lin Z. (2025). A machine learning-based clustering analysis to explore bisphenol A and phthalate exposure from medical devices in infants with congenital heart defects. Environ. Health Perspect..

[98] Du Z., Chen D., Du X., Chen G., Chen T., Zheng W. (2025). Identification of the associations between co-exposure to organophosphate flame retardants and thyroid dysfunction and exposure risk factors in residents of Shanghai, China. Environ. Pollut..

[99] Jung S., Shah S., Oh J., Bang Y., Lee J.H., Kim H.-C., Jeong K.S., Park H., Lee E.-K., Hong Y.-C. (2025). Machine learning-based analysis on factors influencing blood heavy metal concentrations in the Korean CHildren’s ENvironmental health Study (Ko-CHENS). Sci. Total Environ..

[100] Okati N., Ebrahimi-Khusfi Z., Zandifar S., Taghizadeh-Mehrjardi R. (2025). Identifying the key factors of mercury exposure in residents of southwestern Iran using machine learning algorithms. Environ. Geochem. Health.

[101] Qu J., Mao W., Chen M., Jin H. (2025). Prediction of p-phenylenediamine antioxidant concentrations in human urine using machine learning models. J. Hazard. Mater..

[102] Fry R.C., Navasumrit P., Valiathan C., Svensson J.P., Hogan B.J., Luo M., Bhattacharya S., Kandjanapa K., Soontararuks S., Nookabkaew S. (2007). Activation of inflammation/NF-κB signaling in infants born to arsenic-exposed mothers. PLoS Genet..

[103] Demchenko V., Olszewski S., Voronenko M., Zaets E., Savina N., Lurie I., Lytvynenko V. Modeling and predicting the organochlorine pesticides concentration in the child’s body based on their accumulation in the mother’s body. Proceedings of the 2nd International Workshop on Modern Machine Learning Technologies and Data Science, MOMLET+DS 2020.

[104] Gerbi L., Austin C., Pedretti N.F., McRae N., Amarasiriwardena C.J., Mercado-García A., Torres-Olascoaga L.A., Tellez-Rojo M.M., Wright R.O., Arora M. (2022). Biomarkers of maternal lead exposure during pregnancy using micro-spatial child deciduous dentine measurements. Environ. Int..

[105] Jala A., Dutta R., Josyula J.V.N., Mutheneni S.R., Borkar R.M. (2023). Environmental phenol exposure associates with urine metabolome alteration in young Northeast Indian females. Chemosphere.

[106] Khanam T., Liang S., Xu S., Musstjab Akber Shah Eqani S.A., Shafqat M.N., Rasheed H., Bibi N., Shen H., Zhang J. (2023). Arsenic exposure induces urinary metabolome disruption in Pakistani male population. Chemosphere.

[107] Midya V., Lane J.M., Gennings C., Torres-Olascoaga L.A., Gregory J.K., Wright R.O., Arora M., Téllez-Rojo M.M., Eggers S. (2023). Prenatal lead exposure is associated with reduced abundance of beneficial gut microbial cliques in late childhood: An investigation using Microbial Co-Occurrence Analysis (MiCA). Environ. Sci. Technol..

[108] Zhang G., Lin W., Gao N., Lan C., Ren M., Yan L., Pan B., Xu J., Han B., Hu L. (2024). Using machine learning to construct the blood-follicle distribution models of various trace elements and explore the transport-related pathways with multiomics data. Environ. Sci. Technol..

[109] Zhao X., Chen K., Wang J., Qiu Y. (2024). Analysis of prospective genetic indicators for prenatal exposure to arsenic in newborn cord blood using machine learning. Biol. Trace Elem. Res..

[110] Khodasevich D., Holland N., Van Der Laan L., Cardenas A. (2025). A SuperLearner-based pipeline for the development of DNA methylation-derived predictors of phenotypic traits. PLoS Comput. Biol..

[111] Sung J.-M., Hung Y.-C., Wang W.-R., Chu C.-J., Lin Y.-P., Liu K.-H., Mahmudiono T., Chen H.-L. (2025). Integrating machine learning and metabolomics to uncover new biomarkers for predicting pesticide exposure among patients with kidney function decline. Sci. Total Environ..

[112] Zhang Z., Wang Y., Rodgers T.F.M., Wu Y. (2025). Exposure experiments and machine learning revealed that personal care products can significantly increase transdermal exposure of SVOCs from the environment. J. Hazard. Mater..

[113] Tan C., Chen H., Xia C. (2009). The prediction of cardiovascular disease based on trace element contents in hair and a classifier of boosting decision stumps. Biol. Trace Elem. Res..

[114] Golasik M., Jawień W., Przybyłowicz A., Szyfter W., Herman M., Golusiński W., Florek E., Piekoszewski W. (2015). Classification models based on the level of metals in hair and nails of laryngeal cancer patients: Diagnosis support or rather speculation?. Metallomics.

[115] Zabinski J.W., Garcia-Vargas G., Rubio-Andrade M., Fry R.C., Gibson J.M. (2016). Advancing dose-response assessment methods for environmental regulatory impact analysis: A Bayesian belief network approach applied to inorganic arsenic. Environ. Sci. Technol. Lett..

[116] De Benedetti S., Lucchini G., Del Bò C., Deon V., Marocchi A., Penco S., Lunetta C., Gianazza E., Bonomi F., Iametti S. (2017). Blood trace metals in a sporadic amyotrophic lateral sclerosis geographical cluster. Biometals.

[117] Lin T., Liu T., Lin Y., Yan L., Chen Z., Wang J. (2017). Comparative study on serum levels of macro and trace elements in schizophrenia based on supervised learning methods. J. Trace Elem. Med. Biol..

[118] Lin T., Liu T., Lin Y., Zhang C., Yan L., Chen Z., He Z., Wang J. (2017). Serum levels of chemical elements in esophageal squamous cell carcinoma in Anyang, China: A case-control study based on machine learning methods. BMJ Open.

[119] Park H., Kim K. (2019). Comparisons among machine learning models for the prediction of hypercholestrolemia associated with exposure to lead, mercury, and cadmium. Int. J. Environ. Res. Public Health.

[120] Cox L.A. (2020). Using Bayesian networks to clarify interpretation of exposure–response regression coefficients: Blood lead–mortality association as an example. Crit. Rev. Toxicol..

[121] Kim K., Park H. (2021). Machine-learning models predicting osteoarthritis associated with the lead blood level. Environ. Sci. Pollut. Res. Int..

[122] Monaco A., Lacalamita A., Amoroso N., D’orta A., Del Buono A., Di Tuoro F., Tangaro S., Galeandro A.I., Bellotti R. (2021). Random forests highlight the combined effect of environmental heavy metals exposure and genetic damages for cardiovascular diseases. Appl. Sci..

[123] Ximenez J.P.B., Zamarioli A., Kacena M.A., Barbosa R.M., Barbosa F. (2021). Association of urinary and blood concentrations of heavy metals with measures of bone mineral density loss: A data mining approach with the results from the National Health and Nutrition Examination Survey. Biol. Trace Elem. Res..

[124] Liu A., Cai C., Wang Z., Wang B., He J., Xie Y., Deng H., Liu S., Zeng S., Yin Z. (2022). Inductively coupled plasma mass spectrometry based urine metallome to construct clinical decision models for autism spectrum disorder. Metallomics.

[125] Xia F., Li Q., Luo X., Wu J. (2022). Identification for heavy metals exposure on osteoarthritis among aging people and machine learning for prediction: A study based on NHANES 2011–2020. Front. Public Health.

[126] Xia F., Li Q., Luo X., Wu J. (2022). Machine learning model for depression based on heavy metals among aging people: A study with National Health and Nutrition Examination Survey 2017–2018. Front. Public Health.

[127] Chan Y.N., Wang P., Chun K.H., Lum J.T.S., Wang H., Zhang Y., Leung K.S.Y. (2023). A machine learning approach for early prediction of gestational diabetes mellitus using elemental contents in fingernails. Sci. Rep..

[128] Chen Z., Liu X., Wang W., Zhang L., Ling W., Wang C., Jiang J., Song J., Liu Y., Lu D. (2023). Machine learning-aided metallomic profiling in serum and urine of thyroid cancer patients and its environmental implications. Sci. Total Environ..

[129] Chen H., Wang M., Zhang C., Li J. (2023). A methodological study of exposome based on an open database: Association analysis between exposure to metal mixtures and hyperuricemia. Chemosphere.

[130] Li W., Huang G., Tang N., Lu P., Jiang L., Lv J., Qin Y., Lin Y., Xu F., Lei D. (2023). Effects of heavy metal exposure on hypertension: A machine learning modeling approach. Chemosphere.

[131] Li X., Zhao Y., Zhang D., Kuang L., Huang H., Chen W., Fu X., Wu Y., Li T., Zhang J. (2023). Development of an interpretable machine learning model associated with heavy metals’ exposure to identify coronary heart disease among US adults via SHAP: Findings of the US NHANES from 2003 to 2018. Chemosphere.

[132] Li S., Hu X. (2023). Assessing the risk of prostate cancer with nutritional and environmental factors: A cross-sectional study from National Health and Nutrition Examination Survey 2001–2010. Nutr. Cancer.

[133] Ling W., Zhao G., Wang W., Wang C., Zhang L., Zhang H., Lu D., Ruan S., Zhang A., Liu Q. (2023). Metallomic profiling and natural copper isotopic signatures of childhood autism in serum and red blood cells. Chemosphere.

[134] Luo K.-H., Wu C.-H., Yang C.-C., Chen T.-H., Tu H.-P., Yang C.-H., Chuang H.-Y. (2023). Exploring the association of metal mixture in blood to the kidney function and tumor necrosis factor alpha using machine learning methods. Ecotoxicol. Environ. Saf..

[135] Mei P., Zhou Q., Liu W., Huang J., Gao E., Luo Y., Ren X., Huang H., Chen X., Wu D. (2023). Correlating metal exposures and dietary habits with hyperuricemia in a large urban elderly cohort by artificial intelligence. Environ. Sci. Pollut. Res. Int..

[136] Moon S., Lee J., Yu J.M., Choi H., Choi S., Park J., Choi K., Kim E., Kim H., Kim M.J. (2023). Association between environmental cadmium exposure and increased mortality in the U.S. National Health and Nutrition Examination Survey (1999–2018). J. Expo. Sci. Environ. Epidemiol..

[137] Souza M.C.O., Cruz J.C., Rocha B.A., Souza J.M.O., Devóz P.P., Santana A., Campíglia A.D., Barbosa F. (2023). The influence of the co-exposure to polycyclic aromatic hydrocarbons and toxic metals on DNA damage in brazilian lactating women and their infants: A cross-sectional study using machine learning approaches. Chemosphere.

[138] Wen J., Giri M., Xu L., Guo S. (2023). Association between exposure to selected heavy metals and blood eosinophil counts in asthmatic adults: Results from NHANES 2011–2018. J. Clin. Med..

[139] Wu Y., Deng S. Main heavy metals affecting chronic kidney disease: A study based on feature selection algorithm. Proceedings of the Eighth International Conference on Electronic Technology and Information Science (ICETIS 2023).

[140] Zhao M., Wan J., Qin W., Huang X., Chen G., Zhao X. (2023). A machine learning-based diagnosis modelling of type 2 diabetes mellitus with environmental metal exposure. Comput. Methods Programs Biomed..

[141] Angali K.A., Farhadi M., Neisi A., Cheraghian B., Ahmadi M., Takdastan A., Dargahi A., Angali Z.A. (2024). Carcinogenic and non-carcinogenic risks caused by rice contamination with heavy metals and their effect on the prevalence of cardiovascular disease (using machine learning). Food Chem. Toxicol..

[142] Chang Y., Jiang X., Dou J., Xie R., Zhao W., Cao Y., Gao J., Yao F., Wu D., Mei H. (2024). Investigating the potential risk of cadmium exposure on seizure severity and anxiety-like behaviors through the ferroptosis pathway in epileptic mice: An integrated multi-omics approach. J. Hazard. Mater..

[143] Chen H., Wang M., Li J. (2024). Exploring the association between two groups of metals with potentially opposing renal effects and renal function in middle-aged and older adults: Evidence from an explainable machine learning method. Ecotoxicol. Environ. Saf..

[144] Du G., Song X., Zhou F., Ouyang L., Li Q., Ruan S., Su R., Rao S., Zhu Y., Xie J. (2024). Association between multiple metal(loid)s exposure and blood lipid levels: Evidence from a cross-sectional study of Southeastern China. Biol. Trace Elem. Res..

[145] Fan W., Pi Z., Kong K., Qiao H., Jin M., Chang Y., Zhang J., Li H. (2024). Analyzing the impact of heavy metal exposure on osteoarthritis and rheumatoid arthritis: An approach based on interpretable machine learning. Front. Nutr..

[146] Fansler S.D., Bakulski K.M., Park S.K., Walker E., Wang X. (2024). Use of biomarkers of metals to improve prediction performance of cardiovascular disease mortality. Environ. Health.

[147] Gao X., Liu C., Yin L., Wang A., Li J., Gao Z. (2024). Machine learning model for age-related macular degeneration based on heavy metals: The National Health and Nutrition Examination Survey 2005 to 2008. Sci. Rep..

[148] Gui Y., Gui S., Wang X., Li Y., Xu Y., Zhang J. (2024). Exploring the relationship between heavy metals and diabetic retinopathy: A machine learning modeling approach. Sci. Rep..

[149] Invernizzi A., Renzetti S., Rechtman E., Ambrosi C., Mascaro L., Corbo D., Gasparotti R., Tang C.Y., Smith D.R., Lucchini R.G. (2024). Neuro-environmental interactions: A time sensitive matter. Front. Comput. Neurosci..

[150] Li B., Liu H., Mishra D., Yuan Z., Zhang Y., Zhang L., Huang Y., Zhang Y., Lin J., Chen J. (2024). The association between blood metals and cardiovascular diseases: Findings from National Health and Nutrition Examination Survey 2011–2020. Front. Cardiovasc. Med..

[151] Liu J., Li X., Zhu P. (2024). Effects of various heavy metal exposures on insulin resistance in non-diabetic populations: Interpretability analysis from machine learning modeling perspective. Biol. Trace Elem. Res..

[152] Liu P., Wang J., Mei P., Li J., Xu B., Ren X., Chen X., Wu D., Zhu F., Yang X. (2024). The interaction effect of metals exposure and dietary habit on cognitive function in Chinese older adult cohort. J. Nutr. Health Aging.

[153] Midya V., Agrawal M., Lane J.M., Gennings C., Tarassishin L., Torres-Olascoaga L.A., Eggers J., Gregory J.K., Picker M., Peter I. (2024). Association between exposure to metals during pregnancy, childhood gut microbiome, and risk of intestinal inflammation in late childhood. Environ. Health.

[154] Midya V., Nagdeo K., Lane J.M., Torres-Olascoaga L.A., Torres-Calapiz M., Gennings C., Horton M.K., Téllez-Rojo M.M., Wright R.O., Arora M. (2024). Prenatal metal exposures and childhood gut microbial signatures are associated with depression score in late childhood. Sci. Total Environ..

[155] Nabavi A., Safari F., Kashkooli M., Nabavizadeh S.S., Vardanjani H.M. (2024). Early prediction of cognitive impairment in adults aged 20 years and older using machine learning and biomarkers of heavy metal exposure. Curr. Res. Toxicol..

[156] Rog J., Łobejko Ł., Hordejuk M., Marciniak W., Derkacz R., Kiljańczyk A., Matuszczak M., Lubiński J., Nesterowicz M., Żendzian-Piotrowska M. (2024). Pro/antioxidant status and selenium, zinc and arsenic concentration in patients with bipolar disorder treated with lithium and valproic acid. Front. Mol. Neurosci..

[157] Su Z., Zhang Y., Hong S., Zhang Q., Ji Z., Hu G., Zhu X., Yuan F., Yu S., Wang T. (2024). Immune regulation patterns in response to environmental pollutant chromate exposure-related genetic damage: A cross-sectional study applying machine learning methods. Environ. Sci. Technol..

[158] Wang X., Wang X., Cheng Y., Luo C., Xia W., Gao Z., Bu W., Jiang Y., Fei Y., Shi W. (2024). Construction of metal interpretable scoring system and identification of tungsten as a novel risk factor in COPD. Ecotoxicol. Environ. Saf..

[159] Wu M., Hou W., Qin R., Wang G., Sun D., Geng Y., Du Y. (2024). Comparative mathematical modeling of causal association between metal exposure and development of chronic kidney disease. Front. Endocrinol..

[160] Yan L., You H., Wang H., Ding C., He B., Wang J., Fang W., Lin Y., Kang D., Chen F. (2024). Association of multiple trace metals in scalp hair with glioma risk: The mediating role of inflammation. Ann. Clin. Transl. Neurol..

[161] Yao J., Du Z., Yang F., Duan R., Feng T. (2024). The relationship between heavy metals and metabolic syndrome using machine learning. Front. Public Health.

[162] Xiao H., Liang X., Li H., Chen X., Li Y. (2024). Trends in the prevalence of osteoporosis and effects of heavy metal exposure using interpretable machine learning. Ecotoxicol. Environ. Saf..

[163] Xu S., Sun M. (2024). The interpretable machine learning model associated with metal mixtures to identify hypertension via EMR mining method. J. Clin. Hypertens..

[164] Xu S., Sun M. (2024). Assessment of EMR ML mining methods for measuring association between metal mixture and mortality for hypertension. High Blood Press. Cardiovasc. Prev..

[165] Zhao R., Lin S., Han M., Lin Z., Yu M., Zhang B., Ma L., Li D., Peng L. (2024). Association between machine learning-assisted heavy metal exposures and diabetic kidney disease: A cross-sectional survey and Mendelian randomization analysis. Front. Public Health.

[166] Zibibula Y., Tayier G., Maimaiti A., Liu T., Lu J. (2024). Machine learning approaches to identify the link between heavy metal exposure and ischemic stroke using the US NHANES data from 2003 to 2018. Front. Public Health.

[167] Zuo W., Yang X. (2024). A machine learning model predicts stroke associated with blood cadmium level. Sci. Rep..

[168] Chen J. (2025). Development of a machine learning model related to explore the association between heavy metal exposure and alveolar bone loss among US adults utilizing SHAP: A study based on NHANES 2015–2018. BMC Public Health.

[169] Chen J., Zeng H., Pan Z., Li M., Zhou Q., Chen K., Hao Y., Cao X., Zhang L., Wang Q. (2025). Association between metal mixture in urine and abnormal blood pressure and mediated effect of oxidative stress based on BKMR and machine learning method. Ecotoxicol. Environ. Saf..

[170] Cox L.A., Lewis R.J., Rege S.V., Singh S. (2025). AI-assisted exposure-response data analysis: Quantifying heterogeneous causal effects of exposures on survival times. Glob. Epidemiol..

[171] Gu X., Li Q., Wang X. (2025). Using Life’s Essential 8 and heavy metal exposure to determine infertility risk in American women: A machine learning prediction model based on the SHAP method. Front. Endocrinol..

[172] He J., Zhou W., Zhang H., Shen J. (2025). In vivo heavy metal and diabetes association: A cross-sectional interpretable machine learning analysis of NHANES. Int. J. Diabetes Dev. Ctries..

[173] Hu H., Wu Y., Liu J., Zhao M., Xie P. (2025). The relationship between metal exposure and HPV infection: Evidence from explainable machine learning methods. Biol. Trace Elem. Res..

[174] Jin H., Zhang L., Sun Y., Xu Y., Luo M. (2025). Developing machine learning models for predicting cardiovascular disease survival based on heavy metal serum and urine levels. Front. Public Health.

[175] Jin X., Li L., Hu X., Bi P., Zhang S., Wang Q., Xiao Z., Yang H., Liu T., Feng L. (2025). Association of urinary metal elements with sarcopenia and glucose metabolism abnormalities: Insights from NHANES data using machine learning approaches. Ecotoxicol. Environ. Saf..

[176] Johnson H., Longden J., Cameron G., Waiter G.D., Waldron F.M., Gregory J.M., Spence H. (2025). Machine learning identifies routine blood tests as accurate predictive measures of pollution-dependent poor cognitive function. BioRxiv.

[177] Li G., Zhang T., He K., Zhang M., Hu J., Ge T., Wang M., Zou R., Fan X. (2025). Deciphering the influence of heavy metals on adverse cardiovascular events using machine learning. Toxicol. Lett..

[178] Li Y., Tao R., Feng Z., Mei G., Liu Z., Yang W., Mo F., Liu Z. (2025). Exploring the association between heavy metal exposure and periodontitis using interpretable machine learning models: NHANES 2009–2014. Hum. Ecol. Risk Assess..

[179] Mi Y., Sun P. (2025). Machine learning-based prediction of hearing loss: Findings of the US NHANES from 2003 to 2018. Hear. Res..

[180] Nabavi A., Kashkooli M., Nabavizadeh S.S., Safari F. (2025). Heavy metal biomarkers and their impact on hearing loss risk: A machine learning framework analysis. Front. Public Health.

[181] Nahar S., Wang Z.J., Chakrabarty S., Jin X. Leveraging selected lifestyle factors from NHANES data for chronic heart disease risk prediction. Proceedings of the 2025 Intermountain Engineering, Technology and Computing (IETC).

[182] Ren F., Zhao X., Yang Q., Liao H., Zhang Y., Liu X. (2025). A machine learning framework for predicting cognitive impairment in aging populations using urinary metal and demographic data. Front. Genet..

[183] Shen M., Zhang Y., Zhan R., Du T., Shen P., Lu X., Liu S., Guo R., Shen X. (2025). Predicting the risk of cardiovascular disease in adults exposed to heavy metals: Interpretable machine learning. Ecotoxicol. Environ. Saf..

[184] Shi C., Jiang H., Zhao F., Zhang Y., Chen H. (2025). Blood metal levels predict digestive tract cancer risk using machine learning in a U.S. cohort. Sci. Rep..

[185] Wan S., Yang Y., Zhao Q., Xing Z., Li J., Gao H., Yin Y., Liu Z., Chen Q., Tian M. (2025). Proteomic signatures and predictive modeling of cadmium-associated anxiety in middle-aged and elderly populations: An environmental exposure association study. J. Transl. Med..

[186] Wang X., Chen G., He R., Gao Y., Lu J., Xu T., Liu H., Jiang Z. (2025). Machine learning prediction of glaucoma by heavy metal exposure: Results from the National Health and Nutrition Examination Survey 2005 to 2008. Sci. Rep..

[187] Wen J., Wang C., Liu R., Zhuang R., Liu Y., Li Y., Guo S. (2025). Systemic inflammation mediates the relationship between urinary cadmium and chronic cough risk: Findings based on multiple statistical models. Biometals.

[188] Wu Z., Jiang S., Li J., Wang P., Chen Y. (2025). Association between urinary cadmium levels and increased gallstone disease in US adults. Sci. Rep..

[189] Xia T., Han K. (2025). Machine learning prediction model with shap interpretation for chronic bronchitis risk assessment based on heavy metal exposure: A nationally representative study. BMC Pulm. Med..

[190] Xu S., Sun M. (2025). The interpretable machine learning model for depression associated with heavy metals via EMR mining method. Sci. Rep..

[191] Yuting Y., Shan D. (2025). Associations between urinary and blood heavy metal exposure and heart failure in elderly adults: Insights from an interpretable machine learning model based on NHANES (2003–2020). Int. J. Cardiol. Cardiovasc. Risk Prev..

[192] Zhang Y., Li Q., Wang X. (2025). Associations between exposure to heavy metal and sarcopenia prevalence: A cross-sectional study using NHANES data. Front. Public Health.

[193] Zhang N., Xu Y., Liang H., Wang Q., An Y., Gao H., Zhao J., Wang H. (2025). Diagnostic value of small dense low-density lipoprotein and trace elements in coronary artery disease. Ann. Biol. Clin..

[194] Zhong Y., Bao Y., Cheng H., Liu C., Huang S., Qiu H., Huang H., Ren J., Jin H., He C. (2025). A negative combined effect of exposure to maternal Mn-Cu-Rb-Fe metal mixtures on gestational anemia, and the mediating role of creatinine in the Guangxi Birth Cohort Study (GBCS): Twelve machine learning algorithms. Ecotoxicol. Environ. Saf..

[195] Feng Y., Su S., Lin W., Ren M., Gao N., Pan B., Zhang L., Jin L., Zhang Y., Li Z. (2023). Using machine learning to expedite the screening of environmental factors associated with the risk of spontaneous preterm birth: From exposure mixtures to key molecular events. Environ. Sci. Technol. Lett..

[196] Eve A.A., Tunc E., Mehta D., Yoo J.Y., Yilmaz H.E., Emren S.V., Akçay F.A., Erdogan Z.M. (2024). PFAS and their association with the increased risk of cardiovascular disease in postmenopausal women. Toxicol. Sci..

[197] Li Z., Xu X., Zhang K. (2025). Exploring the relationship between per- and polyfluoroalkyl substances exposure and rheumatoid arthritis risk using interpretable machine learning. Front. Public Health.

[198] Shao X., Zhang L., Wang Y., Ying Y., Chen X. (2025). Developing an interpretable machine learning predictive model of chronic obstructive pulmonary disease by serum PFAS concentration. Front. Public Health.

[199] Wang F., Lin Y., Qin L., Zeng X., Jiang H., Liang Y., Wen S., Li X., Huang S., Li C. (2025). Serum metabolome associated with novel and legacy per- and polyfluoroalkyl substances exposure and thyroid cancer risk: A multi-module integrated analysis based on machine learning. Environ. Int..

[200] Wang C., Xu X., Luo S., Luo M., Li S., Si J. (2025). Interpretable machine learning insights into the association between PFAS exposure and diabetes mellitus. Ecotoxicol. Environ. Saf..

[201] Yang J., Wang T., Li K., Wāng Y. (2025). Associations between per- and polyfluoroalkyl chemicals and abdominal aortic calcification in middle-aged and older adults. J. Adv. Res..

[202] Zheng Z., Xu Y., Kang N., Dong Y., Wang X., Zhang L., Bian H., Zeng Q. (2025). Assessing the effect of perfluoroalkyl and polyfluoroalkyl substances on cardiovascular-kidney-metabolic syndrome: Insights from an interpretable machine learning model. Sci. Total Environ..

[203] Colicino E., de Water E., Just A.C., Navarro E., Pedretti N.F., McRae N., Braun J.M., Schnaas L., Rodríguez-Carmona Y., Hernández C. (2021). Prenatal urinary concentrations of phthalate metabolites and behavioral problems in Mexican children: The Programming Research in Obesity, Growth Environment and Social Stress (PROGRESS) study. Environ. Res..

[204] Lu L., Qian Y., Dong Y., Su H., Deng Y., Zeng Q., Li H. (2023). A systematic study of the performance of machine learning models on analyzing the association between semen quality and environmental pollutants. Front. Phys..

[205] You L.-M., Zhang D.-C., Lin C.-S., Lan Q. (2024). Phthalate metabolites were related to the risk of high-frequency hearing loss: A cross-sectional study of National Health and Nutrition Examination Survey. J. Multidiscip. Healthc..

[206] Liu Y., Li K., Zhang Y., Cai Y., Liu X., Jia Y., Yao P., Wei X., Wu H., Liu X. (2025). Impact of phthalate exposure and blood lipids on breast cancer risk: Machine learning prediction. Environ. Sci. Eur..

[207] Wu H.-T., Liao C.-C., Peng C.-F., Lee T.-Y., Liao P.-H. (2025). Exploring the application of machine learning to identify the correlations between phthalate esters and disease: Enhancing nursing assessments. Health Inf. Sci. Syst..

[208] Oh R., Lee H.K., Pak Y.K., Oh M.-S. (2022). An interactive online app for predicting diabetes via machine learning from environment-polluting chemical exposure data. Int. J. Environ. Res. Public Health.

[209] Sharma A., Hooda N., Gupta N.R., Hura G.S., Singh A.K., Siong Hoe L. (2021). Breast cancer recurrence prediction in biopsy using machine learning framework. Advances in Communication and Computational Technology (ICACCT 2019).

[210] Sharma A., Hooda N., Gupta N.R., Sharma R., Tiwari R., Koundal D., Upadhyay S. (2023). Efficient BREV ensemble framework: A case study of breast cancer prediction. Image Based Computing for Food and Health Analytics: Requirements, Challenges, Solutions and Practices.

[211] Gao Y., Lu H., Zhou H., Tan J. (2024). Exploring the impact of polychlorinated biphenyls on comorbidity and potential mitigation strategies. Front. Public Health.

[212] Liu Y., Li K., Li C., Feng Z., Cai Y., Zhang Y., Hu Y., Wei X., Yao P., Liu X. (2024). Pesticides, cancer, and oxidative stress: An application of machine learning to NHANES data. Environ. Sci. Eur..

[213] Tan J., Ma M., Shen X., Xia Y., Qin W. (2024). Potential lethality of organochlorine pesticides: Inducing fatality through inflammatory responses in the organism. Ecotoxicol. Environ. Saf..

[214] Jiang Y.-X., Gui S.-Y., Sun X.-D. (2025). Associations between organophosphorus pesticides exposure and age-related macular degeneration risk in U.S. adults: Analysis from interpretable machine learning approaches. Int. J. Ophthalmol..

[215] Liu J., Wang B., Li Q. (2025). Machine learning model for age related macular degeneration based on pesticides: The National Health and Nutrition Examination Survey 2007–2008. Front. Public Health.

[216] Pan D., Zhou L., Mu C., Lin M., Sheng Y., Xu Y., Huang D., Liu S., Zeng X., Chongsuvivatwong V. (2025). Effects of neonicotinoid pesticide exposure in the first trimester on gestational diabetes mellitus based on interpretable machine learning. Environ. Res..

[217] Shamma S., Hussein M.A., El-Nahrery E.M.A., Shahat A., Shoeib T., Abdelnaser A. (2025). Leveraging machine learning in precision medicine to unveil organochlorine pesticides as predictive biomarkers for thyroid dysfunction. Sci. Rep..

[218] Wang X., Tian M., Shen Z., Tian K., Fei Y., Cheng Y., Ruan J., Mo S., Dai J., Xia W. (2025). Comprehensive cross-sectional study of the triglyceride glucose index, organophosphate pesticide exposure, and cardiovascular diseases: A machine learning integrated approach. Toxics.

[219] Yang X., Zhang Y., Xu Y., Xu Y., Zhang M., Guan Q., Hu W., Tun H.M., Xia Y. (2025). Microbial disturbances caused by pesticide exposure and their predictive implications for gestational diabetes mellitus. Environ. Sci. Technol..

[220] Fu Q., Wu Y., Zhu M., Xia Y., Yu Q., Liu Z., Ma X., Yang R. (2024). Identifying cardiovascular disease risk in the U.S. population using environmental volatile organic compounds exposure: A machine learning predictive model based on the SHAP methodology. Ecotoxicol. Environ. Saf..

[221] Liu X., Chang Y., Xu C., Li Y., Wang Y., Sun Y., Duan M., Li W., Cui J. (2024). Association of volatile organic compound levels with chronic obstructive pulmonary diseases in NHANES 2013–2016. Sci. Rep..

[222] Deng C., Jiang Y., Lin Y., Liang H., Wang W., Huang Y., He J. (2025). Exploring the potential associations between single and mixed volatile compounds and preserved ratio impaired spirometry using five different approaches. Ecotoxicol. Environ. Saf..

[223] Jiang L., Wang H., Xiao Y., Xu L., Chen H. (2025). Exploring the association between volatile organic compound exposure and chronic kidney disease: Evidence from explainable machine learning methods. Ren. Fail..

[224] Zhang Y., Qiu X., Wu Z., Li Y., Shen X., Wu J., Cao P., Sun Z., Wang W. (2025). Comprehensive analysis of the association between volatile organic compound pollutants and chronic kidney disease in hypertensive populations: Insights from multi-omics approaches and identification of potential therapeutic targets. Environ. Res..

[225] Chen Y., Shen X., Li G., Yue S., Liang C., Hao Z. (2022). Association between aldehyde exposure and kidney stones in adults. Front. Public Health.

[226] Liu L., Zhou H., Wang X., Wen F., Zhang G., Yu J., Shen H., Huang R. (2024). Effects of environmental phenols on eGFR: Machine learning modeling methods applied to cross-sectional studies. Front. Public Health.

[227] Cai Y., Huang X.-R., Wang S.-J., Liang Y.-C., Liu D.-L., Chu S.-F., Li H.-L. (2025). Effect of the exposure to brominated flame retardants on hyperuricemia using interpretable machine learning algorithms based on the SHAP methodology. PLoS ONE.

[228] Mulisa G., Pero-Gascon R., McCormack V., Bisanz J.E., Talukdar F.R., Abebe T., De Boevre M., De Saeger S. (2025). Multiple mycotoxin exposure assessment through human biomonitoring in an esophageal cancer case-control study in the Arsi-Bale districts of Oromia region of Ethiopia. Int. J. Hyg. Environ. Health.

[229] Tian Y., Gao S., Zhang F., Wan X., Jia W., Jiao J., Fan Y., Zhang Y. (2025). A new method for internal urinary metabolite exposure and dietary exposure association assessment of 3-MCPD and glycidol and their esters based on machine learning. Ecotoxicol. Environ. Saf..

[230] Xie Q., Qu H., Li J., Zeng R., Li W., Ouyang R., Zhang C., Xie S., Du M. (2025). Identifying emphysema risk using brominated flame retardants exposure: A machine learning predictive model based on the SHAP methodology. Front. Public Health.

[231] Xu Q., Si K., Wang W., Li Y., Zhang Y., Gaskins A.J., Messerlian C., Mustieles V., Xiong C.-L., Pan A. (2025). Organophosphate flame retardants, obesity, and depressive symptoms among 1019 young healthy men. Environ. Health.

[232] Krysiak-Baltyn K., Toppari J., Skakkebaek N.E., Jensen T.S., Virtanen H.E., Schramm K.W., Shen H., Vartiainen T., Kiviranta H., Taboureau O. (2012). Association between chemical pattern in breast milk and congenital cryptorchidism: Modelling of complex human exposures. Int. J. Androl..

[233] Oskar S., Wolff M.S., Teitelbaum S.L., Stingone J.A. (2021). Identifying environmental exposure profiles associated with timing of menarche: A two-step machine learning approach to examine multiple environmental exposures. Environ. Res..

[234] Li W., Huang G., Tang N., Lu P., Jiang L., Lv J., Qin Y., Lin Y., Xu F., Lei D. (2023). Association between co-exposure to phenols, phthalates, and polycyclic aromatic hydrocarbons with the risk of frailty. Environ. Sci. Pollut. Res. Int..

[235] Llopis M., Ventura P.S., Brachowicz N., Sangüesa J., Murcia M., Lopez-Espinosa M.J., García-Baquero G., Lertxundi A., Vrijheid M., Casas M. (2023). Sociodemographic, lifestyle, and environmental determinants of vitamin D levels in pregnant women in Spain. Environ. Int..

[236] Soomro M.H., England-Mason G., Liu J., Reardon A.J.F., MacDonald A.M., Kinniburgh D.W., Martin J.W., Dewey D. (2023). Associations between the chemical exposome and pregnancy induced hypertension. Environ. Res..

[237] Feng Z., Chen Y., Guo Y., Lyu J. (2024). Deciphering the environmental chemical basis of muscle quality decline by interpretable machine learning models. Am. J. Clin. Nutr..

[238] Guo K., Ni W., Du L., Zhou Y., Cheng L., Zhou H. (2024). Environmental chemical exposures and a machine learning-based model for predicting hypertension in NHANES 2003–2016. BMC Cardiovasc. Disord..

[239] Liu S., Lu L., Wang F., Han B., Ou L., Gao X., Luo Y., Huo W., Zeng Q. (2024). Building a predictive model for hypertension related to environmental chemicals using machine learning. Environ. Sci. Pollut. Res. Int..

[240] Soomro M.H., England-Mason G., Reardon A.J.F., Liu J., MacDonald A.M., Kinniburgh D.W., Martin J.W., Dewey D. (2024). Maternal exposure to bisphenols, phthalates, perfluoroalkyl acids, and trace elements and their associations with gestational diabetes mellitus in the APrON cohort. Reprod. Toxicol..

[241] Yang L., Zhang T., Gao Y., Li D., Cui R., Gu C., Wang L., Sun H. (2024). Quantitative identification of the co-exposure effects of e-waste pollutants on human oxidative stress by explainable machine learning. J. Hazard. Mater..

[242] Deng C., Jiang Y., Lin Y., Liang H., Wang W., Huang Y., He J. (2025). Potential effects of endocrine-disrupting chemicals on preserved ratio impaired spirometry revealed by five different approaches. Ecotoxicol. Environ. Saf..

[243] England-Mason G., MacEachern S.J., Amador K., Soomro M.H., Reardon A.J.F., MacDonald A.M., Kinniburgh D.W., Letourneau N., Giesbrecht G.F., Martin J.W. (2025). Using machine learning to investigate the influence of the prenatal chemical exposome on neurodevelopment of young children. Neurotoxicology.

[244] Jo Y., Shin M.Y., Kim S. (2025). Assessing the association of multi-environmental chemical exposures on metabolic syndrome: A machine learning approach. Environ. Int..

[245] Lee I., Noh J., Kim Y., An J.N., Park J.Y., Kim Y.C., Lee J., Lee J.P., Lee J.S., Choi K. (2025). A novel approach to the relation of multi-pollutant effect and kidney dysfunction: Data analysis from the Korean National Environmental Health Survey Cycle 3 (2015–2017). Kidney Res. Clin. Pract..

[246] Liu H., Gu H., Li J., Fang Y., Yang S., Liang G. (2025). Evaluating the relationship between environmental chemicals and obesity: Evidence from a machine learning perspective. Ecotoxicol. Environ. Saf..

[247] Liu S., Wang H., Cao Y., Lu L., Wu Y., Lian F., Yang J., Song Q. (2025). The association between low-concentration heavy metal exposure and chronic kidney disease risk through α-klotho. Sci. Rep..

[248] Lu X., Kou H., Li C., Zhan R., Guo R., Liu S., Shen P., Shen M., Du T., Lu J. (2025). Development and validation of an interpretable machine learning model for predicting hyperuricemia risk: Based on environmental chemical exposure. Ecotoxicol. Environ. Saf..

[249] Shi Y., Li K., Ding R., Li X., Cheng Z., Liu J., Liu S., Zhu H., Sun H. (2025). Untargeted metabolomics and machine learning unveil the exposome and metabolism linked with the risk of early pregnancy loss. J. Hazard. Mater..

[250] Zhang B., Chen L., Li T. (2025). Unveiling the effect of urinary xenoestrogens on chronic kidney disease in adults: A machine learning model. Ecotoxicol. Environ. Saf..

[251] Luo J., Hendryx M. (2020). Metal mixtures and kidney function: An application of machine learning to NHANES data. Environ. Res..

[252] Liu M., Li M., Guo W., Zhao L., Yang H., Yu J., Liu L., Fang Q., Lai X., Yang L. (2022). Co-exposure to priority-controlled metals mixture and blood pressure in Chinese children from two panel studies. Environ. Pollut..

[253] Michael T., Kohn E., Daniel S., Hazan A., Berkovitch M., Brik A., Hochwald O., Borenstein-Levin L., Betser M., Moskovich M. (2022). Prenatal exposure to heavy metal mixtures and anthropometric birth outcomes: A cross-sectional study. Environ. Health.

[254] Su F., Zeeshan M., Xiong L.-H., Lv J.-Y., Wu Y., Tang X.-J., Zhou Y., Ou Y.-Q., Huang W.-Z., Feng W.-R. (2022). Co-exposure to perfluoroalkyl acids and heavy metals mixtures associated with impaired kidney function in adults: A community-based population study in China. Sci. Total Environ..

[255] Takatani T., Eguchi A., Yamamoto M., Sakurai K., Takatani R., Taniguchi Y., Nakayama S.F., Mori C., Kamijima M. (2022). Individual and mixed metal maternal blood concentrations in relation to birth size: An analysis of the Japan Environment and Children’s Study (JECS). Environ. Int..

[256] Borghese M.M., Fisher M., Ashley-Martin J., Fraser W.D., Trottier H., Lanphear B., Johnson M., Helewa M., Foster W., Walker M. (2023). Individual, independent, and joint associations of toxic metals and manganese on hypertensive disorders of pregnancy: Results from the MIREC Canadian pregnancy cohort. Environ. Health Perspect..

[257] Chen Y., Zhao A., Li R., Kang W., Wu J., Yin Y., Tong S., Li S., Chen J. (2023). Independent and combined associations of multiple-heavy-metal exposure with lung function: A population-based study in US children. Environ. Geochem. Health.

[258] Gao H., Zhu N., Deng S., Du C., Tang Y., Tang P., Xu S., Liu W., Shen M., Xiao X. (2023). Combination effect of microcystins and arsenic exposures on CKD: A case-control study in China. Toxins.

[259] Liang J., Pu Y., Liu M., Bao W., Zhang Y., Hu L., Huang S., Jiang N., Huang S., Pu X. (2023). Synergistic impact of co-exposures to whole blood metals on chronic kidney disease in general US adults: A cross sectional study of the National Health and Nutrition Examination Survey 2011–2020. Environ. Sci. Pollut. Res. Int..

[260] Ma J., Geng S., Sun Q., Zhang X., Han L., Yao X., Zhang B., Zhu L., Wen J. (2023). Exposure to metal mixtures and young children’s growth and development: A biomonitoring-based study in Eastern China. Ecotoxicol. Environ. Saf..

[261] Shen Z., Wang R., He P., Zhang Z., Dai Y., Li M., Liu Z., Yang H., Guan S., Sun J. (2023). Association between urinary metal concentrations and abnormal estimated glomerular filtration rate in Chinese community-dwelling elderly: Exploring the mediating effect of triglycerides. Ecotoxicol. Environ. Saf..

[262] Wu Z., Guan T., Cai D., Su G. (2023). Exposure to multiple metals in adults and diabetes mellitus: A cross-sectional analysis. Environ. Geochem. Health.

[263] Yu Y., Meng W., Kuang H., Chen X., Zhu X., Wang L., Tan H., Xu Y., Ding P., Xiang M. (2023). Association of urinary exposure to multiple metal(loid)s with kidney function from a national cross-sectional study. Sci. Total Environ..

[264] An Q., Wang Q., Liu R., Zhang J., Li S., Shen W., Zhou H., Liang Y., Li Y., Mu L. (2024). Analysis of relationship between mixed heavy metal exposure and early renal damage based on a weighted quantile sum regression and Bayesian kernel machine regression model. J. Trace Elem. Med. Biol..

[265] Fan G., Liu Q., Wu M., Bi J., Qin X., Fang Q., Mei S., Wan Z., Lv Y., Song L. (2024). Association between multiple metal exposure and bone mineral density among Chinese adults. Environ. Geochem. Health.

[266] Fu Y., He M., Liu Y., Li M., Zhu M., Wang Y., Lin W., Yu L., Yang L., Zhang Y. (2024). Reduction of haemoglobin is related to metal mixtures exposure in Chinese preschoolers: Joint effect models. J. Trace Elem. Med. Biol..

[267] Huang J., Zhang Y., King L., Wang J., Nie P., Xie Q., Chen H., Wan X., Li Z., Zhao Y. (2024). Associations of urinary heavy metals with age at menarche, age at menopause, and reproductive lifespan: A cross-sectional study in U.S. women. Ecotoxicol. Environ. Saf..

[268] Kim I.-G., Hong S., Yim S., Jeong J.-H., Choi K., Lee J.-H., Hong Y.-S., Eom S.-Y., Kim H., Kim Y.-D. (2024). Sex-specific effects of combined heavy metal exposure on blood pressure: A Bayesian Kernel Machine Regression analysis. Atmosphere.

[269] Long J., Huang H., Tang P., Liang J., Liao Q., Chen J., Pang L., Yang K., Wei H., Chen M. (2024). Associations between maternal exposure to multiple metals and metalloids and blood pressure in preschool children: A mixture-based approach. J. Trace Elem. Med. Biol..

[270] Pan S., Niu Y., Duan S., Zhao D., Wang Q., Dong Z., Cai G., Chen X. (2024). Uric acid mediates the relationship between mixed heavy metal exposure and renal function in older adult people. Front. Public Health.

[271] Qiao G., Shen Z., Duan S., Wang R., He P., Zhang Z., Dai Y., Li M., Chen Y., Li X. (2024). Associations of urinary metal concentrations with anemia: A cross-sectional study of Chinese community-dwelling elderly. Ecotoxicol. Environ. Saf..

[272] Schildroth S., Valeri L., Kordas K., Shi B., Friedman A., Smith D., Placidi D., Wright R.O., Lucchini R.G., White R.F. (2024). Assessing the mediating role of iron status on associations between an industry-relevant metal mixture and verbal learning and memory in Italian adolescents. Sci. Total Environ..

[273] Wei J., Fu D., Guo S., Tian T., Huang Y., Li Z., Wang L., Jin L., Ye W., Ren A. (2024). Elementomics of 32 elements in cord serum depicts the risk of orofacial clefts: A case-control study in Shanxi, China. Environ. Pollut..

[274] Xiang Y., Wang Y., Deng Y., Wang T., Chen J., He M. (2024). Independent and joint associations of multiple metals exposure with vital capacity index: A cross-sectional study in Chinese children and adolescents. Int. Arch. Occup. Environ. Health.

[275] Yang X., Li L., Nie L. (2024). Associations between co-exposure to heavy metals and vertebral compression fracture, as well as femoral neck bone mineral density: A cross-sectional study from NHANES data. PLoS ONE.

[276] Zhou X., Jin H., Zhang Y. (2024). Urinary metals are associated with obesity in U.S. children and adolescents: A cross-sectional study. Nutr. Res..

[277] Go Y.-Y., Hur Y.M., You Y.-A., Park S., Lee G., Chae R., Kim S.-M., Kim Y.J. (2025). Association of maternal multi-metal exposure and dyslipidemia: A study of air pollution on pregnancy outcomes. BMC Pregnancy Childbirth.

[278] Ji R., Wu H., Lin H., Li Y., Shi Y. (2025). Cadmium and selenium blood levels in association with congestive heart failure in diabetic and prediabetic patients: A cross-sectional study from the national health and nutrition examination survey. Diabetol. Metab. Syndr..

[279] Rodríguez D., Lima S.M., Li C., Schildroth S., Xu M., Kordas K. (2025). Associations of a metal mixture and vitamin D with sleep duration among adolescents and young adults from the 2011–2018 NHANES cycles. J. Trace Elem. Med. Biol..

[280] Yang C., Zhang J., Liu H., Hong Q., Fan Y., An J., Zhang H., Shen X., Dong X. (2025). Health effects of mixed metal exposure on accelerating aging among the elderly population. Ecotoxicol. Environ. Saf..

[281] Yu M., Xun J., Ge Y., Li X., Chen X., Cui L., Wang X., Zhang M., Xing Z., Deng L. (2025). Relationship between internal metal exposure and thyroid cancer incidence: A case-control study simultaneously validated by BKMR and WQS models. Food Chem. Toxicol..

[282] Borghese M.M., Liang C.L., Owen J., Fisher M. (2022). Individual and mixture associations of perfluoroalkyl substances on liver function biomarkers in the Canadian Health Measures Survey. Environ. Health.

[283] Guo J., Huang S., Yang L., Zhou J., Xu X., Lin S., Li H., Xie X., Wu S. (2023). Association between polyfluoroalkyl substances exposure and sex steroids in adolescents: The mediating role of serum albumin. Ecotoxicol. Environ. Saf..

[284] Li S., Wang C., Yang C., Chen Y., Cheng Q., Liu J., Zhang Y., Jin L., Li Z., Ren A. (2024). Prenatal exposure to poly/perfluoroalkyl substances and risk for congenital heart disease in offspring. J. Hazard. Mater..

[285] Wang T., Yang J., Han Y., Wāng Y. (2024). Unveiling the intricate connection between per- and polyfluoroalkyl substances and prostate hyperplasia. Sci. Total Environ..

[286] Abuduxukuer K., Wang H., Wang C., Luo X., Zeng X., Da D., Yu J., Lu W., Zhang J., Zhang Y. (2025). Prenatal exposure to per-and polyfluoroalkyl substances and its association with Developmental Defects of Enamel (DDE) and dental caries in 4 years old children: Findings from Shanghai birth cohort. Environ. Int..

[287] Borghese M.M., Feng J., Liang C.L., Kienapple N., Manz K.E., Fisher M., Arbuckle T.E., Atlas E., Braun J.M., Bouchard M.F. (2025). Legacy, alternative, and precursor PFAS and associations with lipids and liver function biomarkers: Results from a cross-sectional analysis of adult females in the MIREC-ENDO study. Int. J. Hyg. Environ. Health.

[288] Liao Q., Liang X., Huang H., Tang P., Liang J., Long J., Ou L., Wen J., Sheng Y., Li H. (2025). Association between prenatal exposure to per- and polyfluoroalkyl substances and blood pressure among preschool-aged children: The moderating effect of child-age and the mediating effect of inflammatory cytokine. Ecotoxicol. Environ. Saf..

[289] Liu Y., Yao J., Ren M., Ye L., Pan A.-P., Xu X. (2025). Association of per-and polyfluoroalkyl substance exposure with cataract prevalence among U.S. adults: A NHANES analysis (2005–2008). Transl. Vis. Sci. Technol..

[290] Yang L.X., Zhai J., Chen Z.J., Du Y. (2025). Female-specific associations of serum perfluoroalkyl and polyfluoroalkyl substances with sex hormonal/insulin dysregulation: An integrated population-based study. Ecotoxicol. Environ. Saf..

[291] Hou J., Yin W., Li P., Huang Y., Wan Y., Hu C., Xu T., Cheng J., Wang L., Yu Z. (2019). Effect of exposure to phthalates on association of polycyclic aromatic hydrocarbons with 8-hydroxy-2′-deoxyguanosine. Sci. Total Environ..

[292] Kang X., Li J., Luo J., Zhang D. (2022). Associations between organophosphate esters metabolites and sleep disorder and trouble sleeping in adults: A machine-learning approach. Environ. Sci. Pollut. Res. Int..

[293] Cano-Sancho G., Warembourg C., Güil N., Stratakis N., Lertxundi A., Irizar A., Llop S., Lopez-Espinosa M.J., Basagaña X., González J.R. (2023). Nutritional modulation of associations between prenatal exposure to persistent organic pollutants and childhood obesity: A prospective cohort study. Environ. Health Perspect..

[294] Hu P., Ke S., Vinturache A., Chen Y., Ding G., Zhang Y. (2023). Organophosphate esters, airway inflammation, and lung function among U.S. participants: A nationally representative cross-sectional study from NHANES, 2011–2012. Sci. Total Environ..

[295] Deng F., He J., Dai Y., Peng R., Pan X., Yuan J., Tan L. (2024). Biomonitoring urinary pesticide metabolites in preschool children by supported liquid extraction and ultra-high performance liquid chromatography-tandem mass spectrometry and their association with oxidative stress. J. Chromatogr. A.

[296] Deng Y., Yi S., Liu W., Yang L., Zhu L., Zhang Q., Jin H., Yang R., Wang R., Tang N.-J. (2024). Identification of primary organophosphate esters contributing to enhanced risk of gestational diabetes mellitus based on a case-control study. Environ. Sci. Technol..

[297] Wu X., Liu Q., Li Y., Yue M., Su Q., Luo J., Li Y., Zeng S., Gao J. (2024). Urinary neonicotinoid concentrations and obesity: A cross-sectional study among Chinese adolescents. Environ. Pollut..

[298] Duan S., Wu Y., Zhu J., Wang X., Fang Y. (2024). Associations of polycyclic aromatic hydrocarbons mixtures with cardiovascular diseases mortality and all-cause mortality and the mediation role of phenotypic ageing: A time-to-event analysis. Environ. Int..

[299] Lu X., Zhou Y., Miao Q., Han X., Zhou Y., Zhao G., Yu H., Chen M. (2024). Independent and joint associations between urinary polycyclic aromatic hydrocarbon metabolites and cognitive function in older adults in the United States. Front. Public Health.

[300] Pi X., Liu C., Jia X., Zhang Y., Liu J., Wang B., Wang L., Li Z., Ren A., Jin L. (2024). Periconceptional polycyclic aromatic hydrocarbon levels in maternal hair and fetal risk for congenital heart defects. Ecotoxicol. Environ. Saf..

[301] Wei J., Wang Y., Kong H., Wu J., Jiang L., Pan B., Guo S., Yang F., Liu G., Qiu F. (2024). Association between plasma CC16 levels and lung function changes in coke oven workers: A cohort study from 2014 to 2023. Ecotoxicol. Environ. Saf..

[302] Wu L., Lu X., Zhang S., Zhong Y., Gao H., Tao F., Wu X. (2024). Co-exposure effects of urinary polycyclic aromatic hydrocarbons and metals on lung function: Mediating role of systematic inflammation. BMC Pulm. Med..

[303] Zang X., Zhou W., Zhang H., Zang X. (2024). Using four machine learning methods to analyze the association between polycyclic aromatic hydrocarbons and visual impairment in American adults: Evidence from NHANES. Toxics.

[304] Zhang L., Yang X. (2024). Association between exposure to polycyclic aromatic hydrocarbons and endometriosis: Data from the NHANES 2001–2006. Front. Public Health.

[305] Liu W., Cao S., Shi D., Yu L., Qiu W., Chen W., Wang B. (2023). Single-chemical and mixture effects of multiple volatile organic compounds exposure on liver injury and risk of non-alcoholic fatty liver disease in a representative general adult population. Chemosphere.

[306] Wei C., Cao L., Zhou Y., Zhang W., Zhang P., Wang M., Xiong M., Deng C., Xiong Q., Liu W. (2023). Multiple statistical models reveal specific volatile organic compounds affect sex hormones in American adult male: NHANES 2013–2016. Front. Endocrinol..

[307] Wang Y., Meng Z., Wei S., Li X., Su Z., Jiang Y., Wu H., Pan H., Wang J., Zhou Q. (2024). Urinary volatile organic compound metabolites and COPD among US adults: Mixture, interaction and mediation analysis. Environ. Health.

[308] Liu H., Chen Z., Xiang R., Liu Y. (2025). Independent and combined effects of volatile organic compounds on sarcopenia: Insights into environmental pollutants and muscle health. Ecotoxicol. Environ. Saf..

[309] Fang Y., Zhang J. (2024). The cumulative and single effect of 12 aldehydes concentrations on cardiovascular diseases: An analysis based on Bayesian kernel machine regression and weighted logistic regression. Rev. Cardiovasc. Med..

[310] Ge L., Liu J., Kang X., Wang W., Zhang D. (2024). Association of serum individual and mixed aldehydes with depressive symptoms in the general population: A machine learning study. J. Affect. Disord..

[311] Tang J., Chen Y., Xue P., Chen Y., Kong H., Lin C., Wang X., Liu S. (2024). Exposure to synthetic steroid hormones and precocious puberty in girls: A case-control study. Ecotoxicol. Environ. Saf..

[312] Xiao F., Wei Y., Zou P., Wu X. (2024). Associations between single and combined exposures to environmental phenols and ulcerative colitis in American adults. Clin. Res. Hepatol. Gastroenterol..

[313] Su H., Xi J., Miao M., Liang H., Chen Y., Wang Z., Zhou Y., Jin Y., Ji H., Yuan W. (2025). Bisphenol analogs exposure in 4-year-old children and their intelligence quotient at 6 years: A prospective cohort study. Environ. Res..

[314] Zhao Z., Zhang C., Li Y., Liu J., Wang L., Wang X., Wang Y., Liu M., Yue X., Wang X. (2025). Association between exposure to brominated flame retardants and atherosclerosis: Evidence for inflammatory status as a potential mediator. Sci. Total Environ..

[315] Tanner E.M., Hallerbäck M.U., Wikström S., Lindh C., Kiviranta H., Gennings C., Bornehag C.-G. (2020). Early prenatal exposure to suspected endocrine disruptor mixtures is associated with lower IQ at age seven. Environ. Int..

[316] Wu B., Jiang Y., Jin X., He L. (2020). Using three statistical methods to analyze the association between exposure to 9 compounds and obesity in children and adolescents: NHANES 2005–2010. Environ. Health.

[317] Hu J.M.Y., Arbuckle T.E., Janssen P., Lanphear B.P., Zhuang L.H., Braun J.M., Chen A., McCandless L.C. (2021). Prenatal exposure to endocrine disrupting chemical mixtures and infant birth weight: A Bayesian analysis using kernel machine regression. Environ. Res..

[318] Kim B., Park B., Kim C.H., Kim S., Park B. (2022). Association between endocrine-disrupting chemical mixture and metabolic indices among children, adolescents, and adults: A population-based study in Korea. Environ. Pollut..

[319] Midya V., Alcala C.S., Rechtman E., Gregory J.K., Kannan K., Hertz-Picciotto I., Teitelbaum S.L., Gennings C., Rosa M.J., Valvi D. (2023). Machine learning assisted discovery of interactions between pesticides, phthalates, phenols, and trace elements in child neurodevelopment. Environ. Sci. Technol..

[320] Jang H., Choi K.-H., Cho Y.M., Han D., Hong Y.S. (2024). Environmental risk score of multiple pollutants for kidney damage among residents in vulnerable areas by occupational chemical exposure in Korea. Environ. Sci. Pollut. Res. Int..

[321] Li R., Lin X., Lu T., Wang J., Wang Y., Xu L. (2024). Associations between exposure to multiple environmental chemicals and metabolic syndrome: A mixture analysis. Hyg. Environ. Health Adv..

[322] Midya V., Gennings C. (2024). Detecting shape-based interactions among environmental chemicals using an ensemble of exposure-mixture regression and interpretable machine learning tools. Stat. Biosci..

[323] Alampi J.D., Lanphear B.P., Macfarlane A.J., Braun J.M., Oulhote Y., Ashley-Martin J., Arbuckle T.E., Chen A., Muckle G., Mccandless L.C. (2025). Association between gestational environmental chemical mixtures and folate exposures with autistic behaviors in a Canadian birth cohort. Environ. Epidemiol..

[324] Guo L.-C., Zhu P., Gui C., Deng J., Gao Y., Long C., Zhang H., Lv Z., Yu S. (2025). Disrupting effects of neonicotinoids and their interaction with metals on thyroid hormone, an evidence of children in a rural area, South China. Ecotoxicol. Environ. Saf..

[325] Haruna I., Obeng-Gyasi E. (2025). Combined effect of per- and polyfluoroalkyl substances, toxic metals, and essential elements on chronic kidney disease. Pollutants.

[326] Huang S., Li Z., Wu C., Zhao X., Xiong J., Xiao W., Su H., Zheng R., Xu Z., Su Q. (2025). Divergent effects of EDCs on bone maturation: Role of body mass index and puberty. Ecotoxicol. Environ. Saf..

[327] Jehu-Appiah D., Obeng-Gyasi E. (2025). Combined Effect of metals, PFAS, phthalates, and plasticizers on cardiovascular disease risk. Toxics.

