# Supplementary material for: An Exploration of Machine Learning Methods in Human Biomonitoring
Source: Int J Environ Res Public Health. 2026 May 20;23(5):680. doi: 10.3390/ijerph23050680 (PMC13206933; doi:10.3390/ijerph23050680)
Supplement: Supplementary file 1 [file ijerph-23-00680-s001.zip › ijerph-4229690-supplementary.pdf]

An Exploration of Machine Learning Methods in Human Biomonitoring

Supplementary Materials

Search Strategy: Ovid MEDLINE(R) ALL 2

Search Strategy: Embase 10

Search Strategy: Global Health 18

Search Strategy: Scopus 25

International Scan Survey 28

Table S1. Categorization of ML Method 37

Studies by Biomonitoring Theme 40

- Table S2. Description – Chemicals 40
- Table S3. Description – Exposure Factors 40
- Table S5. Association/Prediction – Chemicals (from exposure factors) 42
- Table S6. Association/Prediction – Chemicals (from biomarkers of exposure) 44
- Table S7. Association/Prediction – Health-Based Outcomes (from individual or multiple chemicals) 45
- Table S8. Association/Prediction – Health-Based Outcomes (from chemical mixtures) 53

Results of International Scan Survey 85

Search Strategy: Ovid MEDLINE(R) ALL

1946 to August 04, 2025

#	Searches	Results
1	artificial intelligence/ or exp machine learning/ or Natural Language Processing/	154541
2	(artificial intelligen* or deep* learning or expert system? or comput* learning* or intelligen* automation* or intelligen* retrieval or knowledge engineering or machine learn* or natural language process* or Generative language* or machine* learning or cognitive comput* or automat* reason* or intelligen* automat* or synthentic* intelligen* or smart* machine*).tw,kw,kf.	288845
3	or/1-2 [Machine Learning]	326128
4	biological monitoring/ or biological marker/ or body burden/ or exp toxicity testing/ or exp toxicity assay/ or hair analysis/ or exp blood level/ or exp Breath Tests/ or exp blood analysis/ or plasma/ or exp urine level/ or exp urinalysis/ or exp feces analysis/ or exp feces level/ or exp milk level/ or saliva analysis/ or saliva level/ or serum/ or semen analysis/ or sputum/ or sweat/	491308
5	clinical chemistry/	6208
6	((plasma or blood or serum or feces or faeces or fecal or faecal or stool or urin* or hair or human milk or breast milk or placenta or semen or seminal fluid* or seminal plasma* or sweat or perspirat* or saliva* or sputum) adj3 (sampl* or test* or level* or analy* or concentration*)).tw,kf.	1567047
7	((exhal* or expir*) adj2 (breath or air)).tw,kf.	9354
8	(biomonitor* or bio monitor* or biological monitor*).tw,kw,kf.	16074
9	or/4-8 [Biomonitoring]	1936950
10	Environmental Exposure/	88924
11	(chemical* or agrochemical* or pollut* or contamina* or leachate* or runoff* or spray drift or toxic* or poison* or neurotox* or embryotox* or cytotox* or hepatotox* or fetotox* or genotox* or maternotox* or immunotox* or dermatotox* or cardiotoxic* or nephrotoxic* or ototox*).tw,kf,kw.	2724054
12	Metals/ or ((Plasma or blood or serum or feces or faeces or fecal or faecal or serum or stool or urin* or hair or human milk or breast milk or placenta or semen or seminal fluid* or	111197

	plasma* or sweat or perspirat* or saliva* or sputumtest* or level* or analy* or concentration* or expos* or biomonitor* or biological monitor*) adj4 metal*).tw,kw.	
13	Mercury/ or (mercury or Hydrargyrum or mercure or Mercurio or Quecksilber or Quicksilver).tw,kw,kf.	56940
14	Lead/ or (((lead or leaded) and metal?) or pb or plumbum or olow).tw,kw,kf.	125855
15	Cadmium/ or (cadmium or kadmium).tw,kw,kf.	67587
16	Arsenic/ or arsenic*.tw,kw,kf.	44960
17	exp Pesticides/	203132
18	(avicid* or biocid* or biopesticid* or chemosteril* or fumigant* or fungicid* or herbicid* or insecticid* or larvicid* or molluscacid* or nematicid* or pesticid* or piscicid* or rodenticid*).tw,kw,kf.	174464
19	Flame Retardants/	6101
20	((organophosph* or OER? or flame?) adj5 retardant*).tw,kf.	9326
21	OPFR?.tw,kw,kf.	711
22	("bis(2-chloroethyl) 2-hydroxyethyl phosphate" or "Bis(2-chloroethyl) hydrogen phosphate" or "bis-(2-Chloroethyl) hydrogen phosphate" or "bis(2-chloroethyl) phosphate" or "Bis-(2-chloroethyl) phosphate" or "Di-beta,beta'-Chloroethylphosphoric Acid" or "Ethanol, 2-chloro-, hydrogen phosphate" or "Ethanol, 2-chloro-,1,1'-(hydrogen phosphate)").tw,kw,kf.	115
23	("Bis-(2-chloroisopropyl) phosphate" or "2-Propanol, 1,3-dichloro-, hydrogen phosphate" or "bis(1,3-dichloro-2-propyl) hydrogen phosphate" or "Bis(1,3-dichloro-2-propyl) phosphate" or "Bis-(1,3-dichloro-2-propyl) phosphate" or "bis(1,3-dichloropropan-2-yl) hydrogen phosphate" or "Phosphoric acid bis(1,3-dichloropropane-2-yl) ester").tw,kw,kf.	151
24	("(PhO)2(HO)PO" or "diphenoxyphosphinic acid" or "Diphenyl hydrogen phosphate" or "Diphenyl phosphate" or "diphenylphosphoric acid" or "Phenyl hydrogen phosphate" or "Phenyl phosphate" or "Phosphoric acid diphenyl" or "Phosphoric acid diphenyl ester").tw,kw,kf.	966
25	"1-Hydroxyl-2-propyl bis(1-chloro-2-propyl) phosphate".tw,kw,kf.	2
26	("Bis(1-chloro-2-isopropyl) 1-hydroxy-2-isopropyl phosphate" or "Isopropylphenyl phenyl phosphate" or "Diphenyl isopropylphenyl phosphate" or "diphenyl propan-2-yl phosphate" or "Isopropyl diphenyl phosphate" or "Phosphoric acid isopropyldiphenyl ester" or "Tetra-brominated benzoic acid" or "3-Bromobenzenecarboxylic acid" or "3-	24

	BROMOBENZOIC ACID" or "3-Bromobenzoic acid" or "3-Bromobenzoic acid" or "3-Bromo-benzoic acid" or "3-Bromobenzoicacid" or "3-bromophenyl carboxylic acid" or "5-bromobenzoic acid" or "Benzoic acid, 3-bromo-" or "Benzoic acid, m-bromo-" or "m-Bromobenzoic acid" or "meta bromobenzoic acid" or "Phosphoric acid tert-butyldiphenyl ester" or "Phosphoric acid, 1,1-dimethylethyl diphenyl ester" or "t-butyl diphenyl phosphate" or "t-butyl phenyl phenyl phosphate" or "tert-butyl diphenyl phosphate").tw,kw,kf.	
27	(BFR and (bromin* or flame*)).tw,kw,kf.	428
28	("Tetrabromobisphenol A" or "3,3',5,5'-Tetrabromobisphenol A" or "4,4'-(propane-2,2-diyl)bis(2,6-dibromophenol)" or "Bromdian" or "4,4'-Isopropylidenebis(2,6-dibromophenol)" or "2,2-Bis(3,5-dibromo-4-hydroxyphenyl)propane" or "TBBPA" or "Firemaster BP 4A" or "Tetrabromodian" or "Fire Guard 2000" or "Great Lakes BA-59P" or "Saytex RB 100PC" or "Tetrabromodiphenylopropane" or "Firemaster BP4A" or "2,2',6,6'-TETRABROMOBISPHENOL A" or "2,6-dibromo-4-[2-(3,5-dibromo-4-hydroxyphenyl)propan-2-yl]phenol" or "FG 2000" or "Phenol, 4,4'-(1-methylethylidene)bis[2,6-dibromo-" or "4,4'-(1-Methylethylidene)bis(2,6-dibromophenol)" or "3,5,3',5'-Tetrabromobisphenol A" or "NSC 59775" or "4,4'-propane-2,2-diylbis(2,6-dibromophenol)" or "Saytex RB-100" or "4,4'-Isopropylylidenebis(2,6-dibromophenol)" or "2,2-Bis(4-hydroxy-3,5-dibromophenyl)propane" or "FLAME CUT 120G" or "2,2',6,6'-Tetrabromo-4,4'-isopropylidenediphenol" or "Phenol, 4,4'-isopropylidenebis(2,6-dibromo-" or "Phenol, 4,4'-isopropylidenebis[2,6-dibromo-" or "4,4'-(2,2-propanediyl) bis[2,6-dibromo]phenol" or "Phenol, 4,4'-(1-methylethylidene)bis(2,6-dibromo-" or "TETRABROMO-4,4'-ISOPROPYLIDENEDIPHENOL" or "Tetrabromobisphenol A 50 microg/mL in Methanol" or "2,2,6,6-Tetrabromo-4,4-Isopropylidene Phenol" or "4,4'-(1-Methylethylidene)bis(2,6-dibromophenol)2,2-bis(3,5-dibromo-4-hydroxyphenyl)propane" or "2,6-dibromo-4-[1-(3,5-dibromo-4-hydroxyphenyl)-1-methylethyl]phenol" or "Tetrabromo bisphenol A" or "4,4'-(1-methylethylidene)bis[2,6-dibromophenol]" or "3,3',5,5'-Tetrabromo bisphenol A" or "4,4'-(2,2-PROPANEDIYL)BIS(2,6-DIBROMOPHENOL)" or "4,6-dibromophenol)" or "Tetrabromobis phenol A" or "TBBP-A" or "Saytex RB-100 ABS" or "2,5-dibromophenyl)propane" or "bmse000567" or "Tetrabromobisphenol ''A''" or "2,6,6'-Tetrabromobisphenol A" or "33'55'-Tetrabromobisphenol A" or "Tetrabromobisphenol A (TBBP A)" or "3,3',5'-Tetrabromobisphenol A" or "2,5-dibromo-4-hydroxyphenyl)propane" or "2,6-dibromo-4-[1-(3,5-dibromo-4-hydroxy-phenyl)-1-methyl-ethyl]phenol" or "2,2'',6,6''-Tetrabromobisphenol A" or "3,3'',5,5''-tetrabromobisphenol A" or "Phenol,4'-isopropylidenebis[2,6-dibromo-" or "phenol, 4,4'-isopropylidenebis (dibromo-)" or "2,2-bis(3,5dibromo-4-hydroxyphenyl)propane" or "4,4''-Isopropylidenebis(2,6-dibromophenol)" or "4,4'-isopropylidene-bis(2,6-dibromophenol)" or "2,2-bis(3,5-dibromo-4-hydroxyphenyl)-propane" or "2,2-bis-(3,5-dibromo-4-hydroxyphenyl)propane" or "2,2-	1497

	bis-(3,5-dibromo-4-hydroxyphenyl)-propane" or "2,2',6,6'-tetrabrom-4,4'-isopropylidendiphenol" or "2,2',6,6'-TETRABROMOBISPHENOL A [HSDB]" or "Fenol, 4,4'-(1-metiletilideno)bis[2,6-dibromo-" or "Phenol,4'-(1-methylethylidene)bis[2,6-dibromo-" or "Tetrabromodian: tetrabromodihydroxy diphenylpropane" or "4,4''-(1-Methylethylidene)bis(2,6-dibromophenol)" or "4,4''-(2,2-propanediyl) bis[2,6-dibromo]phenol" or "4,4''-(propane-2,2-diyl)bis(2,6-dibromophenol)" or "2,2',6,6'-Tetrabromo-4,4'-isopropylidene bisphenol" or "2,2-bis-(4'-hydroxy-3',5'-dibromophenyl)-propane" or "3,3',5,5'-Tetrabromobisphenol A, analytical standard" or "3,3',5,5'-Tetrabromo-4,4-dihydroxy-2,2-diphenylpropane" or "Phenol, 4,4'-(1-methylethylidene)bis-, tetrabromo deriv." or "3,3',5,5'-Tetrabromo-4,4'-dihydroxy-diphenyl-dimethyl-methane" or "3,3',5,5'-Tetrabromobisphenol A, certified reference material, TraceCERT(R)").tw,kw,kf.	
29	("2,2',4,4',5,5'-hexabromobiphenyl" or "2,4,5,2',4',5'-Hexabromobiphenyl" or "Firemaster FF-1" or "PBB" or "Polybrominated biphenyl" or "brominated biphenyl").tw,kw,kf.	1439
30	Persistent Organic Pollutants/	652
31	(Persistent Organic Pollutants or POP or POPs).tw,kw,kf.	19462
32	dioxin/ or exp furan derivative/ or exp dibenzofuran derivative/ or (Octachlorodibenzofuran or "F 135" or Octapolychlorinated dibenzofuran or Perchlorodibenzofuran).tw,kw.	6594
33	polychlorinated biphenyl/ or ("1,1'-Biphenyl, chloro derivs" or Aroclor or Biphenyl chlorinated or Biphenyl polychloro- or Chlophen or Chlorextol or Chlorinated biphenyl or Chlorinated diphenyl or Chlorinated diphenylene or "Chloro 1,1-biphenyl" or Chloro biphenyl or Clophen or Dykanol or Fenclor or Fenclor 42 or Inerteen or Kanechlor or Montar or Monter or Noflamol or PCB or PCBS or Phenochlor or Phenoclor or Polychlorinated biphenyl or Polychlorinated biphenyls or Polychlorobiphenyl or Polychlorobiphenyls or Pyralene or Pyranol or Santotherm or Sovol or Therminol).tw,kw.	28100
34	PCB*.tw,kw.	25137
35	Fluorocarbons/	17567
36	(perfluor* or polyfluor*).tw,kw,kf.	26634
37	(PFAS? or ((perfluorinated or per-fluorinated or polyfluorinated or poly fluorinated) adj3 alkyl substance*) or ((perfluoroalkyl* or per-fluoroalkyl* or polyfluoroalkyl* or poly-fluoroalkyl* or per-fluoro alkyl) adj3 substance*)).tw,kf.	8610
38	(PFAA? or ((perfluoroalkyl or per fluoroalkyl or per-fluoro alkyl) adj3 acid*)).tw,kw.	1847
39	(PFBA or ((perfluorobutanoic or perfluorobutyric) adj3 acid*) or heptafluoro butyric acid or Heptafluoro-1-butanoic acid or Heptafluorobutanoic acid or heptafluoro-butanoic acid	1037

	or heptafluoro-butanoic acid or Heptafluorobutyric acid or heptafluoro-butyric acid or Heptafluorobutyricacid or Heptafluoro-n-butyric acid or heptaflurorbutyric acid or Kyselina heptafluormaselna or Perfluorobutanoic acid or Perfluorobutyric acid or Perfluoropropanecarboxylic acid).tw,kw.	
40	(PFPeA or perfluoropentanoate or ((perfluoropentanoic or perfluoro-n-pentanoic) adj3 acid) or Nonafluoro-1-pentanoic acid or nonafluoropentanoic acid or Nonafluorovaleric acid or nonafluoro-Valeric acid or Nonafluoro-valeric acid or n-Perfluoropentanoic acid or Pentanoic acid, nonafluoro- or Perfluorovaleric acid).tw,kf.	374
41	(PFHxA or ACMC-1CLF8 or IPC-PFFA-6 or Perfluorocaproic acid or Perfluorohexanoic acid or perfluoro-Hexanoic acid or Undecafluoro-1-hexanoic acid or Undecafluorohexanoic acid).tw,kf.	653
42	(PFNA or Heptadecafluornonansaeure or heptadecafluorononanoic acid or heptadecafluoro-nonanoic acid or Heptadecafluoropelargonic Acid or Heptadekafluornonansaeure or n-Heptadecafluorononanoic acid or Perfluornonansaeure or Perfluoro-n-nonanoic acid or Perfluorononan-1-oic acid or Perfluorononanoic acid or Perfluorononanoicacid or Perfluoropelargonic Acid).tw,kf.	2172
43	(PFDA or Ndfda or Nonadecafluorocapric acid or Nonadecafluorodecanoic acid or Nonadecafluoro-n-decanoic acid or Perfluorocapric acid or Perfluorodecanoic acid or Perfluoro-N-decanoic acid).tw,kf.	1084
44	(PFDoA or n-perfluorododecanoic acid or Perfluorododecanoic acid or Perfluorolauric acid or Tricosafluorododecanoic acid or tricosafluoro-dodecanoic acid or Tricosafluorolauric Acid).tw,kf.	253
45	(PFBS or 1-Perfluorobutanesulfonic acid or Nonafluoro-1-butanesulfonic acid or nonafluoro-1-Butanesulfonic acid or Nonafluoro-1-butanesulfonicAcid or nonafluorobutane sulfonic acid or Nonafluorobutane-1-sulfonic acid or Nonafluorobutanesulfonic acid or nonafluoro-butanesulfonic acid or nonafluorobutanesulphonic acid or Pentyl perfluorobutanoate or Perfluoro-1-butanesulfonic Acid or perfluorobutane sulfonic acid or perfluorobutane-1-sulfonic acid or perfluorobutanesulfonic acid or perfluorobutanesulphonic acid or perfluorobutyl sulfonic acid or PFBuS).tw,kf.	836
46	(PFHxS or perfluorohexane sulfonic acid or Perfluorohexane-1-sulfonic acid or Perfluorohexane-1-sulphonic acid or Perfluorohexanesulfonic acid or perfluorohexanesulphonic acid or tridecafluorohexane-1-sulfonic acid or Perfluorohexanesulfate).tw,kf.	1613

47	("Gen X" or HFPO-DA or "ammonium 2,3,3,3-tetrafluoro-2-(heptafluoropropoxy)propanoate" or FRD-902 or " heptafluoropropyl 1,2,2,2-tetrafluoroethyl ether").tw,kf.	322
48	(Bisphenol? or BPA or BPE or BPS or BPF or BPAF).tw,kw,kf.	33392
49	("2,2-(4,4'-Dihydroxydiphenyl)propane" or "2,2-Bis(4-hydroxyphenyl)propane" or "2,2-Bis(hydroxyphenyl)propane" or "2,2-Bis(p-hydroxyphenyl)propane" or "2,2-Bis-4'-hydroxyfenylpropan" or "2,2-Di(4-hydroxyphenyl)propane" or "2,2-Di(4-phenylol)propane" or "4,4'-(1-Methylethylidene)bisphenol" or "4,4'-Bisphenol A" or "4,4'-Dihydroxydiphenyl-2,2-propane" or "4,4'-Dihydroxydiphenyldimethylmethane" or "4,4'-Dihydroxydiphenylpropane" or "4,4'-Isopropylidene diphenol" or "4,4'-Isopropylidenebisphenol" or "4,4'-Isopropylidenediphenol" or "beta,beta'-Bis(p-hydroxyphenyl)propane" or "beta-Di-p-hydroxyphenylpropane" or "Biphenol A" or "Bis(4-hydroxyphenyl) dimethylmethane" or "Bis(4-hydroxyphenyl)dimethylmethane" or "Bis(4-hydroxyphenyl)propane" or "Bisferol A" or "Bisphenol" or "Bisphenol A" or "Bisphenol-A" or "BPA" or "Dimethyl bis(p-hydroxyphenyl)methane" or "Dimethylbis(p-hydroxyphenyl)methane" or "Dimethylmethylene-p,p'-diphenol" or "Diphenylolpropane" or "Ipognox 88" or "Isopropylidenebis(4-hydroxybenzene)" or "p,p'-Bisphenol A" or "p,p'-Dihydroxydiphenyldimethylmethane" or "p,p'-Dihydroxydiphenylpropane" or "p,p'-Isopropylidenebisphenol" or "p,p'-Isopropylidenediphenol" or "Parabis A" or "Phenol, (1-methylethylidene)bis-" or "Phenol, 4,4'-(1-methylethylidene)bis-" or "Phenol, 4,4'-dimethylmethylenedi-" or "Phenol, 4,4'-isopropylidenedi-" or "Pluracol 245" or "Propane, 2,2-bis(p-hydroxyphenyl)-" or "Rikabanol").tw,kw,kf.	20706
50	("2,2-(4,4'-Dihydroxydiphenyl)propane" or "2,2-Bis(4-hydroxyphenyl)propane" or "2,2-Bis(hydroxyphenyl)propane" or "2,2-Bis(p-hydroxyphenyl)propane" or "2,2-Bis-4'-hydroxyfenylpropan" or "2,2-Di(4-hydroxyphenyl)propane" or "2,2-Di(4-phenylol)propane" or "4,4'-(1-Methylethylidene)bisphenol" or "4,4'-Bisphenol A" or "4,4'-Dihydroxydiphenyl-2,2-propane" or "4,4'-Dihydroxydiphenyldimethylmethane" or "4,4'-Dihydroxydiphenylpropane" or "4,4'-Isopropylidene diphenol" or "4,4'-Isopropylidenebisphenol" or "4,4'-Isopropylidenediphenol" or "beta,beta'-Bis(p-hydroxyphenyl)propane" or "beta-Di-p-hydroxyphenylpropane" or "Biphenol A" or "Bis(4-hydroxyphenyl) dimethylmethane" or "Bis(4-hydroxyphenyl)dimethylmethane" or "Bis(4-hydroxyphenyl)propane" or "Bisferol A" or "Dimethyl bis(p-hydroxyphenyl)methane" or "Dimethylbis(p-hydroxyphenyl)methane" or "Dimethylmethylene-p,p'-diphenol" or "Diphenylolpropane" or "Ipognox 88" or "Isopropylidenebis(4-hydroxybenzene)" or "p,p'-Dihydroxydiphenyldimethylmethane" or "p,p'-Dihydroxydiphenylpropane" or "p,p'-Isopropylidenebisphenol" or "p,p'-Isopropylidenediphenol" or "Parabis A" or "Phenol, (1-methylethylidene)bis-" or "Phenol, 4,4'-(1-methylethylidene)bis-" or "Phenol, 4,4'-	180

	dimethylmethylenedi-" or "Phenol, 4,4'-isopropylidenedi-" or "Pluracol 245" or "Propane, 2,2-bis(p-hydroxyphenyl)-" or "Rikabanol").tw,kw,kf.	
51	("1,1-bis(4-hydroxyphenyl) ethane" or "1,1-Bis(4-hydroxyphenyl)ethane" or "4,4'-(ETHANE-1,1-DIYL)DIPHENOL" or "4,4'-ethane-1,1-diyldiphenol" or "4,4'-Ethylidenebisphenol" or "4,4'-Ethylidenediphenol" or "4-[1-(4-hydroxyphenyl)ethyl]phenol" or "Bisphenol E" or "Phenol, 4,4'-ethylidenebis-").tw,kw,kf.	112
52	("1,1'-Sulfonylbis(4-hydroxybenzene)" or "1,1'-Sulfonylbis[4-hydroxybenzene]" or "4-(4-hydroxybenzenesulfonyl)phenol" or "4-(4-hydroxyphenyl)sulfonylphenol" or "4-(4-hydroxyphenylsulfonyl)phenol" or "4,4'-sulfonyldiphenol" or "4,4'-Bisphenol S" or "4,4'-dihydroxy diphenyl sulfone" or "4,4'-dihydroxy diphenyl sulphone" or "4,4'-dihydroxy diphenylsulfone" or "4,4'-Dihydroxydiphenyl sulfone" or "4,4'-Dihydroxydiphenyl sulfone (4,4'-DHDPS)" or "4,4'-Dihydroxydiphenyl sulfone" or "4,4'-Dihydroxydiphenyl sulphone" or "4,4'-Dihydroxydiphenyl sulphone (4,4'-DHDPS)" or "4,4'-dihydroxydiphenylsulfone" or "4,4'-Dihydroxydiphenylsulphone" or "4,4'-Sulfonylbisphenol" or "4,4'-Sulfonyldiphenol" or "4,4'-Sulfonyldiphenol (4,4'-Dihydroxydiphenylsulfone)" or "4,4'-Sulphonyldiphenol" or "4-[(4-hydroxyphenyl)sulfonyl]phenol" or "4-Hydroxyphenyl sulfone" or "bis(4-hydroxylphenyl) sulfone" or "Bis(4-hydroxyphenyl) sulfone" or "Bis(4-hydroxyphenyl)sulfone" or "Bis(p-hydroxyphenyl) sulfone" or "Bis(p-hydroxyphenyl)sulfone" or "di-(4-Hydroxyphenyl)sulfone" or "Diphone C" or "P,P'-Dihydroxydiphenyl sulfone" or "Phenol, 4,4'-sulfonylbis-" or "Phenol,4'-sulfonylbis-").tw,kw,kf.	91
53	("4-(4-hydroxybenzyl)phenol" or "4,4'-Bis(hydroxyphenyl)methane" or "4,4'-bisphenol F" or "4,4''-Dihydroxydiphenylmethane" or "4,4'-Dihydroxydiphenylmethane" or "4,4-Dihydroxydiphenylmethane" or "4,4'-methanediyl-di-phenol" or "4,4'-Methylene diphenol" or "4,4'-Methylenebis(phenol)" or "4,4'-Methylenebisphenol" or "4,4'-methylenebis-phenol" or "4,4'-Methylenediphenol" or "4,4-Methylenediphenol" or "4-[(4-hydroxyphenyl)methyl]phenol" or "Bis(4-hydroxyphenyl)methane" or "Bis-(4-hydroxyphenyl)methane" or "bis(para-hydroxyphenyl)methane" or "bis-(p-hydroxyphenyl)methane" or "bis-(p-hydroxyphenyl)-methane" or "methyl2-methoxypropionate" or "p-(p-Hydroxybenzyl)phenol" or "p,p'-Bis(hydroxyphenyl)methane" or "p,p'-BPF" or "p,p'-Methylenediphenol" or "para-(para-hydroxybenzyl)phenol" or "Phenol, 4,4'-methylenebis-" or "Phenol, 4,4'-methylenedi-" or "Phenol,4'-methylenebis-").tw,kw,kf.	49
54	Triclosan/	3595
55	("2' hydroxy 2,4,4' trichlorodiphenyl ether" or "2-(2,4-dichlorophenoxy)-5-chlorophenol" or "2,3',4 trichloro 2' hydroxydiphenyl ether" or "2,4,4' trichloro 2' hydroxydiphenyl ether" or "2,4,4'-Trichloro-2'-hydroxy diphenyl ether" or "2,4,4'-Trichloro-2'-hydroxydiphenyl ether"	4924

	or "2,4,4'-trichloro-2'-hydroxydiphenylethe" or "2-[2,4-bis(chloranyl)phenoxy]-5-chloranyl-phenol" or "5 chloro 2 (2,4 dichlorophenoxy)phenol" or "5-chloro-2-(2, 4-dichlorophenoxy)phenol" or "5-Chloro-2-(2,4-dichloro-phenoxy)-phenol" or "5-chloro-2-(2,4-di-chloro-phenoxy)-phenol" or "aquasept" or "cgp 433" or "cgp433" or "ch 3565" or "ch3565" or "cloxifenol" or "Cloxifenolum" or "dp 300" or "dp300" or "DP-300" or "Ether, 2'-hydroxy-2,4,4'-trichlorodiphenyl" or "Irgasan" or "Lexol 300" or "manusept" or "Neostrata" or "novaderm" or "Phenol, 5-chloro-2-(2,4-dichlorophenoxy)-" or "Phenyl ether, 2'-hydroxy-2,4,4'-trichloro-" or "Sapoderm" or "Sterzac" or "Stri-Dex Cleansing Bar" or "Stri-Dex Face Wash" or "Trichlorosan" or "triclosan" or "Triclosanum").tw,kw,kf.	
56	or/10-55 [Environmental and chemical exposures]	3196835
57	("German Environmental Survey" or "GerES" or "Canadian Health Measures Survey" or "CHMS" or "National human biomonitoring programme in France" or "Human Biomonitoring Programme for Ireland" or "HBM4IRE" or "Korea National Health and Nutrition Examination Survey" or "KNHANES" or "China National Human Biomonitoring" or "CNHBM" or "Human biomonitoring for Europe" or "European Human Biomonitoring Initiative" or HBM4EU or "Partnership for the Assessment of Risks in Chemicals" or "Demonstration of a study to Coordinate and Perform Human Biomonitoring on a European Scale" or DEMOCOPHES or "Consortium to Perform Human Biomonitoring on a European Scale" or COPHES or "Human Biomonitoring in Europe" or EUHBM or National Biomonitoring Program* or "National Health and Nutrition Examination Survey" or NHANES).tw,kw,kf.	31700
58	3 and 9 and 56	802
59	3 and 57	578
60	or/58-59	1336

Search Strategy: Embase

1974 to 2025 August 01

#	**Searches**	**Results**
1	artificial intelligence/ or machine learning/	260032
2	(artificial intelligen* or AIVI or "convolutional network*" or DALL-E or "decision support system*" or deep* learning or expert system? or Deep AI or comput* learning* or intelligen* automation* or intelligen* retrieval or knowledge engineering or machine learn* or natural language process* or Generative language* or "language learning model*" or "large language model*" or machine* learning or cognitive comput* or automat* reason* or intelligen* automat* or synthentic* intelligen* or smart* machine* or ChatGPT* or "Chat GPT" or "Google* Bard" or "Google* Gemini" or "IBM Watson" or "Microsoft* Copilot" or OpenAI or "Open AI" or PathAI or "Path AI").tw,kw,kf.	352543
3	or/1-2 [Machine Learning]	407705
4	biological monitoring/ or biological marker/ or body burden/ or exp toxicity testing/ or exp toxicity assay/ or hair analysis/ or exp blood level/ or exp Breath Tests/ or exp blood analysis/ or plasma/ or exp urine level/ or exp urinalysis/ or exp feces analysis/ or exp feces level/ or exp milk level/ or saliva analysis/ or saliva level/ or serum/ or semen analysis/ or sputum/ or sweat/	3628212
5	clinical chemistry/	20479
6	((plasma or blood or serum or feces or faeces or fecal or faecal or stool or urin* or hair or human milk or breast milk or placenta or semen or seminal fluid* or seminal plasma* or sweat or perspirat* or saliva* or sputum) adj3 (sampl* or test* or level* or analy* or concentration*)).tw,kf.	2247629
7	((exhal* or expir*) adj2 (breath or air)).tw,kf.	13522
8	(biomonitor* or bio monitor* or biological monitor*).tw,kw,kf.	20260
9	or/4-8 [Biomonitoring]	4653522
10	Environmental Exposure/	134203
11	(chemical* or agrochemical* or pollut* or contamina* or leachate* or runoff* or spray drift or toxic* or poison* or neurotox* or embryotox* or cytotox* or hepatotox* or fetotox* or genotox* or maternotox* or immunotox* or dermatotox* or cardiotoxic* or nephrotoxic* or ototox*).ti,kf. or (chemical* or agrochemical* or pollut* or contamina* or leachate* or runoff* or spray drift or toxic* or poison* or neurotox* or embryotox* or cytotox* or	1786697

	hepatotox* or fetotox* or genotox* or maternotox* or immunotox* or dermatotox* or cardiotoxic* or nephrotoxic* or ototox*).ab. /freq=2	
12	Metals/ or ((Plasma or blood or serum or feces or faeces or fecal or faecal or serum or stool or urin* or hair or human milk or breast milk or placenta or semen or seminal fluid* or plasma* or sweat or perspirat* or saliva* or sputumtest* or level* or analy* or concentration* or expos* or biomonitor* or biological monitor*) adj4 metal*).tw,kw.	134206
13	Mercury/ or (mercury or Hydrargyrum or mercure or Mercurio or Quecksilber or Quicksilver).tw,kw,kf.	74025
14	Lead/ or (((lead or leaded or pb) and metal?) or plumbum or olow).tw,kw,kf.	126664
15	Cadmium/ or (cadmium or kadmium).tw,kw,kf.	107810
16	Arsenic/ or arsenic*.tw,kw,kf.	63362
17	exp Pesticides/	461437
18	(avicid* or biocid* or biopesticid* or chemosteril* or fumigant* or fungicid* or herbicid* or insecticid* or larvicid* or molluscacid* or nematicid* or pesticid* or piscicid* or rodenticid*).tw,kw,kf.	191528
19	Flame Retardants/	7792
20	((organophosph* or OER? or flame?) adj5 retardant*).tw,kf.	9169
21	OPFR?.tw,kw,kf.	717
22	("bis(2-chloroethyl) 2-hydroxyethyl phosphate" or "Bis(2-chloroethyl) hydrogen phosphate" or "bis-(2-Chloroethyl) hydrogen phosphate" or "bis(2-chloroethyl) phosphate" or "Bis-(2-chloroethyl) phosphate" or "Di-beta,beta'-Chloroethylphosphoric Acid" or "Ethanol, 2-chloro-, hydrogen phosphate" or "Ethanol, 2-chloro-,1,1'-(hydrogen phosphate)").tw,kw,kf.	117
23	("Bis-(2-chloroisopropyl) phosphate" or "2-Propanol, 1,3-dichloro-, hydrogen phosphate" or "bis(1,3-dichloro-2-propyl) hydrogen phosphate" or "Bis(1,3-dichloro-2-propyl) phosphate" or "Bis-(1,3-dichloro-2-propyl) phosphate" or "bis(1,3-dichloropropan-2-yl) hydrogen phosphate" or "Phosphoric acid bis(1,3-dichloropropane-2-yl) ester").tw,kw,kf.	158
24	("(PhO)2(HO)PO" or "diphenoxyphosphinic acid" or "Diphenyl hydrogen phosphate" or "Diphenyl phosphate" or "diphenylphosphoric acid" or "Phenyl hydrogen phosphate" or "Phenyl phosphate" or "Phosphoric acid diphenyl" or "Phosphoric acid diphenyl ester").tw,kw,kf.	973

25	"1-Hydroxyl-2-propyl bis(1-chloro-2-propyl) phosphate".tw,kw,kf.	2
26	("Bis(1-chloro-2-isopropyl) 1-hydroxy-2-isopropyl phosphate" or "Isopropylphenyl phenyl phosphate" or "Diphenyl isopropylphenyl phosphate" or "diphenyl propan-2-yl phosphate" or "Isopropyl diphenyl phosphate" or "Phosphoric acid isopropyldiphenyl ester" or "Tetra-brominated benzoic acid" or "3-Bromobenzenecarboxylic acid" or "3-BROMOBENZOIC ACID" or "3-Bromobenzoic acid" or "3-Bromobenzoic acid" or "3-Bromo-benzoic acid" or "3-Bromobenzoicacid" or "3-bromophenyl carboxylic acid" or "5-bromobenzoic acid" or "Benzoic acid, 3-bromo-" or "Benzoic acid, m-bromo-" or "m-Bromobenzoic acid" or "meta bromobenzoic acid" or "Phosphoric acid tert-butyldiphenyl ester" or "Phosphoric acid, 1,1-dimethylethyl diphenyl ester" or "t-butyl diphenyl phosphate" or "t-butyl phenyl phenyl phosphate" or "tert-butyl diphenyl phosphate").tw,kw,kf.	24
27	(BFR and (bromin* or flame*)).tw,kw,kf.	484
28	("Tetrabromobisphenol A" or "3,3',5,5'-Tetrabromobisphenol A" or "4,4'-(propane-2,2-diyl)bis(2,6-dibromophenol)" or "Bromdian" or "4,4'-Isopropylidenebis(2,6-dibromophenol)" or "2,2-Bis(3,5-dibromo-4-hydroxyphenyl)propane" or "TBBPA" or "Firemaster BP 4A" or "Tetrabromodian" or "Fire Guard 2000" or "Great Lakes BA-59P" or "Saytex RB 100PC" or "Tetrabromodiphenylopropane" or "Firemaster BP4A" or "2,2',6,6'-TETRABROMOBISPHENOL A" or "2,6-dibromo-4-[2-(3,5-dibromo-4-hydroxyphenyl)propan-2-yl]phenol" or "FG 2000" or "Phenol, 4,4'-(1-methylethylidene)bis[2,6-dibromo-" or "4,4'-(1-Methylethylidene)bis(2,6-dibromophenol)" or "3,5,3',5'-Tetrabromobisphenol A" or "NSC 59775" or "4,4'-propane-2,2-diylbis(2,6-dibromophenol)" or "Saytex RB-100" or "4,4'-Isopropylylidenebis(2,6-dibromophenol)" or "2,2-Bis(4-hydroxy-3,5-dibromophenyl)propane" or "FLAME CUT 120G" or "2,2',6,6'-Tetrabromo-4,4'-isopropylidenediphenol" or "Phenol, 4,4'-isopropylidenebis(2,6-dibromo-" or "Phenol, 4,4'-isopropylidenebis[2,6-dibromo-" or "4,4'-(2,2-propanediyl) bis[2,6-dibromo]phenol" or "Phenol, 4,4'-(1-methylethylidene)bis(2,6-dibromo-" or "TETRABROMO-4,4'-ISOPROPYLIDENEDIPHENOL" or "Tetrabromobisphenol A 50 microg/mL in Methanol" or "2,2,6,6-Tetrabromo-4,4-Isopropylidene Phenol" or "4,4'-(1-Methylethylidene)bis(2,6-dibromophenol)2,2-bis(3,5-dibromo-4-hydroxyphenyl)propane" or "2,6-dibromo-4-[1-(3,5-dibromo-4-hydroxyphenyl)-1-methylethyl]phenol" or "Tetrabromo bisphenol A" or "4,4'-(1-methylethylidene)bis[2,6-dibromophenol]" or "3,3',5,5'-Tetrabromo bisphenol A" or "4,4'-(2,2-PROPANEDIYL)BIS(2,6-DIBROMOPHENOL)" or "4,6-dibromophenol)" or "Tetrabromobis phenol A" or "TBBP-A" or "Saytex RB-100 ABS" or "2,5-dibromophenyl)propane" or "bmse000567" or "Tetrabromobisphenol ''A''" or "2,6,6'-Tetrabromobisphenol A" or "33'55'-Tetrabromobisphenol A" or "Tetrabromobisphenol A (TBBP A)" or "3,3',5'-Tetrabromobisphenol A" or "2,5-dibromo-4-hydroxyphenyl)propane" or "2,6-dibromo-4-[1-(3,5-dibromo-4-hydroxy-phenyl)-1-methyl-ethyl]phenol" or "2,2'',6,6''-	1557

	Tetrabromobisphenol A" or "3,3'',5,5''-tetrabromobisphenol A" or "Phenol,4'-isopropylidenebis[2,6-dibromo-" or "phenol, 4,4'-isopropylidenebis (dibromo-)" or "2,2-bis(3,5dibromo-4-hydroxyphenyl)propane" or "4,4''-Isopropylidenebis(2,6-dibromophenol)" or "4,4'-isopropylidene-bis(2,6-dibromophenol)" or "2,2-bis(3,5-dibromo-4-hydroxyphenyl)-propane" or "2,2-bis-(3,5-dibromo-4-hydroxyphenyl)propane" or "2,2-bis-(3,5-dibromo-4-hydroxyphenyl)-propane" or "2,2',6,6'-tetrabrom-4,4'-isopropylidendiphenol" or "2,2',6,6'-TETRABROMOBISPHENOL A [HSDB]" or "Fenol, 4,4'-(1-metiletilideno)bis[2,6-dibromo-" or "Phenol,4'-(1-methylethylidene)bis[2,6-dibromo-" or "Tetrabromodian: tetrabromodihydroxy diphenylpropane" or "4,4''-(1-Methylethylidene)bis(2,6-dibromophenol)" or "4,4''-(2,2-propanediyl) bis[2,6-dibromo]phenol" or "4,4''-(propane-2,2-diyl)bis(2,6-dibromophenol)" or "2,2',6,6'-Tetrabromo-4,4'-isopropylidene bisphenol" or "2,2-bis-(4'-hydroxy-3',5'-dibromophenyl)-propane" or "3,3',5,5'-Tetrabromobisphenol A, analytical standard" or "3,3',5,5'-Tetrabromo-4,4-dihydroxy-2,2-diphenylpropane" or "Phenol, 4,4'-(1-methylethylidene)bis-, tetrabromo deriv." or "3,3',5,5'-Tetrabromo-4,4'-dihydroxy-diphenyl-dimethyl-methane" or "3,3',5,5'-Tetrabromobisphenol A, certified reference material, TraceCERT(R)").tw,kw,kf.	
29	("2,2',4,4',5,5'-hexabromobiphenyl" or "2,4,5,2',4',5'-Hexabromobiphenyl" or "Firemaster FF-1" or "PBB" or "Polybrominated biphenyl" or "brominated biphenyl").tw,kw,kf.	1866
30	Persistent Organic Pollutants/	4377
31	(Persistent Organic Pollutants or POP or POPs).tw,kw,kf.	30500
32	dioxin/ or exp furan derivative/ or exp dibenzofuran derivative/ or (Octachlorodibenzofuran or "F 135" or Octapolychlorinated dibenzofuran or Perchlorodibenzofuran).tw,kw.	493288
33	polychlorinated biphenyl/ or ("1,1'-Biphenyl, chloro derivs" or Aroclor or Biphenyl chlorinated or Biphenyl polychloro- or Chlophen or Chlorextol or Chlorinated biphenyl or Chlorinated diphenyl or Chlorinated diphenylene or "Chloro 1,1-biphenyl" or Chloro biphenyl or Clophen or Dykanol or Fenclor or Fenclor 42 or Inerteen or Kanechlor or Montar or Monter or Noflamol or PCB or PCBS or Phenochlor or Phenoclor or Polychlorinated biphenyl or Polychlorinated biphenyls or Polychlorobiphenyl or Polychlorobiphenyls or Pyralene or Pyranol or Santotherm or Sovol or Therminol).tw,kw.	36844
34	PCB*.tw,kw.	30803
35	Fluorocarbons/	9810
36	(perfluor* or polyfluor*).tw,kw,kf.	28530

37	(PFAS? or ((perfluorinated or per-fluorinated or polyfluorinated or poly fluorinated) adj3 alkyl substance*) or ((perfluoroalkyl* or per-fluoroalkyl* or polyfluoroalkyl* or poly-fluoroalkyl* or per-fluoro alkyl) adj3 substance*)).tw,kf.	8891
38	(PFAA? or ((perfluoroalkyl or per fluoroalkyl or per-fluoro alkyl) adj3 acid*)).tw,kw.	1948
39	(PFBA or ((perfluorobutanoic or perfluorobutyric) adj3 acid*) or heptafluoro butyric acid or Heptafluoro-1-butanoic acid or Heptafluorobutanoic acid or heptafluoro-butanoic acid or heptafluoro-butanoic acid or Heptafluorobutyric acid or heptafluoro-butyric acid or Heptafluorobutyricacid or Heptafluoro-n-butyric acid or heptaflurorbutyric acid or Kyselina heptafluormaselna or Perfluorobutanoic acid or Perfluorobutyric acid or Perfluoropropanecarboxylic acid).tw,kw.	1100
40	(PFPeA or perfluoropentanoate or ((perfluoropentanoic or perfluoro-n-pentanoic) adj3 acid) or Nonafluoro-1-pentanoic acid or nonafluoropentanoic acid or Nonafluorovaleric acid or nonafluoro-Valeric acid or Nonafluoro-valeric acid or n-Perfluoropentanoic acid or Pentanoic acid, nonafluoro- or Perfluorovaleric acid).tw,kf.	375
41	(PFHxA or ACMC-1CLF8 or IPC-PFFA-6 or Perfluorocaproic acid or Perfluorohexanoic acid or perfluoro-Hexanoic acid or Undecafluoro-1-hexanoic acid or Undecafluorohexanoic acid).tw,kf.	677
42	(PFNA or Heptadecafluornonansaeure or heptadecafluorononanoic acid or heptadecafluoro-nonanoic acid or Heptadecafluoropelargonic Acid or Heptadekafluornonansaeure or n-Heptadecafluorononanoic acid or Perfluornonansaeure or Perfluoro-n-nonanoic acid or Perfluorononan-1-oic acid or Perfluorononanoic acid or Perfluorononanoicacid or Perfluoropelargonic Acid).tw,kf.	2451
43	(PFDA or Ndfda or Nonadecafluorocapric acid or Nonadecafluorodecanoic acid or Nonadecafluoro-n-decanoic acid or Perfluorocapric acid or Perfluorodecanoic acid or Perfluoro-N-decanoic acid).tw,kf.	1134
44	(PFDoA or n-perfluorododecanoic acid or Perfluorododecanoic acid or Perfluorolauric acid or Tricosafluorododecanoic acid or tricosafluoro-dodecanoic acid or Tricosafluorolauric Acid).tw,kf.	277
45	(PFBS or 1-Perfluorobutanesulfonic acid or Nonafluoro-1-butanesulfonic acid or nonafluoro-1-Butanesulfonic acid or Nonafluoro-1-butanesulfonicAcid or nonafluorobutane sulfonic acid or Nonafluorobutane-1-sulfonic acid or Nonafluorobutanesulfonic acid or nonafluoro-butanesulfonic acid or nonafluorobutanesulphonic acid or Pentyl perfluorobutanoate or Perfluoro-1-butanesulfonic Acid or perfluorobutane sulfonic acid or perfluorobutane-1-sulfonic acid	876

	or perfluorobutanesulfonic acid or perfluorobutanesulphonic acid or perfluorobutyl sulfonic acid or PFBuS).tw,kf.	
46	(PFHxS or perfluorohexane sulfonic acid or Perfluorohexane-1-sulfonic acid or Perfluorohexane-1-sulphonic acid or Perfluorohexanesulfonic acid or perfluorohexanesulphonic acid or tridecafluorohexane-1-sulfonic acid or Perfluorohexanesulfate).tw,kf.	1775
47	("Gen X" or HFPO-DA or "ammonium 2,3,3,3-tetrafluoro-2-(heptafluoropropoxy)propanoate" or FRD-902 or " heptafluoropropyl 1,2,2,2-tetrafluoroethyl ether").tw,kf.	338
48	(Bisphenol? or BPA).tw,kw,kf.	23450
49	("2,2-(4,4'-Dihydroxydiphenyl)propane" or "2,2-Bis(4-hydroxyphenyl)propane" or "2,2-Bis(hydroxyphenyl)propane" or "2,2-Bis(p-hydroxyphenyl)propane" or "2,2-Bis-4'-hydroxyfenylpropan" or "2,2-Di(4-hydroxyphenyl)propane" or "2,2-Di(4-phenylol)propane" or "4,4'-(1-Methylethylidene)bisphenol" or "4,4'-Bisphenol A" or "4,4'-Dihydroxydiphenyl-2,2-propane" or "4,4'-Dihydroxydiphenyldimethylmethane" or "4,4'-Dihydroxydiphenylpropane" or "4,4'-Isopropylidene diphenol" or "4,4'-Isopropylidenebisphenol" or "4,4'-Isopropylidenediphenol" or "beta,beta'-Bis(p-hydroxyphenyl)propane" or "beta-Di-p-hydroxyphenylpropane" or "Biphenol A" or "Bis(4-hydroxyphenyl) dimethylmethane" or "Bis(4-hydroxyphenyl)dimethylmethane" or "Bis(4-hydroxyphenyl)propane" or "Bisferol A" or "Bisphenol" or "Bisphenol A" or "Bisphenol-A" or "BPA" or "Dimethyl bis(p-hydroxyphenyl)methane" or "Dimethylbis(p-hydroxyphenyl)methane" or "Dimethylmethylene-p,p'-diphenol" or "Diphenylolpropane" or "Ipognox 88" or "Isopropylidenebis(4-hydroxybenzene)" or "p,p'-Bisphenol A" or "p,p'-Dihydroxydiphenyldimethylmethane" or "p,p'-Dihydroxydiphenylpropane" or "p,p'-Isopropylidenebisphenol" or "p,p'-Isopropylidenediphenol" or "Parabis A" or "Phenol, (1-methylethylidene)bis-" or "Phenol, 4,4'-(1-methylethylidene)bis-" or "Phenol, 4,4'-dimethylmethylenedi-" or "Phenol, 4,4'-isopropylidenedi-" or "Pluracol 245" or "Propane, 2,2-bis(p-hydroxyphenyl)-" or "Rikabanol").tw,kw,kf.	23004
50	("2,2-(4,4'-Dihydroxydiphenyl)propane" or "2,2-Bis(4-hydroxyphenyl)propane" or "2,2-Bis(hydroxyphenyl)propane" or "2,2-Bis(p-hydroxyphenyl)propane" or "2,2-Bis-4'-hydroxyfenylpropan" or "2,2-Di(4-hydroxyphenyl)propane" or "2,2-Di(4-phenylol)propane" or "4,4'-(1-Methylethylidene)bisphenol" or "4,4'-Bisphenol A" or "4,4'-Dihydroxydiphenyl-2,2-propane" or "4,4'-Dihydroxydiphenyldimethylmethane" or "4,4'-Dihydroxydiphenylpropane" or "4,4'-Isopropylidene diphenol" or "4,4'-Isopropylidenebisphenol" or "4,4'-Isopropylidenediphenol" or "beta,beta'-Bis(p-hydroxyphenyl)propane" or "beta-Di-p-hydroxyphenylpropane" or "Biphenol A" or "Bis(4-hydroxyphenyl) dimethylmethane" or "Bis(4-hydroxyphenyl)dimethylmethane" or "Bis(4-	206

	hydroxyphenyl)propane" or "Bisferol A" or "Dimethyl bis(p-hydroxyphenyl)methane" or "Dimethylbis(p-hydroxyphenyl)methane" or "Dimethylmethylene-p,p'-diphenol" or "Diphenylolpropane" or "Ipognox 88" or "Isopropylidenebis(4-hydroxybenzene)" or "p,p'-Dihydroxydiphenyldimethylmethane" or "p,p'-Dihydroxydiphenylpropane" or "p,p'-Isopropylidenebisphenol" or "p,p'-Isopropylidenediphenol" or "Parabis A" or "Phenol, (1-methylethylidene)bis-" or "Phenol, 4,4'-(1-methylethylidene)bis-" or "Phenol, 4,4'-dimethylmethylenedi-" or "Phenol, 4,4'-isopropylidenedi-" or "Pluracol 245" or "Propane, 2,2-bis(p-hydroxyphenyl)-" or "Rikabanol").tw,kw,kf.	
51	("1,1-bis(4-hydroxyphenyl) ethane" or "1,1-Bis(4-hydroxyphenyl)ethane" or "4,4'-(ETHANE-1,1-DIYL)DIPHENOL" or "4,4'-ethane-1,1-diyldiphenol" or "4,4'-Ethylidenebisphenol" or "4,4'-Ethylidenediphenol" or "4-[1-(4-hydroxyphenyl)ethyl]phenol" or "Bisphenol E" or "Phenol, 4,4'-ethylidenebis-").tw,kw,kf.	110
52	("1,1'-Sulfonylbis(4-hydroxybenzene)" or "1,1'-Sulfonylbis[4-hydroxybenzene]" or "4-(4-hydroxybenzenesulfonyl)phenol" or "4-(4-hydroxyphenyl)sulfonylphenol" or "4-(4-hydroxyphenylsulfonyl)phenol" or "4,4'-sulfonyldiphenol" or "4,4'-Bisphenol S" or "4,4'-dihydroxy diphenyl sulfone" or "4,4'-dihydroxy diphenyl sulphone" or "4,4'-dihydroxy diphenylsulfone" or "4,4'-Dihydroxydiphenyl sulfone" or "4,4'-Dihydroxydiphenyl sulfone (4,4'-DHDPS)" or "4,4'-Dihydroxydiphenyl sulfone" or "4,4'-Dihydroxydiphenyl sulphone" or "4,4'-Dihydroxydiphenyl sulphone (4,4'-DHDPS)" or "4,4'-dihydroxydiphenylsulfone" or "4,4'-Dihydroxydiphenylsulphone" or "4,4'-Sulfonylbisphenol" or "4,4'-Sulfonyldiphenol" or "4,4'-Sulfonyldiphenol (4,4'-Dihydroxydiphenylsulfone)" or "4,4'-Sulphonyldiphenol" or "4-[(4-hydroxyphenyl)sulfonyl]phenol" or "4-Hydroxyphenyl sulfone" or "bis(4-hydroxylphenyl) sulfone" or "Bis(4-hydroxyphenyl) sulfone" or "Bis(4-hydroxyphenyl)sulfone" or "Bis(p-hydroxyphenyl) sulfone" or "Bis(p-hydroxyphenyl)sulfone" or "di-(4-Hydroxyphenyl)sulfone" or "Diphone C" or "P,P'-Dihydroxydiphenyl sulfone" or "Phenol, 4,4'-sulfonylbis-" or "Phenol,4'-sulfonylbis-").tw,kw,kf.	78
53	("4-(4-hydroxybenzyl)phenol" or "4,4'-Bis(hydroxyphenyl)methane" or "4,4'-bisphenol F" or "4,4''-Dihydroxydiphenylmethane" or "4,4'-Dihydroxydiphenylmethane" or "4,4-Dihydroxydiphenylmethane" or "4,4'-methanediyl-di-phenol" or "4,4'-Methylene diphenol" or "4,4'-Methylenebis(phenol)" or "4,4'-Methylenebisphenol" or "4,4'-methylenebis-phenol" or "4,4'-Methylenediphenol" or "4,4-Methylenediphenol" or "4-[(4-hydroxyphenyl)methyl]phenol" or "Bis(4-hydroxyphenyl)methane" or "Bis-(4-hydroxyphenyl)methane" or "bis(para-hydroxyphenyl)methane" or "bis-(p-hydroxyphenyl)methane" or "bis-(p-hydroxyphenyl)-methane" or "methyl2-methoxypropionate" or "p-(p-Hydroxybenzyl)phenol" or "p,p'-Bis(hydroxyphenyl)methane" or "p,p'-BPF" or "p,p'-Methylenediphenol" or "para-(para-	56

	hydroxybenzyl)phenol" or "Phenol, 4,4'-methylenebis-" or "Phenol, 4,4'-methylenedi-" or "Phenol,4'-methylenebis-").tw,kw,kf.	
54	Triclosan/	7351
55	("2' hydroxy 2,4,4' trichlorodiphenyl ether" or "2-(2,4-dichlorophenoxy)-5-chlorophenol" or "2,3',4 trichloro 2' hydroxydiphenyl ether" or "2,4,4' trichloro 2' hydroxydiphenyl ether" or "2,4,4'-Trichloro-2'-hydroxy diphenyl ether" or "2,4,4'-Trichloro-2'-hydroxydiphenyl ether" or "2,4,4'-trichloro-2'-hydroxydiphenylethe" or "2-[2,4-bis(chloranyl)phenoxy]-5-chloranyl-phenol" or "5 chloro 2 (2,4 dichlorophenoxy)phenol" or "5-chloro-2-(2, 4-dichlorophenoxy)phenol" or "5-Chloro-2-(2,4-dichloro-phenoxy)-phenol" or "5-chloro-2-(2,4-di-chloro-phenoxy)-phenol" or "aquasept" or "cgp 433" or "cgp433" or "ch 3565" or "ch3565" or "cloxifenol" or "Cloxifenolum" or "dp 300" or "dp300" or "DP-300" or "Ether, 2'-hydroxy-2,4,4'-trichlorodiphenyl" or "Irgasan" or "Lexol 300" or "manusept" or "Neostrata" or "novaderm" or "Phenol, 5-chloro-2-(2,4-dichlorophenoxy)-" or "Phenyl ether, 2'-hydroxy-2,4,4'-trichloro-" or "Sapoderm" or "Sterzac" or "Stri-Dex Cleansing Bar" or "Stri-Dex Face Wash" or "Trichlorosan" or "triclosan" or "Triclosanum").tw,kw,kf.	5624
56	or/10-55 [Environmental and chemical exposures]	2998366
57	("German Environmental Survey" or "GerES" or "Canadian Health Measures Survey" or "CHMS" or "National human biomonitoring programme in France" or "Human Biomonitoring Programme for Ireland" or "HBM4IRE" or "Korea National Health and Nutrition Examination Survey" or "KNHANES" or "China National Human Biomonitoring" or "CNHBM" or "Human biomonitoring for Europe" or "European Human Biomonitoring Initiative" or HBM4EU or "Partnership for the Assessment of Risks in Chemicals" or "Demonstration of a study to Coordinate and Perform Human Biomonitoring on a European Scale" or DEMOCOPHES or "Consortium to Perform Human Biomonitoring on a European Scale" or COPHES or "Human Biomonitoring in Europe" or EUHBM or National Biomonitoring Program* or "National Health and Nutrition Examination Survey" or NHANES).tw,kw,kf.	38644
58	3 and 9 and 56	2264
59	3 and 57	645
60	or/58-59	2847

Search Strategy: Global Health

1973 to 2025 Week 31

#	**Searches**	**Results**
1	artificial intelligence/ or machine learning/	8571
2	(artificial intelligen* or deep* learning or expert system? or comput* learning* or intelligen* automation* or intelligen* retrieval or knowledge engineering or machine learn* or natural language process* or Generative language* or machine* learning or cognitive comput* or automat* reason* or intelligen* automat* or synthentic* intelligen* or smart* machine*).ti,ab.	16232
3	or/1-2 [Machine Learning]	17198
4	hair analysis/ or blood analysis/ or blood plasma/ or urine/ or urine analysis/ or faeces/ or human milk/ or saliva/ or semen/ or seminal plasma/ or serum/ or sputum/ or sweat/	196537
5	((plasma or blood or serum or feces or faeces or fecal or faecal or stool or urin* or hair or human milk or breast milk or placenta or semen or seminal fluid* or seminal plasma* or sweat or perspirat* or saliva* or sputum) adj3 (sampl* or test* or level* or analy* or concentration*)).ti,ab.	470927
6	((exhal* or expir*) adj2 (breath or air)).ti,ab.	1272
7	(biomonitor* or bio monitor* or biological monitor*).ti,ab.	5712
8	or/4-7 [Biomonitoring]	570426
9	exposure/	105637
10	(chemical* or agrochemical* or pollut* or contamina* or leachate* or runoff* or spray drift or toxic* or poison* or neurotox* or embryotox* or cytotox* or hepatotox* or fetotox* or genotox* or maternotox* or immunotox* or dermatotox* or cardiotoxic* or nephrotoxic* or ototox*).ti,ab.	738561
11	Metals/ or ((Plasma or blood or serum or feces or faeces or fecal or faecal or serum or stool or urin* or hair or human milk or breast milk or placenta or semen or seminal fluid* or plasma* or sweat or perspirat* or saliva* or sputumtest* or level* or analy* or concentration* or expos* or biomonitor* or biological monitor*) adj4 metal*).ti,ab.	33806
12	Mercury/ or (mercury or Hydrargyrum or mercure or Mercurio or Quecksilber or Quicksilver).ti,ab.	20368

13	Lead/ or (((lead or leaded or pb) and metal?) or plumbum or olow).ti,ab.	44461
14	Cadmium/ or (cadmium or kadmium).ti,ab.	35921
15	Arsenic/ or arsenic*.ti,ab.	25537
16	exp Pesticides/	287483
17	(avicid* or biocid* or biopesticid* or chemosteril* or fumigant* or fungicid* or herbicid* or insecticid* or larvicid* or molluscacid* or nematicid* or pesticid* or piscicid* or rodenticid*).ti,ab.	104377
18	polybrominated biphenyls/ or organobromine compounds/ or polychlorinated biphenyls/	9961
19	((organophosph* or OER? or flame?) adj5 retardant*).ti,ab.	2525
20	OPFR?.ti,ab.	275
21	("bis(2-chloroethyl) 2-hydroxyethyl phosphate" or "Bis(2-chloroethyl) hydrogen phosphate" or "bis-(2-Chloroethyl) hydrogen phosphate" or "bis(2-chloroethyl) phosphate" or "Bis-(2-chloroethyl) phosphate" or "Di-beta,beta'-Chloroethylphosphoric Acid" or "Ethanol, 2-chloro-, hydrogen phosphate" or "Ethanol, 2-chloro-,1,1'-(hydrogen phosphate)").ti,ab.	71
22	("Bis-(2-chloroisopropyl) phosphate" or "2-Propanol, 1,3-dichloro-, hydrogen phosphate" or "bis(1,3-dichloro-2-propyl) hydrogen phosphate" or "Bis(1,3-dichloro-2-propyl) phosphate" or "Bis-(1,3-dichloro-2-propyl) phosphate" or "bis(1,3-dichloropropan-2-yl) hydrogen phosphate" or "Phosphoric acid bis(1,3-dichloropropane-2-yl) ester").ti,ab.	98
23	("(PhO)2(HO)PO" or "diphenoxyphosphinic acid" or "Diphenyl hydrogen phosphate" or "Diphenyl phosphate" or "diphenylphosphoric acid" or "Phenyl hydrogen phosphate" or "Phenyl phosphate" or "Phosphoric acid diphenyl" or "Phosphoric acid diphenyl ester").ti,ab.	305
24	"1-Hydroxyl-2-propyl bis(1-chloro-2-propyl) phosphate".mp.	1
25	("Bis(1-chloro-2-isopropyl) 1-hydroxy-2-isopropyl phosphate" or "Isopropylphenyl phenyl phosphate" or "Diphenyl isopropylphenyl phosphate" or "diphenyl propan-2-yl phosphate" or "Isopropyl diphenyl phosphate" or "Phosphoric acid isopropyldiphenyl ester" or "Tetra-brominated benzoic acid" or "3-Bromobenzenecarboxylic acid" or "3-BROMOBENZOIC ACID" or "3-Bromobenzoic acid" or "3-Bromobenzoic acid" or "3-Bromo-benzoic acid" or "3-Bromobenzoicacid" or "3-bromophenyl carboxylic acid" or "5-bromobenzoic acid" or "Benzoic acid, 3-bromo-" or "Benzoic acid, m-bromo-" or "m-Bromobenzoic acid" or "meta bromobenzoic acid" or "Phosphoric acid tert-butyldiphenyl	7

	ester" or "Phosphoric acid, 1,1-dimethylethyl diphenyl ester" or "t-butyl diphenyl phosphate" or "t-butyl phenyl phenyl phosphate" or "tert-butyl diphenyl phosphate").mp.	
26	(BFR and (bromin* or flame*)).ti,ab.	168
27	("Tetrabromobisphenol A" or "3,3',5,5'-Tetrabromobisphenol A" or "4,4'-(propane-2,2-diyl)bis(2,6-dibromophenol)" or "Bromdian" or "4,4'-Isopropylidenebis(2,6-dibromophenol)" or "2,2-Bis(3,5-dibromo-4-hydroxyphenyl)propane" or "TBBPA" or "Firemaster BP 4A" or "Tetrabromodian" or "Fire Guard 2000" or "Great Lakes BA-59P" or "Saytex RB 100PC" or "Tetrabromodiphenylopropane" or "Firemaster BP4A" or "2,2',6,6'-TETRABROMOBISPHENOL A" or "2,6-dibromo-4-[2-(3,5-dibromo-4-hydroxyphenyl)propan-2-yl]phenol" or "FG 2000" or "Phenol, 4,4'-(1-methylethylidene)bis[2,6-dibromo-" or "4,4'-(1-Methylethylidene)bis(2,6-dibromophenol)" or "3,5,3',5'-Tetrabromobisphenol A" or "NSC 59775" or "4,4'-propane-2,2-diylbis(2,6-dibromophenol)" or "Saytex RB-100" or "4,4'-Isopropylylidenebis(2,6-dibromophenol)" or "2,2-Bis(4-hydroxy-3,5-dibromophenyl)propane" or "FLAME CUT 120G" or "2,2',6,6'-Tetrabromo-4,4'-isopropylidenediphenol" or "Phenol, 4,4'-isopropylidenebis(2,6-dibromo-" or "Phenol, 4,4'-isopropylidenebis[2,6-dibromo-" or "4,4'-(2,2-propanediyl) bis[2,6-dibromo]phenol" or "Phenol, 4,4'-(1-methylethylidene)bis(2,6-dibromo-" or "TETRABROMO-4,4'-ISOPROPYLIDENEDIPHENOL" or "Tetrabromobisphenol A 50 microg/mL in Methanol" or "2,2,6,6-Tetrabromo-4,4-Isopropylidene Phenol" or "4,4'-(1-Methylethylidene)bis(2,6-dibromophenol)2,2-bis(3,5-dibromo-4-hydroxyphenyl)propane" or "2,6-dibromo-4-[1-(3,5-dibromo-4-hydroxyphenyl)-1-methylethyl]phenol" or "Tetrabromo bisphenol A" or "4,4'-(1-methylethylidene)bis[2,6-dibromophenol]" or "3,3',5,5'-Tetrabromo bisphenol A" or "4,4'-(2,2-PROPANEDIYL)BIS(2,6-DIBROMOPHENOL)" or "4,6-dibromophenol)" or "Tetrabromobis phenol A" or "TBBP-A" or "Saytex RB-100 ABS" or "2,5-dibromophenyl)propane" or "bmse000567" or "Tetrabromobisphenol ''A''" or "2,6,6'-Tetrabromobisphenol A" or "33'55'-Tetrabromobisphenol A" or "Tetrabromobisphenol A (TBBP A)" or "3,3',5'-Tetrabromobisphenol A" or "2,5-dibromo-4-hydroxyphenyl)propane" or "2,6-dibromo-4-[1-(3,5-dibromo-4-hydroxy-phenyl)-1-methyl-ethyl]phenol" or "2,2'',6,6''-Tetrabromobisphenol A" or "3,3'',5,5''-tetrabromobisphenol A" or "Phenol,4'-isopropylidenebis[2,6-dibromo-" or "phenol, 4,4'-isopropylidenebis (dibromo-)" or "2,2-bis(3,5dibromo-4-hydroxyphenyl)propane" or "4,4''-Isopropylidenebis(2,6-dibromophenol)" or "4,4'-isopropylidene-bis(2,6-dibromophenol)" or "2,2-bis(3,5-dibromo-4-hydroxyphenyl)-propane" or "2,2-bis-(3,5-dibromo-4-hydroxyphenyl)propane" or "2,2-bis-(3,5-dibromo-4-hydroxyphenyl)-propane" or "2,2',6,6'-tetrabrom-4,4'-isopropylidendiphenol" or "2,2',6,6'-TETRABROMOBISPHENOL A [HSDB]" or "Fenol, 4,4'-(1-metiletilideno)bis[2,6-dibromo-" or "Phenol,4'-(1-methylethylidene)bis[2,6-dibromo-" or "Tetrabromodian: tetrabromodihydroxy diphenylpropane" or "4,4''-(1-Methylethylidene)bis(2,6-dibromophenol)" or "4,4''-(2,2-propanediyl) bis[2,6-	381

	dibromo]phenol" or "4,4''-(propane-2,2-diyl)bis(2,6-dibromophenol)" or "2,2',6,6'-Tetrabromo-4,4'-isopropylidene bisphenol" or "2,2-bis-(4'-hydroxy-3',5'-dibromophenyl)-propane" or "3,3',5,5'-Tetrabromobisphenol A, analytical standard" or "3,3',5,5'-Tetrabromo-4,4-dihydroxy-2,2-diphenylpropane" or "Phenol, 4,4'-(1-methylethylidene)bis-, tetrabromo deriv." or "3,3',5,5'-Tetrabromo-4,4'-dihydroxy-diphenyl-dimethyl-methane" or "3,3',5,5'-Tetrabromobisphenol A, certified reference material, TraceCERT(R)").ti,ab.	
28	("2,2',4,4',5,5'-hexabromobiphenyl" or "2,4,5,2',4',5'-Hexabromobiphenyl" or "Firemaster FF-1" or "PBB" or "Polybrominated biphenyl" or "brominated biphenyl").ti,ab.	503
29	(Persistent Organic Pollutants or POP or POPs).ti,ab.	4719
30	dioxin/ or exp furan derivative/ or exp dibenzofuran derivative/ or (Octachlorodibenzofuran or "F 135" or Octapolychlorinated dibenzofuran or Perchlorodibenzofuran).ti,ab.	5049
31	polychlorinated biphenyl/ or ("1,1'-Biphenyl, chloro derivs" or Aroclor or Biphenyl chlorinated or Biphenyl polychloro- or Chlophen or Chlorextol or Chlorinated biphenyl or Chlorinated diphenyl or Chlorinated diphenylene or "Chloro 1,1-biphenyl" or Chloro biphenyl or Clophen or Dykanol or Fenclor or Fenclor 42 or Inerteen or Kanechlor or Montar or Monter or Noflamol or PCB or PCBS or Phenochlor or Phenoclor or Polychlorinated biphenyl or Polychlorinated biphenyls or Polychlorobiphenyl or Polychlorobiphenyls or Pyralene or Pyranol or Santotherm or Sovol or Therminol).ti,ab.	9920
32	PCB*.ti,ab.	8320
33	(perfluor* or polyfluor*).ti,ab.	4996
34	(PFAS? or ((perfluorinated or per-fluorinated or polyfluorinated or poly fluorinated) adj3 alkyl substance*) or ((perfluoroalkyl* or per-fluoroalkyl* or polyfluoroalkyl* or poly-fluoroalkyl* or per-fluoro alkyl) adj3 substance*)).ti,ab.	3183
35	(PFAA? or ((perfluoroalkyl or per fluoroalkyl or per-fluoro alkyl) adj3 acid*)).ti,ab.	661
36	(PFBA or ((perfluorobutanoic or perfluorobutyric) adj3 acid*) or heptafluoro butyric acid or Heptafluoro-1-butanoic acid or Heptafluorobutanoic acid or heptafluoro-butanoic acid or heptafluoro-butanoic acid or Heptafluorobutyric acid or heptafluoro-butyric acid or Heptafluorobutyricacid or Heptafluoro-n-butyric acid or heptaflurorbutyric acid or Kyselina heptafluormaselna or Perfluorobutanoic acid or Perfluorobutyric acid or Perfluoropropanecarboxylic acid).ti,ab.	317
37	(PFPeA or perfluoropentanoate or ((perfluoropentanoic or perfluoro-n-pentanoic) adj3 acid) or Nonafluoro-1-pentanoic acid or nonafluoropentanoic acid or Nonafluorovaleric	154

	acid or nonafluoro-Valeric acid or Nonafluoro-valeric acid or n-Perfluoropentanoic acid or Pentanoic acid, nonafluoro- or Perfluorovaleric acid).ti,ab.	
38	(PFHxA or ACMC-1CLF8 or IPC-PFFA-6 or Perfluorocaproic acid or Perfluorohexanoic acid or perfluoro-Hexanoic acid or Undecafluoro-1-hexanoic acid or Undecafluorohexanoic acid).ti,ab.	254
39	(PFNA or Heptadecafluornonansaeure or heptadecafluorononanoic acid or heptadecafluoro-nonanoic acid or Heptadecafluoropelargonic Acid or Heptadekafluornonansaeure or n-Heptadecafluorononanoic acid or Perfluornonansaeure or Perfluoro-n-nonanoic acid or Perfluorononan-1-oic acid or Perfluorononanoic acid or Perfluorononanoicacid or Perfluoropelargonic Acid).ti,ab.	960
40	(PFDA or Ndfda or Nonadecafluorocapric acid or Nonadecafluorodecanoic acid or Nonadecafluoro-n-decanoic acid or Perfluorocapric acid or Perfluorodecanoic acid or Perfluoro-N-decanoic acid).ti,ab.	542
41	(PFDoA or n-perfluorododecanoic acid or Perfluorododecanoic acid or Perfluorolauric acid or Tricosafluorododecanoic acid or tricosafluoro-dodecanoic acid or Tricosafluorolauric Acid).ti,ab.	124
42	(PFBS or 1-Perfluorobutanesulfonic acid or Nonafluoro-1-butanesulfonic acid or nonafluoro-1-Butanesulfonic acid or Nonafluoro-1-butanesulfonicAcid or nonafluorobutane sulfonic acid or Nonafluorobutane-1-sulfonic acid or Nonafluorobutanesulfonic acid or nonafluoro-butanesulfonic acid or nonafluorobutanesulphonic acid or Pentyl perfluorobutanoate or Perfluoro-1-butanesulfonic Acid or perfluorobutane sulfonic acid or perfluorobutane-1-sulfonic acid or perfluorobutanesulfonic acid or perfluorobutanesulphonic acid or perfluorobutyl sulfonic acid or PFBuS).ti,ab.	321
43	(PFHxS or perfluorohexane sulfonic acid or Perfluorohexane-1-sulfonic acid or Perfluorohexane-1-sulphonic acid or Perfluorohexanesulfonic acid or perfluorohexanesulphonic acid or tridecafluorohexane-1-sulfonic acid or Perfluorohexanesulfate).ti,ab.	968
44	("Gen X" or HFPO-DA or "ammonium 2,3,3,3-tetrafluoro-2-(heptafluoropropoxy)propanoate" or FRD-902 or " heptafluoropropyl 1,2,2,2-tetrafluoroethyl ether").ti,ab.	79
45	(Bisphenol? or BPA or BPE or BPS or BPF or BPAF).ti,ab.	7377
46	("2,2-(4,4'-Dihydroxydiphenyl)propane" or "2,2-Bis(4-hydroxyphenyl)propane" or "2,2-Bis(hydroxyphenyl)propane" or "2,2-Bis(p-hydroxyphenyl)propane" or "2,2-Bis-4'-hydroxyfenylpropan" or "2,2-Di(4-hydroxyphenyl)propane" or "2,2-Di(4-	5729

	phenylol)propane" or "4,4'-(1-Methylethylidene)bisphenol" or "4,4'-Bisphenol A" or "4,4'-Dihydroxydiphenyl-2,2-propane" or "4,4'-Dihydroxydiphenyldimethylmethane" or "4,4'-Dihydroxydiphenylpropane" or "4,4'-Isopropylidene diphenol" or "4,4'-Isopropylidenebisphenol" or "4,4'-Isopropylidenediphenol" or "beta,beta'-Bis(p-hydroxyphenyl)propane" or "beta-Di-p-hydroxyphenylpropane" or "Biphenol A" or "Bis(4-hydroxyphenyl) dimethylmethane" or "Bis(4-hydroxyphenyl)dimethylmethane" or "Bis(4-hydroxyphenyl)propane" or "Bisferol A" or "Bisphenol" or "Bisphenol A" or "Bisphenol-A" or "BPA" or "Dimethyl bis(p-hydroxyphenyl)methane" or "Dimethylbis(p-hydroxyphenyl)methane" or "Dimethylmethylene-p,p'-diphenol" or "Diphenylolpropane" or "Ipognox 88" or "Isopropylidenebis(4-hydroxybenzene)" or "p,p'-Bisphenol A" or "p,p'-Dihydroxydiphenyldimethylmethane" or "p,p'-Dihydroxydiphenylpropane" or "p,p'-Isopropylidenebisphenol" or "p,p'-Isopropylidenediphenol" or "Parabis A" or "Phenol, (1-methylethylidene)bis-" or "Phenol, 4,4'-(1-methylethylidene)bis-" or "Phenol, 4,4'-dimethylmethylenedi-" or "Phenol, 4,4'-isopropylidenedi-" or "Pluracol 245" or "Propane, 2,2-bis(p-hydroxyphenyl)-" or "Rikabanol").ti,ab.	
47	("2,2-(4,4'-Dihydroxydiphenyl)propane" or "2,2-Bis(4-hydroxyphenyl)propane" or "2,2-Bis(hydroxyphenyl)propane" or "2,2-Bis(p-hydroxyphenyl)propane" or "2,2-Bis-4'-hydroxyfenylpropan" or "2,2-Di(4-hydroxyphenyl)propane" or "2,2-Di(4-phenylol)propane" or "4,4'-(1-Methylethylidene)bisphenol" or "4,4'-Bisphenol A" or "4,4'-Dihydroxydiphenyl-2,2-propane" or "4,4'-Dihydroxydiphenyldimethylmethane" or "4,4'-Dihydroxydiphenylpropane" or "4,4'-Isopropylidene diphenol" or "4,4'-Isopropylidenebisphenol" or "4,4'-Isopropylidenediphenol" or "beta,beta'-Bis(p-hydroxyphenyl)propane" or "beta-Di-p-hydroxyphenylpropane" or "Biphenol A" or "Bis(4-hydroxyphenyl) dimethylmethane" or "Bis(4-hydroxyphenyl)dimethylmethane" or "Bis(4-hydroxyphenyl)propane" or "Bisferol A" or "Dimethyl bis(p-hydroxyphenyl)methane" or "Dimethylbis(p-hydroxyphenyl)methane" or "Dimethylmethylene-p,p'-diphenol" or "Diphenylolpropane" or "Ipognox 88" or "Isopropylidenebis(4-hydroxybenzene)" or "p,p'-Dihydroxydiphenyldimethylmethane" or "p,p'-Dihydroxydiphenylpropane" or "p,p'-Isopropylidenebisphenol" or "p,p'-Isopropylidenediphenol" or "Parabis A" or "Phenol, (1-methylethylidene)bis-" or "Phenol, 4,4'-(1-methylethylidene)bis-" or "Phenol, 4,4'-dimethylmethylenedi-" or "Phenol, 4,4'-isopropylidenedi-" or "Pluracol 245" or "Propane, 2,2-bis(p-hydroxyphenyl)-" or "Rikabanol").ti,ab.	36
48	("1,1-bis(4-hydroxyphenyl) ethane" or "1,1-Bis(4-hydroxyphenyl)ethane" or "4,4'-(ETHANE-1,1-DIYL)DIPHENOL" or "4,4'-ethane-1,1-diyldiphenol" or "4,4'-Ethylidenebisphenol" or "4,4'-Ethylidenediphenol" or "4-[1-(4-hydroxyphenyl)ethyl]phenol" or "Bisphenol E" or "Phenol, 4,4'-ethylidenebis-").ti,ab.	38
49	("1,1'-Sulfonylbis(4-hydroxybenzene)" or "1,1'-Sulfonylbis[4-hydroxybenzene]" or "4-(4-hydroxybenzenesulfonyl)phenol" or "4-(4-hydroxyphenyl)sulfonylphenol" or "4-(4-hydroxyphenylsulfonyl)phenol" or "4,4'-sulfonyldiphenol" or "4,4'-Bisphenol S" or "4,4'-	10

	dihydroxy diphenyl sulfone" or "4,4'-dihydroxy diphenyl sulphone" or "4,4'-dihydroxy diphenylsulfone" or "4,4'-Dihydroxydiphenyl sulfone" or "4,4'-Dihydroxydiphenyl sulfone (4,4'-DHDPS)" or "4,4'-Dihydroxydiphenyl sulfone" or "4,4'-Dihydroxydiphenyl sulphone" or "4,4'-Dihydroxydiphenyl sulphone (4,4'-DHDPS)" or "4,4'-dihydroxydiphenylsulfone" or "4,4'-Dihydroxydiphenylsulphone" or "4,4'-Sulfonylbisphenol" or "4,4'-Sulfonyldiphenol" or "4,4'-Sulfonyldiphenol (4,4'-Dihydroxydiphenylsulfone)" or "4,4'-Sulphonyldiphenol" or "4-[(4-hydroxyphenyl)sulfonyl]phenol" or "4-Hydroxyphenyl sulfone" or "bis(4-hydroxylphenyl) sulfone" or "Bis(4-hydroxyphenyl) sulfone" or "Bis(4-hydroxyphenyl)sulfone" or "Bis(p-hydroxyphenyl) sulfone" or "Bis(p-hydroxyphenyl)sulfone" or "di-(4-Hydroxyphenyl)sulfone" or "Diphone C" or "P,P'-Dihydroxydiphenyl sulfone" or "Phenol, 4,4'-sulfonylbis-" or "Phenol,4'-sulfonylbis-").ti,ab.	
50	("4-(4-hydroxybenzyl)phenol" or "4,4'-Bis(hydroxyphenyl)methane" or "4,4'-bisphenol F" or "4,4''-Dihydroxydiphenylmethane" or "4,4'-Dihydroxydiphenylmethane" or "4,4-Dihydroxydiphenylmethane" or "4,4'-methanediyl-di-phenol" or "4,4'-Methylene diphenol" or "4,4'-Methylenebis(phenol)" or "4,4'-Methylenebisphenol" or "4,4'-methylenebis-phenol" or "4,4'-Methylenediphenol" or "4,4-Methylenediphenol" or "4-[(4-hydroxyphenyl)methyl]phenol" or "Bis(4-hydroxyphenyl)methane" or "Bis-(4-hydroxyphenyl)methane" or "bis(para-hydroxyphenyl)methane" or "bis-(p-hydroxyphenyl)methane" or "bis-(p-hydroxyphenyl)-methane" or "methyl2-methoxypropionate" or "p-(p-Hydroxybenzyl)phenol" or "p,p'-Bis(hydroxyphenyl)methane" or "p,p'-BPF" or "p,p'-Methylenediphenol" or "para-(para-hydroxybenzyl)phenol" or "Phenol, 4,4'-methylenebis-" or "Phenol, 4,4'-methylenedi-" or "Phenol,4'-methylenebis-").ti,ab.	12
51	Triclosan/	1592
52	("2' hydroxy 2,4,4' trichlorodiphenyl ether" or "2-(2,4-dichlorophenoxy)-5-chlorophenol" or "2,3',4 trichloro 2' hydroxydiphenyl ether" or "2,4,4' trichloro 2' hydroxydiphenyl ether" or "2,4,4'-Trichloro-2'-hydroxy diphenyl ether" or "2,4,4'-Trichloro-2'-hydroxydiphenyl ether" or "2,4,4'-trichloro-2'-hydroxydiphenylethe" or "2-[2,4-bis(chloranyl)phenoxy]-5-chloranyl-phenol" or "5 chloro 2 (2,4 dichlorophenoxy)phenol" or "5-chloro-2-(2, 4-dichlorophenoxy)phenol" or "5-Chloro-2-(2,4-dichloro-phenoxy)-phenol" or "5-chloro-2-(2,4-di-chloro-phenoxy)-phenol" or "aquasept" or "cgp 433" or "cgp433" or "ch 3565" or "ch3565" or "cloxifenol" or "Cloxifenolum" or "dp 300" or "dp300" or "DP-300" or "Ether, 2'-hydroxy-2,4,4'-trichlorodiphenyl" or "Irgasan" or "Lexol 300" or "manusept" or "Neostrata" or "novaderm" or "Phenol, 5-chloro-2-(2,4-dichlorophenoxy)-" or "Phenyl ether, 2'-hydroxy-2,4,4'-trichloro-" or "Sapoderm" or "Sterzac" or "Stri-Dex Cleansing Bar" or "Stri-Dex Face Wash" or "Trichlorosan" or "triclosan" or "Triclosanum").ti,ab.	2033
53	or/10-52 [Environmental and chemical exposures]	997870

54	("German Environmental Survey" or "GerES" or "Canadian Health Measures Survey" or "CHMS" or "National human biomonitoring programme in France" or "Human Biomonitoring Programme for Ireland" or "HBM4IRE" or "Korea National Health and Nutrition Examination Survey" or "KNHANES" or "China National Human Biomonitoring" or "CNHBM" or "Human biomonitoring for Europe" or "European Human Biomonitoring Initiative" or HBM4EU or "Partnership for the Assessment of Risks in Chemicals" or "Demonstration of a study to Coordinate and Perform Human Biomonitoring on a European Scale" or DEMOCOPHES or "Consortium to Perform Human Biomonitoring on a European Scale" or COPHES or "Human Biomonitoring in Europe" or EUHBM or National Biomonitoring Program* or "National Health and Nutrition Examination Survey" or NHANES).ti,ab.	16560
55	3 and 8 and 53	185
56	3 and 54	132
57	3 and (8 or 53) and 54	67
58	or/55-57	290

Search Strategy: Scopus

((TITLE-ABS-KEY (("artificial intelligen*" OR "deep* learning" OR "expert system*" OR "comput* learning*" OR "intelligen* automation*" OR "intelligen* retrieval" OR "knowledge engineering" OR "machine learn*" OR "natural language process*" OR "Generative language*" OR "machine* learning" OR "cognitive comput*" OR "automat* reason*" OR "intelligen* automat*" OR "synthentic* intelligen*" OR "smart* machine*"))) AND ((TITLE-ABS-KEY (((plasma OR blood OR serum OR feces OR faeces OR fecal OR faecal OR stool OR urin* OR hair OR "human milk" OR "breast milk" OR placenta OR semen OR "seminal fluid*" OR "seminal plasma*" OR sweat OR perspirat* OR saliva* OR sputum) W/3 (sampl* OR test* OR level* OR analy* OR concentration*))) OR TITLE-ABS-KEY (((exhal* OR expir*) W/2 (breath OR air))) OR TITLE-ABS-KEY ((biomonitor* OR "bio monitor*" OR "biological monitor*")))) AND (((TITLE-ABS-KEY ((chemical* OR agrochemical* OR pollut* OR contamina* OR leachate* OR runoff* OR "spray drift" OR toxic* OR poison* OR neurotox* OR embryotox* OR cytotox* OR hepatotox* OR fetotox* OR genotox* OR maternotox* OR immunotox* OR dermatotox* OR cardiotoxic* OR nephrotoxic* OR ototox*)) OR TITLE-ABS-KEY (((plasma OR blood OR serum OR feces OR faeces OR fecal OR faecal OR serum OR stool OR urin* OR hair OR "human milk" OR "breast milk" OR placenta OR semen OR "seminal fluid*" OR plasma* OR sweat OR perspirat* OR saliva* OR sputumtest* OR level* OR analy* OR concentration* OR expos* OR biomonitor* OR "biological monitor*") W/4 metal*)) OR TITLE-ABS-KEY ((mercury OR hydrargyrum OR mercure OR mercurio OR quecksilber OR quicksilver OR plumbum OR olow OR cadmium OR kadmium OR arsenic*)) OR TITLE-ABS-KEY ((avicid* OR biocid* OR biopesticid* OR chemosteril* OR fumigant* OR fungicid* OR herbicid* OR insecticid* OR larvicid* OR

molluscacid* OR nematicid* OR pesticid* OR piscicid* OR rodenticid*)) OR TITLE-ABS-KEY (((organophosph* OR oer* OR flame*) W/5 retardant*)) OR TITLE-ABS-KEY ((opfr*)) OR TITLE-ABS-KEY (("bis(2-chloroethyl) 2-hydroxyethyl phosphate" OR "Bis(2-chloroethyl) hydrogen phosphate" OR "bis-(2-Chloroethyl) hydrogen phosphate" OR "bis(2-chloroethyl) phosphate" OR "Bis-(2-chloroethyl) phosphate" OR "Di-beta,beta'-Chloroethylphosphoric Acid" OR "Ethanol, 2-chloro-, hydrogen phosphate" OR "Ethanol, 2-chloro-,1,1'-(hydrogen phosphate)")) OR TITLE-ABS-KEY (("Bis-(2-chloroisopropyl) phosphate" OR "2-Propanol, 1,3-dichloro-, hydrogen phosphate" OR "bis(1,3-dichloro-2-propyl) hydrogen phosphate" OR "Bis(1,3-dichloro-2-propyl) phosphate" OR "Bis-(1,3-dichloro-2-propyl) phosphate" OR "bis(1,3-dichloropropan-2-yl) hydrogen phosphate" OR "Phosphoric acid bis(1,3-dichloropropane-2-yl) ester")) OR TITLE-ABS-KEY ("diphenoxyphosphinic acid" OR "Diphenyl hydrogen phosphate" OR "Diphenyl phosphate" OR "diphenylphosphoric acid" OR "Phenyl hydrogen phosphate" OR "Phenyl phosphate" OR "Phosphoric acid diphenyl" OR "Phosphoric acid diphenyl ester") OR TITLE-ABS-KEY (("1-Hydroxyl-2-propyl bis(1-chloro-2-propyl) phosphate")) OR TITLE-ABS-KEY (("Bis(1-chloro-2-isopropyl) 1-hydroxy-2-isopropyl phosphate" OR "Isopropylphenyl phenyl phosphate" OR "Diphenyl isopropylphenyl phosphate" OR "diphenyl propan-2-yl phosphate" OR "Isopropyl diphenyl phosphate" OR "Phosphoric acid isopropyldiphenyl ester" OR "Tetra-brominated benzoic acid" OR "3-Bromobenzenecarboxylic acid" OR "3-BROMOBENZOIC ACID")) OR TITLE-ABS-KEY ((bfr AND (bromin* OR flame*))))) OR ((TITLE-ABS-KEY ((pfhxs OR "perfluorohexane sulfonic acid")) OR TITLE-ABS-KEY (("Gen X" OR "HFPO-DA")) OR TITLE-ABS-KEY ((bisphenol* OR bpa OR bpe OR bps OR bpf OR bpaf)))) OR ((TITLE-ABS-KEY ((pfba OR ((perfluorobutanoic OR perfluorobutyric) W/3 acid*) OR "heptafluoro butyric acid" OR "Heptafluoro-1-butanoic acid" OR "Heptafluorobutanoic acid" OR "heptafluoro-butanoic acid" OR "heptafluoro-butanoic acid" OR "Heptafluorobutyric acid" OR "heptafluoro-butyric acid" OR heptafluorobutyricacid OR "Heptafluoro-n-butyric acid" OR "heptaflurorbutyric acid" OR "Kyselina heptafluormaselna" OR "Perfluorobutanoic acid" OR "Perfluorobutyric acid" OR "Perfluoropropanecarboxylic acid")) OR TITLE-ABS-KEY ((pfpea OR perfluoropentanoate OR ((perfluoropentanoic OR "perfluoro-n-pentanoic") W/3 acid) OR "Nonafluoro-1-pentanoic acid" OR "nonafluoropentanoic acid" OR "Nonafluorovaleric acid" OR "nonafluoro-Valeric acid" OR "Nonafluoro-valeric acid" OR "n-Perfluoropentanoic acid" OR "Pentanoic acid, nonafluoro-" OR "Perfluorovaleric acid")) OR TITLE-ABS-KEY ((pfhxa OR "ACMC-1CLF8" OR "IPC-PFFA-6" OR "Perfluorocaproic acid" OR "Perfluorohexanoic acid" OR "perfluoro-Hexanoic acid" OR "Undecafluoro-1-hexanoic acid" OR "Undecafluorohexanoic acid")) OR TITLE-ABS-KEY ((pfna OR heptadecafluornonansaeure OR "heptadecafluorononanoic acid" OR "heptadecafluoro-nonanoic acid" OR "Heptadecafluoropelargonic Acid" OR heptadekafluornonansaeure OR "n-Heptadecafluorononanoic acid" OR perfluornonansaeure OR "Perfluoro-n-nonanoic acid" OR "Perfluorononan-1-oic acid" OR "Perfluorononanoic acid" OR perfluorononanoicacid OR "Perfluoropelargonic Acid")) OR TITLE-ABS-KEY ((pfda OR ndfda OR "Nonadecafluorocapric acid" OR "Nonadecafluorodecanoic acid" OR "Nonadecafluoro-n-decanoic acid" OR "Perfluorocapric acid" OR "Perfluorodecanoic acid" OR "Perfluoro-N-decanoic acid")) OR TITLE-ABS-KEY ((pfdoa OR "n-perfluorododecanoic acid" OR "Perfluorododecanoic acid" OR "Perfluorolauric acid" OR "Tricosafluorododecanoic acid" OR "tricosafluoro-dodecanoic acid" OR "Tricosafluorolauric Acid")) OR TITLE-ABS-KEY (pfbs OR "1-Perfluorobutanesulfonic acid" OR "Nonafluoro-1-butanesulfonic acid" OR "nonafluoro-1-Butanesulfonic

acid"))) OR ((TITLE-ABS-KEY (("Tetrabromobisphenol A" OR "3,3',5,5'-Tetrabromobisphenol A" OR "2,2',4,4',5,5'-hexabromobiphenyl" OR "2,4,5,2',4',5'-Hexabromobiphenyl" OR "Firemaster FF-1" OR pbb OR "Polybrominated biphenyl" OR "brominated biphenyl")) OR TITLE-ABS-KEY ("Persistent Organic Pollutants") OR TITLE-ABS-KEY ((octachlorodibenzofuran OR "F 135" OR "Octapolychlorinated dibenzofuran" OR perchlorodibenzofuran)) OR TITLE-ABS-KEY (("1,1'-Biphenyl, chloro derivs" OR aroclor OR "Biphenyl chlorinated" OR "Biphenyl polychloro-" OR chlophen OR chlorextol OR "Chlorinated biphenyl" OR "Chlorinated diphenyl" OR "Chlorinated diphenylene" OR "Chloro 1,1-biphenyl" OR "Chloro biphenyl" OR clophen OR dykanol OR fenclor OR "Fenclor 42" OR inerteen OR kanechlor OR montar OR monter OR noflamol OR pcb OR pcbs OR phenochlor OR phenoclor OR "Polychlorinated biphenyl" OR "Polychlorinated biphenyls" OR polychlorobiphenyl OR polychlorobiphenyls OR pyralene OR pyranol OR santotherm OR sovol OR therminol)) OR TITLE-ABS-KEY (pcb* OR perfluor* OR polyfluor*) OR TITLE-ABS-KEY ((pfas* OR ((perfluorinated OR "per-fluorinated" OR polyfluorinated OR "poly fluorinated") W/3 "alkyl substance*") OR ((perfluoroalkyl* OR "per-fluoroalkyl*" OR polyfluoroalkyl* OR "poly-fluoroalkyl*" OR "per-fluoro alkyl") W/3 substance*))) OR TITLE-ABS-KEY ((pfaa* OR ((perfluoroalkyl OR "per fluoroalkyl" OR "per-fluoro alkyl") W/3 acid*))))))) OR ((TITLE-ABS-KEY (("artificial intelligen*" OR "deep* learning" OR "expert system*" OR "comput* learning*" OR "intelligen* automation*" OR "intelligen* retrieval" OR "knowledge engineering" OR "machine learn*" OR "natural language process*" OR "Generative language*" OR "machine* learning" OR "cognitive comput*" OR "automat* reason*" OR "intelligen* automat*" OR "synthentic* intelligen*" OR "smart* machine*"))) AND (TITLE-ABS-KEY (("German Environmental Survey" OR geres OR "Canadian Health Measures Survey" OR chms OR "National human biomonitoring programme in France" OR "Human Biomonitoring Programme for Ireland" OR hbm4ire OR "Korea National Health and Nutrition Examination Survey" OR knhanes OR "China National Human Biomonitoring" OR cnhbm OR "Human biomonitoring for Europe" OR "European Human Biomonitoring Initiative" OR hbm4eu OR "Partnership for the Assessment of Risks in Chemicals" OR "Demonstration of a study to Coordinate and Perform Human Biomonitoring on a European Scale" OR democophes OR "Consortium to Perform Human Biomonitoring on a European Scale" OR cophes OR "Human Biomonitoring in Europe" OR euhbm OR "National Biomonitoring Program*" OR "National Health and Nutrition Examination Survey" OR nhanes))))

International Scan Survey

(PAGE 1)

The Applications of Artificial Intelligence to Human Biomonitoring

Privacy Notice

The personal information you provide to Health Canada will be collected by the National Biomonitoring Section under the *Department of Health Act* and handled in accordance with the *Privacy Act*.

Why are we collecting your personal information? This survey is part of a project to explore applications of artificial intelligence (AI) and machine learning (ML) in human biomonitoring of environmental chemicals. We will use your responses to help us identify current practices, challenges, and opportunities for integrating AI and ML methods into biomonitoring workflows. If you choose to provide your business contact information, we will use it solely to follow-up with you if we need more information.

Will we use or share your personal information for any other reason? We will use summarized information in a report which we will share with our stakeholders, we may also publish summary information in an article. We will not include your name or contact information in any reports or publications. We may include quotes in reports or publications, however any quotes will be chosen so that the risk you may be identified by your responses is very low. Separate from your completion of the survey, you may contact us at kavita.singh@hc-sc.gc.ca to request a copy of the final report. If you do so, we will only use this information to provide you with a copy of the report, once we have completed our analysis of the data. The contact information you provide for this purpose will not be connected in any way with your survey responses.

What are your rights? You have the right to access and request a correction and/or notation to your personal information. You also have a right to complain to the Privacy Commissioner of Canada if you feel your personal information has been handled improperly. For more information about these rights, or about how we handle your personal information, please contactbiomonitoring-biosurveillance@hc-sc.gc.ca.

For more information: The collection of your personal information is described in Info Source at infosource.gc.ca. Refer to the personal information banks (PIB) PSU 938 – Outreach Activitiesrelated to the collection of personal information via the survey, and PSU 914 – Public Communications related to the collection of your contact information for the purpose of sending you a copy of the final report.

Please select an option. (required)

- Yes, I would like to participate
- No, I do not want to participate

(PAGE 2)

Please note: When responding to free text questions, please do not include information that can be used to directly identify you or someone else.

Q1. Please select the option that best describes your program. (required)

- o National-level
- o Provincial-level
- o State-level
- o Other
- o Prefer not to answer

If participant selects "Other":

Please briefly describe your biomonitoring program or study. __________

Q2. What is your current role? (required)

- □ Principal Investigator
- □ Research Scientist
- □ Data Analyst
- □ Other
- □ Prefer not to answer

If participant selects "Other":

Please specify: __________

Q3. How long have you been involved in human biomonitoring? (required)

- o <1 year
- o 1-3 years
- o 3-5 years
- o >5 years
- o Prefer not to answer

Q4. In which areas of human biomonitoring do you primarily conduct research? (required)

- □ Environmental exposures
- □ Chemical risk assessment
- □ Epidemiology
- □ Toxicology

- □ Other
- □ Prefer not to answer

If participant selects "Other":

Please specify: __________

Q5. What types of data do you commonly use in your biomonitoring research? (check all that apply) (required)

- □ Chemical concentrations in blood, urine or other matrices
- □ Biomarker data (e.g., metabolomics, proteomics, genomics)
- □ Physiological or clinical endpoint data
- □ Demographic and lifestyle data
- □ Other
- □ Prefer not to answer

If participant selects "Other":

Please specify: __________

Q6. How would you describe the size and complexity of your biomonitoring datasets? (required)

- o Small and limited (e.g., <100 participants and <10 variables)
- o Moderate (e.g., 100–1,000 participants, 10–50 variables)
- o Large and complex (e.g., >1,000 participants, >50 variables)
- o Other
- o Prefer not to answer

If participant selects "Other":

Please describe: __________

Q7. How would you rate your familiarity with artificial intelligence or machine learning methodologies? (required)

- o None
- o Beginner
- o Intermediate
- o Advanced
- o Prefer not to answer

(PAGE 3)

Q8. Has your program implemented artificial intelligence or machine learning methods in the collection or analysis of biomonitoring data? (required)

- Yes
- No
- Prefer not to answer

Q9. Does your program plan to implement or expand the use of artificial intelligence or machine learning in biomonitoring over the next 1-2 years? (required)

- Yes
- No
- Unsure
- Prefer not to answer

Q10. Is your program currently exploring new artificial intelligence or machine learning technologies for biomonitoring that have not yet been implemented? (required)

- Yes
- No
- Prefer not to answer

If participant selects "Yes":

Please describe: __________

Q11. Is there a specific artificial intelligence or machine learning technology or approach you believe holds the most potential for improving biomonitoring in the future? (required)

- Yes
- No
- Unsure
- Prefer not to answer

If participant selects "Yes":

Please describe: __________

Q12. Are you collaborating with any external organizations for artificial intelligence or machine learning biomonitoring projects (e.g., research institutions, private companies)? (required)

- Yes
- No
- Prefer not to answer

If participant selects "Yes": **Please describe: __________**

Q13. Are there any external factors that might increase the urgency for adopting artificial intelligence or machine learning technologies in your program's biomonitoring efforts? (required)

- o Yes
- o No
- o Prefer not to answer

If participant selects "Yes":

Please describe: __________

Q14. Do you think artificial intelligence or machine learning technology has the potential to improve the future of biomonitoring practices? (required)

- o Yes
- o No
- o Unsure
- o Prefer not to answer

If participant selects "Yes":

Please describe: __________

Q15. What do you see as the primary barriers to implementing artificial intelligence or machine learning in your program's biomonitoring efforts? (select all that apply) (required)

- □ Lack of funding
- □ Lack of technical expertise
- □ Limited computational resources
- □ Lack of training opportunities
- □ Data limitations (e.g., poor quality, missing data, noise)
- □ Difficulty interpreting model outputs
- □ Organizational priorities
- □ Regulatory or ethical concerns
- □ Other
- □ Prefer not to answer

If participant selects "Other":

Please specify: __________

(PAGE 4)

You indicated that your program has implemented artificial intelligence or machine learning methods in the collection or analysis of biomonitoring data. The following questions ask about the methods you have implemented in your program.

Q16. Does your program have dedicated personnel or teams trained in artificial intelligence/machine learning methods for biomonitoring purposes? (required)

- o Yes
- o No
- o Prefer not to answer

Q17. What are your main objectives when applying artificial intelligence/machine learning methods? (check all that apply) (required)

- □ Pattern detection in exposure data
- □ Identification of exposure subgroups/population clusters
- □ Prediction of health outcomes or risk levels
- □ Interpretation of exposure-response relationships
- □ Integration of multi-omics data
- □ Other
- □ Prefer not to answer

If participant selects "Other":

Please specify: __________

Q18. Which artificial intelligence/machine learning methods have been implemented in your program? Briefly describe their primary applications (e.g., exposure modeling, exposure-outcome associations, etc.). If you do not want to provide a response, please indicate "prefer not to answer". (required)

Q19. What software frameworks or platforms do you use for artificial intelligence/machine learning analyses? (check all that apply) (required)

- □ Python libraries (e.g., scikit-learn, TensorFlow, PyTorch)
- □ R packages (e.g., caret)
- □ Commercial software (e.g., SAS, SPSS)
- □ Cloud platforms (e.g., AWS, Google Cloud ML, Azure ML)

- ◻ Other
- ◻ Prefer not to answer

If participant selects "Other":

Please specify: __________

Q20. What types of data do you use in the artificial intelligence/machine learning methods (select all that apply)? (required)

- ◻ Quantitative data
- ◻ Images
- ◻ Text
- ◻ Other
- ◻ Prefer not to answer

If participant selects "Other":

Please specify: __________

21. Are you integrating multiple data types (e.g., chemical exposures, omics data, clinical outcomes) into a single analysis? (required)

- o Yes, regularly
- o Yes, occasionally
- o Not currently, but plan to
- o No
- o Prefer not to answer

If participant selects options 1-3:

Please describe: __________

22. How significant are data cleaning, harmonization, and preprocessing efforts in your workflow? (required)

- o Minimal effort required
- o Moderate effort required (manageable with standard tools)
- o Significant effort required (a major challenge in most projects)
- o Unsure
- o Prefer not to answer

Q23. Are your artificial intelligence/machine learning applications capable of real-time analysis, prediction or decision-making? (required)

- o Yes
- o No
- o Prefer not to answer

If participant selects "Yes":

Please provide examples: __________

Q24. How often does your program review or update its artificial intelligence/machine learning models for biomonitoring to ensure they remain effective? (required)

- o Quarterly
- o Annually
- o As needed
- o We do not currently update AI/ML models
- o Other
- o Prefer not to answer

If participant selects "Other":

Please specify: __________

Q25. How does your program handle issues of data quality or gaps in data when using artificial intelligence/machine learning for biomonitoring? If you do not want to provide a response, please indicate "prefer not to answer". (required)

Q26. How do you ensure that ethical concerns, such as data privacy and the protection of sensitive data, are addressed in artificial intelligence/machine learning applications for biomonitoring? If you do not want to provide a response, please indicate "prefer not to answer". (required)

Q27. What challenges has your program faced in implementing artificial intelligence/machine learning for biomonitoring? If you do not want to provide a response, please indicate "prefer not to answer". (required)

Q28. Have you observed measurable improvements in outcomes (e.g., accuracy, efficiency) due to artificial intelligence/machine learning applications? (required)

- o Yes
- o No
- o Unsure
- o Prefer not to answer

If participant selects "Yes": **Please describe: __________**

Q29. What area(s) in your biomonitoring program have benefitted the most from the implementation of artificial intelligence/machine learning methods? (select all that apply) (required)

- □ Data collection
- □ Data management
- □ Data analysis
- □ Knowledge dissemination
- □ None
- □ Other
- □ Prefer not to answer

If participant selects "Other":

Please specify: __________

Q30. Please provide any citations or links to reports, case studies, and research that your program has produced that applies artificial intelligence/machine learning methods in biomonitoring? (required)

(PAGE 5)

Please provide any additional comments you would like to share with us.

Can we contact you for further information? (required)

- o Yes
- o No

If participant selects "Yes":

Full name: __________

Organization: __________

Name of your biomonitoring program or study: __________

E-mail (work): __________

Table S1. Categorization of ML Method

	Method	Paradigm		Task						Structure
		Supervised	Un-supervised	Regression	Classification	Clustering	Dimension Reduction	Feature Selection	Feature Importance	
1	ALE								☑	Model interpretation; Mathematical and Statistical
2	Adabag	☑		☑	☑					Tree-based; Ensemble
3	Adaboost	☑		☑	☑				☑	Tree-based; Ensemble
4	Bagging	☑		☑	☑					Tree-based; Ensemble
5	BART	☑		☑	☑			☑	☑	Tree- and kernel-based; Ensemble
6	BKMR	☑		☑				☑	☑	Kernel-based; Bayesian
7	BNN	☑		☑	☑					Neural network; Bayesian
8	Boruta algorithm	☑		☑	☑			☑	☑	Tree-based
9	CDA	☑			☑		☑	☑	☑	Linear
10	CatBoost	☑		☑	☑			☑	☑	Tree-based; Ensemble
11	Causal survival forest	☑		☑					☑	Tree-based; Ensemble
12	CART	☑		☑	☑			☑	☑	Tree-based
13	CLV		☑			☑	☑	☑		Clustering
14	DBSCAN		☑			☑				Clustering; Density-based
15	Decision stump	☑		☑	☑			☑		Tree-based
16	Decision table	☑			☑					Rule-based
17	Decision tree	☑		☑	☑			☑	☑	Tree-based
18	Discriminant analysis	☑			☑		☑	☑	☑	Linear; Bayesian
19	ENET	☑		☑	☑			☑	☑	Linear
20	EMMA		☑				☑			Mathematical and Statistical
21	Ensemble regressor chains	☑		☑						Meta-learning; Ensemble
22	EBM	☑		☑	☑			☑	☑	Additive model; Ensemble
23	EFA		☑				☑	☑	☑	Dimensionality reduction
24	ExtraTrees/ ExtraSurvival Trees	☑		☑	☑			☑	☑	Tree-based; Ensemble
25	Gaussian process	☑		☑	☑				☑	Bayesian
26	Genetic programming	☑		☑	☑			☑		Mathematical and Statistical; Evolutionary
27	glmBoost	☑		☑	☑			☑		Linear; Ensemble
28	Gradient boosting machine	☑		☑	☑			☑	☑	Tree-based; Ensemble
29	GRRF	☑		☑	☑		☑	☑	☑	Tree-based; Ensemble
30	Hierarchical clustering		☑			☑				Clustering

31	Huber regressor (HUBER)	☑		☑					☑	Linear
32	Independent component analysis		☑				☑	☑	☑	Mathematical and Statistical
33	J48 (C4.5)	☑			☑			☑	☑	Tree-based
34	k-Means clustering		☑			☑				Clustering
35	k-Medoid clustering		☑			☑				Clustering
36	k-Nearest neighbours	☑		☑	☑					Instance-based
37	Latent class/profile analysis		☑			☑				Clustering
38	LASSO	☑		☑				☑	☑	Linear
39	Least angle regression	☑		☑				☑	☑	Linear
40	LightGBM	☑		☑	☑			☑	☑	Tree-based; Ensemble
41	Linear discriminant analysis	☑		☑	☑		☑	☑	☑	Linear
42	LIME								☑	Model Interpretation
43	Logistic regression	☑			☑					Linear
44	LUCID		☑			☑	☑	☑	☑	Clustering; Bayesian
45	Markov blanket		☑				☑	☑	☑	Graphical
46	Meridian lossless packing						☑			Audio compression
47	Mixed graphical model		☑			☑		☑	☑	Graphical
48	MARS	☑		☑	☑			☑	☑	Spline-based
49	Multiple linear regression	☑		☑						Linear
50	Naive Bayes/Gaussian Naïve Bayes	☑			☑				☑	Bayesian
51	Neural network	☑	☑	☑	☑		☑		☑	Neural Network
52	Non-negative matrix factorization		☑			☑	☑	☑		Mathematical and Statistical
53	Orthogonal matching pursuit	☑		☑			☑	☑		Mathematical and Statistical
54	PLS-GLM	☑		☑	☑		☑	☑	☑	Linear
55	PLS-DA/OPLS-DA	☑			☑		☑	☑	☑	Linear
56	Passive aggressive regressor	☑		☑						Linear; Online Learning
57	Principal component analyses		☑				☑		☑	Dimensionality reduction
58	Principal component pursuit		☑				☑			Mathematical and Statistical

59	Quantile based G-computation	☑		☑					☑	Mathematical and Statistical
60	Radial basis function kernel	☑	☑	☑	☑	☑	☑			Kernel-based
61	Random forest	☑		☑	☑			☑	☑	Tree-based; Ensemble
62	Random linear combinations		☑			☑	☑			Linear
63	Random tree	☑		☑	☑			☑	☑	Tree-based
64	Redescription mining		☑			☑		☑		Clustering
65	Regression tree	☑		☑				☑	☑	Tree-based
66	Ridge regression/ classifier	☑		☑	☑		☑		☑	Linear
67	RReliefF variable selection algorithm	☑		☑	☑			☑	☑	Mathematical and Statistical
68	Select K best algorithm	☑		☑	☑		☑	☑	☑	Mathematical and Statistical
69	SHAP							☑	☑	Model interpretation
70	sPLS	☑		☑	☑		☑	☑	☑	Linear
71	Stacked single-target methods	☑		☑	☑					Meta-learning; Ensemble
72	Stepwise generalized linear model	☑		☑	☑			☑	☑	Mathematical and Statistical
73	Stochastic gradient boosting	☑		☑	☑			☑	☑	Tree-based; Ensemble
74	Stochastic gradient descent	☑		☑	☑					Linear
75	Super learner	☑		☑	☑					Meta-learning; Ensemble
76	Support vector machine	☑		☑	☑				☑	Kernel-based
77	TMLE	☑		☑						Mathematical and Statistical
78	Tree bag	☑		☑	☑			☑	☑	Tree-based; Ensemble
79	Voted perceptron	☑			☑					Linear; Ensemble
80	Voting classifier	☑			☑					Meta-learning; Ensemble
81	WQSR	☑		☑				☑	☑	Mathematical and Statistical
82	XGBoost	☑		☑	☑			☑	☑	Tree-based; Ensemble

Abbreviations: Adabag = adaptive bagging; Adaboost = adaptive boosting; ALE = accumulated local effects; BART = Bayesian additive regression tree; BKMR = Bayesian kernel machine regression; BNN = Bayesian neural network; CART = classification and regression tree; CatBoost = categorical boosting; CDA = canonical discriminant analysis; CLV = clustering of variables around latent variables; DBSCAN = density-based spatial clustering of applications with noise; EBM = explainable boosting machine; EFA = exploratory factor analysis; EMMA = end-member modelling analysis; ENET = elastic net; GLM = generalized linear model; GRRF = guided regularized random forest; LASSO = least absolute shrinkage and selection operator; LightGBM = light gradient boosting machine; LIME = local interpretable model-agnostic explanations; LUCID = latent unknown clustering with integrated data; MARS = multivariate adaptive regression splines; OPLS-DA = orthogonal partial least square-discriminant analysis; PLS-DA = partial least squares discriminant analysis; PLS-GLM = partial least squares generalized linear model; SHAP = Shapley additive explanation; sPLS = sparse partial least squares; TMLE = targeted maximum likelihood/minimum loss estimation; WQSR = weighted quantile sum regression; XGBoost = extreme gradient boosting

Studies by Biomonitoring Theme

Table S2. Description – Chemicals

- Question a: What chemicals are people exposed and at what levels?
- Question b: Are there trends in chemical concentration levels over time?

References	Brief Description	Methods
1. Buck Louis (2019)[1]	Endocrine disrupting chemicals and temporal patterns during pregnancy	HC (with linear mixed-effects model)
2. Stanfield (2024)[2]	Trends for metabolites and parent compounds in urine	HC & UC

Abbreviations: HC = hierarchical clustering; UC = unsupervised clustering

Table S3. Description – Exposure Factors

- Question a: How do chemical concentration levels vary by demographic and lifestyle factors?
- Question b: What are the characteristics of subgroups that are highly exposed to chemicals?
- Question c: What are the characteristics of subgroups that are close to the limits of detection (i.e. less exposed to chemicals)?

References	Brief Description	Methods[1]
1. Kalloo (2018)[3]	Grouping of participants by similar chemical exposure profiles	k-Means & PCA
2. Carroll (2020)[4]	Grouping of participants by similar chemical exposures	k-Means & LCA
3. Fu (2022)[5]	Identification of participant characteristics with similar chemical exposure patterns	k-Medoid
4. Sen (2022)[6]	Effect of demographic and clinical factors on chemical concentrations	Factor analysis[2]
5. Yonkman (2023)[7]	Grouping of participants by similar chemical profiles	k-Means & LPA
6. Liu (2024)[8]	Grouping of participants into high and low metal clusters and difference in characteristics	k-Medoid
7. Wang (2024)[9]	Predicting e-waste workers and non-workers from volatile organic compounds	DT, GBM, PCA, RF & SVM
8. Moro (2025)[10]	Prediction of occupation (gas attendants, taxi drivers and farmers) based on titanium and other biomarkers of chemical exposures	DT, kNN, RF & SVM
9. Zhou (2025)[11]	Characteristics of participants by clusters	SOM

[1]The ML methods for dimensionality reduction/clustering (i.e., k-Means, k-Medoid, PCA, LCA, LPA and SOM) were used to group participants into clusters, and differences in participant characteristics between clusters were identified through descriptive statistics.
[2]Use of 'Scater' package in R.

Abbreviations: DT = decision tree; GBM = gradient boosting machine; HC = hierarchical clustering; kNN = k-nearest neighbours; LCA = latent class analysis; LPA = latent profile analysis; PAH = polycyclic aromatic hydrocarbon; RF = random forest; SOM = self-organizing maps; SVM = support vector machine; UC = unsupervised clustering

Table S4. Description – Mixtures

- Question a: Can chemical clusters be identified (i.e. chemicals that co-exist) in the population?
- Question b: What are the most prevalent chemicals or chemical combinations in mixtures?

References	Brief Description	Methods
1. Chiu (2018)[12]	Components based on 8 phthalates	PCA
2. Hendryx (2018)[13]	Chemical composition of classes based on 7 PAHs	LCA
3. Kalloo (2018)[3]	Components based on multiple chemical classes	k-Means & PCA
4. Gibson (2019)[14]	Components/factors based on 18 persistent organic pollutants	HC, EFA, k-Means & PCA
5. Carroll (2020)[4]	Chemical composition of clusters	k-Means & LCA
6. Matta (2020)[15]	Clustering and components of multiple chemicals	CLV & PCA
7. Tao (2021)[16]	Component based on 14 metal species	PCA
8. Fu (2022)[5]	Clusters within 7 personal care and consumer product chemicals	k-Medoid
9. Grau-Perez (2022)[17]	Components based on 9 metals	HC & PCA
10. Sen (2022)[6]	Clusters based on 4 per and poly-fluoroalkyl substances, methylparaben and bisphenol A	Model-based clustering
11. Babic Leko (2023)[18]	Components based on metals and non-metal elements	PCA
12. Yonkman (2023)[7]	Chemicals composition of clusters	k-Means & LPA
13. Yu (2023)[19]	Components based on 11 phthalates	PCA
14. Stanfield (2024)[2]	Chemical clusters and components within clusters	HC & UC
15. Wang (2024)[20]	Clustering and components of semi-volatile organic compound exposures	CLV & PCA
16. Wu (2024)[21]	Pattern identification among 43 chemicals	PCP
17. Zang (2024)[22]	Chemical composition of components and clusters based on 9 polycyclic aromatic hydrocarbons	k-Means & PCA
18. Zhang (2024)[23]	Components based on 11 phthalates	PCA
19. Chi (2025)[24]	Loading scores of metals/metalloids on each principal component	PCA
20. Lai (2025)[25]	Patterns of per and poly-fluoroalkyl substances by region	EMMA
21. Zhou (2025)[11]	Clustering of 9 phenols	SOM

Abbreviations: CLV = clustering of variables around latent variables; EFA = exploratory factor analysis; EMMA = end-member modelling analysis; HC = hierarchical clustering; LCA = latent class analysis; LPA = latent profile analysis; PCA = principal component analysis; PCP = principal component pursuit; SOM = self-organizing maps; UC = unsupervised clustering;

Table S5. Association/Prediction – Chemicals (from exposure factors)

- Question: Can sociodemographic and lifestyle factors predict what chemicals are likely to be present in an individual?

References	Brief Description	Methods
1. Jovanovic (2019)[26]	Persistent organic pollutants in breast milk – from mother age and parity	GRRF (Ensemble method)
2. Potash (2020)[27]	Elevated blood lead levels in children – from housing, census-level variables, insurance, income and other factors	LR & RF
3. Liu (2021)[28]	Blood lead in children – from environmental and sociodemographic factors	ExtraTrees, GBM, GLM, NN, RF & XGBoost
4. Nishihama (2021)[29]	Organophosphate pesticides in pregnant women – from various factors, such as income, diet and smoking status	GBM, MLR, NN & RF
5. Mulhern (2022)[30]	Elevated blood lead levels in children – from sociodemographic, housing and water system factors	BN, Markov blanket & NB
6. Abbasi (2023)[31]	Blood lead in children – from census block socioeconomic and housing factors, and distance from lead emissions/roadways	ENET, LASSO (regularized LR) & RF
7. Chaurasia (2023)[32]	Blood lead in pregnant women – from sociodemographic factors	AB, BA, DT, kNN, NB, NN & SVM
8. Sambanis (2023)[33]	Blood lead in children – from housing, behavioural and sociodemographic factors	DT & k-Means
9. Shin (2023)[34]	Bisphenol A in children - from modifiable factors	LASSO
10. Wang (2023)[35]	Bone lead level prediction model – from various factors, such as age, education and occupation	BA, DT, ENET, LASSO, RF, RR, SL & XGBoost
11. Aker (2024)[36]	PFAS in plasma – from food items	ENET & HC
12. Frndak (2024)[37]	Blood lead in children – from individual and neighbourhood-level features	ENET (regularized GLM), Ensemble, GBM, NN & RF
13. Jeong (2024)[38]	Metals – from nutrient intake patterns	kNN
14. Keil (2024)[39]	Prediction of PCBs, dioxins and furans in serum given other congeners and confounders, such as age, body mass index, race and education	SL
15. Mahfouz (2024)[40]	Persistent organic pollutants in pregnant women – from sociodemographic, dietary and other factors	NN, RF, SHAP, SVM & XGBoost
16. Suwannarin (2024)[41]	Phenols in pregnant women – from sociodemographic, lifestyle and dietary factors	GBM, MLR, NN, RF & XGBoost
17. Sy (2024)[42]	Cadmium and lead in children – from age and co-exposures	Ensemble, Random linear combinations & Stacked single-target methods
18. Tao (2024)[43]	PFAS in pregnant women – from diet and fatty acids	LASSO, LightGBM, MLR, RF, RR & XGBoost
19. Yang (2024)[44]	Polychlorinated napthalenes in breast milk- from emissions, diet, and chemical properties	AB, DT, GBM, kNN, Linear regression, RF, RR, SHAP, SVM & XGBoost

20. Chaurasia (2025)[45]	Blood lead in pregnant women – from various factors such as age, education, occupation and water source	BA, DT, kNN, LIME, NN, RF & SHAP
21. Chou (2025)[46]	Bisphenol A and phthalates in urine of neonates undergoing cardiac surgery – from type and intensity/duration of medical care	Time-series HC
22. Du (2025)[47]	Organophosphate flame retardants in serum – from factors such as age, education, occupation and diet	ENET, LASSO, RF, RR, SHAP, SVM & XGBoost
23. Jung (2025)[48]	Metals in pregnant women – from diet, lifestyle, health and disease history and indoor/outdoor environment	MLR, RF, SHAP & XGBoost
24. Lai (2025)[25]	PFAS in plasma of pregnant women – from more than 200 determinants, including parity, age and diet	ALE, GBM, LASSO, LightGBM, MLR, NN, RF, SHAP & XGBoost
25. Okati (2025)[49]	Hair mercury levels – from demographics, occupation, diet and dental amalgams	DT, Genetic programming, kNN, LASSO, MARS, MLR, RF & XGBoost
26. Qu (2025)[50]	p-Phenylenediamine antioxidants – from factors such as age, sex, body mass index and occupation	ENET, LightGBM, Linear regression, NN, RF, RR, SHAP, SVM & XGBoost

Abbreviations: AB = adaptive boosting; ALE = accumulated local effects; BA = Boruta algorithm; BN = Bayesian network; DT = decision tree; ENET = elastic net; GBM = gradient boosting machine; GLM = generalized linear model; GRRF = guided regularized random forest; HC = hierarchical clustering; kNN = k-nearest neighbours; LASSO = least absolute shrinkage and selection operator; LightGBM = light gradient boosting machine; LIME = local interpretable model-agnostic explanations; LR = logistic regression; MARS = multivariate adaptive regression splines; MLR = multiple linear regression; NB = Naïve Bayes; NN = neural network; PFAS = per- and polyfluoroalkyl substances; RF = random forest; RR = ridge regression; SHAP = Shapley additive explanation; SL = super learner; SVM = support vector machine; XGBoost = extreme gradient boosting machine

Table S6. Association/Prediction – Chemicals (from biomarkers of exposure)

- Question: Can biomarkers of exposure predict chemical exposures?

References	Brief Description	Methods
1. Fry (2007)[51]	Prenatal arsenic exposure – gene expression biomarkers	SVM
2. Demchenko (2020)[52]	Organochlorine pesticides in children – predicted from mothers' levels	NN
3. Levin-Schwartz (2020)[53]	Creation of a multi-media biomarker for blood lead, based on lead in blood, urine, hair and nails	ICA, NMF & WQSR
4. Gerbi (2022)[54]	Maternal blood lead level – predicted from lead in child tooth dentine	LASSO, SL & XGBoost
5. Jala (2023)[55]	Phenol exposures – urinary metabolite biomarkers	HC, LR, PCA, PLS-DA, RF & SVM
6. Khanam (2023)[56]	Arsenic exposures – urinary metabolite biomarkers	RF
7. Midya (2023)[57]	Blood lead in pregnant women – predicted from gut microbial cliques in children	LR & RF[1]
8. Zhang (2024)[58]	Metals/metalloids in follicular (oocyte) fluid – predicted from serum concentrations and other factors	ENET, HC, RF, SVM & XGBoost
9. Zhao (2024)[59]	Prenatal arsenic exposure – DEGs and immune cell biomarkers	LASSO & SVM
10. Khodasevich (2025)[60]	Polybrominated biphenyls - epigenetic clocks as biomarkers of exposure	ENET, PCA & SL
11. Sung (2025)[61]	Pesticide urinary levels in patients with kidney function decline – metabolomic biomarker	OPLS-DA, PCA & PLS-DA
12. Zhang (2025)[62]	Semi-volatile organic compounds in serum – predicted from levels in air and other molecular descriptors	AB, DT, ExtraTrees, Linear regression, RF, SVM & XGBoost

[1] RF was used to discover gut microbial cliques that were predictive of prenatal lead. A randomization-based inference model and logistic regression were then applied to restore directionality and estimate association between lead exposure and abundance of the gut microbial cliques.

Abbreviations: AB = adaptive boosting; DEGs = differentially expressed genes; DT = decision tree; ENET = elastic net; HC = hierarchical clustering; ICA = independent component analysis; LASSO = least absolute shrinkage and selection operator; LR = logistic regression; NMF = non-negative matrix factorization; NN = neural network; OPLS-DA = orthogonal projections to latent structures discriminant analysis; PCA = principal component analysis; PLS-DA = partial least squares discriminant analysis; RF = random forest; SL = super learner; SVM = support vector machine; WQSR = weighted quantile sum regression; XGBoost = extreme gradient boosting machine

Table S7. Association/Prediction – Health-Based Outcomes (from individual or multiple chemicals)

- Question: Is there an association between a chemical and a health-related outcome? OR Can exposure to a chemical predict a health-related outcome?

References	Brief Description	Methods
**Metals/Metalloids**		
1. Tan (2009)[63]	Metals/metalloids and cardiovascular disease	AB & PCA
2. Golasik (2015)[64]	Metals/metalloids and laryngeal cancer	CDA, DT, LR, NN, PCA & SVM
3. Zabinski (2016)[65]	Arsenic and lower birthweight for gestational age	BN
4. De Benedetti (2017)[66]	Metals/metalloids and amyotrophic lateral sclerosis/DNA damage	NN
5. Lin (2017)[67]	Metals/metalloids and schizophrenia	AB, LR, NB, NN, PCA, RF & SVM
6. Lin (2017)[68]	Metals/metalloids and esophageal squamous cell carcinoma	AB, LR, NB, NN, PCA, RF & SVM
7. Park (2019)[69]	Metals/metalloids and hypercholesterolemia	DT, kNN, LR, RF & SVM
8. Cox (2020)[70]	Lead and mortality	BN & LR
9. Kim (2021)[71]	Lead and osteoarthritis	DT, kNN, LR, RF & SVM
10. Monaco (2021)[72]	Metals/metalloids and cardiovascular disease	BA, GLM, NN & RF
11. Ximenez (2021)[73]	Metals/metalloids and bone mineral density	RF & SVM
12. Liu (2022)[74]	Metals/metalloids and autism	LASSO
13. Xia (2022)[75]	Metals/metalloids and osteoarthritis	DT, GBM, kNN, MARS; NN, RF, SVM & XGBoost
14. Xia (2022)[76]	Metals/metalloids and depression	DT, GBM, kNN, MARS, NN, RF, SVM & XGBoost
15. Babic Leko (2023)[18]	Metals/metalloids and Alzheimer's disease	PCA (with linear regression), RF & RM
16. Chan (2023)[77]	Metals/metalloids and gestational diabetes mellitus	Discriminant analysis, Ensemble, kNN, NB & SVM
17. Chen (2023)[78]	Metals/metalloids and thyroid cancer	DT, GP, kNN, NN, RF, SHAP, SVM & XGBoost
18. Chen (2023)[79]	Metals/metalloids and hyperuricemia	Explainable boosting machine, LightGBM, NB, NN, RF, SHAP & XGBoost
19. Li (2023)[80]	Metals/metalloids and hypertension	AB, DT, Ensemble, GBM, kNN, LASSO, NN, RF, RR, SHAP & SVM
20. Li (2023)[81]	Metals/metalloids and coronary heart disease	AB, DT, kNN, RF, SHAP, SKB & SVM

21. Li (2023)[82]	Lead and prostate cancer risk	LASSO, LR, RF, SHAP & XGBoost
22. Ling (2023)[83]	Metals/metalloids and autism	PCA, PLS-DA/OPLS-DA & SVM
23. Luo (2023)[84]	Metals/metalloids and kidney function/tumour necrosis factor-α	CatBoost, DT, GBM, kNN, NB, SHAP, SVM & XGBoost
24. Mei (2023)[85]	Metals/metalloids and hyperuricemia	ENET
25. Moon (2023)[86]	Cadmium and cardiovascular/cancer/all-cause mortality	RF
26. Souza (2023)[87]	Metals/PAHs and oxidative stress	AB, DT, ENET, ExtraTrees, GBM, Huber regressor, kNN, LASSO, Least angle regression, LightGBM, Orthogonal matching pursuit, Passive aggressive regressor, RF & RR
27. Wen (2023)[88]	Metals/metalloids and blood eosinophil counts	XGBoost
28. Wu (2023)[89]	Metals/metalloids and chronic kidney disease	XGBoost & SHAP
29. Zhao (2023)[90]	Metals/metalloids and diabetes mellitus	Ensemble, LightGBM, RF & XGBoost
30. Angali (2024)[91]	Metals/metalloids and cardiovascular disease	DT, GBM, kNN, LDA, LR, NB, NN, SHAP & SVM
31. Bowles (2024)[92]	Metals/metalloids and cardiovascular disease	BN & Mixed graphical mode
32. Chang (2024)[93]	Cadmium and metabolome	RF & SVM
33. Chen (2024)[94]	Metals/metalloids and chronic kidney disease	CatBoost, LightGBM, NB, NN, RF, SHAP & XGBoost
34. Du (2024)[95]	Metals/metalloids and blood lipids	ENET
35. Fan (2024)[96]	Metals/metalloids and osteoarthritis/rheumatoid arthritis	CatBoost, ExtraTrees, kNN, LASSO, LightGBM, NN, RF, SHAP & XGBoost
36. Fansler (2024)[97]	Metals/metalloids and mortality	ENET (penalized Cox) & RF
37. Gao (2024)[98]	Metals/metalloids and age-related macular degeneration	DT, GBM, GP, kNN, LR, NB, NN, RF, SHAP, SVM & XGBoost
38. Gui (2024)[99]	Metals/metalloids and diabetic retinopathy	DT, GBM, GP, kNN, LR, NB, NN, RF, SHAP, SVM & XGBoost
39. Invernizzi (2024)[100]	Metals/metalloids and resting state fMRI	SHAP & XGBoost
40. Jeong (2024)[38]	Metals/metalloids and metabolic syndrome	DT, kNN, LightGBM & RF
41. Li (2024)[101]	Metals/metalloids and cardiovascular disease	RF
42. Liu (2024)[102]	Metals/metalloids and insulin resistance	AB, DT, Ensemble, GBM, kNN, LASSO, LR, NB, NN, RF, RR, SHAP, SVM & XGBoost
43. Liu (2024)[103]	Metals/metalloids and cognitive function	ENET & LR

44. Midya (2024)[104]	Metal-microbial cliques and intestinal inflammation	RF[1]
45. Midya (2024)[105]	Metal-microbial cliques and depression	RF (with linear regression)[1]
46. Nabavi (2024)[106]	Metals/metalloids and cognitive function	CatBoost, DBSCAN, Ensemble, GBM, NN, RF, SHAP & SVM
47. Rog (2024)[107]	Metals/metalloids and bipolar disorder	DT
48. Su (2024)[108]	Chromium and genetic damage/immune parameters	EFA
49. Wang (2024)[109]	Metals/metalloids and pulmonary function	CatBoost, ExtraTrees, kNN, LightGBM, NN, RF, SHAP & XGBoost
50. Wu (2024)[110]	Metals/metalloids and chronic kidney disease	GP, LASSO & SVM
51. Yan (2024)[111]	Metals/metalloids and glioma	LASSO, NB, NN, RF & SVM
52. Yao (2024)[112]	Metals/metalloids and metabolic syndrome	AB, kNN, LASSO, LR, NB, NN, RF, SHAP, SVM & XGBoost
53. Xiao (2024)[113]	Metals/metalloids and osteoporosis	AB, CatBoost, DT, GBM, kNN, LightGBM, LR, NB, NN, RF, SHAP, SVM & XGBoost
54. Xu (2024)[114]	Metals/metalloids and hypertension	DT, LIME, NB, NN, PCA, SHAP, SKB, SVM & XGBoost
55. Xu (2024)[115]	Metals/metalloids and mortality	DT, LIME, NB, NN, PCA, SHAP, SKB, SVM & XGBoost
56. Zhao (2024)[116]	Metals/metalloids and diabetic kidney disease	ENET, GBM, LASSO, LDA, NB, PLS-GLM, RF, RR, SVM & XGBoost
57. Zibibula (2024)[117]	Metals/metalloids and ischemic stroke	DT, kNN, LightGBM, LR, RF, SVM & XGBoost
58. Zuo (2024)[118], [119]	Cadmium and stroke	DT, kNN, LR, NN, RF & SHAP
59. Chen (2025)[120]	Metals/metalloids and alveolar bone loss	DT, kNN, LASSO, LR, RF, SHAP, SVM & XGBoost
60. Chen (2025)[121]	Metals/metalloids and blood pressure	kNN, LightGBM, RF, SHAP & XGBoost
61. Cox Jr. (2025)[122]	Lead and mortality	Causal survival forest, DT (survival tree) & RF
62. Gu (2025)[123]	Metals/metalloids and infertility	AB, BA, DT, GBM, LightGBM, LR, RF & SHAP
63. He (2025)[124]	Metals/metalloids and diabetes mellitus	AB, Ensemble, GBM, kNN, LASSO, NN, RF, RR, SHAP, SVM & XGBoost
64. Hu (2025)[125]	Metals/metalloids and human papilloma virus infection	LASSO, SHAP & XGBoost
65. Jin (2025)[126]	Metals/metalloids and mortality	CoxPH, ExtraSurvival Trees, GBM, k-Means, RF, SHAP & SVM

66. Jin (2025)[127]	Metals/metalloids and sarcopenia	Clustering (10 algorithms), ENET, GBM, LASSO, LDA, NB, PLS-GLM, RC, RF, StepGLM, SVM & XGBoost
67. Johnson (2025)[128]	Metals/metalloids and cognitive function	PCA & RF
68. Li (2025)[129]	Metals/metalloids and major adverse cardiovascular events	AB, DT, GBM, LightGBM, LR, NN, RF, SHAP & XGBoost
69. Li (2025)[130]	Metals/metalloids and periodontitis	DT, kNN, LightGBM, NN, RF, SHAP, SVM & XGBoost
70. Mi (2025)[131]	Metals/metalloids and hearing loss	DT, GBM, kNN, LR, NN, RF, SVM & XGBoost
71. Nabavi (2025)[132]	Metals/metalloids and hearing loss	CatBoost, GBM, LR, NN, RF, SHAP & XGBoost
72. Nahar (2025)[133]	Lead and chronic heart disease	DT, kNN, LR, RF, SVM & XGBoost
73. Ren (2025)[134]	Metals/metalloids and cognitive function	kNN, LASSO, NB, RF, SHAP, SVM & XGBoost
74. Shen (2025)[135]	Metals/metalloids and cardiovascular disease	AB, DT, GBM, kNN, LASSO, RF, SHAP & SVM
75. Shi (2025)[136]	Metals/metalloids and digestive tract cancer	RF & SHAP
76. Wan (2025)[137]	Metals/metalloids and anxiety	LASSO, LR & XGBoost
77. Wang (2025)[138]	Metals/metalloids and glaucoma	DT, GBM, GP, kNN, LIME, LR, NB, NN, RF, SHAP, SVM & XGBoost
78. Wen (2025)[139]	Metals/metalloids and chronic cough	DT, RF, SVM & XGBoost
79. Wu (2025)[140]	Metals/metalloids and gallstone disease	DT, GBM, LASSO, LR, NB, RF, RR, SHAP, SVM & XGBoost
80. Xia (2025)[141]	Metals/metalloids and chronic bronchitis	AB, BA, CatBoost, kNN, LightGBM, LR, NB, NN, RF, SHAP, SVM & XGBoost
81. Xu (2025)[142]	Metals/metalloids and depression	DT, LIME, NB, NN, PCA, SHAP, SKB, SVM & XGBoost
82. You (2025)[143]	Metals/metalloids and prostate cancer	CatBoost, LASSO, LightGBM, LR, NB, NN, RF, SHAP, SVM & XGBoost
83. Yuting (2025)[144]	Metals/metalloids and heart failure	GBM, kNN, RF, SHAP, SKB, SVM & XGBoost
84. Zhang (2025)[145]	Metals/metalloids and sarcopenia	BA, CatBoost, DT, GBM, LASSO, LightGBM, LR, RF & SHAP
85. Zhang (2025)[146]	Metals/metalloids and coronary artery disease	DT, kNN, LR, NB RF & XGBoost

86. Zhong (2025)[147]	Metals/metalloids and gestational anemia	AB, DT, GBM, NB, RF, SVM & XGBoost
**Per- and Polyfluoroalkyl Substances**		
87. Feng (2023)[148]	PFAS/elements and spontaneous preterm birth	ENET, LASSO, RF & XGBoost
88. Eve (2024)[149]	PFAS and cardiovascular disease	BA, DT, LR, PCA, RF & SVM
89. Li (2025)[150]	PFAS and rheumatoid arthritis	AB, CatBoost, DT, Ensemble, ExtraTrees, GBM, kNN, LightGBM, NN, RF, SHAP, SVM & XGBoost
90. Shao (2025)[151]	PFAS and COPD	CatBoost, DT, Ensemble, kNN, LightGBM, NN, RF, SHAP, SVM & XGBoost
91. Wang (2025)[152]	PFAS and thyroid cancer and metabolome	AB, DT, ENET, ExtraTrees, GBM, kNN, LASSO, LDA/QDA, LightGBM, LR, LUCID, NB, PCA, RF, SHAP & sPLS
92. Wang (2025)[153]	PFAS and diabetes mellitus	AB, CatBoost, DT, Ensemble, ExtraTrees, GBM, kNN, LightGBM, NN, RF, SHAP, SVM & XGBoost
93. Yang (2025)[154]	PFAS and abdominal aortic calcification	XGBoost & SHAP
94. Zheng (2025)[155]	PFAS and cardiovascular-kidney-metabolic syndrome	DT, kNN, LASSO, LightGBM, LR, NN, RF, SHAP, SVM & XGBoost
**Phthalates**		
95. Colicino (2021)[156]	Phthalates and behaviour problems	LASSO
96. Lu (2023)[157]	Phthalates/PAHs and semen quality	AB, GBM, NN, RF, SVM & XGBoost
97. You (2024)[158]	Phthalates and hearing loss	LR, RF & XGBoost
98. Zhang (2024)[23]	Phthalates and sex steroid hormones/obesity	DT, GBM, kNN, NB, PCA (with LR), RF, SHAP, SVM & XGBoost
99. Liu (2025)[159]	Phthalates and breast cancer	DT, GBM, kNN, LR, Meridian lossless packing, NB, RF, SHAP & SVM
100. Wu (2025)[160]	Phthalates and multiple diseases	BN, NN & XGBoost
**Pesticides and Other Persistent Organic Pollutants**		
101. Gibson (2019)[14]	PCBs/dioxins/furans and leukocyte telomere length	EFA, ENET, HC, k-Means, LASSO & PCA

102. Matta (2020)[15]	Persistent organic pollutants and deep endometriosis	AB, CLV, ENET (regularized LR), NN, PCA, PLS-DA, RR (regularized LR) & SVM
103. Oh (2022)[161]	$TCDD_{eq}$ and prediabetes/diabetes	BN
104. Sharma (2021, 2023)[162], [163]	Organochlorine pesticides and breast cancer	AB, BN, Decision table, DT, Ensemble, LR, NB, NN, RF & SVM
105. Gao (2024)[164]	PCBs and hyperuricemia, hypertension, diabetes, chronic kidney disease, arthritis and mortality	DT, Ensemble, GBM, NB, NN, PCA, RF, SGD, SVM & WQSR
106. Liu (2024)[165]	Pesticides and cancer	DT, GBM, kNN, LR, Meridian lossless packing, NB, Radial basis function kernel, RF & SHAP
107. Tan (2024)[166]	Organochlorine pesticides and chronic kidney disease/diabetes/hypertension/inflammation/mortality	AB, ExtraTrees, LightGBM, NN & RF
108. Jiang (2025)[167]	Organophosphorus pesticides and age-related macular degeneration	ALE, DT, GBM, GP, kNN, LIME, LR, NB, NN, RF, SHAP, SVM & XGBoost
109. Liu (2025)[168]	Pesticides and age-related macular degeneration	DT, ENET, kNN, LightGBM, LR, NN, RF, SHAP, SVM & XGBoost
110. Pan (2025)[169]	Neonicotinoids and gestational diabetes mellitus	SHAP & XGBoost
111. Shamma (2025)[170]	Organochlorine pesticides and thyroid hormones	GBM, LASSO (regularized LR), RF, SVM & XGBoost
112. Wang (2025)[171]	Organophosphate pesticides and triglyceride glucose index	LR, RF, SHAP, SVM & XGBoost
113. Yang (2025)[172]	Pesticides and gestational diabetes mellitus	ENET, G-computation models & RF
**Volatile Organic Compounds**		
114. Fu (2024)[173]	Volatile organic compounds and cardiovascular disease	BA, DT, LightGBM, NN, RF, SHAP, SVM & XGBoost
115. Liu (2024)[174]	Volatile organic compounds and COPD	LR, NB, DT, RF, SVM & XGBoost
116. Wang (2024)[20]	Semi-volatile organic compounds and thyroid nodule	AB, CLV, ENET (regularized LR), GBM, NN, PCA, RF, SVM & XGBoost
117. Deng (2025)[175]	Volatile organic compounds and preserved ratio impaired spirometry	AB, CatBoost, ExtraTrees, GBM, LASSO, LDA, LightGBM, LR, NN, RF, SHAP & XGBoost
118. Jiang (2025)[176]	Volatile organic compounds and chronic kidney disease	kNN, LASSO, LIME, LR, NN, RF, SHAP & XGBoost

119. Zhang (2025)[177]	Volatile organic compounds and chronic kidney disease	LASSO, RF, SHAP, SVM & XGBoost
**Other**		
120. Chen (2022)[178]	Aldehydes and kidney stones	XGBoost
121. Liu (2024)[179]	Phenols and kidney function	AB, DT, kNN, RF, SHAP & SVM
122. Pala (2024)[180]	Environmental exposure indicators of exposome[2] and difference between phenotypical and chronological ages	DT, ENET, GBM, k-means, kNN, LASSO, LightGBM, RF, RR, SVM & XGBoost
123. Cai (2025)[181]	Brominated flame retardants and hyperuricemia	DT, ENET, kNN, LightGBM, LR, NN, RF, SHAP SVM & XGBoost
124. Mulisa (2025)[182]	Mycotoxins and esophageal cancer	AB, DT, ExtraTrees, GBM, kNN, LDA, LightGBM, LR, NB, PCA, QDA, RC, RF, SVM & XGBoost
125. Tian (2025)[183]	Dihydroxypropyl mercapturic acid (DHPMA) and dietary 3-monochloropropane-1,2-diol (3-MCPD), glycidol and their esters	CatBoost, GAM, LightGBM, MLR, RF, SVM & XGBoost
126. Xie (2025)[184]	Brominated flame retardants and emphysema	BA, DT, ENET, kNN, LightGBM, NN, PCA, RF, SHAP, SVM & XGBoost
127. Xu (2025)[185]	Organophosphate flame retardants and depression	DT
**Multiple Chemical Classes**		
128. Krysiak-Baltyn (2012)[186]	Multiple chemicals and congenital cryptorchidism	Linear PLS, NN & SVM
129. Oskar (2021)[187]	Multiple chemicals and timing of menarche	RF
130. Sen (2022)[6]	Multiple chemicals and measures of non-alcoholic fatty liver disease	GBM, Model-based clustering, PCA & SHAP
131. Wei (2022)[188]	Multiple chemicals and diabetes mellitus	GLM, LASSO, LR, PCA & RF
132. Li (2023)[189]	Multiple chemicals and frailty	NN & SHAP
133. Llopis (2023)[190]	Multiple chemicals and vitamin D	AB, DT, LR, NB, NN, RF, SHAP & XGBoost
134. Soomro (2023)[191]	Multiple chemicals and pregnancy-induced hypertension	LASSO (regularized and double LASSO LR)
135. Duan (2024)[192]	Multiple chemicals and cardiovascular/cancer/all-cause mortality	CoxPH, ENET, ExtraSurvival Trees, GBM, RF & SHAP
136. Feng (2024)[193]	Multiple chemicals and muscle quality	AB, ENET, LR, NB, RF, SHAP, SVM & XGBoost
137. Guo (2024)[194]	Multiple chemicals and hypertension	ENET, Ensemble, kNN, NB, NN, PCA, RF, RT & SVM

138. Liu (2024)[195]	Multiple chemicals and hypertension	LR, NN, RF, SHAP, SKB, SVM & XGBoost
139. Soomro (2024)[196]	Multiple chemicals and gestational diabetes mellitus	LASSO (regularized and double LASSO LR)
140. Yang (2024)[197]	Multiple chemicals and oxidative stress	ENET, GBM, kNN, LASSO, RF, RR, SHAP, SVM & XGBoost
141. Deng (2025)[198]	Multiple chemicals and preserved ratio impaired spirometry	AB, CatBoost, DT, ExtraTrees, LASSO, LDA, LightGBM, SHAP & XGBoost
142. England-Mason (2025)[199]	Multiple chemicals and neurodevelopment	RReliefF algorithm & SVM
143. Jo (2025)[200]	Multiple chemicals and metabolic syndrome	DT, GBM, LightGBM, NN, RF, SHAP, SVM & XGBoost
144. Lee (2025)[201]	Multiple chemicals and chronic kidney disease	DT, Ensemble, LASSO, LR, RF & RR
145. Liu (2025)[202]	Multiple chemicals and obesity	AB, ENET, kNN, NB, RF, SHAP, SVM & XGBoost
146. Liu (2025)[203]	Multiple chemicals and chronic kidney disease	GBM, NN, RF, SHAP & XGBoost
147. Lu (2025)[204]	Multiple chemicals and hyperuricemia	AB, LASSO, LightGBM, NB, RF, SHAP, SVM & XGBoost
148. Shi (2025)[205]	Multiple chemicals and early pregnancy loss	LASSO (L1 regularized LR), OPLS-DA, PCA, RF, SHAP, SVM & XGBoost
149. Yan (2025)[206]	Multiple chemicals and hepatic steatosis	Ensemble, LASSO, NN, SHAP, SVM & XGBoost
150. Yang (2025)[207]	Multiple chemicals and biomarkers of kidney injury and oxidative stress	Graphical causal model, RF & SHAP
151. Zhang (2025)[208]	Multiple chemicals and chronic kidney disease	kNN, LR, RF, SHAP, SKB SVM & XGBoost

[1] Focus of RF analysis was to identify metal-microbial cliques. [2] Specific chemicals included were not reported in the paper.

Abbreviations: AB = adaptive boosting; ALE = accumulated local effects; BA = Boruta algorithm; BKMR = Bayesian kernel machine regression; BN = Bayesian network; CatBoost = categorical boosting; CDA = canonical discriminant analysis; CLV = clustering of variables around latent variables; COPD = chronic obstructive pulmonary disease; CoxPH = Cox proportional hazards model; DBSCAN = density-based spatial clustering of applications with noise; DNA = deoxyribonucleic acid; DT = decision tree; EFA = exploratory factor analysis; ENET = elastic net; fMRI = functional magnetic resonance imaging; GAM = generalized additive model; GBM = gradient boosting machine; GLM = generalized linear model; GP = Gaussian process; HC = hierarchical clustering; kNN = k-nearest neighbours; LASSO = least absolute shrinkage and selection operator; LDA = linear discriminant analysis; LightGBM = light gradient boosting machine; LIME = local interpretable model-agnostic explanations; LR = logistic regression; LUCID = latent unknown clustering with integrated data; MARS = multivariate adaptive regression splines; MLR = multiple linear regression; NB = Naïve Bayes; NN = neural network; OPLS-DA = orthogonal partial least square-discriminant analysis; PAHs = polycyclic aromatic hydrocarbons; PCA = principal component analysis; PCBs = polychlorinated biphenyls; PFAS = per- and polyfluoroalkyl substances; PLS = partial least squares; PLS-DA = partial least squares discriminant analysis; PLS-GLM = partial least squares-generalized linear model; QDA = quadratic discriminant analysis; RC = ridge classifier; RF = random forest; RM = redescription mining; RR = ridge regression; RT = random tree; SGD = stochastic gradient descent; SHAP = Shapley additive explanation; SKB = select K best algorithm; sPLS = sparse partial least squares; SVM = support vector machine; $TCDD_{eq}$ = tetrachlorodibenzo-p-dioxin equivalent; TMLE = targeted maximum likelihood/minimum loss estimation; WQSR = weighted quantile sum regression; XGBoost = extreme gradient boosting machine

Table S8. Association/Prediction – Health-Based Outcomes (from chemical mixtures)

- Question: Is there an association between chemical mixtures and a health-related outcome? OR Can chemical mixtures predict a health-related outcome?

References	Brief Description	Methods
**Metals**		
1. Park (2017)[209]	Metals/metalloids and gamma-glutamyl transferase	BART, BKMR, ENET & SL[1]
2. Luo (2020)[210]	Metals/metalloids and kidney function	BKMR
3. Tao (2021)[16]	Metals/metalloids and sex steroid hormones	PCA (with linear regression) & WQSR
4. Grau-Perez (2022)[17]	Metals/metalloids and atherosclerosis	HC, PCA (with linear regression) & BKMR
5. Liu (2022)[211]	Metals/metalloids and blood pressure/hypertension	BKMR
6. Michael (2022)[212]	Metals/metalloids and anthropometric measures	BKMR
7. Su (2022)[213]	Metals/metalloids/PFAS and kidney function	BKMR
8. Takatani (2022)[214]	Metals/metalloids and anthropometric measures/SGA	BKMR & QGC[2]
9. Borghese (2023)[215]	Metals/metalloids and hypertensive disorders of pregnancy	QGC
10. Chen (2023)[216]	Metals/metalloids and pulmonary function	BKMR & QGC
11. Gao (2023)[217]	Metals-microcystins and chronic kidney disease	BKMR
12. Liang (2023)[218]	Metals/metalloids and chronic kidney disease	BKMR & WQSR
13. Ma (2023)[219]	Metals/metalloids and growth/behavioural development	BKMR, QGC & WQSR
14. Shen (2023)[220]	Metals/metalloids and kidney function	BKMR, LASSO & LR
15. Wu (2023)[221]	Metals/metalloids and diabetes mellitus/HBA1c	BKMR & WQSR
16. Yu (2023)[222]	Metals/metalloids and kidney function	BKMR
17. Zhu (2023)[223]	Metals/metalloids and prediabetes/diabetes	BKMR, WQSR & LASSO
18. An (2024)[224]	Metals/metalloids and early kidney damage	BKMR & WQSR
19. Chen (2024)[94]	Metals/metalloids and chronic kidney disease	BKMR
20. Du (2024)[95]	Metals/metalloids and blood lipids	BKMR & QGC
21. Fan (2024)[225]	Metals/metalloids and bone mineral density	BKMR
22. Fu (2024)[226]	Metals/metalloids and hemoglobin	BKMR & QGC
23. Huang (2024)[227]	Metals/metalloids and age at menarche, menopause and reproductive lifespan	BKMR & WQSR
24. Kim (2024)[228]	Metals/metalloids and blood pressure	BKMR
25. Liu (2024)[8]	Metals/metalloids and osteoporosis	k-Medoid (with LR) & WQSR
26. Long (2024)[229]	Metals/metalloids and blood pressure	BKMR
27. Pan (2024)[230]	Metals/metalloids and kidney function	BKMR & WQSR

28. Qiao (2024)[231]	Metals/metalloids and anemia	BKMR & QGC
29. Schildroth (2024)[232]	Metals/metalloids and verbal learning/memory	BKMR-CMA[3]
30. Wei (2024)[233]	Metals/metalloids and orofacial clefts	BA, LASSO & BKMR
31. Wu (2024)[110]	Metals/metalloids and chronic kidney disease	BKMR
32. Xiang (2024)[234]	Metals/metalloids and vital capacity index	ENET & BKMR, QGC, WQSR
33. Yan (2024)[111]	Metals/metalloids and glioma	QGC & WQSR
34. Yang (2024)[235]	Metals/metalloids and fracture/bone mineral density	BKMR & QGC
35. Zhou (2024)[236]	Metals/metalloids and obesity	BKMR, QCG & WQSR
36. Chen (2025)[121]	Metals/metalloids and blood pressure	PCA & BKMR
37. Chi (2025)[24]	Metals/metalloids and infertility	PCA (with LR), BKMR & QGC
38. Go (2025)[237]	Metals/metalloids and gestational diabetes mellitus, preterm birth and dyslipidemia	BKMR
39. Hu (2025)[125]	Metals/metalloids and human papilloma virus infection	WQSR
40. Ji (2025)[238]	Metals/metalloids and congestive heart failure	BKMR & WQSR
41. Liu (2025)[203]	Metals/metalloids and chronic kidney disease	BKMR
42. Rodriguez (2025)[239]	Metals/metalloids and sleep duration	BKMR
43. Shi (2025)[136]	Metals/metalloids and digestive tract cancer	WQSR
44. Wen (2025)[139]	Metals/metalloids and chronic cough	BKMR & WQSR
45. Wu (2025)[140]	Metals/metalloids and gallstone disease	WQSR
46. Yang (2025)[240]	Metals/metalloids and accelerated aging	BKMR
47. Yu (2025)[241]	Metals/metalloids and thyroid cancer	BKMR & WQSR
48. Zhong (2025)[147]	Metals/metalloids and gestational anemia	BKMR & QGC
**Per- and Polyfluoroalkyl Substances**		
49. Borghese (2022)[242]	PFAS and liver function biomarkers	QGC
50. Guo (2023)[243]	PFAS and sex steroid hormones	BKMR & WQSR
51. Li (2024)[244]	PFAS and congenital heart disease	BKMR & QGC
52. Wang (2024)[245]	PFAS and prostate hyperplasia	WQSR
53. Abuduxukuer (2025)[246]	PFAS and developmental defects of enamel and dental carries	BKMR, QGC & WQSR
54. Borghese (2025)[247]	PFAS and lipids/liver function biomarkers	QGC & WQSR
55. Liao (2025)[248]	PFAS and blood pressure	BKMR, QGC & RF[4]
56. Liu (2025)[249]	PFAS and cataract	BKMR & WQSR

57. Wang (2025)[152]	PFAS and thyroid cancer and metabolome	BKMR & WQSR
58. Yang (2025)[154]	PFAS and abdominal aortic calcification	QGC
59. Yang (2025)[250]	PFAS and glucose, insulin, HOMA-IR indices and sex hormones	BKMR & WQSR
**Phthalates**		
60. Chiu (2018)[12]	Phthalates and birthweight	PCA (with linear regression) & BKMR
61. Hou (2019)[251]	Phthalates/PAHs and DNA damage	BKMR
62. Yu (2023)[19]	Phthalates and cognitive impairment	PCA & WQSR
63. Zhang (2024)[23]	Phthalates and sex steroid hormones/obesity	PCA & WQSR
**Pesticides and Other Persistent Organic Pollutants**		
64. Gibson (2019)[14]	PCBs/dioxins/furans and leukocyte telomere length	EFA, HC, k-Means, PCA, BKMR & WQSR
65. Kang (2022)[252]	Organophosphate esters and sleep outcomes	BKMR
66. Cano-Sancho (2023)[253]	Persistent organic pollutants and childhood obesity	BKMR, GBM & LASSO[5]
67. Hu (2023)[254]	Organophosphate esters and lung function	BKMR
68. Deng (2024)[255]	Pesticides and oxidative stress	BKMR
69. Deng (2024)[256]	Organophosphate esters and gestational diabetes mellitus and glucose parameters	DT, QGC, RF & SVM
70. Wu (2024) [257]	Neonicotinoids and obesity	BKMR & WQSR
71. Pan (2025)[169]	Neonicotinoids and gestational diabetes mellitus	BKMR & QGC
72. Wang (2025)[171]	Organophosphate pesticides and triglyceride glucose index	QGC & WQSR
**Polycyclic Aromatic Hydrocarbons**		
73. Duan (2024)[258]	PAHs and cardiovascular/all-cause mortality	BKMR & QGC
74. Lu (2024)[259]	PAHs and cognitive function	BKMR & WQSR
75. Pi (2024)[260]	PAHs and congenital heart defects	BKMR & WQSR
76. Wei (2024)[261]	PAHs and lung function and club cell secretory protein	BKMR
77. Wu (2024)[262]	PAHs/metals and lung function	BKMR, QGC & LASSO
78. Zang (2024)[22]	PAHs and chronic bowel disorders	k-Means (with LR), PCA (with LR), BKMR & WQSR
79. Zang (2024)[263]	PAHs and visual impairment	BKMR, ENET, LASSO & WQSR
80. Zhang (2024)[264]	PAHs and endometriosis	BKMR
**Volatile Organic Compounds**		
81. Liu (2023)[265]	Volatile organic compounds and NAFLD	BKMR & WQSR
82. Wei (2023)[266]	Volatile organic compounds and sex hormones	BKMR, WQSR & XGBoost
83. Liu (2024)[174]	Volatile organic compounds and COPD	WQSR

84. Wang (2024)[267]	Volatile organic compounds and COPD	BKMR, QGC & WQSR
85. Wang (2024)[20]	Semi-volatile organic compounds and thyroid nodule	BKMR, CLV, PCA & WQSR
86. Wang (2024)[9]	Volatile organic compounds and oxidative damage biomarkers	BKMR, QGC & WQSR
87. Deng (2025)[175]	Volatile organic compounds and preserved ratio impaired spirometry	BKMR, QGC & WQSR
88. Liu (2025)[268]	Volatile organic compounds and sarcopenia	BKMR, LASSO, QGC & WQSR
89. Zhang (2025)[177]	Volatile organic compounds and chronic kidney disease	BKMR, QGC, WQSR & LASSO
**Other**		
90. Fu (2022)[5]	Personal care & consumer chemicals and hearing threshold	k-Medoid & BKMR
91. Fang (2024)[269]	Aldehydes and cardiovascular disease	BKMR
92. Ge (2024)[270]	Aldehydes and depression	BKMR
93. Liu (2024)[179]	Phenols and kidney function	QGC
94. Tang (2024)[271]	Synthetic steroid hormones and precocious puberty	BKMR, WQSR & LASSO
95. Xiao (2024)[272]	Phenols and ulcerative colitis	BKMR
96. Cai (2025)[181]	Brominated flame retardants and hyperuricemia	WQSR
97. Du (2025)[47]	Organophosphate flame retardants and thyroid function indicators	BKMR & WQSR
98. Su (2025)[273]	Bisphenol analogues and intelligence quotient	BKMR
99. Xie (2025)[184]	Brominated flame retardants and emphysema	WQSR
100. Zhao (2025)[274]	Brominated flame retardants and atherosclerosis	BKMR & QGC
101. Zhou (2025)[11]	Phenols and child neurodevelopment	BKMR, QGC, SOM (with linear regression) & WQSR
**Multiple Chemical Classes**		
102. Tanner (2020)[275]	Multiple chemicals and intelligence quotient	WQSR
103. Wu (2020)[276]	Multiple chemicals and obesity/BMI	BKMR & WQSR
104. Hu (2021)[277]	Multiple chemicals and birthweight	BKMR
105. Kim (2022)[278]	Multiple chemicals and triglycerides/HDL-C/BMI	BKMR
106. Li (2023)[189]	Multiple chemicals and frailty	WQSR
107. Midya (2023)[279]	Multiple chemicals and autism	BKMR, LR, RF & WQSR[6]
108. Jang (2024)[280]	Multiple chemicals and kidney disease biomarkers	BART, BKMR, ENET, MLR, SL & WQSR[7]
109. Keil (2024)[39]	Multiple chemicals and leukocyte telomere length	ENET, LASSO, QGC, RF, SL, TMLE & WQSR
110. Li (2024)[281]	Multiple chemicals and metabolic syndrome	QGC, RF, WQSR, BA, LASSO & XGBoost

111. Midya (2024)[282]	Multiple chemicals and LDL-C	RF, WQSR & XGBoost
112. Wu (2024)[21]	Multiple chemicals and semen quality	PCA (with linear regression), PCP & BKMR
113. Alampi (2025)[283]	Multiple chemicals and autistic-like behaviours	QGC & WQSR
114. Deng (2025)[198]	Multiple chemicals and preserved ratio impaired spirometry	BKMR, QGC & WQSR
115. Guo (2025)[284]	Multiple chemicals and thyroid hormones	BKMR
116. Haruna (2025)[285]	Multiple chemicals and chronic kidney disease	BKMR, QGC & WQSR
117. Huang (2025)[286]	Multiple chemicals and accelerated bone maturation	BKMR & WQSR
118. Jehu-Appiah (2025)[287]	Multiple chemicals and cardiovascular disease markers (blood pressure, HDL-C, LDL-C, total cholesterol and triglycerides)	BKMR
119. Lee (2025)[201]	Multiple chemicals and chronic kidney disease	WQSR
120. Puvvula (2025)[288]	Multiple chemicals and cognitive abilities	BKMR & QGC

[1] The ML methods were used to construct an ERS for metals based on outcome of gamma-glutamyl transferase (a marker of oxidative stress). The ERS was associated with blood pressure, hypertension, and mortality using traditional statistical methods (i.e., linear regression, logistic regression, and Cox-proportional hazards model).
[2] Focus of BKMR analysis was to identify metal-metal interactions.
[3] A CMA extension to BMKR was applied to quantify mediation by iron status.
[4] The purpose of RF analysis was to identify covariates that interact with PFAS on blood pressure.
[5] Focus of ML analyses was to identify chemical-nutrient interactions.
[6] The purpose of RF analysis was to identify chemical interactions.
[7] The ML methods were used to construct an environmental risk score (ERS) from 9 heavy metals, 4 polycyclic aromatic hydrocarbons, and 4 volatile organic compounds. The optimal model was then applied to associate the ERS with kidney disease biomarkers (beta-2-microglobulin, N-acetyl-β-glucosaminidase, and estimated glomerular filtration rate).

Abbreviations: BA = Boruta algorithm; BART = Bayesian additive regression tree; BKMR = Bayesian kernel machine regression; BMI = body mass index; CLV = clustering of variables around latent variables; CMA = causal mediation analysis; COPD = chronic obstructive pulmonary disease; DNA = deoxyribonucleic acid; DT = decision tree; EFA = exploratory factor analysis; ENET = elastic net; GBM= gradient boosting machine; HBA1c = glycated hemoglobin; HC = hierarchical clustering; HDL-C = high density lipoprotein cholesterol; HOMA-IR = homeostatic model assessment of insulin resistance; LASSO = least absolute shrinkage and selection operator; LDL-C = low density lipoprotein cholesterol; LR = logistic regression; MLR = multiple linear regression; NAFLD = non-alcoholic fatty liver disease; PAHs = polycyclic aromatic hydrocarbons; PCA = principal component analysis; PCBs = polychlorinated biphenyls; PCP = principal component pursuit; PFAS = per- and polyfluoroalkyl substances; QGC = quantile based G-computation; RF = random forest; SGA = small for gestational age; SL = super learner; SOM = self-organizing maps; SVM = support vector machine; TMLE = targeted maximum likelihood/minimum loss estimation; WQSR = weighted quantile sum regression; XGBoost = extreme gradient boosting

1. Buck Louis, G.M.; Yeung, E.; Kannan, K.; Maisog, J.; Zhang, C.; Grantz, K.L.; Sundaram, R. Patterns and variability of endocrine-disrupting chemicals during pregnancy: Implications for understanding the exposome of normal pregnancy. *Epidemiology* **2019**, *30*, S65–S75. doi: 10.1097/EDE.0000000000001082

2. Stanfield, Z.; Setzer, R.W.; Hull, V.; Sayre, R.R.; Isaacs, K.K.; Wambaugh, J.F. Characterizing chemical exposure trends from NHANES urinary biomonitoring data. *Environ. Health Perspect.* **2024**, *132*, 017009. doi: 10.1289/EHP12188

3. Kalloo, G.; Wellenius, G.A.; McCandless, L.; Calafat, A.M.; Sjodin, A.; Karagas, M.; Chen, A.; Yolton, K.; Lanphear, B.P.; Braun, J.M. Profiles and predictors of environmental chemical mixture exposure among pregnant women: The Health Outcomes and Measures of the Environment Study. *Environ. Sci. Technol.* **2018**, *52,* 10104–10113, 2018. doi: 10.1021/acs.est.8b02946

4. Carroll, R.; White, A.J.; Keil, A.P.; Meeker, J.D.; McElrath, T.F.; Zhao, S.; Ferguson, K.K. Latent classes for chemical mixtures analyses in epidemiology: An example using phthalate and phenol exposure biomarkers in pregnant women. *J. Expo. Sci. Environ. Epidemiol.* **2020**, *30,* 149–159. doi: 10.1038/s41370-019-0181-y

5. Fu, Y.-P.; Chen, W.-Y.; Guo, L.-Q.; Zhu, Y.-Q.; Yuan, J.-S.; Liu, Y.-H. The association between hearing threshold and urinary personal care and consumer product metabolites in middle-aged and elderly people from the USA. *Environ. Sci. Pollut. Res. Int.* **2022**, *29*, 81076–81086. doi: 10.1007/s11356-022-21459-5

6. Sen, P.; Qadri, S.; Luukkonen, P.K.; Ragnarsdottir, O.; McGlinchey, A.; Jäntti, S.; Juuti, A.; Arola, J.; Schlezinger, J.J.; Webster, T.F.; Orešič, M.; Yki-Järvinen, H.; Hyötyläinen, T. Exposure to environmental contaminants is associated with altered hepatic lipid metabolism in non-alcoholic fatty liver disease. *J. Hepatol.* **2022**, *76,* 283–293. doi: 10.1016/j.jhep.2021.09.039

7. Yonkman, A.M.; Alampi, J.D.; Kaida, A.; Allen, R.W.; Chen, A.; Lanphear, B.P.; Braun, J.M.; Muckle, G.; Arbuckle, T.E.; McCandless, L.C. Using latent profile analysis to identify associations between gestational chemical mixtures and child neurodevelopment. *Epidemiology* **2023**, *34,* 45–55. doi: 10.1097/EDE.0000000000001554

8. Liu, J.; Wang, K. Disentangling the relationship between urinary metal exposure and osteoporosis risk across a broad population: A comprehensive supervised and unsupervised analysis. *Toxics* **2024**, *12*, 866. doi: 10.3390/toxics12120866

9. Wang, J.-R.; Kuang, H.-X.; Liu, Y.; Li, X.-Y.; Chen, T.-H.; Zhu, X.-H.; Fan, R.-F.; Xiang, M.-D.; Yu, Y.-J. Associations between volatile organic compounds exposure and multiple oxidative damage biomarkers: Method development, human exposure, and application for e-waste pollution prediction. *Sci. Total Environ.* **2024**, *956*, 177402. doi: 10.1016/j.scitotenv.2024.177402

10. Moro, A.M.; Brucker, N.; Goethel, G.; Flesch, I.; Nascimento, S.; Charão, M.; Gauer, B.; Sauer, E.; Cestonaro, L.V.; Viçozzi, G.P.; Gioda, A.; Saint'Pierre, T.D.; Arbo, M.D.; Garcia, I.; Cattani, S.A.; Petrecelli, R.R.; Martins, M.O.; Garcia, S.C. The influence of blood titanium levels on DNA damage in Brazilian workers occupationally exposed to different chemical agents. *Biol. Trace Elem. Res.* **2025**, *203*, 4013–4026. doi: 10.1007/s12011-024-04472-2

11. Zhou, T.; Abrishamcar, S.; Christensen, G.; Eick, S.M.; Barr, D.B.; Vanker, A.; Hoffman, N.; Donald, K.A.; Wedderburn, C.J.; Andra, S.S.; Wright, R.O.; Zar, H.J.; Stein, D.J.; Hüls, A. Associations between prenatal exposure to environmental phenols and child neurodevelopment at two years of age in a South African birth cohort. *Environ. Res.* **2025**, *264*, 120325. doi: 10.1016/j.envres.2024.120325

12. Chiu, Y.H.; Bellavia, A.; James-Todd, T.; Correia, K.F.; Valeri, L.; Messerlian, C.; Ford, J.B.; Mínguez-Alarcón, L.; Calafat, A.M.; Hauser, R.; Williams, P.L. Evaluating effects of prenatal exposure to phthalate mixtures on birth weight: A comparison of three statistical approaches. *Environ. Int.* **2018**, *113*, 231–239. doi: 10.1016/j.envint.2018.02.005

13. Hendryx, M.; Luo, J. Latent class analysis of the association between polycyclic aromatic hydrocarbon exposures and body mass index. *Environ. Int.* **2018**, *121*, 227–231. doi: 10.1016/j.envint.2018.09.016

14. Gibson, E.A.; Nunez, Y.; Abuawad, A.; Zota, A.R.; Renzetti, S.; Devick, K.L.; Gennings, C.; Goldsmith, J.; Coull, B.A.; Kioumourtzoglou, M.-A. An overview of methods to address distinct research questions on environmental mixtures: An application to persistent organic pollutants and leukocyte telomere length. *Environ. Health* **2019**, *18*, 76. doi: 10.1186/s12940-019-0515-1

15. Matta, K.; Vigneau, E.; Cariou, V.; Mouret, D.; Ploteau, S.; Le Bizec, B.; Antignac, J.-P.; Cano-Sancho, G. Associations between persistent organic pollutants and endometriosis: A multipollutant assessment using machine learning algorithms. *Environ. Pollut.* **2020**, *260*, 114066. doi: 10.1016/j.envpol.2020.114066

16. Tao, C.; Li, Z.; Fan, Y.; Li, X.; Qian, H.; Yu, H.; Xu, Q.; Lu, C. Independent and combined associations of urinary heavy metals exposure and serum sex hormones among adults in NHANES 2013–2016. *Environ. Pollut.* **2021**, *281*, 117097. doi: 10.1016/j.envpol.2021.117097

17. Grau-Perez, M.; Caballero-Mateos, M.J.; Domingo-Relloso, A.; Navas-Acien, A.; Gomez-Ariza, J.L.; Garcia-Barrera, T.; Leon-Latre, M.; Soriano-Gil, Z.; Jarauta, E.; Cenarro, A.; Moreno-Franco, B.; Laclaustra, M.; Civeira, F.; Casasnovas, J.A.; Guallar, E.; Tellez-Plaza, M. Toxic metals and subclinical atherosclerosis in carotid, femoral, and coronary vascular territories: The Aragon Workers Health Study. *Arterioscler. Thromb. Vasc. Biol.* **2022**, *42*, 87–99. doi: 10.1161/ATVBAHA.121.316358

18. Babić Leko, M.; Mihelčić, M.; Jurasović, J.; Nikolac Perković, M.; Španić, E.; Sekovanić, A.; Orct, T.; Zubčić, K.; Langer Horvat, L.; Pleić, N.; Kiđemet-Piskač, S.; Vogrinc, Ž.; Pivac, N.; Diana, A.; Borovečki, F.; Hof, P.R.; Šimić, G. Heavy metals and essential metals are associated with cerebrospinal fluid biomarkers of Alzheimer's disease. *Int. J. Mol. Sci.* **2023**, *24*, 467. doi: 10.3390/ijms24010467

19. Yu, Y.; Wang, Y.; Dong, Y.; Shu, S.; Zhang, D.; Xu, J.; Zhang, Y.; Shi, W.; Wang, S.-L. Butyl benzyl phthalate as a key component of phthalate ester in relation to cognitive impairment in NHANES elderly individuals and experimental mice. *Environ. Sci. Pollut. Res. Int.* **2023**, *30*, 47544–47560. doi: 10.1007/s11356-023-25729-8

20. Wang, F.; Lin, Y.; Xu, J.; Wei, F.; Huang, S.; Wen, S.; Zhou, H.; Jiang, Y.; Wang, H.; Ling, W.; Li, X.; Yang, X. Risk of papillary thyroid carcinoma and nodular goiter associated with exposure to semi-volatile organic compounds: A multi-pollutant assessment based on machine learning algorithms. *Sci. Total Environ.* **2024**, *915*, 169962. doi: 10.1016/j.scitotenv.2024.169962

21. Wu, H.; Kalia, V.; Manz, K.E.; Chillrud, L.; Dishon, N.H.; Jackson, G.L.; Dye, C.K.; Orvieto, R.; Aizer, A.; Levine, H.; Kioumourtzoglou, M.-A.; Pennell, K.D.; Baccarelli, A.A.; Machtinger, R. Exposome profiling of environmental pollutants in seminal plasma and novel associations with semen parameters. *Environ. Sci. Technol.* **2024**, *58*, 13594–13604. doi: 10.1021/acs.est.3c10314

22. Zang, X.; Feng, L.; Qin, W.; Wang, W.; Zang, X. Using machine learning methods to analyze the association between urinary polycyclic aromatic hydrocarbons and chronic bowel disorders in American adults. *Chemosphere* **2024**, *346,* 140602. doi: 10.1016/j.chemosphere.2023.140602

23. Zhang, J.; Gu, W.; Zhai, S.; Liu, Y.; Yang, C.; Xiao, L.; Chen, D. Phthalate metabolites and sex steroid hormones in relation to obesity in US adults: NHANES 2013–2016. *Front. Endocrinol.* **2024**, *15*, 1340664. doi: 10.3389/fendo.2024.1340664

24. Chi, H.-B.; Tang, J.-J.; Fan, X.-Y.; Zhang, H.-W.; Tang, F.; Lin, X.-S.; Yang, B.-R.; Li, N.; Guo, J.; Wu, L.-A.-S.; Huang, Q.-Q.; Xia, Y.-Y. Single- and combined-heavy metals/metalloids exposures are associated with infertility in US women aged 20–44: NHANES 2013–2020 analysis. *Reprod. Toxicol.* **2025**, *132*, 108851. doi: 10.1016/j.reprotox.2025.108851

25. Lai, Y.; Nakayama, S.F.; Nishihama, Y.; Isobe, T.; The Japan Environment and Children's Study Group. Determinants of plasma poly- and perfluoroalkyl substances during pregnancy: The Japan Environment and Children's Study. *Ecotoxicol. Environ. Saf.* **2025**, *294*, 118107. doi: 10.1016/j.ecoenv.2025.118107

26. Jovanović, G.; Romanić, S.H.; Stojić, A.; Klinčić, D.; Sarić, M.M.; Letinić, J.G.; Popović, A. Introducing of modeling techniques in the research of POPs in breast milk – A pilot study. *Ecotoxicol. Environ. Saf.* **2019**, *172,* 341–347. doi: 10.1016/j.ecoenv.2019.01.087

27. Potash, E.; Ghani, R.; Walsh, J.; Jorgensen, E.; Lohff, C.; Prachand, N.; Mansour, R. Validation of a machine learning model to predict childhood lead poisoning. *JAMA Netw Open* **2020**, *3,* e2012734. doi: 10.1001/jamanetworkopen.2020.12734

28. Liu, X.; Taylor, M.P.; Aelion, C.M.; Dong, C. Novel application of machine learning algorithms and model-agnostic methods to identify factors influencing childhood blood lead levels. *Environ. Sci. Technol.* **2021**, *55,* 13387–13399. doi: 10.1021/acs.est.1c01097

29. Nishihama, Y.; Nakayama, S.F.; Isobe, T.; Jung, C.-R.; Iwai-Shimada, M.; Kobayashi, Y.; Michikawa, T.; Sekiyama, M.; Taniguchi, Y.; Yamazaki, S. Urinary metabolites of organophosphate pesticides among pregnant women participating in the Japan Environment and Children's Study (JECS). *Int. J. Environ. Res. Public Health* **2021**, *18,* 5929. doi: 10.3390/ijerph18115929

30. Mulhern, R.; Roostaei, J.; Schwetschenau, S.; Pruthi, T.; Campbell, C.; MacDonald Gibson, J. A new approach to a legacy concern: Evaluating machine-learned Bayesian networks to predict

childhood lead exposure risk from community water systems. *Environ. Res.* **2022**, *204,* 112146. doi: 10.1016/j.envres.2021.112146

31. Abbasi, A.; DiTraglia, F.J.; Gazze, L.; Pals, B. Hidden hazards and screening policy: Predicting undetected lead exposure in Illinois. *J. Health. Econ.* **2023**, *90,* 102783. doi: 10.1016/j.jhealeco.2023.102783

32. Chaurasia, P.; McClean, S.I.; Mahdi, A.A.; Yogarajah, P.; Ansari, J.A.; Kunwar, S.; Ahmad, M.K. Automated lead toxicity prediction using computational modelling framework. *Health Inf. Sci. Syst.* **2023**, *11,* 56. doi: 10.1007/s13755-023-00257-4

33. Sambanis, A.; Osiecki, K.; Cailas, M.; Quinsey, L.; Jacobs, D.E. Using artificial intelligence to identify sources and pathways of lead exposure in children. *J. Public Health Manag. Pract.* **2023**, *29,* E208–E213. doi: 10.1097/PHH.0000000000001759

34. Shin, S.; Ryoo, J.-H. Environment-wide association study to identify exposure pathways of bisphenol A in Korean children and adolescents: Korean National Environmental Health Survey (KoNEHS) 2018-2020. *Environ. Res.* **2023**, *238,* 117187. doi: 10.1016/j.envres.2023.117187

35. Wang, X.; Bakulski, K.M.; Mukherjee, B.; Hu, H.; Park, S.K. Predicting cumulative lead (Pb) exposure using the Super Learner algorithm. *Chemosphere*, **2023**, *311,* 137125. doi: 10.1016/j.chemosphere.2022.137125

36. Aker, A.; Nguyen, V.; Ayotte, P.; Ricard, S.; Lemire, M. Characterizing important dietary exposure sources of perfluoroalkyl acids in Inuit youth and adults in Nunavik using a feature selection tool. *Environ. Health Perspect.* **2024**, *132,* 047014. doi: 10.1289/EHP13556

37. Frndak, S.; Queirolo, E.I.; Mañay, N.; Yu, G.; Ahmed, Z.; Barg, G.; Colder, C.; Kordas, K. Predicting blood lead in Uruguayan children: Individual- vs neighborhood-level ensemble learners. *PLOS Glob. Public Health* **2024**, *4,* e0003607. doi: 10.1371/journal.pgph.0003607

38. Jeong, S.; Choi, Y.-J. Investigating the influence of heavy metals and environmental factors on metabolic syndrome risk based on nutrient intake: Machine learning analysis of data from the eighth Korea National Health and Nutrition Examination Survey (KNHANES). *Nutrients* **2024**, *16,* 724. doi: 10.3390/nu16050724

39. Keil, A.P.; O'Brien, K.M. Considerations and targeted approaches to identifying bad actors in exposure mixtures. *Stat. Biosci.* **2024**, *16,* 459–481. doi: 10.1007/s12561-023-09409-2

40. Mahfouz, M.; Mahfouz, Y.; Harmouche-Karaki, M.; Matta, J.; Younes, H.; Helou, K.; Finan, R.; Abi-Tayeh, G.; Meslimani, M.; Moussa, G.; Chahrour, N.; Osseiran, C.; Skaiki, F.; Narbonne, J.-F. Utilizing machine learning to classify persistent organic pollutants in the serum of pregnant women: A predictive modeling approach. *Environ. Sci. and Pollut. Res. Int.* **2024**, *31,* 52980–52995. doi: 10.1007/s11356-024-34684-x

41. Suwannarin, N.; Nishihama, Y.; Isobe, T.; Nakayama, S.F. Urinary concentrations of environmental phenol among pregnant women in the Japan Environment and Children's Study. *Environ. Int.* **2024**, *183,* 108373. doi: 10.1016/j.envint.2023.108373

42. Sy, M.; Conrad, A.; Jung, C.; Lindtner, O.; Greiner, M. Analysis of human co-exposure to lead and cadmium using human biomonitoring (HBM) data in a Bayesian copula-based regression framework. *Expo. Health* **2024**, *16,* 503–516. doi: 10.1007/s12403-023-00573-w

43. Tao, L.; Tang, W.; Xia, Z.; Wu, B.; Liu, H.; Fu, J.; Lu, Q.; Guo, L.; Gao, C.; Zhou, Q.; Fan, Y.; Xu, D.-X.; Huang, Y. Machine learning predicts the serum PFOA and PFOS levels in pregnant women: Enhancement of fatty acid status on model performance. *Environ. Int.* **2024**, *190,* 108837. doi: 10.1016/j.envint.2024.108837

44. Yang, Y.; Li, C.; Yang, L.; Zhu, H.; Xie, Z.; Falandysz, J.; Weber, R.; Qin, L.; Liu, G. Linking industrial emissions and dietary exposure to human burdens of polychlorinated naphthalenes. *Sci. Total Environ.* **2024**, *951,* 175733. doi: 10.1016/j.scitotenv.2024.175733

45. Chaurasia, P.; Yogarajah, P.; Mahdi, A.A.; McClean, S.; Ahmad, M.K.; Jafar, T.; Singh, S.K. Machine learning and explainable artificial intelligence to predict and interpret lead toxicity in pregnant women and unborn baby. *Front. Digit. Health* **2025**, *7,* 1608949. doi: 10.3389/fdgth.2025.1608949

46. Chou, W.-C.; Gaynor, J.W.; Graham, E.M.; Klepczynski, B.; Walker, T.; Coker, E.S.; Ittenbach, R.F.; Lin, Z. A machine learning-based clustering analysis to explore bisphenol A and phthalate exposure from medical devices in infants with congenital heart defects. *Environ. Health Perspect.* **2025**, *133,* 67016. doi: 10.1289/EHP15034

47. Du, Z.; Chen, D.; Du, X.; Chen, G.; Chen, T.; Zheng, W. Identification of the associations between co-exposure to organophosphate flame retardants and thyroid dysfunction and exposure risk factors in residents of Shanghai, China. *Environ. Pollut.* **2025**, *370,* 125911. doi: 10.1016/j.envpol.2025.125911

48. Jung, S.; Shah, S.; Oh, J.; Bang, Y.; Lee, J.H.; Kim, H.-C.; Jeong, K.S.; Park, H.; Lee, E.-K.; Hong, Y.-C.; Ha, E.; Ko-CHENS Study Group. Machine learning-based analysis on factors influencing blood heavy metal concentrations in the Korean CHildren's ENvironmental health Study (Ko-CHENS). *Sci. Total Environ.* **2025**, *978,* 179401. doi: 10.1016/j.scitotenv.2025.179401

49. Okati, N.; Ebrahimi-Khusfi, Z.; Zandifar, S.; Taghizadeh-Mehrjardi, R. Identifying the key factors of mercury exposure in residents of southwestern Iran using machine learning algorithms. *Environ. Geochem. Health* **2025**, *47,* 239. doi: 10.1007/s10653-025-02533-6

50. Qu, J.; Mao, W.; Chen, M.; Jin, H. Prediction of p-phenylenediamine antioxidant concentrations in human urine using machine learning models. *J. Hazard. Mater.* **2025**, *487,* 137184. doi: 10.1016/j.jhazmat.2025.137184

51. Fry, R.C.; Navasumrit, P.; Valiathan, C.; Svensson, J.P.; Hogan, B.J.; Luo, M.; Bhattacharya, S.; Kandjanapa, K.; Soontararuks, S.; Nookabkaew, S.; Mahidol, C.; Ruchirawat, M.; Samson, L.D. Activation of inflammation/NF-κB signaling in infants born to arsenic-exposed mothers. *PLoS Genet.* **2007**, *11,* 2180–2189. doi: 10.1371/journal.pgen.0030207

52. Demchenko, V.; Olszewski, S.; Voronenko, M.; Zaets, E.; Savina, N.; Lurie, I.; Lytvynenko, V. Modeling and predicting the organochlorine pesticides concentration in the child's body based on their accumulation in the mother's body. In CEUR Workshop Proceedings, 2020, 419–432.

53. Levin-Schwartz, Y.; Gennings, C.; Claus Henn, B.; Coull, B.A.; Placidi, D.; Lucchini, R.; Smith, D.R.; Wright, R.O. Multi-media biomarkers: Integrating information to improve lead exposure assessment. *Environ. Res.* **2020**, *183,* 109148. doi: 10.1016/j.envres.2020.109148

54. Gerbi, L.; Austin, C.; Pedretti, N.F.; McRae, N.; Amarasiriwardena, C.J.; Mercado-García, A.; Torres-Olascoaga, L.A.; Tellez-Rojo, M.M.; Wright, R.O.; Arora, M.; Elena, C. Biomarkers of maternal lead exposure during pregnancy using micro-spatial child deciduous dentine measurements. *Environ. Int.* **2022**, *169,* 107529. doi: 10.1016/j.envint.2022.107529

55. Jala, A.; Dutta, R.; Josyula, J.V.N.; Mutheneni, S.R.; Borkar, R.M. Environmental phenol exposure associates with urine metabolome alteration in young Northeast Indian females. *Chemosphere* **2023**, *317,* 137830. doi: 10.1016/j.chemosphere.2023.137830

56. Khanam, T.; Liang, S.; Xu, S.; Musstjab Akber Shah Eqani, S.A.; Shafqat, M.N.; Rasheed, H.; Bibi, N.; Shen, H.; Zhang, J. Arsenic exposure induces urinary metabolome disruption in Pakistani male population. *Chemosphere* **2023**, *312,* 137228. doi: 10.1016/j.chemosphere.2022.137228

57. Midya, V.; Lane, J.M.; Gennings, C.; Torres-Olascoaga, L.A.; Gregory, J.K.; Wright, R.O.; Arora, M.; Téllez-Rojo, M.M.; Eggers, S. Prenatal lead exposure is associated with reduced abundance of beneficial gut microbial cliques in late childhood: An investigation using Microbial Co-Occurrence Analysis (MiCA). *Environ. Sci. Technol.* **2023**, *57,* 16800–16810. doi: 10.1021/acs.est.3c04346

58. Zhang, G.; Lin, W.; Gao, N.; Lan, C.; Ren, M.; Yan, L.; Pan, B.; Xu, J.; Han, B.; Hu, L.; Chen, Y.; Wu, T.; Zhuang, L.; Lu, Q.; Wang, B.; Fang, M. Using machine learning to construct the blood-follicle distribution models of various trace elements and explore the transport-related pathways with multiomics data. *Environ. Sci. Technol.* **2024**, *58,* 7743–7757. doi: 10.1021/acs.est.3c10904

59. Zhao, X.; Chen, K.; Wang, J.; Qiu, Y. Analysis of prospective genetic indicators for prenatal exposure to arsenic in newborn cord blood using machine learning. *Biol. Trace Elem. Res.* **2024**, *202,* 2466–2473. doi: 10.1007/s12011-023-03863-1

60. Khodasevich, D.; Holland, N.; Van Der Laan, L.; Cardenas, A. A SuperLearner-based pipeline for the development of DNA methylation-derived predictors of phenotypic traits. *PLoS Comput. Biol.* **2025**, *21,* e1012768. doi: 10.1371/journal.pcbi.1012768

61. Sung, J.-M.; Hung, Y.-C.; Wang, W.-R.; Chu, C.-J.; Lin, Y.-P.; Liu, K.-H.; Mahmudiono, T.; Chen, H.-L. Integrating machine learning and metabolomics to uncover new biomarkers for predicting pesticide exposure among patients with kidney function decline. *Sci. Total Environ.* **2025**, *995,* 180066. doi: 10.1016/j.scitotenv.2025.180066

62. Zhang, Z.; Wang, Y.; Rodgers, T.F.M.; Wu, Y. Exposure experiments and machine learning revealed that personal care products can significantly increase transdermal exposure of SVOCs from the environment. *J. Hazard. Mater.* **2025**, *487,* 137271. doi: 10.1016/j.jhazmat.2025.137271

63. Tan, C.; Chen, H.; Xia, C. The prediction of cardiovascular disease based on trace element contents in hair and a classifier of boosting decision stumps. *Biol. Trace Elem. Res.* **2009**, *129,* 9–19. doi: 10.1007/s12011-008-8279-4

64. Golasik, M.; Jawień, W.; Przybyłowicz, A.; Szyfter, W.; Herman, M.; Golusiński, W.; Florek, E.; Piekoszewski, W. Classification models based on the level of metals in hair and nails of laryngeal cancer patients: Diagnosis support or rather speculation? *Metallomics* **2015**, *7,* 455–465. doi: 10.1039/c4mt00285g

65. Zabinski, J.W.; Garcia-Vargas, G.; Rubio-Andrade, M.; Fry, R.C.; Gibson, J.M. Advancing dose-response assessment methods for environmental regulatory impact analysis: A Bayesian belief network approach applied to inorganic arsenic. *Environ. Sci. Technol. Lett.* **2016**, *3,* 200–204. doi: 10.1021/acs.estlett.6b00076

66. De Benedetti, S.; Lucchini, G.; Del Bò, C.; Deon, V.; Marocchi, A.; Penco, S.; Lunetta, C.; Gianazza, E.; Bonomi, F.; Iametti, S. Blood trace metals in a sporadic amyotrophic lateral sclerosis geographical cluster. *Biometals* **2017**, *30,* 355–365. doi: 10.1007/s10534-017-0011-4

67. Lin, T.; Liu, T.; Lin, Y.; Yan, L.; Chen, Z.; Wang, J. Comparative study on serum levels of macro and trace elements in schizophrenia based on supervised learning methods. *J. Trace Elem. Med. Biol.* **2017**, *43,* 202–208. doi: 10.1016/j.jtemb.2017.03.010

68. Lin, T.; Liu, T.; Lin, Y.; Zhang, C.; Yan, L.; Chen, Z.; He, Z.; Wang, J. Serum levels of chemical elements in esophageal squamous cell carcinoma in Anyang, China: A case-control study based on machine learning methods. *BMJ Open* **2017**, *7,* e015443. doi: 10.1136/bmjopen-2016-015443

69. Park, H.; Kim, K. Comparisons among machine learning models for the prediction of hypercholestrolemia associated with exposure to lead, mercury, and cadmium. *Int. J. Environ. Res. Public Health* **2019**, *16,* 2666. doi: 10.3390/ijerph16152666

70. Cox, L.A. Using Bayesian networks to clarify interpretation of exposure–response regression coefficients: Blood lead–mortality association as an example. *Crit. Rev. Toxicol.* **2020**, *50,* 539–550. doi: 10.1080/10408444.2020.1787329

71. Kim, K.; Park, H. Machine-learning models predicting osteoarthritis associated with the lead blood level. Environ. *Sci. Pollut. Res. Int.* **2021**, *28,* 44079–44084. doi: 10.1007/s11356-021-13887-6

72. Monaco, A.; Lacalamita, A.; Amoroso, N.; D'orta, A.; Del Buono, A.; Di Tuoro, F.; Tangaro, S.; Galeandro, A.I.; Bellotti, R. Random forests highlight the combined effect of environmental heavy metals exposure and genetic damages for cardiovascular diseases. *Applied Sciences* **2021**, *11,* 8405. doi: 10.3390/app11188405

73. Ximenez, J.P.B.; Zamarioli, A.; Kacena, M.A.; Barbosa, R.M.; Barbosa, F. Jr. Association of urinary and blood concentrations of heavy metals with measures of bone mineral density loss: A data mining approach with the results from the National Health and Nutrition Examination Survey. *Biol. Trace Elem. Res.* **2021**, *199,* 92–101. doi: 10.1007/s12011-020-02150-7

74. Liu, A.; Cai, C.; Wang, Z.; Wang, B.; He, J.; Xie, Y.; Deng, H.; Liu, S.; Zeng, S.; Yin, Z.; Wang, M. Inductively coupled plasma mass spectrometry based urine metallome to construct clinical decision models for autism spectrum disorder. *Metallomics* **2022**, *14,* mfac091. doi: 10.1093/mtomcs/mfac091

75. Xia, F.; Li, Q.; Luo, X.; Wu, J. Identification for heavy metals exposure on osteoarthritis among aging people and machine learning for prediction: A study based on NHANES 2011-2020. *Front. Public Health* **2022**, *10,* 906774. doi: 10.3389/fpubh.2022.906774

76. Xia, F.; Li, Q.; Luo, X.; Wu, J. Machine learning model for depression based on heavy metals among aging people: A study with National Health and Nutrition Examination Survey 2017–2018. *Front. Public Health* **2022**, *10,* 939758. doi: 10.3389/fpubh.2022.939758

77. Chan, Y.N.; Wang, P.; Chun, K.H.; Lum, J.T.S.; Wang, H.; Zhang, Y.; Leung, K.S.Y. A machine learning approach for early prediction of gestational diabetes mellitus using elemental contents in fingernails. *Sci. Rep.* **2023**, *13,* 4184. doi: 10.1038/s41598-023-31270-y

78. Chen, Z.; Liu, X.; Wang, W.; Zhang, L.; Ling, W.; Wang, C.; Jiang, J.; Song, J.; Liu, Y.; Lu, D.; Liu, F.; Zhang, A.; Liu, Q.; Zhang, J.; Jiang, G. Machine learning-aided metallomic profiling in serum and urine of thyroid cancer patients and its environmental implications. *Sci. Total Environ.* **2023**, *895,* 165100. doi: 10.1016/j.scitotenv.2023.165100

79. Chen, H.; Wang, M.; Zhang, C.; Li, J. A methodological study of exposome based on an open database: Association analysis between exposure to metal mixtures and hyperuricemia. *Chemosphere* **2023**, *344,* 140318. doi: 10.1016/j.chemosphere.2023.140318

80. Li, W.; Huang, G.; Tang, N.; Lu, P.; Jiang, L.; Lv, J.; Qin, Y.; Lin, Y.; Xu, F.; Lei, D. Effects of heavy metal exposure on hypertension: A machine learning modeling approach. *Chemosphere* **2023**, *337,* 139435. doi: 10.1016/j.chemosphere.2023.139435

81. Li, X.; Zhao, Y.; Zhang, D.; Kuang, L.; Huang, H.; Chen, W.; Fu, X.; Wu, Y.; Li, T.; Zhang, J.; Yuan, L.; Hu, H.; Liu, Y.; Zhang, M.; Hu, F.; Sun, X.; Hu, D. Development of an interpretable machine learning model associated with heavy metals' exposure to identify coronary heart disease among US adults via SHAP: Findings of the US NHANES from 2003 to 2018. *Chemosphere* **2023**, *311,* 137039. doi: 10.1016/j.chemosphere.2022.137039

82. Li, S.; Hu, X. Assessing the risk of prostate cancer with nutritional and environmental factors: A cross-sectional study from National Health and Nutrition Examination Survey 2001–2010. *Nutr. Cancer* **2023**, *75,* 1361–1372. doi: 10.1080/01635581.2023.2197687

83. Ling, W.; Zhao, G.; Wang, W.; Wang, C.; Zhang, L.; Zhang, H.; Lu, D.; Ruan, S.; Zhang, A.; Liu, Q.; Jiang, J.; Jiang, G. Metallomic profiling and natural copper isotopic signatures of childhood autism in serum and red blood cells. *Chemosphere* **2023**, *330,* 138700. doi: 10.1016/j.chemosphere.2023.138700

84. Luo, K.-H.; Wu, C.-H.; Yang, C.-C.; Chen, T.-H.; Tu, H.-P.; Yang, C.-H.; Chuang, H.-Y. Exploring the association of metal mixture in blood to the kidney function and tumor necrosis factor alpha using machine learning methods. *Ecotoxicol. Environ. Saf.* **2023**, *265,* 115528. doi: 10.1016/j.ecoenv.2023.115528

85. Mei, P.; Zhou, Q.; Liu, W.; Huang, J.; Gao, E.; Luo, Y.; Ren, X.; Huang, H.; Chen, X.; Wu, D.; Huang, X.; Yu, H.; Liu, J. Correlating metal exposures and dietary habits with hyperuricemia in a large urban elderly cohort by artificial intelligence. *Environ. Sci. Pollut. Res. Int.* **2023**, *30,* 41570–41580. doi: 10.1007/s11356-022-24824-6

86. Moon, S.; Lee, J.; Yu, J.M.; Choi, H.; Choi, S.; Park, J.; Choi, K.; Kim, E.; Kim, H.; Kim, M.J.; Park, Y.J. Association between environmental cadmium exposure and increased mortality in the U.S. National Health and Nutrition Examination Survey (1999–2018). *J. Expo. Sci. Environ. Epidemiol.* **2023**, *33,* 874–882. doi: 10.1038/s41370-023-00556-8

87. Souza, M.C.O.; Cruz, J.C.; Rocha, B.A.; Souza, J.M.O.; Devóz, P.P.; Santana, A.; Campíglia, A.D.; Barbosa, F. The influence of the co-exposure to polycyclic aromatic hydrocarbons and toxic metals on DNA damage in brazilian lactating women and their infants: A cross-sectional study using machine learning approaches. *Chemosphere* **2023**, *334,* 138975. doi: 10.1016/j.chemosphere.2023.138975

88. Wen, J.; Giri, M.; Xu, L.; Guo, S. Association between exposure to selected heavy metals and blood eosinophil counts in asthmatic adults: Results from NHANES 2011–2018. *J. Clin. Med.* **2023**, *12,* 1543. doi: 10.3390/jcm12041543

89. Wu, Y.; Deng, S. Main heavy metals affecting chronic kidney disease: A study based on feature selection algorithm. In Proceedings Volume 12715, Eighth International Conference on Electronic Technology and Information Science (ICETIS 2023), Dalian, China. doi: 10.1117/12.2682554

90. Zhao, M.; Wan, J.; Qin, W.; Huang, X.; Chen, G.; Zhao, X. A machine learning-based diagnosis modelling of type 2 diabetes mellitus with environmental metal exposure. *Comput. Methods Programs Biomed.* **2023**, *235,* 107537. doi: 10.1016/j.cmpb.2023.107537

91. Angali, K.A.; Farhadi, M.; Neisi, A.; Cheraghian, B.; Ahmadi, M.; Takdastan, A.; Dargahi, A.; Angali, Z.A. Carcinogenic and non-carcinogenic risks caused by rice contamination with heavy metals and their effect on the prevalence of cardiovascular disease (using machine learning). *Food Chem. Toxicol.* **2024**, *194,* 115085. doi: 10.1016/j.fct.2024.115085

92. Bowles, N.P.; He, Y.; Huang, Y.H.; Stecker, E.C.; Seixas, A.; Thosar, S.S. Cardiovascular disease risk: It is complicated, but race and ethnicity are key, a Bayesian network analysis. *Front. Public Health* **2024**, *12,* 1364730. doi: 10.3389/fpubh.2024.1364730

93. Chang, Y.; Jiang, X.; Dou, J.; Xie, R.; Zhao, W.; Cao, Y.; Gao, J.; Yao, F.; Wu, D.; Mei, H.; Zhong, Y.; Ge, Y.; Xu, H.; Jiang, W.; Xiao, X.; Jiang, Y.; Hu, S.; Wu, Y.; Liu, Y. Investigating the potential risk of cadmium exposure on seizure severity and anxiety-like behaviors through the ferroptosis pathway in epileptic mice: An integrated multi-omics approach. *J. Hazard. Mater.* **2024**, *480,* 135814. doi: 10.1016/j.jhazmat.2024.135814

94. Chen, H.; Wang, M.; Li, J. Exploring the association between two groups of metals with potentially opposing renal effects and renal function in middle-aged and older adults: Evidence from an explainable machine learning method. *Ecotoxicol. Environ. Saf.* **2024**, *269,* 115812. doi: 10.1016/j.ecoenv.2023.115812

95. Du, G.; Song, X.; Zhou, F.; Ouyang, L.; Li, Q.; Ruan, S.; Su, R.; Rao, S.; Zhu, Y.; Xie, J.; Feng, C.; Fan, G. Association between multiple metal(loid)s exposure and blood lipid levels: Evidence from a cross-sectional study of Southeastern China. *Biol. Trace Elem. Res.* **2024**, *202,* 3483–3495. doi: 10.1007/s12011-023-03951-2

96. Fan, W.; Pi, Z.; Kong, K.; Qiao, H.; Jin, M.; Chang, Y.; Zhang, J.; Li, H. Analyzing the impact of heavy metal exposure on osteoarthritis and rheumatoid arthritis: An approach based on interpretable machine learning. *Front. Nutr.* **2024**, *11,* 1422617. doi: 10.3389/fnut.2024.1422617

97. Fansler, S.D.; Bakulski, K.M.; Park, S.K.; Walker, E.; Wang, X. Use of biomarkers of metals to improve prediction performance of cardiovascular disease mortality. *Environ. Health* **2024**, *23,* 96. doi: 10.1186/s12940-024-01137-4

98. Gao, X.; Liu, C.; Yin, L.; Wang, A.; Li, J.; Gao, Z. Machine learning model for age-related macular degeneration based on heavy metals: The National Health and Nutrition Examination Survey 2005 to 2008. *Sci. Rep.* **2024**, *14,* 26913. doi: 10.1038/s41598-024-78412-4

99. Gui, Y.; Gui, S.; Wang, X.; Li, Y.; Xu, Y.; Zhang, J. Exploring the relationship between heavy metals and diabetic retinopathy: A machine learning modeling approach. *Sci. Rep.* **2024**, *14,* 13049. doi: 10.1038/s41598-024-63916-w

100. Invernizzi, A.; Renzetti, S.; Rechtman, E.; Ambrosi, C.; Mascaro, L.; Corbo, D.; Gasparotti, R.; Tang, C.Y.; Smith, D.R.; Lucchini, R.G.; Wright, R.O.; Placidi, D.; Horton, M.K.; Curtin, P. Neuro-environmental interactions: A time sensitive matter. *Front. Comput. Neurosci.* **2024**, *17,* 1302010. doi: 10.3389/fncom.2023.1302010

101. Li, B.; Liu, H.; Mishra, D.; Yuan, Z.; Zhang, Y.; Zhang, L.; Huang, Y.; Zhang, Y.; Lin, J.; Chen, J.; Liu, Z. The association between blood metals and cardiovascular diseases: Findings from National Health and Nutrition Examination Survey 2011–2020. *Front. Cardiovasc. Med.* **2024**, *11,* 1479665. doi: 10.3389/fcvm.2024.1479665

102. Liu, J.; Li, X.; Zhu, P. Effects of various heavy metal exposures on insulin resistance in non-diabetic populations: Interpretability analysis from machine learning modeling perspective. *Biol. Trace Elem. Res.* **2024**, *202,* 5438–5452. doi: 10.1007/s12011-024-04126-3

103. Liu, P.; Wang, J.; Mei, P.; Li, J.; Xu, B.; Ren, X.; Chen, X.; Wu, D.; Zhu, F.; Yang, X.; He, M.; Liu, J.; Huang, H. The interaction effect of metals exposure and dietary habit on cognitive function in Chinese older adult cohort. *J. Nutr. Health Aging* **2024**, *28,* 100284. doi: 10.1016/j.jnha.2024.100284

104. Midya, V.; Agrawal, M.; Lane, J.M.; Gennings, C.; Tarassishin, L.; Torres-Olascoaga, L.A.; Eggers, J.; Gregory, J.K.; Picker, M.; Peter, I.; Faith, J.J.; Arora, M.; Téllez-Rojo, M.M.; Wright, R.O.; Colombel, J.-F.; Eggers, S. Association between exposure to metals during pregnancy, childhood gut microbiome, and risk of intestinal inflammation in late childhood. *Environ. Health* **2024**, *2,* 739–749. doi: 10.1021/envhealth.4c00125.

105. Midya, V.; Nagdeo, K.; Lane, J.M.; Torres-Olascoaga, L.A.; Torres-Calapiz, M.; Gennings, C.; Horton, M.K.; Téllez-Rojo, M.M.; Wright, R.O.; Arora, M.; Eggers, S. Prenatal metal exposures and childhood gut microbial signatures are associated with depression score in late childhood. *Sci. Total Environ.* **2024**, *916,* 170361. doi: 10.1016/j.scitotenv.2024.170361

106. Nabavi, A.; Safari, F.; Kashkooli, M.; Nabavizadeh, S.S.; Vardanjani, H.M. Early prediction of cognitive impairment in adults aged 20 years and older using machine learning and biomarkers of heavy metal exposure. *Curr. Res. Toxicol.* **2024**, *7,* 100198. doi: 10.1016/j.crtox.2024.100198.

107. Rog, J.; Łobejko, Ł.; Hordejuk, M.; Marciniak, W.; Derkacz, R.; Kiljańczyk, A.; Matuszczak, M.; Lubiński, J.; Nesterowicz, M.; Żendzian-Piotrowska, M.; Zalewska, A.; Maciejczyk, M.; Karakula-Juchnowicz, H. Pro/antioxidant status and selenium, zinc and arsenic concentration in patients with bipolar disorder treated with lithium and valproic acid. *Front. Mol. Neurosci.* **2024**, *17,* 1441575. doi: 10.3389/fnmol.2024.1441575

108. Su, Z.; Zhang, Y.; Hong, S.; Zhang, Q.; Ji, Z.; Hu, G.; Zhu, X.; Yuan, F.; Yu, S.; Wang, T.; Wang, L.; Jia, G. Immune regulation patterns in response to environmental pollutant chromate exposure-related genetic damage: A cross-sectional study applying machine learning methods. *Environ. Sci. Technol.* **2024**, *58,* 7279–7290. doi: 10.1021/acs.est.4c00433

109. Wang, X.; Wang, X.; Cheng, Y.; Luo, C.; Xia, W.; Gao, Z.; Bu, W.; Jiang, Y.; Fei, Y.; Shi, W.; Tang, J.; Liu, L.; Zhu, J.; Zhao, X. Construction of metal interpretable scoring system and identification of tungsten as a novel risk factor in COPD. *Ecotoxicol. Environ. Saf.* **2024**, *283,* 116842. doi: 10.1016/j.ecoenv.2024.116842

110. Wu, M.; Hou, W.; Qin, R.; Wang, G.; Sun, D.; Geng, Y.; Du, Y. Comparative mathematical modeling of causal association between metal exposure and development of chronic kidney disease. *Front. Endocrinol.* **2024**, *15,* 1362085. doi: 10.3389/fendo.2024.1362085

111. Yan, L.; You, H.; Wang, H.; Ding, C.; He, B.; Wang, J.; Fang, W.; Lin, Y.; Kang, D.; Chen, F. Association of multiple trace metals in scalp hair with glioma risk: The mediating role of inflammation. *Ann. Clin. Transl. Neurol.* **2024**, *11,* 2987–2997. doi: 10.1002/acn3.52210

112. Yao, J.; Du, Z.; Yang, F.; Duan, R.; Feng, T. The relationship between heavy metals and metabolic syndrome using machine learning. *Front. Public Health* **2024**, *12,* 1378041. doi: 10.3389/fpubh.2024.1378041

113. Xiao, H.; Liang, X.; Li, H.; Chen, X.; Li, Y. Trends in the prevalence of osteoporosis and effects of heavy metal exposure using interpretable machine learning. *Ecotoxicol. Environ. Saf.* **2024**, *286,* 117238. doi: 10.1016/j.ecoenv.2024.117238

114. Xu, S.; Sun, M. The interpretable machine learning model associated with metal mixtures to identify hypertension via EMR mining method. *J. Clin. Hypertens.* **2024**, *26,* 187–196. doi: 10.1111/jch.14768

115. Xu, S.; Sun, M. Assessment of EMR ML mining methods for measuring association between metal mixture and mortality for hypertension. *High Blood Press. Cardiovasc. Prev.* **2024**, *31,* 473–483. doi: 10.1007/s40292-024-00666-w

116. Zhao, R.; Lin, S.; Han, M.; Lin, Z.; Yu, M.; Zhang, B.; Ma, L.; Li, D.; Peng, L. Association between machine learning-assisted heavy metal exposures and diabetic kidney disease: A cross-sectional survey and Mendelian randomization analysis. *Front. Public Health* **2024**, *12,* 1367061. doi: 10.3389/fpubh.2024.1367061

117. Zibibula, Y.; Tayier, G.; Maimaiti, A.; Liu, T.; Lu, J. Machine learning approaches to identify the link between heavy metal exposure and ischemic stroke using the US NHANES data from 2003 to 2018. *Front. Public Health* **2024**, *12,* 1388257. doi: 10.3389/fpubh.2024.1388257

118. Zuo, W.; Yang, X. A machine learning model predicts stroke associated with blood cadmium level. *Sci. Rep.* **2024**, *14,* 14739. doi: 10.1038/s41598-024-65633-w

119. Zuo, W.; Yang, X. Correction to: A machine learning model predicts stroke associated with blood cadmium level. *Sci. Rep.* **2024**, *14,* 18502. doi: 10.1038/s41598-024-69583-1.

120. Chen, J. Development of a machine learning model related to explore the association between heavy metal exposure and alveolar bone loss among US adults utilizing SHAP: A study based on NHANES 2015–2018. *BMC Public Health* **2025**, *25,* 455. doi: 10.1186/s12889-025-21658-y

121. Chen, J.; Zeng, H.; Pan, Z.; Li, M.; Zhou, Q.; Chen, K.; Hao, Y.; Cao, X.; Zhang, L.; Wang, Q. Association between metal mixture in urine and abnormal blood pressure and mediated effect of oxidative stress based on BKMR and machine learning method. *Ecotoxicol. Environ. Saf.* **2025**, *301,* 118478. doi: 10.1016/j.ecoenv.2025.118478

122. Cox, L.A. Jr.; Lewis, R.J.; Rege, S. V.; Singh, S. AI-assisted exposure-response data analysis: Quantifying heterogeneous causal effects of exposures on survival times. *Glob. Epidemiol.* **2025**, *9,* 100179. doi: 10.1016/j.gloepi.2024.100179

123. Gu, X.; Li, Q.; Wang, X. Using Life's Essential 8 and heavy metal exposure to determine infertility risk in American women: A machine learning prediction model based on the SHAP method. *Front. Endocrinol.* **2025**, *16,* 1586828. doi: 10.3389/fendo.2025.1586828

124. He, J.; Zhou, W.; Zhang, H.; Shen, J. In vivo heavy metal and diabetes association: A cross-sectional interpretable machine learning analysis of NHANES. *Int. J. Diabetes Dev. Ctries.* **2025**. doi: 10.1007/s13410-025-01524-8

125. Hu, H.; Wu, Y.; Liu, J.; Zhao, M.; Xie, P. The relationship between metal exposure and HPV infection: Evidence from explainable machine learning methods. *Biol. Trace Elem. Res.* **2025**, *203,* 2206–2215. doi: 10.1007/s12011-024-04322-1

126. Jin, H.; Zhang, L.; Sun, Y.; Xu, Y.; Luo, M. Developing machine learning models for predicting cardiovascular disease survival based on heavy metal serum and urine levels. *Front. Public Health* **2025**, *13,* 1582779. doi: 10.3389/fpubh.2025.1582779

127. Jin, X.; Li, L.; Hu, X.; Bi, P.; Zhang, S.; Wang, Q.; Xiao, Z.; Yang, H.; Liu, T.; Feng, L.; Wang, J. Association of urinary metal elements with sarcopenia and glucose metabolism abnormalities: Insights from NHANES data using machine learning approaches. *Ecotoxicol. Environ. Saf.* **2025**, *300,* 118469. doi: 10.1016/j.ecoenv.2025.118469

128. Johnson, H.; Longden, J.; Cameron, G.; Waiter, G.D.; Waldron, F.M.; Gregory, J.M.; Spence, H. Machine learning identifies routine blood tests as accurate predictive measures of pollution-dependent poor cognitive function. *bioRxiv* [preprint] **2025**. doi: 10.1101/2025.01.10.632396

129. Li, G.; Zhang, T.; He, K.; Zhang, M.; Hu, J.; Ge, T.; Wang, M.; Zou, R.; Fan, X. Deciphering the influence of heavy metals on adverse cardiovascular events using machine learning. *Toxicol. Lett.* **2025**, *411,* 101–110. doi: 10.1016/j.toxlet.2025.07.1414

130. Li, Y.; Tao, R.; Feng, Z.; Mei, G.; Liu, Z.; Yang, W.; Mo, F.; Liu, Z. Exploring the association between heavy metal exposure and periodontitis using interpretable machine learning models: NHANES 2009–2014. *Hum. Ecol. Risk Assess.* **2025**, *31*, 1084–1099. doi: 10.1080/10807039.2025.2530028

131. Mi, Y.; Sun, P. Machine learning-based prediction of hearing loss: Findings of the US NHANES from 2003 to 2018. *Hear. Res.* **2025**, *461*, 109252. doi: 10.1016/j.heares.2025.109252

132. Nabavi, A.; Kashkooli, M.; Nabavizadeh, S.S.; Safari, F. Heavy metal biomarkers and their impact on hearing loss risk: A machine learning framework analysis. *Front. Public Health* **2025**, *13*, 1471490. doi: 10.3389/fpubh.2025.1471490

133. Nahar, S.; Wang, Z.J.; Chakrabarty, S.; Jin, X. Leveraging selected lifestyle factors from NHANES data for chronic heart disease risk prediction. In 2025 Intermountain Engineering, Technology and Computing (IETC), UT, United States, 2025. doi: 10.1109/IETC64455.2025.11039383

134. Ren, F.; Zhao, X.; Yang, Q.; Liao, H.; Zhang, Y.; Liu, X. A machine learning framework for predicting cognitive impairment in aging populations using urinary metal and demographic data. *Front. Genet.* **2025**, *16*, 1631228. doi: 10.3389/fgene.2025.1631228

135. Shen, M.; Zhang, Y.; Zhan, R.; Du, T.; Shen, P.; Lu, X.; Liu, S.; Guo, R.; Shen, X. Predicting the risk of cardiovascular disease in adults exposed to heavy metals: Interpretable machine learning. *Ecotoxicol. Environ. Saf.* **2025**, *290*, 117570. doi: 10.1016/j.ecoenv.2024.117570

136. Shi, C.; Jiang, H.; Zhao, F.; Zhang, Y.; Chen, H. Blood metal levels predict digestive tract cancer risk using machine learning in a U.S. cohort. *Sci. Rep.* **2025**, *15*, 1285. doi: 10.1038/s41598-025-85659-y

137. Wan, S.; Yang, Y.; Zhao, Q.; Xing, Z.; Li, J.; Gao, H.; Yin, Y.; Liu, Z.; Chen, Q.; Tian, M.; Shi, X.; Ji, Z.; Huang, S. Proteomic signatures and predictive modeling of cadmium-associated anxiety in middle-aged and elderly populations: an environmental exposure association study. *J. Transl. Med.* **2025**, *23*, 499. doi: 10.1186/s12967-025-06466-7

138. Wang, X.; Chen, G.; He, R.; Gao, Y.; Lu, J.; Xu, T.; Liu, H.; Jiang, Z. Machine learning prediction of glaucoma by heavy metal exposure: Results from the National Health and Nutrition Examination Survey 2005 to 2008. *Sci. Rep.* **2025**, *15*, 4891. doi: 10.1038/s41598-025-88698-7

139. Wen, J.; Wang, C.; Liu, R.; Zhuang, R.; Liu, Y.; Li, Y.; Guo, S. Systemic inflammation mediates the relationship between urinary cadmium and chronic cough risk: Findings based on multiple statistical models. *Biometals* **2025**, *38*, 983–995. doi: 10.1007/s10534-025-00690-w

140. Wu, Z.; Jiang, S.; Li, J.; Wang, P.; Chen, Y. Association between urinary cadmium levels and increased gallstone disease in US adults. *Sci. Rep.* **2025**, *15*, 15974. doi: 10.1038/s41598-025-00648-5

141. Xia, T.; Han, K. Machine learning prediction model with shap interpretation for chronic bronchitis risk assessment based on heavy metal exposure: A nationally representative study. *BMC Pulm. Med.* **2025**, *25*, 252. doi: 10.1186/s12890-025-03724-8

142. Xu, S.; Sun, M. The interpretable machine learning model for depression associated with heavy metals via EMR mining method. *Sci. Rep.* **2025**, *15,* 10811. doi: 10.1038/s41598-025-95938-3

143. You, Z.-M.; Li, Y.-S.; Meng, F.-S.; Zhang, R.-X.; Xie, C.-X.; Liang, Z.; Zhou, J.-Y. Interpretable machine learning approaches for predicting prostate cancer by using multiple heavy metal exposures based on the data from NHANES 2003–2018. *Ecotoxicol. Environ. Saf.* **2025**, *302,* 118730. doi: 10.1016/j.ecoenv.2025.118730

144. Yuting, Y.; Shan, D. Associations between urinary and blood heavy metal exposure and heart failure in elderly adults: Insights from an interpretable machine learning model based on NHANES (2003–2020). *Int. J. Cardiol. Cardiovasc. Risk Prev.* **2025**, *25,* 200418. doi: 10.1016/j.ijcrp.2025.200418

145. Zhang, Y.; Li, Q.; Wang, X. Associations between exposure to heavy metal and sarcopenia prevalence: A cross-sectional study using NHANES data. *Front. Public Health* **2025**, *13,* 1588041. doi: 10.3389/fpubh.2025.1588041

146. Zhang, N.; Xu, Y.; Liang, H.; Wang, Q.; An, Y.; Gao, H.; Zhao, J.; Wang, H. Diagnostic value of small dense low-density lipoprotein and trace elements in coronary artery disease. *Ann. Biol. Clin.* **2025**, *83,* 161–175. doi: 10.1684/abc.2025.1960

147. Zhong, Y.; Bao, Y.; Cheng, H.; Liu, C.; Huang, S.; Qiu, H.; Huang, H.; Ren, J.; Jin, H.; He, C.; Tian, L.; Zhang, Y.; Luo, B.; Liang, T.; Li, M.; Mo, Z.; Li, L.; Yang, X. A negative combined effect of exposure to maternal Mn-Cu-Rb-Fe metal mixtures on gestational anemia, and the mediating role of creatinine in the Guangxi Birth Cohort Study (GBCS): Twelve machine learning algorithms. *Ecotoxicol. Environ. Saf.* **2025**, *300,* 118418. doi: 10.1016/j.ecoenv.2025.118418

148. Feng, Y.; Su, S.; Lin, W.; Ren, M.; Gao, N.; Pan, B.; Zhang, L.; Jin, L.; Zhang, Y.; Li, Z.; Ye, R.; Ren, A.; Wang, B. Using machine learning to expedite the screening of environmental factors associated with the risk of spontaneous preterm birth: From exposure mixtures to key molecular events. *Environ. Sci. Technol. Lett.* **2023**, *10,* 1036–1044. doi: 10.1021/acs.estlett.3c00085

149. Eve, A.A.; Tunc, E.; Mehta, D.; Yoo, J.Y.; Yilmaz, H.E.; Emren, S.V.; Akçay, F.A.; Erdogan, Z.M. PFAS and their association with the increased risk of cardiovascular disease in postmenopausal women. *Toxicol. Sci.* **2024**, *200,* pp. 312–323. doi: 10.1093/toxsci/kfae065

150. Li, Z.; Xu, X.; Zhang, K. Exploring the relationship between per- and polyfluoroalkyl substances exposure and rheumatoid arthritis risk using interpretable machine learning. *Front. Public Health* **2025**, *13,* 1581717. doi: 10.3389/fpubh.2025.1581717

151. Shao, X.; Zhang, L.; Wang, Y.; Ying, Y.; Chen, X. Developing an interpretable machine learning predictive model of chronic obstructive pulmonary disease by serum PFAS concentration. *Front. Public Health* **2025**, *13,* 1602566. doi: 10.3389/fpubh.2025.1602566

152. Wang, F.; Lin, Y.; Qin, L.; Zeng, X.; Jiang, H.; Liang, Y.; Wen, S.; Li, X.; Huang, S.; Li, C.; Luo, X.; Yang, X. Serum metabolome associated with novel and legacy per- and polyfluoroalkyl substances exposure and thyroid cancer risk: A multi-module integrated analysis based on machine learning. *Environ. Int.* **2025**, *195,* 109203. doi: 10.1016/j.envint.2024.109203

153. Wang, C.; Xu, X.; Luo, S.; Luo, M.; Li, S.; Si, J. Interpretable machine learning insights into the association between PFAS exposure and diabetes mellitus. *Ecotoxicol. Environ. Saf.* **2025**, *302,* 118569. doi: 10.1016/j.ecoenv.2025.118569

154. Yang, J.; Wang, T.; Li, K.; Wāng, Y. Associations between per- and polyfluoroalkyl chemicals and abdominal aortic calcification in middle-aged and older adults. *J. Adv. Res.* **2025**, *70,* 203–222. doi: 10.1016/j.jare.2024.04.022

155. Zheng, Z.; Xu, Y.; Kang, N.; Dong, Y.; Wang, X.; Zhang, L.; Bian, H.; Zeng, Q. Assessing the effect of perfluoroalkyl and polyfluoroalkyl substances on cardiovascular-kidney-metabolic syndrome: Insights from an interpretable machine learning model. *Sci. Total Environ.* **2025**, *993,* 180003. doi: 10.1016/j.scitotenv.2025.180003

156. Colicino, E.; de Water, E.; Just, A.C.; Navarro, E.; Pedretti, N.F.; McRae, N.; Braun, J.M.; Schnaas, L.; Rodríguez-Carmona, Y.; Hernández, C.; Tamayo-Ortiz, M.; Téllez-Rojo, M.M.; Deierlein, A.L.; Calafat, A.M.; Baccarelli, A.; Wright, R.O.; Horton, M.K. Prenatal urinary concentrations of phthalate metabolites and behavioral problems in Mexican children: The Programming Research in Obesity, Growth Environment and Social Stress (PROGRESS) study. *Environ. Res.* **2021**, *201,* 111338. doi: 10.1016/j.envres.2021.111338

157. Lu, L.; Qian, Y.; Dong, Y.; Su, H.; Deng, Y.; Zeng, Q.; Li, H. A systematic study of the performance of machine learning models on analyzing the association between semen quality and environmental pollutants. *Front. Phys.* **2023**, *11,* 1259273. doi: 10.3389/fphy.2023.1259273

158. You, L.-M.; Zhang, D.-C.; Lin, C.-S.; Lan, Q. Phthalate metabolites were related to the risk of high-frequency hearing loss: A cross-sectional study of National Health and Nutrition Examination Survey. *J. Multidiscip. Healthc.* **2024**, *17,* 5151–5161. doi: 10.2147/JMDH.S481288

159. Liu, Y.; Li, K.; Zhang, Y.; Cai, Y.; Liu, X.; Jia, Y.; Yao, P.; Wei, X.; Wu, H.; Liu, X.; Feng, C.; Li, C.; Wang, W.; Zhang, S.; Du, C. Impact of phthalate exposure and blood lipids on breast cancer risk: Machine learning prediction. *Environ. Sci. Eur.* **2025**, *37,* 39. doi: 10.1186/s12302-025-01071-3

160. Wu, H.-T.; Liao, C.-C.; Peng, C.-F.; Lee, T.-Y.; Liao, P.-H. Exploring the application of machine learning to identify the correlations between phthalate esters and disease: Enhancing nursing assessments. *Health Inf. Sci. Syst.* **2025**, *13,* 10. doi: 10.1007/s13755-024-00324-4

161. Oh, R.; Lee, H.K.; Pak, Y.K.; Oh, M.-S. An interactive online app for predicting diabetes via machine learning from environment-polluting chemical exposure data. *Int. J. Environ. Res. Public Health* **2022**, *19,* 5800. doi: 10.3390/ijerph19105800

162. Sharma, A.; Hooda, N.; Gupta, N.R. Breast cancer recurrence prediction in biopsy using machine learning framework. In *Advances in Communication and Computational Technology (ICACCT 2019). Lecture notes in electrical engineering*; Hura, G.S; Singh, A.K.; Siong Hoe, L., Eds.; Springer: Singapore, 2021, 668. https://link.springer.com/chapter/10.1007/978-981-15-5341-7_28

163. Sharma, A.; Hooda, N.; Gupta, N.R.; Sharma, R. Efficient BREV ensemble framework: A case study of breast cancer prediction. In *Image Based Computing for Food and Health Analytics: Requirements, Challenges, Solutions and Practices*; Tiwari, R.; Koundal, D.; Upadhyay, S., Eds.; Springer Nature: Switzerland, 2023, pp. 97-113.

164. Gao, Y.; Lu, H.; Zhou, H.; Tan, J. Exploring the impact of polychlorinated biphenyls on comorbidity and potential mitigation strategies. *Front. Public Health* **2024**, *12,* 1474994. doi: 10.3389/fpubh.2024.1474994

165. Liu, Y.; Li, K.; Li, C.; Feng, Z.; Cai, Y.; Zhang, Y.; Hu, Y.; Wei, X.; Yao, P.; Liu, X.; Jia, Y.; Lv, W.; Zhang, Y.; Zhou, Z.; Wu, F.; Yan, W.; Zhang, S.; Du, C. Pesticides, cancer, and oxidative stress: An application of machine learning to NHANES data. *Environ. Sci. Eur.* **2024**, *36,* 8. doi: 10.1186/s12302-023-00834-0

166. Tan, J.; Ma, M.; Shen, X.; Xia, Y.; Qin, W. Potential lethality of organochlorine pesticides: Inducing fatality through inflammatory responses in the organism. *Ecotoxicol. Environ. Saf.* **2024**, *279,* 116508. doi: 10.1016/j.ecoenv.2024.116508

167. Jiang, Y.-X.; Gui, S.-Y.; Sun, X.-D. Associations between organophosphorus pesticides exposure and age-related macular degeneration risk in U.S. adults: Analysis from interpretable machine learning approaches. *Int. J. Ophthalmol.* **2025**, *18,* 1214–1230. doi: 10.18240/ijo.2025.07.04

168. Liu, J.; Wang, B.; Li, Q. Machine learning model for age related macular degeneration based on pesticides: The National Health and Nutrition Examination Survey 2007–2008. *Front. Public Health* **2025**, *13,* 1561913. doi: 10.3389/fpubh.2025.1561913

169. Pan, D.; Zhou, L.; Mu, C.; Lin, M.; Sheng, Y.; Xu, Y.; Huang, D.; Liu, S.; Zeng, X.; Chongsuvivatwong, V.; Qiu, X. Effects of neonicotinoid pesticide exposure in the first trimester on gestational diabetes mellitus based on interpretable machine learning. *Environ. Res.* **2025**, *273,* 121168. doi: 10.1016/j.envres.2025.121168

170. Shamma, S.; Hussein, M.A.; El-Nahrery, E.M.A.; Shahat, A.; Shoeib, T.; Abdelnaser, A. Leveraging machine learning in precision medicine to unveil organochlorine pesticides as predictive biomarkers for thyroid dysfunction. *Sci. Rep.* **2025**, *15,* 12501. doi: 10.1038/s41598-025-94827-z

171. Wang, X.; Tian, M.; Shen, Z.; Tian, K.; Fei, Y.; Cheng, Y.; Ruan, J.; Mo, S.; Dai, J.; Xia, W.; Jiang, M.; Zhao, X.; Zhu, J.; Xiao, J. Comprehensive cross-sectional study of the triglyceride glucose index, organophosphate pesticide exposure, and cardiovascular diseases: A machine learning integrated approach. *Toxics* **2025**, *13,* 118. doi: 10.3390/toxics13020118

172. Yang, X.; Zhang, Y.; Xu, Y.; Xu, Y.; Zhang, M.; Guan, Q.; Hu, W.; Tun, H.M.; Xia, Y. Microbial disturbances caused by pesticide exposure and their predictive implications for gestational diabetes mellitus. *Environ. Sci. Technol.* **2025**, *59,* 9449–9460. doi: 10.1021/acs.est.5c01076

173. Fu, Q.; Wu, Y.; Zhu, M.; Xia, Y.; Yu, Q.; Liu, Z.; Ma, X.; Yang, R. Identifying cardiovascular disease risk in the U.S. population using environmental volatile organic compounds exposure: A machine learning predictive model based on the SHAP methodology. *Ecotoxicol. Environ. Saf.* **2024**, *286,* 117210. doi: 10.1016/j.ecoenv.2024.117210

174. Liu, X.; Chang, Y.; Xu, C.; Li, Y.; Wang, Y.; Sun, Y.; Duan, M.; Li, W.; Cui, J. Association of volatile organic compound levels with chronic obstructive pulmonary diseases in NHANES 2013–2016. *Sci. Rep.* **2024**, *14,* 16085. doi: 10.1038/s41598-024-67210-7

175. Deng, C.; Jiang, Y.; Lin, Y.; Liang, H.; Wang, W.; Huang, Y.; He, J. Exploring the potential associations between single and mixed volatile compounds and preserved ratio impaired spirometry using five different approaches. *Ecotoxicol. Environ. Saf.* **2025**, *302,* 118686. doi: 10.1016/j.ecoenv.2025.118686

176. Jiang, L.; Wang, H.; Xiao, Y.; Xu, L.; Chen, H. Exploring the association between volatile organic compound exposure and chronic kidney disease: Evidence from explainable machine learning methods. *Ren. Fail.* **2025**, *47,* 2520906. doi: 10.1080/0886022X.2025.2520906

177. Zhang, Y.; Qiu, X.; Wu, Z.; Li, Y.; Shen, X.; Wu, J.; Cao, P.; Sun, Z.; Wang, W. Comprehensive analysis of the association between volatile organic compound pollutants and chronic kidney disease in hypertensive populations: Insights from multi-omics approaches and identification of potential therapeutic targets. *Environ. Res.* **2025**, *282,* 121966. doi: 10.1016/j.envres.2025.121966

178. Chen, Y.; Shen, X.; Li, G.; Yue, S.; Liang, C.; Hao, Z. Association between aldehyde exposure and kidney stones in adults. *Front. Public Health* **2022**, *10,* 978338. doi: 10.3389/fpubh.2022.978338

179. Liu, L.; Zhou, H.; Wang, X.; Wen, F.; Zhang, G.; Yu, J.; Shen, H.; Huang, R. Effects of environmental phenols on eGFR: Machine learning modeling methods applied to cross-sectional studies. *Front. Public Health* **2024**, *12,* 1405533. doi: 10.3389/fpubh.2024.1405533

180. Pala, D.; Xu, J.; Xie, Y.; Zhang, Y.; Shen, L. Identifying biological markers and sociodemographic factors that influence the gap between phenotypic and chronological ages. *Inform. Health Soc. Care* **2024**, *49,* 162–176. doi: 10.1080/17538157.2024.2400247

181. Cai, Y.; Huang, X.-R.; Wang, S.-J.; Liang, Y.-C.; Liu, D.-L.; Chu, S.-F.; Li, H.-L. Effect of the exposure to brominated flame retardants on hyperuricemia using interpretable machine learning algorithms based on the SHAP methodology. *PLoS One* **2025**, *20,* e0325896. doi: 10.1371/journal.pone.0325896

182. Mulisa, G.; Pero-Gascon, R.; McCormack, V.; Bisanz, J.E.; Talukdar, F.R.; Abebe, T.; De Boevre, M.; De Saeger, S. Multiple mycotoxin exposure assessment through human biomonitoring in an esophageal cancer case-control study in the Arsi-Bale districts of Oromia region of Ethiopia. *Int. J. Hyg. Environ. Health* **2025**, *263,* 114466. doi: 10.1016/j.ijheh.2024.114466

183. Tian, Y.; Gao, S.; Zhang, F.; Wan, X.; Jia, W.; Jiao, J.; Fan, Y.; Zhang, Y. A new method for internal urinary metabolite exposure and dietary exposure association assessment of 3-MCPD and glycidol and their esters based on machine learning. *Ecotoxicol. Environ. Saf.* **2025**, *302,* 118550. doi: 10.1016/j.ecoenv.2025.118550

184. Xie, Q.; Qu, H.; Li, J.; Zeng, R.; Li, W.; Ouyang, R.; Zhang, C.; Xie, S.; Du, M. Identifying emphysema risk using brominated flame retardants exposure: A machine learning predictive model based on the SHAP methodology. *Front. Public Health* **2025**, *13,* 1600729. doi: 10.3389/fpubh.2025.1600729

185. Xu, Q.; Si, K.; Wang, W.; Li, Y.; Zhang, Y.; Gaskins, A.J.; Messerlian, C.; Mustieles, V.; Xiong, C.-L.; Pan, A.; Shao, H.; Mei, S.; Wang, Y.-X. Organophosphate flame retardants, obesity, and depressive symptoms among 1019 young healthy men. *Environ. Health* **2025**, *3,* 1220–1230. doi: 10.1021/envhealth.5c00051

186. Krysiak-Baltyn, K.; Toppari, J.; Skakkebaek, N.E.; Jensen, T.S.; Virtanen, H.E.; Schramm, K.W.; Shen, H.; Vartiainen, T.; Kiviranta, H.; Taboureau, O.; Audouze, K.; Brunak, S.; Main, K.M. Association between chemical pattern in breast milk and congenital cryptorchidism: Modelling of complex human exposures. *Int. J. Androl.* **2012**, *35,* 294–302. doi: 10.1111/j.1365-2605.2012.01268.x

187. Oskar, S.; Wolff, M.S.; Teitelbaum, S.L.; Stingone, J.A. Identifying environmental exposure profiles associated with timing of menarche: A two-step machine learning approach to examine multiple environmental exposures. *Environ. Res.* **2021**, *195,* 110524. doi: 10.1016/j.envres.2020.110524

188. Wei, H.; Sun, J.; Shan, W.; Xiao, W.; Wang, B.; Ma, X.; Hu, W.; Wang, X.; Xia, Y. Environmental chemical exposure dynamics and machine learning-based prediction of diabetes mellitus. *Sci. Total Environ.* **2022**, *806,* 150674. doi: 10.1016/j.scitotenv.2021.150674

189. Li, W.; Huang, G.; Tang, N.; Lu, P.; Jiang, L.; Lv, J.; Qin, Y.; Lin, Y.; Xu, F.; Lei, D. Association between co-exposure to phenols, phthalates, and polycyclic aromatic hydrocarbons with the risk of frailty. *Environ. Sci. Pollut. Res. Int.* **2023**, *30,* 105181–105193. doi: 10.1007/s11356-023-29887-7

190. Llopis, M.; Ventura, P.S.; Brachowicz, N.; Sangüesa, J.; Murcia, M.; Lopez-Espinosa, M.J.; García-Baquero, G.; Lertxundi, A.; Vrijheid, M.; Casas, M.; Petrone, P. Sociodemographic, lifestyle, and environmental determinants of vitamin D levels in pregnant women in Spain. *Environ. Int.* **2023**, *182,* 108293. doi: 10.1016/j.envint.2023.108293

191. Soomro, M.H.; England-Mason, G.; Liu, J.; Reardon, A.J.F.; MacDonald, A.M.; Kinniburgh, D.W.; Martin, J.W.; Dewey, D. Associations between the chemical exposome and pregnancy induced hypertension. *Environ. Res.* **2023**, *237,* 116838. doi: 10.1016/j.envres.2023.116838

192. Duan, S.; Wu, Y.; Zhu, J.; Wang, X.; Zhang, Y.; Gu, C.; Fang, Y. Development of interpretable machine learning models associated with environmental chemicals to predict all-cause and specific-cause mortality: A longitudinal study based on NHANES. *Ecotoxicol. Environ. Saf.* **2024**, *270,* 115864. doi: 10.1016/j.ecoenv.2023.115864

193. Feng, Z.; Chen, Y.; Guo, Y.; Lyu, J. Deciphering the environmental chemical basis of muscle quality decline by interpretable machine learning models. *Am. J. Clin. Nutr.* **2024**, *120,* 407–418. doi: 10.1016/j.ajcnut.2024.05.022

194. Guo, K.; Ni, W.; Du, L.; Zhou, Y.; Cheng, L.; Zhou, H. Environmental chemical exposures and a machine learning-based model for predicting hypertension in NHANES 2003–2016. *BMC Cardiovasc. Disord.* **2024**, *24,* 544. doi: 10.1186/s12872-024-04216-z

195. Liu, S.; Lu, L.; Wang, F.; Han, B.; Ou, L.; Gao, X.; Luo, Y.; Huo, W.; Zeng, Q. Building a predictive model for hypertension related to environmental chemicals using machine learning. *Environ. Sci. Pollut. Res. Int.* **2024**, *31,* 4595–4605. doi: 10.1007/s11356-023-31384-w

196. Soomro, M.H.; England-Mason, G.; Reardon, A.J.F.; Liu, J.; MacDonald, A.M.; Kinniburgh, D.W.; Martin, J.W.; Dewey, D. Maternal exposure to bisphenols, phthalates, perfluoroalkyl acids, and trace elements and their associations with gestational diabetes mellitus in the APrON cohort. *Reprod. Toxicol.* **2024**, *127,* 108612. doi: 10.1016/j.reprotox.2024.108612

197. Yang, L.; Zhang, T.; Gao, Y.; Li, D.; Cui, R.; Gu, C.; Wang, L.; Sun, H. Quantitative identification of the co-exposure effects of e-waste pollutants on human oxidative stress by explainable machine learning. *J. Hazard. Mater.* **2024**, *466,* 133560. doi: 10.1016/j.jhazmat.2024.133560

198. Deng, C.; Jiang, Y.; Lin, Y.; Liang, H.; Wang, W.; Huang, Y.; He, J. Potential effects of endocrine-disrupting chemicals on preserved ratio impaired spirometry revealed by five different approaches. *Ecotoxicol. Environ. Saf.* **2025**, *302,* 118701. doi: 10.1016/j.ecoenv.2025.118701

199. England-Mason, G.; MacEachern, S.J.; Amador, K.; Soomro, M.H.; Reardon, A.J.F.; MacDonald, A.M.; Kinniburgh, D.W.; Letourneau, N.; Giesbrecht, G.F.; Martin, J.W.; Forkert, N.D.; Dewey, D. Using machine learning to investigate the influence of the prenatal chemical exposome on neurodevelopment of young children. *Neurotoxicology* **2025**, *108,* 218–230. doi: 10.1016/j.neuro.2025.04.001

200. Jo, Y.; Shin, M.Y.; Kim, S. Assessing the association of multi-environmental chemical exposures on metabolic syndrome: A machine learning approach. *Environ. Int.* **2025**, *199,* 109481. doi: 10.1016/j.envint.2025.109481

201. Lee, I.; Noh, J.; Kim, Y.; An, J.N.; Park, J.Y.; Kim, Y.C.; Lee, J.; Lee, J.P.; Lee, J.S.; Choi, K.; Yoo, K.D. A novel approach to the relation of multi-pollutant effect and kidney dysfunction: Data analysis from the Korean National Environmental Health Survey Cycle 3 (2015–2017). *Kidney Res. Clin. Pract.* **2025**. doi: 10.23876/j.krcp.24.173

202. Liu, H.; Gu, H.; Li, J.; Fang, Y.; Yang, S.; Liang, G. Evaluating the relationship between environmental chemicals and obesity: Evidence from a machine learning perspective. *Ecotoxicol. Environ. Saf.* **2025**, *300,* 118457. doi: 10.1016/j.ecoenv.2025.118457

203. Liu, S.; Wang, H.; Cao, Y.; Lu, L.; Wu, Y.; Lian, F.; Yang, J.; Song, Q. The association between low-concentration heavy metal exposure and chronic kidney disease risk through α-klotho. *Sci. Rep.* **2025**, *15,* 11320. doi: 10.1038/s41598-025-96016-4

204. Lu, X.; Kou, H.; Li, C.; Zhan, R.; Guo, R.; Liu, S.; Shen, P.; Shen, M.; Du, T.; Lu, J.; Shen, X. Development and validation of an interpretable machine learning model for predicting hyperuricemia risk: Based on environmental chemical exposure. *Ecotoxicol. Environ. Saf.* **2025**, *299,* 118392. doi: 10.1016/j.ecoenv.2025.118392

205. Shi, Y.; Li, K.; Ding, R.; Li, X.; Cheng, Z.; Liu, J.; Liu, S.; Zhu, H.; Sun, H. Untargeted metabolomics and machine learning unveil the exposome and metabolism linked with the risk of early pregnancy loss. *J. Hazard. Mater.* **2025**, *488,* 137362. doi: 10.1016/j.jhazmat.2025.137362

206. Yan, C.; Zhu, Z.; Guo, X.; Zong, W.; Liu, G.; Jin, Y.; Cui, S.; Liu, F.; Gao, S. The impact of multipollutant exposure on hepatic steatosis: A machine learning-based investigation into multipollutant synergistic effects. *Front. Public Health* **2025**, *13,* 1598639. doi: 10.3389/fpubh.2025.1598639

207. Yang, L.; Gao, C.; Qin, R.; Wu, Q.; Sun, H.; Zhang, T. Impact of e-waste pollutant exposure on renal injury and oxidative stress biomarkers: Evidence from causal machine learning. *J. Hazard. Mater.* **2025**, *495,* 138831. doi: 10.1016/j.jhazmat.2025.138831

208. Zhang, B.; Chen, L.; Li, T. Unveiling the effect of urinary xenoestrogens on chronic kidney disease in adults: A machine learning model. *Ecotoxicol. Environ. Saf.* **2025**, *292,* 117945. doi: 10.1016/j.ecoenv.2025.117945

209. Park, S.K.; Zhao, Z.; Mukherjee, B. Construction of environmental risk score beyond standard linear models using machine learning methods: Application to metal mixtures, oxidative stress and cardiovascular disease in NHANES. *Environ. Health* **2017**, *16,* 102. doi: 10.1186/s12940-017-0310-9

210. Luo, J.; Hendryx, M. Metal mixtures and kidney function: An application of machine learning to NHANES data. *Environ. Res.* **2020**, *191,* 110126. doi: 10.1016/j.envres.2020.110126

211. Liu, M.; Li, M.; Guo, W.; Zhao, L.; Yang, H.; Yu, J.; Liu, L.; Fang, Q.; Lai, X.; Yang, L.; Zhu, K.; Dai, W.; Mei, W.; Zhang, X. Co-exposure to priority-controlled metals mixture and blood pressure in Chinese children from two panel studies. *Environ.Pollut.* **2022**, *306,* 119388. doi: 10.1016/j.envpol.2022.119388

212. Michael, T.; Kohn, E.; Daniel, S.; Hazan, A.; Berkovitch, M.; Brik, A.; Hochwald, O.; Borenstein-Levin, L.; Betser, M.; Moskovich, M.; Livne, A.; Keidar, R.; Rorman, E.; Groisman, L.; Weiner, Z.; Rabin, A.M.; Solt, I.; Levy, A. Prenatal exposure to heavy metal mixtures and anthropometric birth outcomes: A cross-sectional study. *Environ. Health* **2022**, *21,* 139. doi: 10.1186/s12940-022-00950-z

213. Su, F.; Zeeshan, M.; Xiong, L.-H.; Lv, J.-Y.; Wu, Y.; Tang, X.-J.; Zhou, Y.; Ou, Y.-Q.; Huang, W.-Z.; Feng, W.-R.; Zeng, X.-W.; Dong, G.-H. Co-exposure to perfluoroalkyl acids and heavy metals mixtures associated with impaired kidney function in adults: A community-based population study in China. *Sci. Total Environ.* **2022**, *839,* 156299. doi: 10.1016/j.scitotenv.2022.156299

214. Takatani, T.; Eguchi, A.; Yamamoto, M.; Sakurai, K.; Takatani, R.; Taniguchi, Y.; Nakayama, S.F.; Mori, C.; Kamijima, M. Individual and mixed metal maternal blood concentrations in relation to birth size: An analysis of the Japan Environment and Children's Study (JECS). *Environ. Int.* **2022**, *165,* 107318. doi: 10.1016/j.envint.2022.107318

215. Borghese, M.M.; Fisher, M.; Ashley-Martin, J.; Fraser, W.D.; Trottier, H.; Lanphear, B.; Johnson, M.; Helewa, M.; Foster, W.; Walker, M.; Arbuckle, T.E. Individual, independent, and joint associations of toxic metals and manganese on hypertensive disorders of pregnancy: Results from the MIREC Canadian pregnancy cohort. *Environ. Health Perspect.* **2023**, *131,* 47014. doi: 10.1289/EHP10825

216. Chen, Y.; Zhao, A.; Li, R.; Kang, W.; Wu, J.; Yin, Y.; Tong, S.; Li, S.; Chen, J. Independent and combined associations of multiple-heavy-metal exposure with lung function: A population-based study in US children. *Environ. Geochem. Health* **2023**, *45,* 5213–5230. doi: 10.1007/s10653-023-01565-0

217. Gao, H.; Zhu, N.; Deng, S.; Du, C.; Tang, Y.; Tang, P.; Xu, S.; Liu, W.; Shen, M.; Xiao, X.; Yang, F. Combination effect of microcystins and arsenic exposures on CKD: A case-control study in China. *Toxins* **2023**, *15,* 144. doi: 10.3390/toxins15020144

218. Liang, J.; Pu, Y.; Liu, M.; Bao, W.; Zhang, Y.; Hu, L.; Huang, S.; Jiang, N.; Huang, S.; Pu, X.; Dong, G.; Chen, Y. Synergistic impact of co-exposures to whole blood metals on chronic kidney disease

in general US adults: A cross sectional study of the National Health and Nutrition Examination Survey 2011–2020. *Environ. Sci. Pollut. Res. Int.* **2023**, *30,* 113948–113961. doi: 10.1007/s11356-023-30177-5

219. Ma, J.; Geng, S.; Sun, Q.; Zhang, X.; Han, L.; Yao, X.; Zhang, B.; Zhu, L.; Wen, J. Exposure to metal mixtures and young children's growth and development: A biomonitoring-based study in Eastern China. *Ecotoxicol. Environ. Saf.* **2023**, *268,* 115726. doi: 10.1016/j.ecoenv.2023.115726

220. Shen, Z.; Wang, R.; He, P.; Zhang, Z.; Dai, Y.; Li, M.; Liu, Z.; Yang, H.; Guan, S.; Sun, J. Association between urinary metal concentrations and abnormal estimated glomerular filtration rate in Chinese community-dwelling elderly: Exploring the mediating effect of triglycerides. *Ecotoxicol. Environ. Saf.* **2023**, *259,* 114966. doi: 10.1016/j.ecoenv.2023.114966

221. Wu, Z.; Guan, T.; Cai, D.; Su, G. Exposure to multiple metals in adults and diabetes mellitus: A cross-sectional analysis. *Environ. Geochem. Health* **2023**, *45,* 3251–3261. doi: 10.1007/s10653-022-01411-9

222. Yu, Y.; Meng, W.; Kuang, H.; Chen, X.; Zhu, X.; Wang, L.; Tan, H.; Xu, Y.; Ding, P.; Xiang, M.; Hu, G.; Zhou, Y.; Dong, G.-H. Association of urinary exposure to multiple metal(loid)s with kidney function from a national cross-sectional study. *Sci. Total Environ.* **2023**, *882,* 163100. doi: 10.1016/j.scitotenv.2023.163100

223. Zhu, J.; Hu, S.; Wang, S.; Zhang, Y.; Zhu, Q.; Zhang, M.; Shi, Z. Association between metal mixture exposure and abnormal glucose metabolism in multiple mixture exposure models: Evidence from NHANES 2015–2016. *Curr. Res. Toxicol.* **2023**, *5,* 100141. doi: 10.1016/j.crtox.2023.100141

224. An, Q.; Wang, Q.; Liu, R.; Zhang, J.; Li, S.; Shen, W.; Zhou, H.; Liang, Y.; Li, Y.; Mu, L.; Lei, L. Analysis of relationship between mixed heavy metal exposure and early renal damage based on a weighted quantile sum regression and Bayesian kernel machine regression model. J. Trace Elem. Med. Biol. 2024, 84, p. 127438, 2024, doi: 10.1016/j.jtemb.2024.127438

225. Fan, G.; Liu, Q.; Wu, M.; Bi, J.; Qin, X.; Fang, Q.; Mei, S.; Wan, Z.; Lv, Y.; Song, L.; Wang, Y. Association between multiple metal exposure and bone mineral density among Chinese adults. *Environ. Geochem. Health* **2024**, *46,* 475. doi: 10.1007/s10653-024-02261-3

226. Fu, Y.; He, M.; Liu, Y.; Li, M.; Zhu, M.; Wang, Y.; Lin, W.; Yu, L.; Yang, L.; Zhang, Y.; Liu, Y.; Ji, H.; Ding, H.; Wang, J. Reduction of haemoglobin is related to metal mixtures exposure in Chinese preschoolers: Joint effect models. *J. Trace Elem. Med. Biol.* **2024**, *84,* 127427. doi: 10.1016/j.jtemb.2024.127427

227. Huang, J.; Zhang, Y.; King, L.; Wang, J.; Nie, P.; Xie, Q.; Chen, H.; Wan, X.; Li, Z.; Zhao, Y.; Xu, H. Associations of urinary heavy metals with age at menarche, age at menopause, and reproductive lifespan: A cross-sectional study in U.S. women. *Ecotoxicol. Environ. Saf.* **2024**, *283,* 116950. doi: 10.1016/j.ecoenv.2024.116950

228. Kim, I.-G.; Hong, S.; Yim, S.; Jeong, J.-H.; Choi, K.; Lee, J.-H.; Hong, Y.-S.; Eom, S.-Y.; Kim, H.; Kim, Y.-D. Sex-specific effects of combined heavy metal exposure on blood pressure: A Bayesian Kernel Machine Regression analysis. *Atmosphere* **2024**, *15,* 1157. doi: 10.3390/atmos15101157

229. Long, J.; Huang, H.; Tang, P.; Liang, J.; Liao, Q.; Chen, J.; Pang, L.; Yang, K.; Wei, H.; Chen, M.; Wu, X.; Huang, D.; Pan, D.; Liu, S.; Zeng, X.; Qiu, X. Associations between maternal exposure to multiple metals and metalloids and blood pressure in preschool children: A mixture-based approach. *J. Trace Elem. Med. Biol.* **2024**, *84,* 127460. doi: 10.1016/j.jtemb.2024.127460

230. Pan, S.; Niu, Y.; Duan, S.; Zhao, D.; Wang, Q.; Dong, Z.; Cai, G.; Chen, X. Uric acid mediates the relationship between mixed heavy metal exposure and renal function in older adult people. *Front. Public Health* **2024**, *12,* 1403878. doi: 10.3389/fpubh.2024.1403878

231. Qiao, G.; Shen, Z.; Duan, S.; Wang, R.; He, P.; Zhang, Z.; Dai, Y.; Li, M.; Chen, Y.; Li, X.; Zhao, Y.; Liu, Z.; Yang, H.; Zhang, R.; Guan, S.; Sun, J. Associations of urinary metal concentrations with anemia: A cross-sectional study of Chinese community-dwelling elderly. *Ecotoxicol. Environ. Saf.* **2024**, *270,* 115828. doi: 10.1016/j.ecoenv.2023.115828

232. Schildroth, S.; Valeri, L.; Kordas, K.; Shi, B.; Friedman, A.; Smith, D.; Placidi, D.; Wright, R.O.; Lucchini, R.G.; White, R.F.; Horton, M.; Henn, B.C. Assessing the mediating role of iron status on associations between an industry-relevant metal mixture and verbal learning and memory in Italian adolescents. *Sci. Total Environ.* **2024**, *906,* 167435. doi: 10.1016/j.scitotenv.2023.167435

233. Wei, J.; Fu, D.; Guo, S.; Tian, T.; Huang, Y.; Li, Z.; Wang, L.; Jin, L.; Ye, W.; Ren, A.; Yin, S. Elementomics of 32 elements in cord serum depicts the risk of orofacial clefts: A case-control study in Shanxi, China. *Environ. Pollut.* **2024**, *362,* 125037. doi: 10.1016/j.envpol.2024.125037

234. Xiang, Y.; Wang, Y.; Deng, Y.; Wang, T.; Chen, J.; He, M. Independent and joint associations of multiple metals exposure with vital capacity index: A cross-sectional study in Chinese children and adolescents. *Int. Arch. Occup. Environ. Health* **2024**, *97,* 791–801. doi: 10.1007/s00420-024-02085-9

235. Yang, X.; Li, L.; Nie, L. Associations between co-exposure to heavy metals and vertebral compression fracture, as well as femoral neck bone mineral density: A cross-sectional study from NHANES data. *PLoS One* **2024**, *19,* e0303418. doi: 10.1371/journal.pone.0303418

236. Zhou, X.; Jin, H.; Zhang, Y. Urinary metals are associated with obesity in U.S. children and adolescents: A cross-sectional study. *Nutr. Res.* **2024**, *132,* 40–52. doi: 10.1016/j.nutres.2024.09.017

237. Go, Y.-Y.; Hur, Y.M.; You, Y.-A.; Park, S.; Lee, G.; Chae, R.; Kim, S.-M.; Kim, Y.J. Association of maternal multi-metal exposure and dyslipidemia: A study of air pollution on pregnancy outcomes. *BMC Pregnancy Childbirth* **2025**, *25,* 518. doi: 10.1186/s12884-025-07596-y

238. Ji, R.; Wu, H.; Lin, H.; Li, Y.; Shi, Y. Cadmium and selenium blood levels in association with congestive heart failure in diabetic and prediabetic patients: A cross-sectional study from the national health and nutrition examination survey. *Diabetol. Metab. Syndr.* **2025**, *17,* 12. doi: 10.1186/s13098-024-01556-w

239. Rodríguez, D.; Lima, S.M.; Li, C.; Schildroth, S.; Xu, M.; Kordas, K. Associations of a metal mixture and vitamin D with sleep duration among adolescents and young adults from the 2011–2018 NHANES cycles. *J. Trace Elem. Med. Biol.* **2025**, *88,* 127606. doi: 10.1016/j.jtemb.2025.127606

240. Yang, C.; Zhang, J.; Liu, H.; Hong, Q.; Fan, Y.; An, J.; Zhang, H.; Shen, X.; Dong, X. Health effects

of mixed metal exposure on accelerating aging among the elderly population. *Ecotoxicol. Environ. Saf.* **2025**, *291,* 117760. doi: 10.1016/j.ecoenv.2025.117760

241. Yu, M.; Xun, J.; Ge, Y.; Li, X.; Chen, X.; Cui, L.; Wang, X.; Zhang, M.; Xing, Z.; Deng, L.; AiErken, N.J.; Lu, S.; Lei, K. Relationship between internal metal exposure and thyroid cancer incidence: A case-control study simultaneously validated by BKMR and WQS models. *Food Chem. Toxicol.* **2025**, *201,* 115443. doi: 10.1016/j.fct.2025.115443

242. Borghese, M.M.; Liang, C.L.; Owen, J.; Fisher, M. Individual and mixture associations of perfluoroalkyl substances on liver function biomarkers in the Canadian Health Measures Survey. Environ. Health **2022**, *21,* 85. doi: 10.1186/s12940-022-00892-6

243. Guo, J.; Huang, S.; Yang, L.; Zhou, J.; Xu, X.; Lin, S.; Li, H.; Xie, X.; Wu, S. Association between polyfluoroalkyl substances exposure and sex steroids in adolescents: The mediating role of serum albumin. *Ecotoxicol. Environ. Saf.* **2023**, *253,* 114687. doi: 10.1016/j.ecoenv.2023.114687

244. Li, S.; Wang, C.; Yang, C.; Chen, Y.; Cheng, Q.; Liu, J.; Zhang, Y.; Jin, L.; Li, Z.; Ren, A.; Wang, L. Prenatal exposure to poly/perfluoroalkyl substances and risk for congenital heart disease in offspring. *J. Hazard. Mater.* **2024**, *469,* 134008. doi: 10.1016/j.jhazmat.2024.134008

245. Wang, T.; Yang, J.; Han, Y.; Wāng, Y. Unveiling the intricate connection between per- and polyfluoroalkyl substances and prostate hyperplasia. *Sci. Total Environ.* **2024**, *932,* 173085. doi: 10.1016/j.scitotenv.2024.173085

246. Abuduxukuer, K.; Wang, H.; Wang, C.; Luo, X.; Zeng, X.; Da, D.; Yu, J.; Lu, W.; Zhang, J.; Zhang, Y.; Luo, J.; Zhang, H. Prenatal exposure to per-and polyfluoroalkyl substances and its association with Developmental Defects of Enamel (DDE) and dental caries in 4 years old children: Findings from Shanghai birth cohort. *Environ. Int.* **2025**, *198,* 109411. doi: 10.1016/j.envint.2025.109411

247. Borghese, M.M.; Feng, J.; Liang, C.L.; Kienapple, N.; Manz, K.E.; Fisher, M.; Arbuckle, T.E.; Atlas, E.; Braun, J.M.; Bouchard, M.F.; Foster, W.; Ashley-Martin, J. Legacy, alternative, and precursor PFAS and associations with lipids and liver function biomarkers: Results from a cross-sectional analysis of adult females in the MIREC-ENDO study. *Int. J. Hyg. Environ. Health* **2025**, *267,* 114592. doi: 10.1016/j.ijheh.2025.114592

248. Liao, Q.; Liang, X.; Huang, H.; Tang, P.; Liang, J.; Long, J.; Ou, L.; Wen, J.; Sheng, Y.; Li, H.; Qiu, X. Association between prenatal exposure to per- and polyfluoroalkyl substances and blood pressure among preschool-aged children: The moderating effect of child-age and the mediating effect of inflammatory cytokine. *Ecotoxicol. Environ. Saf.* **2025**, *302,* 118615. doi: 10.1016/j.ecoenv.2025.118615

249. Liu, Y.; Yao, J.; Ren, M.; Ye, L.; Pan, A.-P.; Xu, X. Association of per-and polyfluoroalkyl substance exposure with cataract prevalence among U.S. adults: A NHANES analysis (2005–2008). *Transl. Vis. Sci. Technol.* **2025**, *14,* 1. doi: 10.1167/tvst.14.4.1

250. Yang, L.X.; Zhai, J.; Chen, Z.J.; Du, Y. Female-specific associations of serum perfluoroalkyl and polyfluoroalkyl substances with sex hormonal/insulin dysregulation: An integrated population-based study. *Ecotoxicol. Environ. Saf.* **2025**, *298,* 118273. doi: 10.1016/j.ecoenv.2025.118273

251. Hou, J.; Yin, W.; Li, P.; Huang, Y.; Wan, Y.; Hu, C.; Xu, T.; Cheng, J.; Wang, L.; Yu, Z.; Yuan, J. Effect of exposure to phthalates on association of polycyclic aromatic hydrocarbons with 8-hydroxy-2′-deoxyguanosine. *Sci. Total Environ.* **2019**, *691,* 378–392. doi: 10.1016/j.scitotenv.2019.07.113

252. Kang, X.; Li, J.; Luo, J.; Zhang, D. Associations between organophosphate esters metabolites and sleep disorder and trouble sleeping in adults: A machine-learning approach. *Environ. Sci. Pollut. Res. Int.* **2022**, *29,* 67287–67300. doi: 10.1007/s11356-022-20596-1

253. Cano-Sancho, G.; Warembourg, C.; Güil, N.; Stratakis, N.; Lertxundi, A.; Irizar, A.; Llop, S.; Lopez-Espinosa, M.J.; Basagaña, X.; González, J.R.; Coumoul, X.; Fernández-Barrés, S.; Antignac, J.P.; Vrijheid, M.; Casas, M. Nutritional modulation of associations between prenatal exposure to persistent organic pollutants and childhood obesity: A prospective cohort study. *Environ. Health Perspect.* **2023**, *131,* 037011. doi: 10.1289/EHP11258

254. Hu, P.; Ke, S.; Vinturache, A.; Chen, Y.; Ding, G.; Zhang, Y. Organophosphate esters, airway inflammation, and lung function among U.S. participants: A nationally representative cross-sectional study from NHANES, 2011–2012. *Sci. Total Environ.* **2023**, *892,* 164755. doi: 10.1016/j.scitotenv.2023.164755

255. Deng, F.; He, J.; Dai, Y.; Peng, R.; Pan, X.; Yuan, J.; Tan, L. Biomonitoring urinary pesticide metabolites in preschool children by supported liquid extraction and ultra-high performance liquid chromatography-tandem mass spectrometry and their association with oxidative stress. *J. Chromatogr. A* **2024**, *1725,* 464944. doi: 10.1016/j.chroma.2024.464944

256. Deng, Y.; Yi, S.; Liu, W.; Yang, L.; Zhu, L.; Zhang, Q.; Jin, H.; Yang, R.; Wang, R.; Tang, N.-J. Identification of primary organophosphate esters contributing to enhanced risk of gestational diabetes mellitus based on a case-control study. *Environ. Sci. Technol.* **2024**, *58,* 17532–17542. doi: 10.1021/acs.est.4c04180

257. Wu, X.; Liu, Q.; Li, Y.; Yue, M.; Su, Q.; Luo, J.; Li, Y.; Zeng, S.; Gao, J. Urinary neonicotinoid concentrations and obesity: A cross-sectional study among Chinese adolescents. *Environ. Pollut.* **2024**, *345,* 123516. doi: 10.1016/j.envpol.2024.123516

258. Duan, S.; Wu, Y.; Zhu, J.; Wang, X.; Fang, Y. Associations of polycyclic aromatic hydrocarbons mixtures with cardiovascular diseases mortality and all-cause mortality and the mediation role of phenotypic ageing: A time-to-event analysis. *Environ. Int.* **2024**, *186,* 108616. doi: 10.1016/j.envint.2024.108616

259. Lu, X.; Zhou, Y.; Miao, Q.; Han, X.; Zhou, Y.; Zhao, G.; Yu, H.; Chen, M. Independent and joint associations between urinary polycyclic aromatic hydrocarbon metabolites and cognitive function in older adults in the United States. *Front. Public Health* **2024**, *12,* 1392813. doi: 10.3389/fpubh.2024.1392813

260. Pi, X.; Liu, C.; Jia, X.; Zhang, Y.; Liu, J.; Wang, B.; Wang, L.; Li, Z.; Ren, A.; Jin, L. Periconceptional polycyclic aromatic hydrocarbon levels in maternal hair and fetal risk for congenital heart defects. *Ecotoxicol. Environ. Saf.* **2024**, *286,* 117251. doi: 10.1016/j.ecoenv.2024.117251

261. Wei, J.; Wang, Y.; Kong, H.; Wu, J.; Jiang, L.; Pan, B.; Guo, S.; Yang, F.; Liu, G.; Qiu, F.; Guo, J.;

Zhang, Y.; Nie, J.; Yang, J. Association between plasma CC16 levels and lung function changes in coke oven workers: A cohort study from 2014 to 2023. *Ecotoxicol. Environ. Saf.* **2024**, *284,* 117002. doi: 10.1016/j.ecoenv.2024.117002

262. Wu, L.; Lu, X.; Zhang, S.; Zhong, Y.; Gao, H.; Tao, F.; Wu, X. Co-exposure effects of urinary polycyclic aromatic hydrocarbons and metals on lung function: Mediating role of systematic inflammation. *BMC Pulm. Med.* **2024**, *24,* 386. doi: 10.1186/s12890-024-03173-9

263. Zang, X.; Zhou, W.; Zhang, H.; Zang, X. Using four machine learning methods to analyze the association between polycyclic aromatic hydrocarbons and visual impairment in American adults: Evidence from NHANES. *Toxics* **2024**, *12,* 789. doi: 10.3390/toxics12110789

264. Zhang, L.; Yang, X. Association between exposure to polycyclic aromatic hydrocarbons and endometriosis: Data from the NHANES 2001–2006. *Front. Public Health* **2024**, *11,* 1267124. doi: 10.3389/fpubh.2023.1267124

265. Liu, W.; Cao, S.; Shi, D.; Yu, L.; Qiu, W.; Chen, W.; Wang, B. Single-chemical and mixture effects of multiple volatile organic compounds exposure on liver injury and risk of non-alcoholic fatty liver disease in a representative general adult population. *Chemosphere* **2023**, *339,* 139753. doi: 10.1016/j.chemosphere.2023.139753

266. Wei, C.; Cao, L.; Zhou, Y.; Zhang, W.; Zhang, P.; Wang, M.; Xiong, M.; Deng, C.; Xiong, Q.; Liu, W.; He, Q.; Guo, Y.; Shao, Z.; Chen, X.; Chen, Z. Multiple statistical models reveal specific volatile organic compounds affect sex hormones in American adult male: NHANES 2013–2016. *Front. Endocrinol.* **2023**, *13,* 1076664. doi: 10.3389/fendo.2022.1076664

267. Wang, Y.; Meng, Z.; Wei, S.; Li, X.; Su, Z.; Jiang, Y.; Wu, H.; Pan, H.; Wang, J.; Zhou, Q.; Qiao, Y.; Fan, Y. Urinary volatile organic compound metabolites and COPD among US adults: Mixture, interaction and mediation analysis. *Environ. Health* **2024**, *23,* 45. doi: 10.1186/s12940-024-01086-y

268. Liu, H.; Chen, Z.; Xiang, R.; Liu, Y. Independent and combined effects of volatile organic compounds on sarcopenia: Insights into environmental pollutants and muscle health. *Ecotoxicol. Environ. Saf.* **2025**, *298,* 118344. doi: 10.1016/j.ecoenv.2025.118344

269. Fang, Y.; Zhang, J. The cumulative and single effect of 12 aldehydes concentrations on cardiovascular diseases: An analysis based on Bayesian kernel machine regression and weighted logistic regression. *Rev. Cardiovasc. Med.* **2024**, *25,* 206. doi: 10.31083/j.rcm2506206

270. Ge, L.; Liu, J.; Kang, X.; Wang, W.; Zhang, D. Association of serum individual and mixed aldehydes with depressive symptoms in the general population: A machine learning study. *J. Affect. Disord.* **2024**, *345,* 8–17. doi: 10.1016/j.jad.2023.10.123

271. Tang, J.; Chen, Y.; Xue, P.; Chen, Y.; Kong, H.; Lin, C.; Wang, X.; Liu, S. Exposure to synthetic steroid hormones and precocious puberty in girls: A case-control study. *Ecotoxicol. Environ. Saf.* **2024**, *283,* 116814. doi: 10.1016/j.ecoenv.2024.116814

272. Xiao, F.; Wei, Y.; Zou, P.; Wu, X. Associations between single and combined exposures to environmental phenols and ulcerative colitis in American adults. *Clin. Res. Hepatol. Gastroenterol.* **2024**, *48,* 102468. doi: 10.1016/j.clinre.2024.102468

273. Su, H.; Xi, J.; Miao, M.; Liang, H.; Chen, Y.; Wang, Z.; Zhou, Y.; Jin, Y.; Ji, H.; Yuan, W. Bisphenol analogs exposure in 4-year-old children and their intelligence quotient at 6 years: A prospective cohort study. *Environ. Res.* **2025**, *276,* 121528. doi: 10.1016/j.envres.2025.121528

274. Zhao, Z.; Zhang, C.; Li, Y.; Liu, J.; Wang, L.; Wang, X.; Wang, Y.; Liu, M.; Yue, X.; Wang, X.; Wang, Y.; Ji, L.; Zhao, X.; Li, D. Association between exposure to brominated flame retardants and atherosclerosis: Evidence for inflammatory status as a potential mediator. *Sci. Total Environ.* **2025**, *967,* 178822. doi: 10.1016/j.scitotenv.2025.178822

275. Tanner, E.M.; Hallerbäck, M.U.; Wikström, S.; Lindh, C.; Kiviranta, H.; Gennings, C.; Bornehag, C.-G. Early prenatal exposure to suspected endocrine disruptor mixtures is associated with lower IQ at age seven. *Environ. Int.* **2020**, *134,* 105185. doi: 10.1016/j.envint.2019.105185

276. Wu, B.; Jiang, Y.; Jin, X.; He, L. Using three statistical methods to analyze the association between exposure to 9 compounds and obesity in children and adolescents: NHANES 2005-2010. *Environ. Health* **2020**, *19,* 94. doi: 10.1186/s12940-020-00642-6

277. Hu, J.M.Y.; Arbuckle, T.E.; Janssen, P.; Lanphear, B.P.; Zhuang, L.H.; Braun, J.M.; Chen, A.; McCandless, L.C. Prenatal exposure to endocrine disrupting chemical mixtures and infant birth weight: A Bayesian analysis using kernel machine regression. *Environ. Res.* **2021**, *195,* 110749. doi: 10.1016/j.envres.2021.110749

278. Kim, B.; Park, B.; Kim, C.H.; Kim, S.; Park, B. Association between endocrine-disrupting chemical mixture and metabolic indices among children, adolescents, and adults: A population-based study in Korea. *Environ. Pollut.* **2022**, *315,* 120399. doi: 10.1016/j.envpol.2022.120399

279. Midya, V.; Alcala, C.S.; Rechtman, E.; Gregory, J.K.; Kannan, K.; Hertz-Picciotto, I.; Teitelbaum, S.L.; Gennings, C.; Rosa, M.J.; Valvi, D. Machine learning assisted discovery of interactions between pesticides, phthalates, phenols, and trace elements in child neurodevelopment. *Environ. Sci. Technol.* **2023**, *57,* 18139–18150. doi: 10.1021/acs.est.3c00848

280. Jang, H.; Choi, K.-H.; Cho, Y.M.; Han, D.; Hong, Y.S. Environmental risk score of multiple pollutants for kidney damage among residents in vulnerable areas by occupational chemical exposure in Korea. *Environ. Sci. Pollut. Res. Int.* **2024**, *31,* 35938–35951. doi: 10.1007/s11356-024-33567-5

281. Li, R.; Lin, X.; Lu, T.; Wang, J.; Wang, Y.; Xu, L. Associations between exposure to multiple environmental chemicals and metabolic syndrome: A mixture analysis. *Hyg. Environ. Hlth Adv.* **2024**, *12,* 100112. doi: 10.1016/j.heha.2024.100112

282. Midya, V.; Gennings, C. Detecting shape-based interactions among environmental chemicals using an ensemble of exposure-mixture regression and interpretable machine learning tools. *Stat. Biosci.* **2024**, *16,* 395–415. doi: 10.1007/s12561-023-09405-6

283. Alampi, J.D.; Lanphear, B.P.; Macfarlane, A.J.; Braun, J.M.; Oulhote, Y.; Ashley-Martin, J.; Arbuckle, T.E.; Chen, A.; Muckle, G.; Mccandless, L.C. Association between gestational environmental chemical mixtures and folate exposures with autistic behaviors in a Canadian birth cohort. *Environ. Epidemiol.* **2025**, *9,* e402. doi: 10.1097/EE9.0000000000000402

284. Guo, L.-C.; Zhu, P.; Gui, C.; Deng, J.; Gao, Y.; Long, C.; Zhang, H.; Lv, Z.; Yu, S. Disrupting effects of neonicotinoids and their interaction with metals on thyroid hormone, an evidence of children in a rural area, South China. *Ecotoxicol. Environ. Saf.* **2025**, *290,* 117788. doi: 10.1016/j.ecoenv.2025.117788

285. Haruna, I.; Obeng-Gyasi, E. Combined effect of per- and polyfluoroalkyl substances, toxic metals, and essential elements on chronic kidney disease. *Pollutants* **2025**, *5,* 12. doi: 10.3390/pollutants5020012

286. Huang, S.; Li, Z.; Wu, C.; Zhao, X.; Xiong, J.; Xiao, W.; Su, H.; Zheng, R.; Xu, Z.; Su, Q.; Lu, X.; Wang, Q.; Su, Z. Divergent effects of EDCs on bone maturation: Role of body mass index and puberty. *Ecotoxicol. Environ. Saf.* **2025**, *302,* 118525. doi: 10.1016/j.ecoenv.2025.118525

287. Jehu-Appiah, D.; Obeng-Gyasi, E. Combined Effect of metals, PFAS, phthalates, and plasticizers on cardiovascular disease risk. *Toxics* **2025**, *13,* 476. doi: 10.3390/toxics13060476

288. Puvvula, J.; Hwang, W-T.; McCandless, L.; Xie, C.; Braun, J.M.; Vuong, A.M.; Oulhote, Y.; Schisterman, E.F.; Shinohara, R.T.; Booij, L.; Bouchard, M.F.; Linn, K., Borghese, M.M.; Seguin, J.R.; Zidek, A.; Till, C.; Fraser, W.; Yolton, K.; Cecil, K.M.; Ashley-Martin, J.; Arbuckle, T.E.; Lanphear, B.; Chen, A. Gestational exposure to environmental chemical mixtures and cognitive abilities in children: A pooled analysis of two North American birth cohorts. *Environ. Int.* **2025**, *196,* 109298. doi: 10.1016/j.envint.2025.109298

Results of International Scan Survey

Q1. Please select the option that best describes your program. (required – check one)

Category	Frequency (%)
National-level	17 (56.7)
Provincial-level	2 (6.7)
State-level	1 (3.3)
Other	9 (30.0)
Prefer not to answer	1 (3.3)
Total	30

Q2. What is your current role? (required – check all that apply)

Category	Frequency
Principal investigator	18
Research scientist	12
Data analyst	4
Other	5
Prefer not to answer	0

Q3. How long have you been involved in human biomonitoring? (required – check one)

Category	Frequency (%)
<1 year	1 (3.3)
1-3 years	2 (6.7)
3-5 years	4 (13.3)
>5 years	23 (76.7)
Prefer not to answer	0
Total	30

Q4. In which areas of human biomonitoring do you primarily conduct research? (required – check all that apply)

Category	Frequency
Environmental exposures	25
Chemical risk assessment	15
Epidemiology	21
Toxicology	7
Other	1
Prefer not to answer	0

Q5. What types of data do you commonly use in your biomonitoring research? (required – check all that apply)

Category	Frequency
Chemical concentrations in blood, urine or other matrices	28
Biomarker data (e.g., metabolomics, proteomics, genomics)	10
Physiological or clinical endpoint data	15
Demographic and lifestyle data	26
Other	0
Prefer not to answer	0

Q6. How would you describe the size and complexity of your biomonitoring datasets? (required – check one)

Category	Frequency (%)
Small and limited (e.g., <100 participants and <10 variables)	5 (16.7)
Moderate (e.g., 100–1,000 participants, 10–50 variables)	12 (40.0)
Large and complex (e.g., >1,000 participants, >50 variables)	10 (33.3)
Other	3 (10.0)
Prefer not to answer	0
Total	30

Q7. How would you rate your familiarity with artificial intelligence or machine learning methodologies? (required – check one)

Category	Frequency (%)
None	3 (30.0)
Beginner	18 (60.0)
Intermediate	7 (23.3)
Advanced	2 (6.7)
Prefer not to answer	0
Total	30

Q8. Has your program implemented artificial intelligence or machine learning methods in the collection or analysis of biomonitoring data? (required)

Category	Frequency (%)
Yes	8 (26.7)
No	22 (73.3)
Prefer not to answer	0
Total	30

Q9. Does your program plan to implement or expand the use of artificial intelligence or machine learning in biomonitoring over the next 1-2 years? (required)

**Category**	**Frequency (%)**
Yes	13 (43.3)
No	8 (26.7)
Unsure	9 (30.0)
Prefer not to answer	0
Total	30

Q10. Is your program currently exploring new artificial intelligence or machine learning technologies for biomonitoring that have not yet been implemented? (required)

**Category**	**Frequency (%)**
Yes	3 (10.0)
No	26 (86.7)
Prefer not to answer	1 (3.3)
Total	30

Q11. Is there a specific artificial intelligence or machine learning technology or approach you believe holds the most potential for improving biomonitoring in the future? (required)

**Category**	**Frequency (%)**
Yes	5 (16.7)
No	4 (13.3)
Unsure	21 (70.0)
Prefer not to answer	0
Total	30

Q12. Are you collaborating with any external organizations for artificial intelligence or machine learning biomonitoring projects (e.g., research institutions, private companies)? (required)

**Category**	**Frequency (%)**
Yes	3 (10.0)
No	25 (83.3)
Prefer not to answer	2 (6.7)
Total	30

Q13. Are there any external factors that might increase the urgency for adopting artificial intelligence or machine learning technologies in your program's biomonitoring efforts? (required)

Category	Frequency (%)
Yes	7 (23.3)
No	20 (66.7)
Prefer not to answer	3 (10.0)
Total	30

Q14. Do you think artificial intelligence or machine learning technology has the potential to improve the future of biomonitoring practices? (required)

Category	Frequency (%)
Yes	22 (73.3)
No	0
Unsure	8 (26.7)
Prefer not to answer	0
Total	30

Q15. What do you see as the primary barriers to implementing artificial intelligence or machine learning in your program's biomonitoring efforts? (required – check all that apply)

Category	Frequency
Lack of funding	17
Lack of technical expertise	24
Limited computational resources	15
Lack of training opportunities	13
Data limitations (e.g., poor quality, missing data, noise)	12
Difficulty interpreting model outputs	10
Organizational priorities	6
Regulatory or ethical concerns	7
Other	2
Prefer not to answer	2

The following questions (Q16-30) were presented to respondents who indicated they had implemented AI/ML ("yes" to Q8).

Q16. Does your program have dedicated personnel or teams trained in artificial intelligence/machine learning methods for biomonitoring purposes? (required)

Category	Frequency (%)
Yes	3 (37.5)
No	4 (50.0)
Prefer not to answer	1 (12.5)
Total	8

Q17. What are your main objectives when applying artificial intelligence/machine learning methods? (required – check all that apply)

Category	Frequency
Pattern detection in exposure data	6
Identification of exposure subgroups/population clusters	4
Prediction of health outcomes or risk levels	3
Interpretation of exposure-response relationships	7
Integration of multi-omics data	5
Other	0
Prefer not to answer	0

Q18. Which artificial intelligence/machine learning methods have been implemented in your program? Briefly describe their primary applications (e.g., exposure modeling, exposure-outcome associations, etc.). If you do not want to provide a response, please indicate "prefer not to answer". (required – free-text)

Most respondents opted for supervised ML approaches, with many mentioning the Least Absolute Shrinkage and Selection Operator [LASSO], Support Vector Machines (SVM), Bayesian Kernel Machine Regression (BKMR), random forest, regression trees and quantile-based g-computation. Unsupervised ML methods, such as k-means, Principal Component Analysis (PCA), Partial Least Squares (PLS), dimensionality reduction and other methods, such as pattern detection (general) and Whole Genome Sequencing (WGS), although less common, were also methods mentioned by respondents.

Exposure–outcome associations were mentioned in more than three responses as a common primary application. Supervised ML methods were also used to examine dose-response functions, handle multiple chemicals at once, select important predictors, and analyze chemical mixtures. The listed unsupervised ML or pattern detection methods were cited to be used for exploratory data analysis and latent group identification, while dimensionality reduction was most likely used to visualise complex datasets.

Q19. What software frameworks or platforms do you use for artificial intelligence/machine learning analyses? (required – check all that apply)

Category	Frequency
Python libraries (e.g., scikit-learn, TensorFlow, PyTorch)	2
R packages (e.g., caret)	7
Commercial software (e.g., SAS, SPSS)	5
Cloud platforms (e.g., AWS, Google Cloud ML, Azure ML)	1
Other	2
Prefer not to answer	0

Q20. What types of data do you use in the artificial intelligence/machine learning methods? (required – check all that apply)

Category	Frequency
Quantitative data	8
Images	2
Text	2
Other	0
Prefer not to answer	0

21. Are you integrating multiple data types (e.g., chemical exposures, omics data, clinical outcomes) into a single analysis? (required)

Category	Frequency (%)
Yes, regularly	5 (62.5)
Yes, occasionally	1 (12.5)
Not currently, but plan to	1 (12.5)
No	1 (12.5)
Prefer not to answer	0
Total	8

22. How significant are data cleaning, harmonization, and preprocessing efforts in your workflow? (required)

Category	Frequency (%)
Minimal effort required	0
Moderate effort required (manageable with standard tools)	4 (50.0)
Significant effort required (a major challenge in most projects)	4 (50.0)
Unsure	0
Prefer not to answer	0
Total	8

Q23. Are your artificial intelligence/machine learning applications capable of real-time analysis, prediction or decision-making? (required)

Category	Frequency (%)
Yes	2 (25.0)
No	4 (50.0)
Prefer not to answer	2 (50.0)
Total	8

Q24. How often does your program review or update its artificial intelligence/machine learning models for biomonitoring to ensure they remain effective? (required)

Category	Frequency (%)
Quarterly	1 (12.5)
Annually	0
As needed	4 (50.0)
We do not currently update AI/ML models	2 (25.0)
Other	0
Prefer not to answer	1 (12.5)
Total	8

Q25. How does your program handle issues of data quality or gaps in data when using artificial intelligence/machine learning for biomonitoring? If you do not want to provide a response, please indicate "prefer not to answer". (required – free-text))

3 of the 8 respondents provided responses. One respondent emphasized using descriptive statistics to assess missingness and summarize data before applying ML, while another reported actively replacing missing data. The third respondent chose to exclude low-quality data.

Q26. How do you ensure that ethical concerns, such as data privacy and the protection of sensitive data, are addressed in artificial intelligence/machine learning applications for biomonitoring? If you do not want to provide a response, please indicate "prefer not to answer". (required – free-text)

4 respondents answered this question. One respondent mentioned using de-identified data, while another specified anonymizing data and working with aggregated data. The third respondent used secure platforms supported by their institution to manage and analyze data. The fourth respondent cited that their institution has a policy for AI use and privacy.

Q27. What challenges has your program faced in implementing artificial intelligence/machine learning for biomonitoring? If you do not want to provide a response, please indicate "prefer not to answer". (required – free-text)

> 4 respondents answered this question. The responses fell under themes of technical infrastructure and access limitations, data complexity and quality, lack of standardized protocols across datasets, issues with handling detection limits and interpretation and uncertainty in ML outputs.

Q28. Have you observed measurable improvements in outcomes (e.g., accuracy, efficiency) due to artificial intelligence/machine learning applications? (required)

Category	Frequency (%)
Yes	0
No	0
Unsure	8 (100)
Prefer not to answer	0
Total	8

Q29. What area(s) in your biomonitoring program have benefitted the most from the implementation of artificial intelligence/machine learning methods? (required – check all that apply)

Category	Frequency
Data collection	2
Data management	2
Data analysis	7
Knowledge dissemination	2
None	1
Other	0
Prefer not to answer	0

Q30. Please provide any citations or links to reports, case studies, and research that your program has produced that applies artificial intelligence/machine learning methods in biomonitoring? (required)

Answers suppressed to maintain confidentiality.
